# Temporal and Gene Reassortment Analysis of Influenza C Virus Outbreaks in Hong Kong, SAR, China

**DOI:** 10.1128/jvi.01928-21

**Published:** 2022-02-09

**Authors:** Rodney S. Daniels, Monica Galiano, Burcu Ermetal, Jasmine Kwong, Chi S. Lau, Zheng Xiang, John W. McCauley, Janice Lo

**Affiliations:** a Worldwide Influenza Centre, The Francis Crick Institute, London, United Kingdom; b Centre for Health Protection, Department of Health, Hong Kong SAR, China; Cornell University

**Keywords:** influenza C virus, outbreak surveillance, genome sequencing, reassortment

## Abstract

From 2014 to week 07/2020 the Centre for Health Protection in Hong Kong conducted screening for influenza C virus (ICV). A retrospective analysis of ICV detections to week 26/2019 revealed persistent low-level circulation with outbreaks occurring biennially in the winters of 2015 to 2016 and 2017 to 2018 (R. S. Daniels et al., J Virol 94:e01051-20, 2020, https://doi.org/10.1128/JVI.01051-20). Here, we report on an outbreak occurring in 2019 to 2020, reinforcing the observation of biennial seasonality in Hong Kong. All three outbreaks occurred in similar time frames, were subsequently dwarfed by seasonal epidemics of influenza types A and B, and were caused by similar proportions of C/Kanagawa/1/76 (K)-lineage and C/São Paulo/378/82 S1- and S2-sublineage viruses. Ongoing genetic drift was observed in all genes, with some evidence of amino acid substitution in the hemagglutinin-esterase-fusion (HEF) glycoprotein possibly associated with antigenic drift. A total of 61 ICV genomes covering the three outbreaks were analyzed for reassortment, and 9 different reassortant constellations were identified, 1 K-lineage, 4 S1-sublineage, and 4 S2-sublineage, with 6 of these being identified first in the 2019–1920 outbreak (2 S2-lineage and 4 S1-lineage). The roles that virus interference/enhancement, ICV persistent infection, genome evolution, and reassortment might play in the observed seasonality of ICV in Hong Kong are discussed.

**IMPORTANCE** Influenza C virus (ICV) infection of humans is common, with the great majority of people being infected during childhood, though reinfection can occur throughout life. While infection normally results in “cold-like” symptoms, severe disease cases have been reported in recent years. However, knowledge of ICV is limited due to poor systematic surveillance and an inability to propagate the virus in large amounts in the laboratory. Following recent systematic surveillance in Hong Kong SAR, China, and direct ICV gene sequencing from clinical specimens, a 2-year cycle of disease outbreaks (epidemics) has been identified, with gene mixing playing a significant role in ICV evolution. Studies like those reported here are key to developing an understanding of the impact of influenza C virus infection in humans, notably where comorbidities exist and severe respiratory disease can develop.

## INTRODUCTION

While influenza C virus (ICV) was first isolated in 1947 from patients in the United States (1), it has been considered a pathogen of low significance, as it generally causes mild disease or clinically inapparent infection (2–4), but in recent years it has been associated with severe disease in children with lower respiratory tract infections (4–10). Seroprevalence studies for ICV in humans have shown high rates worldwide (between 57 and 100%, being lower in children and rising in adulthood) following initial exposure (infection) during childhood, with recurrent infection occurring in both children and adults (11).

Despite these high seroprevalence rates, taken as an indicator of widespread infection, epidemiologic and virologic studies have been limited because long-term monitoring/surveillance of ICV had been conducted in Japan only (12) and because of generally poor ICV isolation or propagation to high titer in hens’ eggs (13) and mammalian cell-lines (14). Studies that have been performed largely relate to retrospective research-related projects, rather than systematic surveillance, focusing on ICV detections and genetic characterization of the hemagglutinin-esterase (HE) gene that encodes the hemagglutinin-esterase-fusion (HEF) glycoprotein, which combines the functions of the hemagglutinin (HA) and neuraminidase (NA) of influenza type A and B viruses and is the major antigenic component of ICVs (15). The other six RNA segments of the ICV genome encode proteins of the polymerase complex (three polymerases, PB2, PB1, P3, and NP [nucleoprotein]), while the matrix (CM) and nonstructural (NS) genes encode two proteins each, CM1 and CM2, and NS1 and NS2, respectively; their genetics and functions have been reviewed (15, 16). Evidence for gene reassortment has been presented for ICVs (12, 13, 17), and biennial ICV epidemics/outbreaks have been identified in Japan (18) and Hong Kong (13), with similar trends having been indicated in studies from other countries as reviewed in Sederdahl and Williams (19).

Here, we provide further evidence of biennial outbreaks occurring in Hong Kong, based on gene-sequencing analyses of ICV-positive clinical specimens collected in 2019 to 2020. Phylogenetic analyses show continued circulation of C/Kanagawa/1/76 lineage and C/São Paulo/378/82 S1- and S2-sublineage viruses with some genetic evolution. Deeper evolutionary analyses using Bayesian evolutionary analysis by sampling trees (BEAST [20]) with a subset of viruses detected in the period of 1950 to 2020, for which complete genome sequences were available, revealed ongoing gene reassortment and detection of new genome compositions. Factors that may contribute to the observed seasonality and genome reassortment are discussed.

## RESULTS

### Detection of ICV infections in Hong Kong.

**FIG 1:**
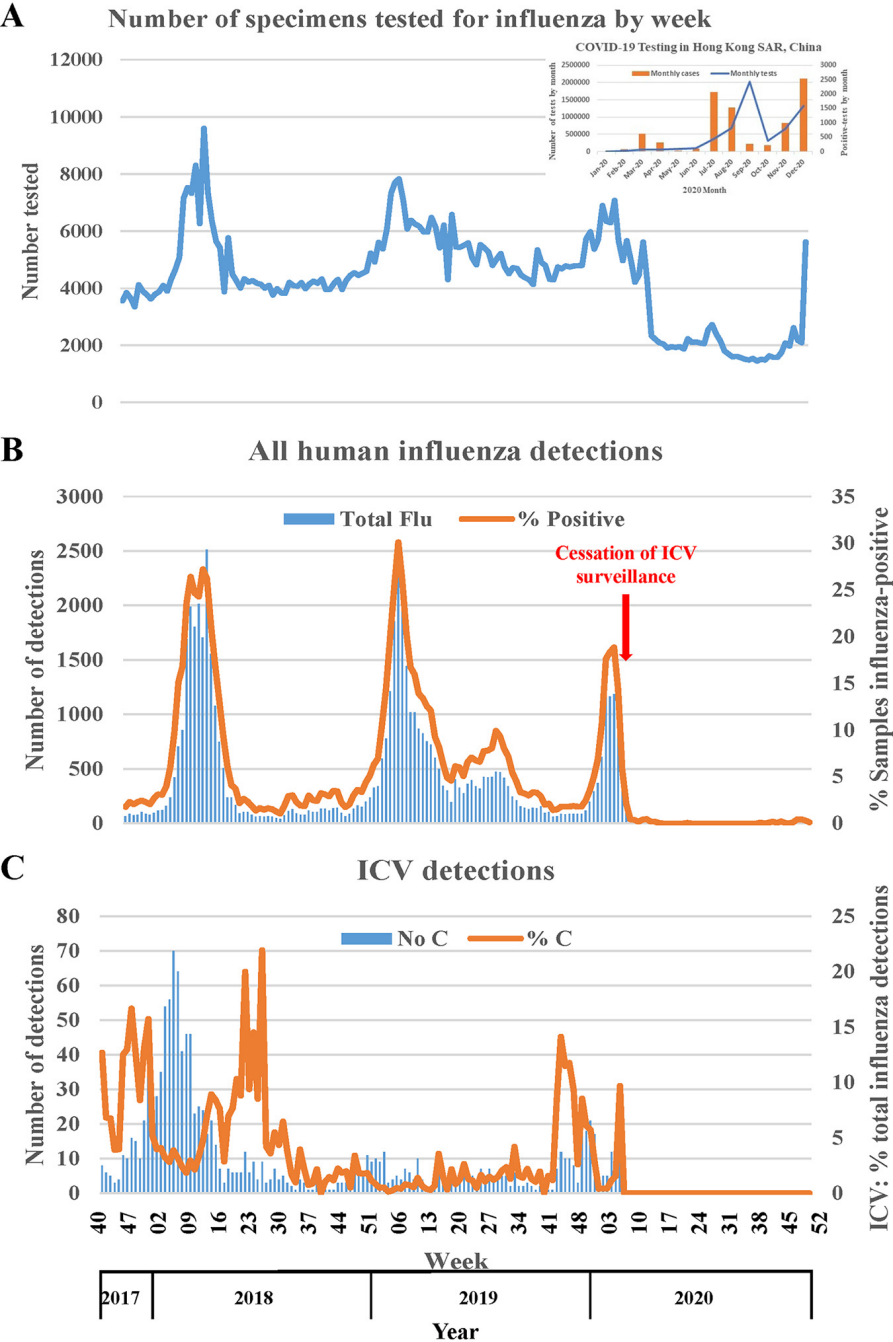
ICV surveillance in Hong Kong weeks 40/2017 to 52/2020. Results of weekly influenza surveillance in Hong Kong covering the period from week 40/2017 to week 52/2020 are shown. (A) numbers of human respiratory specimens tested for influenza type A, B, and C viruses, with the inset showing by month for 2020 the numbers of specimens tested for the COVID-19 agent, SARS-CoV-2, and the numbers of SARS-CoV-2-positive cases detected. (B) Numbers of influenza-positive detections by week and expressed as the percentage of tested specimens showing positivity, with testing for ICV being stopped after week 07/2020. (C) ICV detections by number and as a percentage of the total number of influenza detections by week. All influenza-related data were taken from Flu Express reports published by the Centre for Health Protection, Hong Kong (https://www.chp.gov.hk/en/resources/29/304.html).

As presented previously, over the period of weeks 01/2014 to 26/2019 a total of 1,088,090 specimens were tested for ICV, and a total of 2,394 (0.22%) detections were made, with the great majority being detected in two outbreaks during the 2015–2016 and 2017–2018 influenza seasons, suggestive of 2-year cycling of ICV (13). ICV surveillance was continued until week 07/2020, after which surveillance was restricted to influenza types A and B (seasonal influenza) and increased for the COVID-19 agent, SARS-CoV-2 (Fig. 1).

Figure 1A shows a sharp decline in the numbers of specimens being tested for influenza types A and B after week 07/2020, with levels increasing to more usual numbers toward the end of 2020, which may have been related to a realignment of resources dedicated to testing for disease agents. Notably, over this time frame, while there was considerable variation in the numbers of tests for SARS-CoV-2 being carried out, there was an overall upward trend through the year (Fig. 1A, inset). Variation in SARS-CoV-2 testing was due to a range of testing facilities being utilized and the following numbers of tests performed: Department of Health and Hospital Authority (January to December, *n* = 2,386,137), private testing services (July to December, *n* = 1,116,308), Universal Community Testing Program (September, *n* = 1,783,232), temporary testing centers (October, *n* = 16,802) and community testing centers (November to December, *n* = 451,515), yielding a total of 5,753,994 tests performed, significant numbers of which would have been repeat tests on a number of individuals, especially COVID-19 patients. Variation in the number of positive cases identified per month was observed, but there were only 8,847 laboratory-confirmed SARS-CoV-2 infections throughout 2020.

Figure 1B shows that influenza type A and B detections fell off sharply after week 07/2020 and, as in the rest of the world, remained at very low levels after March 2020. This is presumably a result of nonpharmaceutical intervention: (i) measures introduced to limit the spread of SARS-CoV-2 (e.g., increased use of sanitization and personal protective equipment, social-distancing, restrictions on travel and social/workplace gatherings, also impeding the spread of influenza viruses); (ii) the function of sentinel and nonsentinel sites for influenza surveillance being compromised due to reduced attendance, possible staff shortages, and redistribution of resources to SARS-CoV-2 testing; and (iii) possible viral interference, with SARS-CoV-2 infection either impeding infection by influenza viruses and other respiratory disease agents or limiting the titers of these agents following infection.

Figure 1C specifically shows the ICV detections and shows that following the outbreak in 2017 to 2018 there was a 2-year gap until the recent 2019 to 2020 outbreak. Table 1 shows that all three outbreaks (2015 to 2016, 2017 to 2018, and 2019 to 2020) started at similar times and showed a slight excess of cases among males and similar age distribution of cases, though the duration of the 2019–2020 outbreak is not known, as data are not available beyond week 07/2020 (Fig. 1C). In all three outbreaks, ICV detections represented minorities of the total influenza detections, 661/14,781 (4.5%), 719/19,125 (3.8%), and 158/6,937 (2.3%), respectively, and significant majorities, 72%, 68%, and 67%, respectively, occurred in those aged up to 5 years.

**TABLE 1:** Characteristics of the ICV outbreaks in Hong Kong

Outbreak	Duration^*a*^		No. Of detections	Gender		No. in age range (yrs)^*b*^				
	From wk:	To wk (no. of wks):		Male	Female	≤5	6–15	16–45	46–65	>65
2015–2016	49/2015	27/2016 (32)	661	364	297	474 (71.7)	43 (6.5)	41 (6.2)	40 (6.1)	63 (9.5)
2017–2018	45/2017	15/2018 (23)	719	415	304	486 (67.6)	61 (8.5)	63 (8.8)	44 (6.1)	65 (9.0)
	21/2019–44/2019^*c*^		23^*c*^	13	10	13 (56.5)	3 (13.0)	2 (8.7)	1 (4.4)	4 (17.4)
2019–2020	45/2019	07/2020 (15)^*d*^	87^*d*^	46	41	58 (66.7)	3 (3.5)	13 (14.9)	8 (9.2)	5 (5.7)

a Duration relates to weeks (wks) when there were ≥10 detections/week; number of weeks is shown in parentheses.

b Percentages of the total number of detections are shown in parentheses.

c ICV-positive specimens from 2019 before the start of the 2019–2020 outbreak, those with real-time reverse transcriptase PCR (rtRT-PCR) *C_T_* values of ≤30 (*n* = 23), were shared with WHO CC, London.

d Surveillance for ICV ceased after week 07/2020, by which time 158 ICV detections were recorded over the 15-week period, and 87 of the corresponding clinical specimens, those with rtRT-PCR *C_T_* values of ≤30, were shared with WHO CC, London.

### ICV-positive samples shared with WHO collaborating center (CC), London.

A total of 110 ICV-positive clinical specimens, 23 from weeks 21/2019 to 44/2019, preoutbreak, and 87 from the outbreak (weeks 45/2019 to 07/2020), were shared for genetic characterization (Table 1). The clinical specimens were from 51 females and 59 males and fell across the spectrum of age ranges, in years, up to 5 (*n* = 71), 6 to 15 (*n* = 6), 16 to 45 (*n* = 15), 46 to 65 (*n* = 9), and >65 (*n* = 9). For 101 of the patients, a clinical diagnosis at the time of specimen collection was given, with 15 patients being admitted to hospital for various reasons (e.g., allergic reaction, blood disorders, cancer, cardiac conditions, cellulitis, gastroenterological problems, hemorrhage) without symptoms often related to infection and/or respiratory conditions (see Table S1 in the supplemental material); these patients were in the year age ranges 0 to 5 (*n* = 8), 46 to 65 (*n* = 3), and >65 (*n* = 4). The other 86 patients all presented symptoms often associated with infection, although not all would have fulfilled influenza-like illness (ILI) classification criteria; these patients were in year age ranges 0 to 5 (*n* = 58), 6 to 15 (*n* = 5), 16 to 45 (*n* = 13), 46 to 65 (*n* = 5), and >65 (*n* = 5).

### ICV HE gene sequencing.

HE gene sequences encoding full-length HEF glycoproteins were obtained for 17/23 (74%) clinical specimens from weeks 21/2019 to 44/2019, preoutbreak, and 48/87 (55%) relating to the 2019–2020 outbreak that started around week 45/2019 (Table S1). Phylogenetic analyses of HE gene evolution led to identification of six lineages, Taylor (T), Mississippi (M), Yamagata (Y), Aichi (A), Kanagawa (K), and Sao Paulo (21, 22), with the S-lineage splitting into two sublineages (23) which were subsequently designated S1 and S2 based on analysis of a greater number of sequences and strong bootstrap values (13). These sublineages were defined by specific amino acid substitutions in HEF1, S1 by K323Q and Q358K, and S2 by K345R, with ICVs of both sublineages and the K-lineage having cocirculated during 2014 to 2018. Tables 2 and 3 provide a breakdown of the HE gene sequences we recovered from Hong Kong ICV-positive clinical specimens by clade, patient age group, and time period. The S1-sublineage dominated across all age groups and time periods by a large margin, followed by the S2-sublineage and the K-lineage. However, it was observed that K-lineage viruses showed equal distribution, 33% each, between the ≤5 years and >65 years age groups (Table 2) and, after a period of decline between 2015 and 2019, an upsurge was observed during the 2019–2020 outbreak (Table 3).

**TABLE 2:** Statistics relating to HE gene sequences recovered from ICV-positive clinical specimens from Hong Kong across the entire study period (2015–2020) categorized by clade and patient age group

HE lineage/sublineage	Total no.	Total %^*a*^	Data for age group (yrs)														
			<5			6–15			16–45			46–65			>65		
			No.	Age (%)^*b*^	Clade (%)^*c*^	No.	Age (%)^*b*^	Clade (%)^*c*^	No.	Age (%)^*b*^	Clade (%)^*c*^	No.	Age (%)^*b*^	Clade (%)^*c*^	No.	Age (%)^*b*^	Clade (%)^*c*^
Kanagawa	18	14.4	6	8.8	**33.3**	1	7.7	5.6	3	18.8	16.7	2	22.3	11.1	6	31.6	**33.3**
Sao Paulo 1	81	**64.8**	45	**66.2**	**55.6**	11	**84.6**	13.6	11	**68.8**	13.6	4	**44.4**	4.9	10	**52.6**	12.3
Sao Paulo 2	26	20.8	17	25.0	**65.4**	1	7.7	3.9	2	12.4	7.7	3	33.3	11.5	3	15.8	11.5
**Total**	125	100.0	68	54.4		13	10.4		16	12.7		9	7.1		19	15.1	

a Percentages in each clade based on the total of 125 HE gene sequences obtained.

b Percentages of the total number of detections in each age group.

c Percentages of the total numbers in each clade are shown. Bold type indicates the highest proportion by clade, S1-sublineage in all age groups for ^a^ and ^b^, and age group <5 but for the K-lineage ^c^.

**TABLE 3:** Statistics relating to HE gene sequences recovered categorized by clade and clinical specimen collection time period

HE lineage/sublineage	Total no.	Total %^*a*^	Data for outbreak/period (yrs)							
			2015–2016		2017–2018		2019		2019–2020	
			No.	% total^*b*^	No.	% total^*b*^	No.	% total^*b*^	No.	% total^*b*^
Kanagawa	18	14.4	4	16.0	2	5.0	1	10.0	11	20.0
Sao Paulo 1	81	**64.8**	**17**	**68.0**	**24**	**69.0**	**7**	**70.0**	**33**	**60.0**
Sao Paulo 2	26	20.8	4	16.0	9	26.0	2	20.0	11	20.0
**Total**	125	100.0	25		35		10		55	

a Percentages in each clade based on the total of 125 HE gene sequences obtained.

b Percentages of each clade are shown in relation to the total number of sequences from each time-period. Bold type indicates the highest proportion by clade, S1-sublineage, in all time periods.

**FIG 2:**
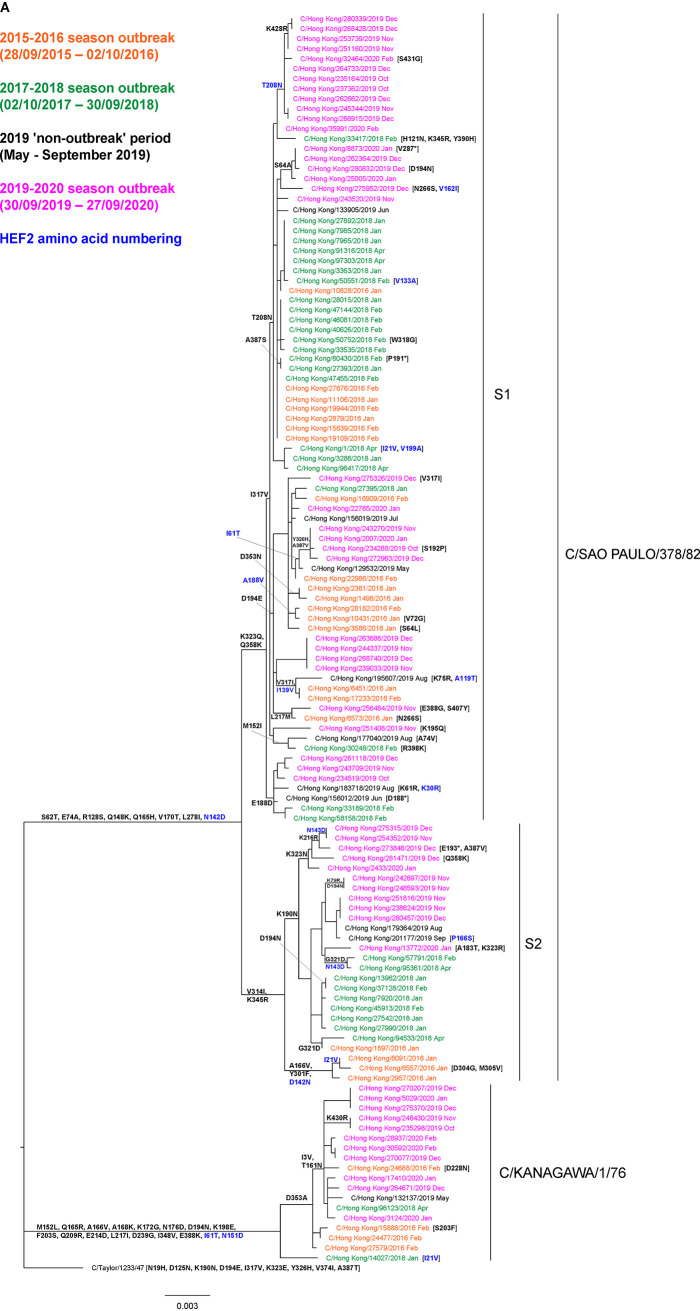
(Continued)

**FIG 3:**
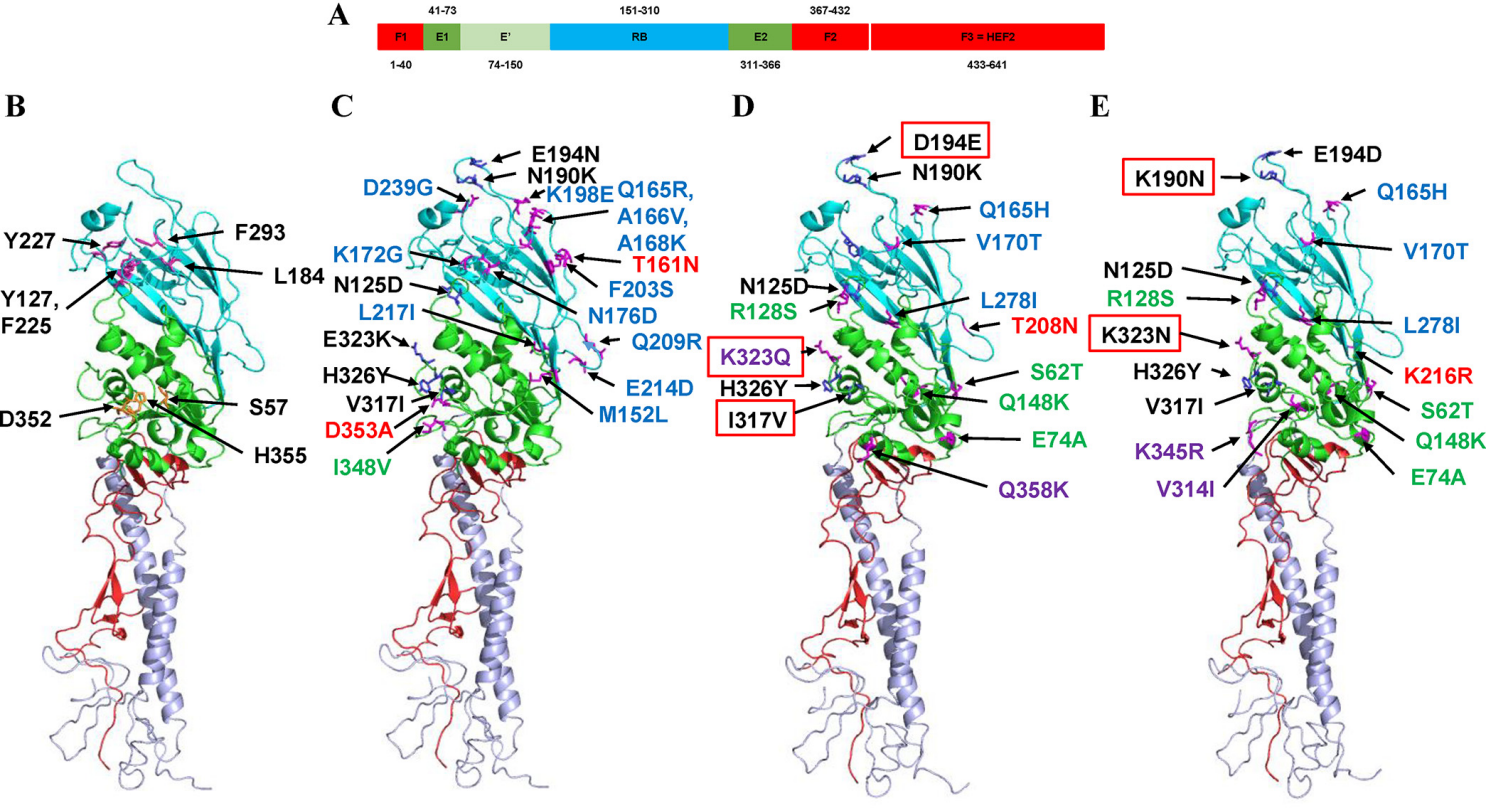
HEF structures indicating the amino acid substitutions that define the currently circulating ICV lineages. The structure of an HEF monomer derived from C/Johannesburg/1/66 (25) was downloaded from the Protein Database (ID: 1FLC) and annotated using PyMOL. (A) The receptor-binding domain (RBD, cyan; HEF1 residues 151 to 310), esterase domain (ED, green; HEF1 residues 41 to 150 and 311 to 366) and fusion domain (FD, red; HEF1 residues 1 to 40 and 367 to 432, and HEF2) are shown as defined previously (15, 25). In panels B to E the N and C termini of HEF1 are shown in red, ED is shown in green, and RBD is shown in cyan, with HEF2 colored mauve. (B) Conserved amino acid residues that define the receptor-binding site (*n* = 5) and esterase catalytic site (*n* = 3) are indicated. (C to E) HEF1 amino acid substitutions in the RBD and ED, compared to HEF of C/Taylor/1233/47, that define (C) the Kanagawa lineage and São Paulo sublineages (D) S1 and (E) S2 are indicated. In cartoons C to E the following amino acid substitutions are shown: (i) those “common” to the Kanagawa and São Paulo sublineages in black, (ii) those defining each lineage in blue (RBD) and green (ED), (iii) those defining the São Paulo sublineages in purple, (iv) those involved in lineage/sublineage evolution (refer to Fig. 2A) in red, and (v) common substitutions that have changed during sublineage evolution in red boxes. Side chains of the relevant amino acid residues in the HEF structure of C/Johannesburg/1/66 are shown as sticks. Y127 and F225 (B) and Q165R, A166V, and A168K (C) cannot be clearly distinguished in the HEF monomer orientation used to produce this figure.

Matsuzaki et al. have identified five neutralization-sensitive epitopes on HEF1 (A1 to A4 and Y1) using a panel of mouse monoclonal antibodies, possibly associated with antigenic drift, involving the following amino acid residues: 125, 172, 173, 175, 192, 193, 235, and 269 (A1); 65, 68, 351, and 353 (A2); 164, 198, 201, and 203 (A3); 212 (A4); and 192, 193, 194, 195, and 198 (Y1) (24). The great majority of these residues fall within the receptor-binding domain (RBD; residues 151 to 310), while those of epitope A2 fall within the esterase domain (ED; residues 41 to 150 and 311 to 366) (25). Figure 2A shows an HE gene phylogeny, rooted against C/Taylor/1233/47 with amino acid substitutions defining various virus groups indicated along the trunk and those relating to individual viruses shown after the virus names, for ICVs from Hong Kong covering the three outbreak periods characterized in this and our previous study (13). Compared to C/Taylor/1233/47 HEF1, the K-lineage is defined by M152L, Q165R, A166V, A168K, K172G, N176D, N190K, E194N, K198E, F203S, Q209R, E214D, L217I, and D239G (*n* = 14) in the RBD and N125D, V317I, E323K, H326Y, and I348V (*n* = 5) in the ED. Conversely, the S-lineage is defined by Q165H, V170T, N190K, E194D, and L278I (*n* = 5) in the RBD and S62T, E74A, N125D, R128S, Q148K, V317I, E323K, and H326Y (*n* = 8) in the ED. Further substitutions in the ED define S1 (K323Q and Q358K) and S2 (V314I and K345R) sublineages. Continued HEF1 evolution can be seen in all clades; all K-lineage viruses from 2019 to 2020 have T161N in the RBD, and all but one have a D353A substitution in the ED; among the S2-sublineage viruses detected in 2016, with substitutions in the RBD, those with A166V and Y301F seem to have disappeared, while those with K190N (a reversion to be C/Taylor/1233/47-like) have expanded and become dominant throughout 2018 to 2020; viruses of the S1-sublineage show greater divergence with various subgroups having cocirculated in 2019 to 2020 defined by specific substitutions in the RBD (e.g., E188D or D194E [a reversion] or T208N) or in the ED (e.g., S64A or I317V or Y326H [the latter two being reversions]). A number of these virus subgroup-defining substitutions, as well as some of those that are virus-specific, map within or in the vicinity of designated neutralization-sensitive epitopes (24). Amino acid substitutions at these additional positions possibly reflect antigenic change in humans which may exert somewhat different antibody-selection pressures on ICV HEF than in mice. The domain structure of HEF and locations of amino acid substitutions defining K- and S-lineages and S1- and S2-sublineages are shown on an HEF monomer structure in Fig. 3, and a full HE gene phylogeny is shown in Fig. S1A, based on all full-length HE gene sequences available in the EpiFlu database of GISAID as of 13 November 2020.

A fuller, non-Hong Kong-focused, analysis of HE using BEAST shows that the oldest viruses with available sequences detected during the 1950s to 1960s conform to the roots of four (T, Y, K, and A) of the six recognized lineages identified on the HE tree topology (Fig. 2B and Fig. S2A). Of these four, the T-lineage represents the root of the tree and shows an estimated time of most recent common ancestor (tMRCA) dated in 1932 (Table 4). Viruses belonging to this lineage died out after 1967. Y-, A-, and K-lineages have tMRCAs dated between 1944 and 1959. A- and Y-lineages cocirculated during the next 40 to 50 years; A-lineage viruses have not been detected since 1991, whereas Y-lineage viruses were last detected in 2004. K-lineage viruses were first detected in 1964 and sporadically thereafter until their reappearance around 1996, since when they have continued to circulate to the present day. M-lineage viruses emerged later (tMRCA 1966), with the oldest representative detected in 1979; viruses of this lineage have not been detected since 2004. S-lineage viruses emerged during the 1980s (tMRCA 1979) and subsequently split into sublineages S1 and S2 (common tMRCAs of 2003 for S1 and 1997 for S2), which together with modern representative viruses of the K-lineage, continue to cocirculate.

**TABLE 4:** BEAST-derived estimates of time of most recent common ancestor (tMRCA) of HE-lineage viruses^*a*^

Lineage	Yr for:		
	tMRCA	95% LHPD	95% UHPD
C/Taylor (T)	1932	1927	1936
C/Yamagata (Y)	1944	1939	1949
C/Kanagawa (K)	1950	1945	1956
C/Aichi (A)	1959	1956	1962
C/Mississippi (M)	1966	1962	1971
C/Sao Paulo (S)	1979	1976	1981
S1 sublineage	2003	2001	2005
S2 sublineage	1997	1996	1998

a For each clade corresponding confidence intervals (LHPD and UHPD) are shown.

As a way of possibly identifying what ICVs might emerge in coming years, levels of minority variants (those with a frequency of <20% in the population of sequences generated for an individual ICV) were considered, although such variants might be detected as a result of either mixed infection or within-host evolution. Minority variants detected in the HE gene sequences of samples collected in Hong Kong during 2019 to 2020, representing those found in less than 20% of sequence reads, are indicated in Table S2. Only 71 nucleotide variants were identified—32 synonymous, 35 nonsynonymous, and 4 indels (all 4 causing frame shifts). Of these, 53, 11, and 7 had frequencies of <5%, 5 to 10%, and >10%, respectively. A total of 23 nucleotide variants were seen in the HEF1 coding region, spread across 13 viruses, and were located at 22 positions that involved 22 codons; 11 (48%) of these variant calls, including the 4 causing frame shifts, were nonsynonymous. The seven minority variants that would be associated with amino acid substitutions were randomly distributed at positions within various domains at the frequencies indicated—E′ (S147F [2.2%], K148R [1.0%]), RB (E188K [5.1%], P192S [11.4%]), and F2 (A387V [1.1%], E389K [2.8%], S431G [2.3%]). A total of 48 nucleotide variants were seen in the HEF2 coding region, spread across 23 viruses, and were located at 32 positions involving 26 codons; 28 (58%) of these variant calls were nonsynonymous. Overall, variant calls were randomly distributed in both HEF1 and HEF2 coding domains, though five and four viruses showed HEF2 I453V and S464A amino acid substitutions, respectively.

### ICV non-glycoprotein-encoding (internal) gene sequencing.

Percentage recoveries from clinical specimens of nucleotide sequences encoding full-length open reading frame (ORF) protein products for NP (58%), CM (61%), and NS (61%) were comparable to that for HE (59%), but those for the polymerase genes (PB2, 46%; PB1, 42%; P3, 49%) were somewhat lower (Table S1), though considerably higher than in our previous study (13), probably reflecting the “freshness” of the samples collected in 2019 to 2020. Generally, internal genes of the recently sequenced ICVs from Hong Kong showed low levels of minority (below 20%) variants (Table S2). For those that were detected in ICV sequences within at least three clinical specimens, with levels ranging from 1.0 to 18.9%, the great majority had not been reported as consensus amino acid residues in the phylogenetic analyses presented in Fig. S1 (Table S3). The functional significances of those that had reached consensus levels (G578E in PB2, S698A and A699G in PB1, T10A in CM1, S23F in CM2) in at least one ICV, together with rare substitutions observed at the positions identified (G424R in P3, S160N in CM1, E75 deletion and E75E/G polymorphism in NS1), are unknown. These observations correlate with the mean rates of nucleotide substitution (number of nucleotide [nt] substitutions/site/year) in the main ORFs, which varied from 4.16 × 10^−4^ to 6.08 × 10^−4 ^nt substitution/site/year (Table 5). The highest rates were observed for the HE-gene and the CM1-ORF, and the lowest rates were for the polymerase genes. However, ratios of nonsynonymous to synonymous evolutionary substitutions (*dN/dS* ratios) were low for CM1 and high for HE but not as high as for NS1, which had a relatively low nucleotide substitution rate.

**TABLE 5:** Mean rates of nucleotide substitution for the main ORFs of ICVs circulating from 1947 to 2020^*a*^

Gene segment	Nucleotide substitution rate^*a*^			*dN/dS* ratio for:	
	10^−4^ substitutions/site/yr	95% LHPD	95% UHPD	SLAC	FEL
HE	5.90	5.33	6.46	0.1520	0.1330
PB2	4.16	3.65	4.65	0.0427	0.0386
PB1	4.71	4.19	5.26	0.0419	0.0385
P3	4.52	4.02	5.06	0.0559	0.0516
NP	5.00	4.36	5.65	0.0734	0.0630
CM1	6.08	5.01	7.15	0.0598	0.0541
NS1	4.99	4.11	5.98	0.3100	0.3020

a Mean rates of nucleotide substitution (10^−4^ substitutions/site/year) and corresponding confidence intervals (lower [L] and upper [U] highest posterior density [HPD]) for the main ORFs of ICVs circulating between 1947 and 2020 were calculated using BEAST, and *dN/dS* ratios were calculated using the SLAC and FEL algorithms; both calculations were performed using the full data sets for each ORF as used for generation of RAxML trees (Fig. S1). As is generally the case, values from SLAC and FEL are not identical but are similar, and there are no significant differences between the values for each protein.

RAxML phylogenetic analyses of the eight ORFs (PB2, PB1, P3, NP, CM1, CM2, NS1, and NS2) within the six genes, relative to the HE-lineage, are shown in Fig. S1B to I. While clustering of HE sequences into six delineated lineages is clear in both RAxML and BEAST trees (Fig. S1A and S2A), the internal gene phylogenies reveal a different pattern. Time-stamped phylogenies for PB2, PB1, P3, NP, CM1, and NS1 (Fig. S2B to G) show that the topology of these six trees is similar, with a small “bottom” branch grouping older viruses (and a few more recent ones in some phylogenies) and a larger “upper” branch grouping viruses with dates ranging from the 1980s to the present. The topology observed is consistent with previous observations by Matsuzaki et al., who described two major lineages of internal genes related to the M- and the Y-defined HE-lineages (12). Our BEAST analysis shows that the age of the node where the split into these bottom and upper branches occurred varies from 1937 (95% highest posterior density [HPD], 1931 to 1941) for PB1 to 1960 (95% HPD, 1951 to 1967) for NS1 (Table 6).

**TABLE 6:** BEAST-derived estimates of time of most recent common ancestor (tMRCA) of the internal genes split (between M- and Y-lineages)

Gene segment	Yr (range) for:		
	Time of split	tMRCA “old” lineage	tMRCA “new” lineage
PB2	1946 (1940–1951)	1952 (1946–1958)	1961 (1956–1964)
PB1	1937 (1931–1941)	1942 (1937–1946)	1970 (1966–1974)
P3	1945 (1941–1949)	1961 (1957–1965)	1976 (1974–1978)
NP	1957 (1953–1961)	1967 (1964–1970)	1977 (1974–1978)
CM1	1945 (1941–1949)	1957 (1952–1961)	1967 (1964–1970)
NS1	1960 (1951–1967)	1971 (1968–1973)	1972 (1967–1976)

Internal gene segments from A-, Y-, and M-lineage viruses cluster either in the bottom branch or at the root of the upper branch. The topology of the maximum clade credibility (MCC) trees representing PB2, PB1, CM1, and NS1 segments consistently show a small cluster of M-lineage viruses (including C/Mississippi/80) as the root of the bottom branch, whereas the upper branch shows C/São Paulo/378/82 as one of the earliest ancestors together with Y-lineage viruses, as described previously (12). Notably, the tree layout is strikingly different for P3 and NP, where the bottom branch is related to S- and Y-lineage viruses, while the upper branch stems from C/Mississippi/80 and related viruses. These incongruences in the tree topologies are strongly indicative of reassortment events occurring at the time of or soon after the split into the two major internal gene lineages.

K-lineage and S1-/S2-sublineage viruses constitute the majority of viruses in the upper branch of each tree. The oldest K-lineage viruses (C/Kanagawa/1/76, C/Miyagi/77, C/Aomori/74, and C/Yamagata/64) cluster in the bottom branch, except in the CM1 tree, whereas the reemergent 1990–2000 viruses cluster in the upper branch. A cluster of S2-sublineage viruses dated between 2005 and 2016 was observed in the bottom branch in the PB2 and NP trees, with the oldest representative being C/Aichi/1/99. This is another clear case of internal gene reassortment, which features heavily in ICV phylogenies.

Amino acid substitutions annotated on the internal gene phylogenies indicate that the M- and Y-lineage split was complemented by a combination of nonsynonymous mutations occurring across the genomes of viruses in the upper branch. Viruses from the 1970 to 1980s were the earliest to show substitutions in either PB2 (S387N, S436T), PB1 (D76N, S88G), P3 (T55A, S58N, I708T, E193K), NP (E74D), or NS1 (F91L, Q212R) but not together. These changes only appear concomitantly in more recent viruses from 1990 onward, such as C/Yamagata/5/92 and C/Miyagi/3/93. Additional amino acid substitutions include V359I, N178D, and V330I in P3 and K59E in PB1, which are fixed in currently circulating viruses, or V289I in PB2, which is present in the majority of S1-/S2-sublineage viruses from 2019 to 2020. The CM1 phylogeny is the only tree where no particular signature changes were related to the proposed split.

Both CM1 and NS1 trees show a split of the upper branch into two further branches; one contains two clusters corresponding to viruses from the Y-lineage and S2-sublineage, the latter ones displaying T189A in CM1. The second branch shows 3 clades, one with K-lineage viruses from the early 2000s, another K-lineage group of more recent viruses with L241I in NS1, and a S1-sublineage group with a proportion of them showing G88E and T39A substitutions in NS1.

The bottom branches, containing old genome arrangements, continued to survive in a minority of viruses, particularly in a group of S2-sublineage viruses collected between 1999 and 2016. These viruses display a mixture of “old” PB2 and NP and “new” PB1, P3, CM1, and NS1 segments, with substitutions A298T in NP and K119R in PB1, which were not seen in other clusters.

Overall, these analyses provide further evidence of several ICV evolutionary events having occurred in the 1940s to 1960s across most internal gene ORFs which caused a major split and the emergence of a new internal gene lineage through reassortment and accumulation of amino acid changes. These events may have forged a new genome constellation combining the internal genes of HE Y-lineage viruses with P3 and NP genes from HE M-lineage viruses, which may have produced viruses of improved fitness compared to those constituting the bottom branch.

### Patterns of gene reassortment in ICVs.

The observations arising from HE gene phylogeny analysis compared with those emerging from internal gene ORF phylogenies reveal that viruses from the same HE-lineage often display different internal gene constellations in different seasons, while viruses from different HE-lineages circulating in the same or contemporary seasons share a similar combination of internal genes. Whereas the HE phylogeny shows at least three lineages cocirculating in most seasons, the internal gene ORF trees show a major split resulting in two lineages composed of different gene constellations which continued to evolve. The large number of reassortment events occurring in each or most seasons suggests these lineages are continuously exchanging gene segments to potentially maintain virus fitness in the face of changing selective pressures.

**FIG 4:**
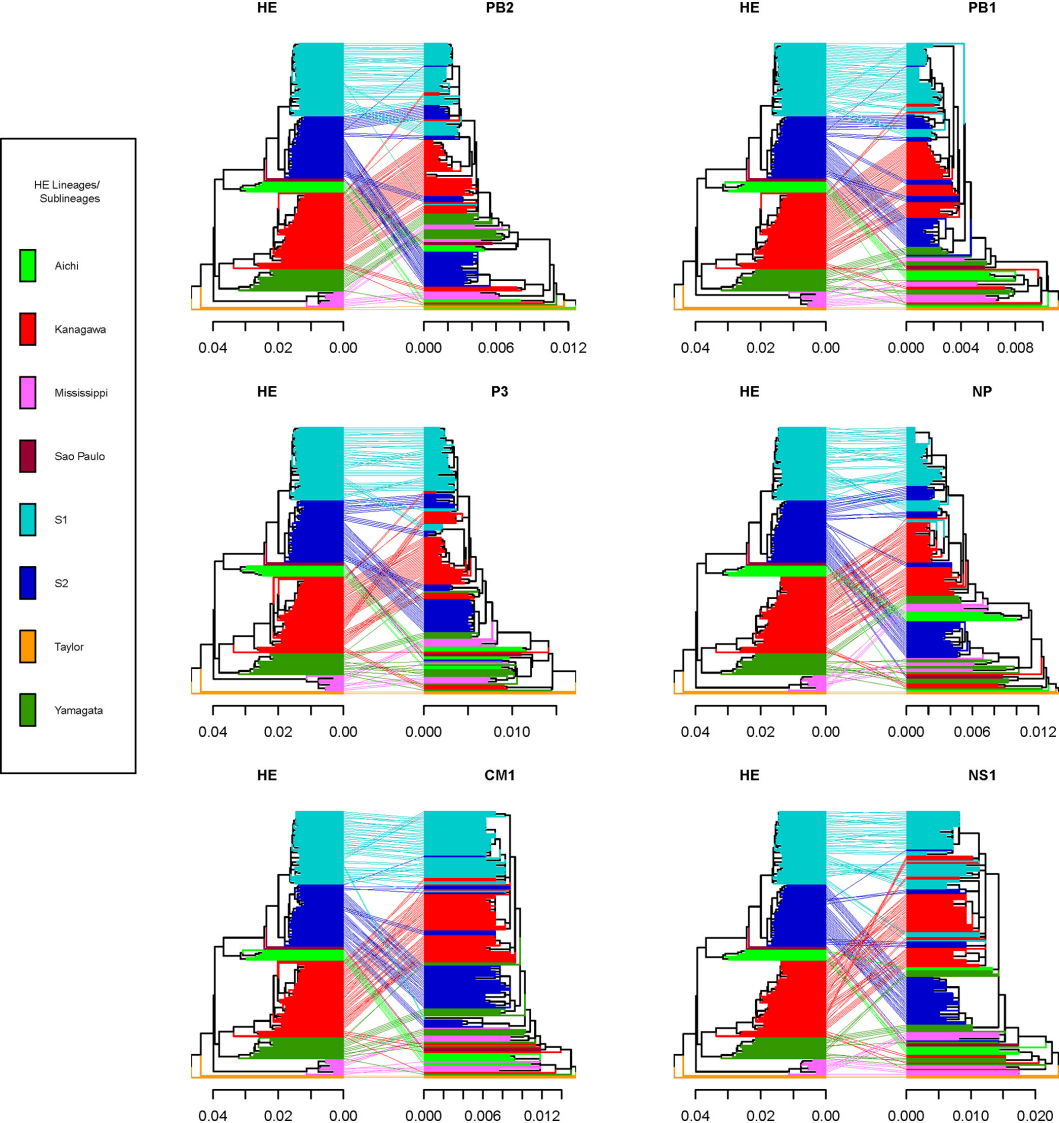
Reassortment events for ICVs with complete genome sequences. Tanglegrams were built based on maximum likelihood (ML) phylogenies and are displayed with the ML HE tree on the left and the mirrored trees of PB2, PB1, P3, NP, CM1, and NS1 on the right. Branches and twines between paired trees are colored according to HE lineage/sublineage.

To investigate reassortment over the entire genome, a combined approach of tanglegram and GiRaF analyses was applied. A subset of viruses (*n* = 168, with 64 being from specimens collected in Hong Kong) with complete full-genome sequences were used for this analysis. Tanglegrams were constructed by mirroring the HE gene phylogeny against those built for the internal gene ORFs. Reassortment events are characterized by segment sequences from the same virus placed in different positions on the corresponding phylogenies; in the absence of reassortment, segments should be linked by horizontal, noncrossed lines. Given the striking differences in the topology of the HE phylogeny versus those of the other ORFs, the resulting tanglegrams showed an elevated proportion of crossed lines, suggesting a high degree of reassortment events, as suggested (Fig. 4).

**FIG 5:**
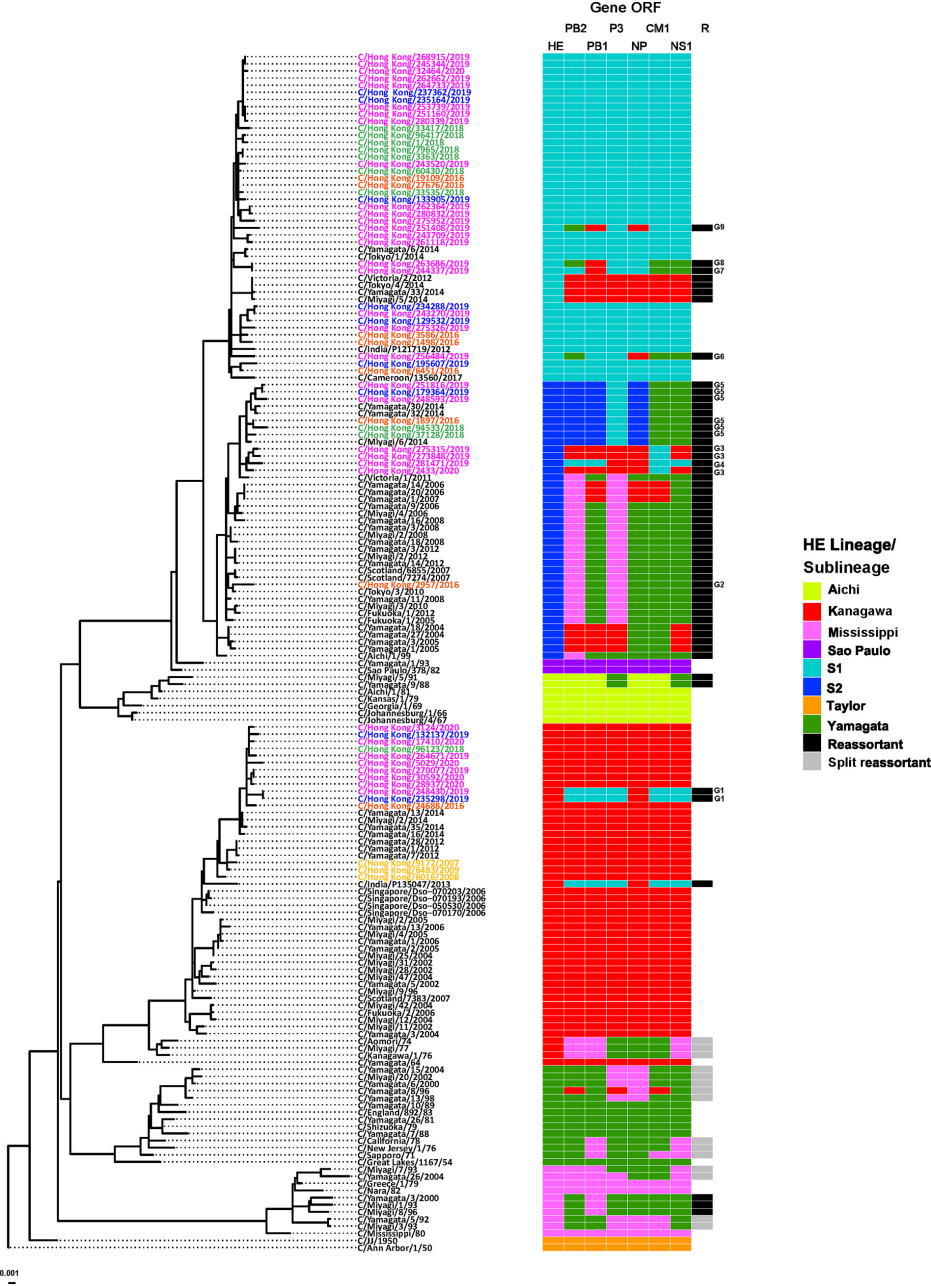
Reassortment heatmap for ICVs with complete genome sequences. A maximum likelihood (ML) HE tree of a set of 168 ICVs with HE lineage color-coded genome constellations is shown. Based on an approach using tanglegrams combined with GiRaF analysis for characterization of reassortment events, the HE lineage classification was extended to all the internal protein ORFs and is indicated by color-coded bars. Column R highlights reassortant viruses (black bars), while those characteristic of the “split reassortment” which occurred between C/Mississippi- and C/Yamagata-lineage viruses in the 1970s are marked with gray bars. Reassortant genotypes (G1 to G9) detected in Hong Kong over the period 2016 to 2020 are indicated after the corresponding black bars, and the names of ICVs from Hong Kong are colored by time period: pre-2016, yellow; 2015–2016 outbreak, orange; 2017–2018 outbreak, green; 2019 interoutbreak, blue; 2019–2020 outbreak, pink.

To expand this analysis, we applied GiRaF to all ORFs, to catalogue reassortment events more comprehensively by dividing segments into donors and acceptors. Based on these results, we assigned an HE-lineage identity to each of the internal gene ORFs to allow construction of a final picture of gene constellations across the genomes of ICVs identified as reassortants (Fig. 5). We observed two types of reassortment events, multiple independent reassortments observed in viruses with collection dates after 1990 and the internal gene “split” reassortments which are evident in viruses from M- and Y-lineages up to the early 2000s, reflecting the segment mixing pattern that followed the major split observed among internal gene phylogenies, with emergent ICVs having a new gene constellation comprising PB2, PB1, CM1, and NS1 derived from Y-lineage viruses and P3 plus NP from M-lineage ICVs. Of the prototype viruses selected to represent the six HE-lineages (26), those for M-, Y-, A-, and S-lineages appear to be nonreassortant (i.e., all six internal genes matching the HE-lineage), while the internal genes of the K-lineage prototype (C/Kanagawa/1/76) were all derived from either M- or Y-lineage viruses; the C/Taylor/1233/47 prototype was not included, as the individual gene sequences available in GISAID were not all from the same virus stock. The prototype selected to represent the S1-sublineage, C/Tokyo/1/2014, appears to be nonreassortant, while that for the S2-sublineage, C/Fukuoka/1/2005, again has internal genes derived from either M- or Y-lineage viruses (13).

Of the 168 ICV genomes, 70 showed gene constellations indicative of reassortment spread across 23 different genotypes representative of double (*n* = 10), triple (*n* = 12), and quadruple (*n* = 1) reassortants, i.e., derived from 2, 3, or 4 parental prototypes (Table 7, Fig. 5). For the ICV lineage and sublineages still in circulation, reassortant genotypes detected in Hong Kong are designated G1 to G9. Of 48 K-lineage viruses collected from 1964 to 2020 just 6, represented by two genotypes, were reassortant, with 2 from genotype G1 being detected in Hong Kong in 2019; similarly, of 46 S1-sublineage viruses collected from 2012 to 2020 just 8, represented by 5 genotypes, were reassortant, with the 4 from Hong Kong being detected in 2019 and each representing a novel genotype (G6 to G9), but all 39 S2-sublineage viruses collected from 1999 to 2020 were reassortant. Overall, seven genotype categories were observed among HEF S2-sublineage viruses: (a) G2, M-lineage-related PB2 and P3 with Y-lineage-related PB1, NP, CM1, and NS1 in viruses detected between 2005 and 2016 (e.g., C/Fukuoka/1/2005, *n* = 18); (b) K-lineage-related PB2, PB1, P3, and NS1 with Y-lineage-related NP and CM1 in viruses from Yamagata in 2004 to 2005 (e.g., C/Yamagata/1/2005, *n* = 4); (c) G5, S1-sublineage-derived P3, and Y-lineage-derived CM1 and NS1 in viruses from 2014 to the present (e.g., C/Miyagi/6/2014, *n* = 9); (d) G3, K-lineage-derived internal genes with an S1-sublineage-derived CM1 in viruses from the last season (e.g., C/Hong Kong/2433/2020, *n* = 3); (e) G4, S1-sublineage-derived PB2, PB1, CM1, and NS1 with K-lineage-derived P3 and NP (e.g., C/Hong Kong/281471/2019, *n* = 1); (f) M-lineage derived PB2 with other internal genes being Y-lineage-derived (e.g., C/Aichi/1/99, *n* = 1); (g) M-lineage PB2 and P3, K-lineage PB1, NP, and CM1, and Y-lineage NS1 (e.g., C/Yamagata/20/2006, *n* = 3). Categories a to f are all triple reassortants, while category g is quadruple reassortant.

**TABLE 7:** Summary of data presented in Fig. 4 for all 168 ICV full genomes analyzed^*a*^

HE lineage	No. of viruses	No. of reassortants (RA)	No. of RA genotypes
Taylor	2	0	0
Mississippi	10	7	4
Yamagata	14	8	4
Aichi	7	2	1
Kanagawa	48	6	2
Sao Paulo	2	0	0
S1-sublineage	46	8	5
S2-sublineage	39	39	7
Totals	168	70	23

a The number of ICVs by lineage/sublineage followed by the number of ICVs having a reassortant (RA) genome and the number of genotypes associated with the RAs is indicated.

Focusing on ICVs detected in Hong Kong, similar ratios of viruses in the lineage and sublineages were observed in the 2015–2016 and 2017–2018 outbreaks and for viruses collected in 2019 prior to the start of the 2019–2020 outbreak (Table 8). Two S2-sublineage viruses collected in 2015 to 2016 were determined to be distinct triple reassortants (G2 and G5), and two viruses of the G5 genotype were detected in 2017 to 2018. Detection of the G5 genotype continued into the pre-2019–2020 outbreak period, and the G1 genotype, a double reassortant with K-lineage HE and NP, and other genes from a S1-sublineage virus, emerged. During the 2019–2020 outbreak, multiple reassortant genotypes were identified—four triple reassortants (G6, G7, G8, and G9; *n* = 1 each) among S1-sublineage viruses, G1 (*n* = 1) among K-lineage viruses, and three triple reassortants (G3, *n* = 3; G4, *n* = 1; and G5, *n* = 2) among S2-sublineage viruses.

**TABLE 8:** Summary of data presented in Fig. 4 for ICVs from Hong Kong detected 2015–2020^*a*^

Lineage	2015–16 outbreak		2017–18 outbreak		2019 outbreak		2019–2020 outbreak	
	No. of ICVs	RA genotypes	No. of ICVs	RA genotypes	No. of ICVs	RA genotypes	No. of ICVs	RA genotypes
Kanagawa	1		1		2	G1 (1)	8	G1 (1)
S1-sublineage	5		7		6		20	G6 (1), G7 (1), G8 (1), G9 (1)
S2-sublineage	2	G2 (1), G5 (1)	2	G5 (2)	1	G5 (1)	6	G3 (3), G4 (1), G5 (2)
Total	8	2 genotypes (2)	10	1 genotype (2)	9	2 genotypes (2)	34	8 genotypes (11)

a Data specific for ICVs from Hong Kong collected in the period 2015 to 2020, spanning the three outbreaks and the 2019 period prior to the 2019–2020 outbreak; three viruses collected in 2007 to 2009 were all non-RA K-lineage viruses. Specific genotypes (G1 to G9) are shown for RA genomes associated with each lineage/sublineage, with the number of viruses having the specified genotype indicated in parentheses.

## DISCUSSION

### Seasonality.

Seasonality of acute infectious diseases has been long recognized (27), commonly occurring on an annual basis but for some disease agents occurring less frequently, such that seasonality might be defined as recurrence at regular intervals. Numerous, multifaceted factors, which vary for different disease agents, act as drivers of seasonality and have been the subject of many reviews (28–31). These factors include environmental changes (notably, climate, e.g., temperature, humidity, wind conditions, and sunlight), persistence of pathogens in the off-season in the absence of epidemic spread, host behavior changes (crowding at certain times of year, notably in relation to festivities and school closure/reopening), and seasonal physiologic changes in the host (possibly related to changes in photoperiod with consequent effects on vitamin D and melatonin levels that may result in weakening of immune system functions) resulting in increased susceptibility to some disease agents. The winter seasonality of many respiratory virus pathogens may well be driven by enhanced wintertime survival of pathogens (outside a host) and increased host susceptibility due to a level of immune suppression during winter.

**FIG 6:**
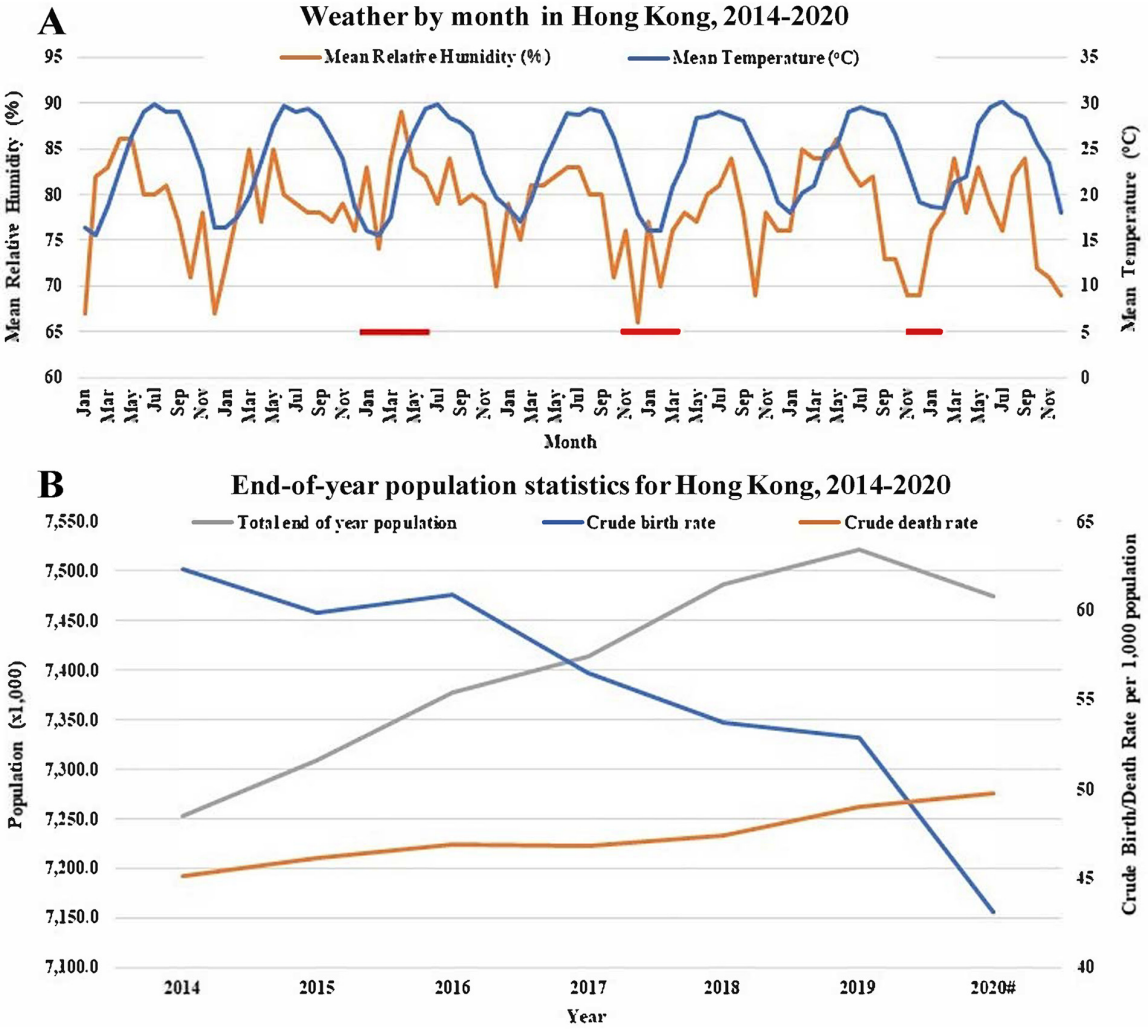
Hong Kong statistics relating to weather and human population. (A) Means of relative humidity (%) and temperature (^o^C) by month for the years 2014 to 2020 are shown. Data were downloaded from https://www.hko.gov.hk/en/wxinfo/pastwx/mws/mws.htm for the individual years. The times of ICV outbreaks in 2015 to 2016, 2017 to 2018, and 2019 to 2020 are indicated by red bars. The end time for the 2019–2020 outbreak is unknown due to cessation of ICV surveillance after week 07/2020. (B) Crude birth and death rates calculated at the end of each year for the years 2014 to 2020 together with total end-of year population estimations are shown. Data were downloaded from the https://www.censtatd.gov.hk/en/website with data for 2020 (#) being provisional as of 14 March 2021.

In terms of environmental conditions, the three outbreaks we have studied showed seasonality, and all occurred at the coldest time of year, when temperatures were between 15 and 20°C and mean humidity was in the lower range of 65 to 80% (Fig. 6A). However, similar conditions were seen for the same periods in nonoutbreak years, so differing weather conditions seem unlinked to seasons of high and low ICV activity. Population dynamics might play a role, and changes in the ICV-susceptible population, especially children, need to be considered. Statistics have shown a fall in the birth rate between 2014 and 2020 (and hence a reduction in numbers of naive hosts) and a smaller increase in the death rate, but an increase in population and therefore hosts (with the longevity of both sexes having increased over the period) susceptible to reinfection up to 2019 (Fig. 6B). The ∼50,000 drop in population during 2020 may be related largely to emigration (https://www.censtatd.gov.hk/en/press_release_detail.html?id=4825), given the low impact of SARS-CoV-2 in Hong Kong (Fig. 1A, inset).

Overall, weather conditions, susceptible host numbers, and antigenic evolution of ICV, over the 7-year period studied, do not appear to play significant roles in determining the 2-year seasonality of ICV infection in Hong Kong. It is possible, therefore, that levels of herd immunity play a role in the emergence of biennial ICV epidemics, which have also been seen in Japan (18), with similar trends having been indicated in other countries (19). Temporary curtailment of ICV circulation after each peak of transmission could occur due to high levels of herd immunity and an inability of the HEF glycoprotein to accommodate frequent antigenic drift substitutions owing to functional constraints. In support of this, it has been suggested that replacement of a dominant antigenic group may be caused by selection within immune or partially immune older children and/or adults in the community being responsible for selecting the HE lineage that infects young children who lack anti-ICV antibodies (18). Equally, waning (short-lived) immunity in previously infected individuals may be a significant contributing factor to the 2-year cycling of ICV outbreaks. Such a scenario has been shown in human volunteers infected with coronavirus 229E, where waning immunity over a year was shown to result in reinfection, though periods of virus shedding were shorter and symptoms were milder than during the initial infection event (32). Further, 2-year cycling of outbreaks caused by human coronavirus NL63 has also been documented (33).

### Genome evolution and reassortment.

Table 3 shows that similar ratios of S1- (60 to 70%) and S2-sublineages (16 to 26%) and K-lineage (6 to 20%) viruses were observed in all three outbreaks, and comparable ratios were observed in the 2019 intervening period, suggestive of low-level circulation of ICV between the 2017–2018 and 2019–2020 outbreaks. However, the highest level of K-lineage viruses (20%) was seen in the 2019–2020 outbreak and was largely associated with viruses having an HEF1 T161N amino acid substitution which may affect antigenicity and/or receptor-binding.

Selection pressures exerted on the surface glycoproteins of viruses (HEF of ICV), largely by the host immune system, result in the genes encoding them often displaying the greatest divergence over time (virus evolution) compared to the other genes that encode the virus. Figure 3 shows HEF amino acid substitutions that have occurred, compared to the sequence of C/Taylor/1233/47, in viruses of the currently circulating K-lineage and S1-/S2-sublineages. We have previously discussed at some length the possibility that many of the amino acid substitutions in and around the receptor-binding domain are selected under host immune pressure, and we drew parallels with observations made for influenza A (IAV) and influenza B (IBV) viruses (13). However, there are also significant numbers of substitutions within the HEF esterase domain and, like those within the receptor-binding domain, there are different substitutions within the K-lineage and S1-/S2-sublineages. While several of these may also have been selected under host immune pressure, it is possible that some may represent compensatory substitutions to maintain receptor-binding/esterase balance and virus fitness, as has been postulated for HA and NA of IAV and IBV (34–37).

Consistently, rates of nucleotide substitution estimated for the HE-gene have been higher than those for the other ICV gene segments, but for that encoding CM1. The overall substitution rates determined here (Table 5) were slightly higher than those determined in another report (12), perhaps reflecting our longer study period (1947 to 2020 versus 1947 to 2014), although confidence intervals show no significant differences. The rates of nucleotide substitution calculated are similar to those seen for IBV, which has been interpreted to indicate that humans are the natural host for IBV and ICV (17), something that has been supported by a study of ICV HE-gene codon usage (22). All ICV proteins show numbers of amino acid substitutions along the trunks of the phylogenies associated with evolution from the Taylor lineage to the currently circulating K-lineage and S1-/S2-sublineages (Fig. S1), but the functional significance of most of these substitutions, together with those in the proteins of individual viruses, is unknown.

HEF is the main target of hosts’ antibody responses, but there are considerable structure/function restrictions on variation that can be tolerated to ensure maintenance of the receptor-binding, acetylesterase, and membrane fusion activities of the glycoprotein. In relation to the *dN/dS* ratios (Table 5), only HEF had sites undergoing positive selection (*P* < 0.05) at amino acid positions 194, 323, and 624. Position 194 is a component of antigenic site Y-1 residing in the receptor-binding domain (13, 24), and selection at position 194 was identified previously (22). Position 323 has shown considerable variation (E323/K/Q/N/R) over time (Fig. S1A), possibly indicating that it may be a component of an antigenic site, and is within the esterase domain but somewhat distant from residues (S57, D352, and H355) that define the catalytic site (Fig. 3B). However, such amino acid substitutions may affect the esterase kinetics. For IAV H3N2 neuraminidase, it has been shown that introduction of positively charged amino acid substitutions in a region that has been identified as an antigenic site, which is remote from the catalytic site, results in reduced sialidase activity (38). Amino acid substitutions at both positions 194 and 323 have played roles in ICV lineage-evolution (Fig. 3) and may be significant players in maintaining the receptor-binding/receptor-destroying balance of HEF. Position 624 equates to position 192 of HEF2 and falls in the middle of the transmembrane anchor domain. This domain had been considered a passive anchor for HAs of IAV and IBV, but recent mutagenesis and structural studies have revealed that the transmembrane domain (TMD) of IAV plays important roles in determining the conformation and fusogenicity of HA (39–42). There is significant variation in amino acid sequences of TMDs between the IAV subtypes and concomitant variation in their physicochemical properties (42), which possibly translates into differences in functionality between the TMDs of different subtypes (43–45). A comparison of the TMD of ICV with the TMDs of IAV H1, H2, and H3 HAs is shown in Table 9; while their TMDs are of similar length, the cytoplasmic tail of ICV HEF is considerably shorter than those of IAV HA subtypes. Of all the IAV HA subtypes, only H2 shows conservation of an SLA amino acid motif, equivalent to positions 626 to 628 in ICV HEF, in the middle of the TMD. In a series of mutagenesis experiments, Lin et al. showed that either Ser to Ala substitution or deletion of the Ser at a location equivalent to position 624 in ICV HEF resulted in a shift in budding of HA from the apical to the basolateral membranes of Madin-Darby canine kidney (MDCK) cells (46). Of the 432 HE genes analyzed in Fig. S1A, the great majority (415) have an AA motif at positions 623 to 624 of HEF, with just five viruses in the T-, M-, and Y-lineages having the TP motif, nine viruses in the M-lineage having an A624V substitution, and three viruses having an A624T substitution. C/Johannesburg/1/66, for which the structure of HEF has been determined (25, 47), is one of the viruses with an A624T substitution. If ICV does tend to bud basolaterally, it could contribute to the inability of recovering ICVs that propagate to high titer in adherent mammalian cell cultures. Further experimentation would be required to address the functionality of the three HEF positions under positive selection.

**TABLE 9:** The transmembrane domain of ICV HEF^*a*^

Virus	Accession no.	Transmembrane domain/cytoplasmic tail sequences	Experimental result
HEF position 624		**$**	
ICV HEF TM cons	N/A	WGSSLGLAIT AA I SLA ALVISGIAIC | RTK	N/A
ICV HEF TM early	N/A	..........TP. ............. | ...	N/A
A(H2N2) HUMAN cons	N/A	∼ILAIYATVAGSL...IMMAGISFWM | CSNGSLQCRICI	Predominantly apical budding
Mutant-2A520	N/A	∼......... *AA*.............. |............	Predominantly basolateral budding
Mutant-4A520	N/A	∼......... *AAAA*............ |............	Predominantly basolateral budding
Mutant-S521A	N/A	∼.......... *A*.............. |............	Predominantly basolateral budding
Mutant-ΔG520ΔS521	N/A	∼.........--.............. |............	Predominantly basolateral budding
Mutant-G520S	N/A	∼......... *S*............... |............	Random transport to budding sites
A/South Carolina/1/18 (H1N1)	EPI5571	∼.....S...S..V.LVSLGA..... |............	N/A
A/Lyon/CHU-0211681950/2021 (H1N1) pdm09	EPI1920739	∼.....S….S..V.VVSLGA..... |............	N/A
A/Hong Kong/1/68 (H3N2)	EPI8441	W..W.SFAISCF.∼.CVVLL.FIM.A.Q | R.NIR.N…	N/A

a Consensus (cons) sequences are shown for ICV HEF and human H2 HA with alignment around a conserved SLA motif (underlined). The location of residue 624 in ICV HEF is indicated ($). Sequences of prototype human H1, H1pdm09, and H3 HAs, with accession numbers related to sequences available in the EpiFlu database of GISAID (https://www.gisaid.org/), are shown aligned against H2 HA cons. Sequence identities (.) to ICV cons are shown for ICV HEF early (a few viruses isolated between 1950 and 1983) and for H1, H1pdm09, and H3 HAs against H2 HA cons, with spacing (∼) introduced to improve alignments. Transmembrane domain and cytoplasmic tail sequences are separated by a vertical bar (|). For H2 mutant HAs the effects of mutagenesis involving the equivalent of ICV HEF residues 623 and 624 are shown in terms of HA budding in MDCK cells (46). All mutant sequences are aligned to the H2 HA cons sequence with amino acid substitutions indicated in italics. Δ/− indicate amino acid deletions. N/A, not applicable.

The observation of positive selection in HEF only is somewhat surprising, as NS1 and NS2 show high levels of amino acid substitution (Fig. S1H and I), and NS1 has the highest *dN/dS* ratio (Table 5), but this probably relates to NS1 being a multifunctional protein that needs to be flexible to allow interaction with several intracellular components of the host (48–51). In contrast, despite the high nucleotide substitution rate of CM1, the majority of amino acid substitutions are synonymous, such that CM1 shows the lowest number of amino acid substitutions over time, while variation in CM2 is considerably higher (Fig. S1F and G), as was reported by Tada et al. based on a data set derived from just 24 ICVs from the period 1964 to 1991 (52). This suggests that the two CM-gene products are evolving differently in response to selective pressures or structural and functional constraints.

CM1 of ICV is the key component in inducing the formation of protrusions, called cord-like structures (CLS), radiating out of infected cells (53) and in regulating their size (54, 55), thus demonstrating a role for CM1 in membrane remodeling. An amino acid substitution at position 24 of CM1 (T24A) has been shown to cause a change in virus morphology from filamentous to spherical (55). Moreover, structural studies have shown that CM1 assembly on membranes is the driving force for virus budding and egress from infected cells (56). Conversely, CM2 appears to play roles in genome packaging and virus uncoating during the ICV replication cycle (57).

In addition to CM1, the HEF glycoprotein may play a role in ICV assembly, as it forms hexagonal lattices on the virion surface which persist when released from the virus by proteolysis (58–60). However, these studies were performed with ICVs (C/Taylor/1233/47 or C/Johannesburg/1/66) that had undergone multiple passages in cell culture or eggs, and it has been reported that hexagonal surface lattices were not observed on a newly isolated ICV but could be seen on many virions after a few additional passages of the virus in hens’ eggs (61). Recently, using the ICV C/Johannesburg/1/66 cultured in MDCK cells, *in situ* structures of HEF have been determined, and while lattice formation occurred independently of other virus components, a major role for CM1 in particle formation was shown, with a well-defined CM1 layer being associated with virus particles having a more defined size and curvature (47). The high degree of ICV CM1 amino acid conservation probably reflects the important role it plays in the ICV replication cycle.

All influenza viruses have segmented genomes, which makes them susceptible to gene reassortment when cells are infected with two or more viruses. Reassortment has been studied in greatest depth for IAVs that are known to be made up of 18 HA (H1 to H18) subtypes and 11 NA (N1 to N11) subtypes, existing with various H and N combinations and various internal gene cassettes. These various HN combinations are maintained in birds and bats, with spillover into other animals, notably, pigs, horses, and dogs (62). A(H9N2) viruses in birds and swine have been identified as key donor viruses in generating reassortant viruses with zoonotic potential that pose a threat to public health (63, 64). While the HA and NA are key factors in determining the zoonotic potential of IAVs, a functional viral ribonucleoprotein (vRNP) complex, composed of polymerase genes and nucleoprotein, is required for a reassortant virus to be viable, a requirement that is a restricting factor for gene reassortment (65). In this context, cooperation between PB2 and PB1 has been shown to be important, and restriction can be overcome by complementary mutations in PB1 (66), while H9N2 PB2 and PA genes, with mammalian adaptive mutations, were shown to confer enhanced polymerase activity in human cells and greater virus replication in mice (67). Reassortment is a major feature of ICV evolution, and it has been proposed that ICV cannot evolve through generation of antigenic variants because of the stringent functional constraints on the HEF glycoprotein, such that ICV relies on its genome composition to influence spread in humans (26), possibly by selection for virus genomes that restrict virus fitness with periodic acquisition of an advantageous internal gene composition (12). Functional studies of the ICV vRNP are limited, but structural studies have shown that the ICV vRNP is remarkably different from that of IAV and IBV, possibly indicating significant functional differences (68), with a subsequent study identifying amino acids in PB2 and P3 (all of which were conserved across our alignments) responsible for interacting with the human ANP32A protein (69). Our phylogenetic and BEAST analyses of the NP segment show that most amino acid substitutions occur within the C-terminal region (Fig. S1E and S2E), often at the same position across the trees, such as 522 or 540. The NP of ICV has an extended C-terminal domain, compared with those of IAV and IBV, which contains nuclear localization signals (NLS) spanning from R513 to K549, notably a KKMK (546 to 549) motif. Mutations within this region can modulate the kinetics of nuclear import of NP and assembly of vRNP complexes to different extents, thereby affecting polymerase activity and, ultimately, viral replication and fitness (70).

A total of 61 complete genomes from ICVs detected in Hong Kong in the period 2016 to 2020 were analyzed for reassortment, 18 from 2016 to 2018 and 43 from 2019 to 2020, with 9 reassortant genotypes being identified (G1 to G9; Fig. 5). Four of the viruses from 2016 to 2018 (22%) were reassortant; all had S2-sublineage HE and were split between G2 (*n* = 1) and G5 (*n* = 3). Greater diversity was seen among the 13 (30%) reassortant ICVs from 2019 to 2020 involving 4 S1-sublineage viruses (1 each of G6, G7, G8, and G9), 2 K-lineage viruses (both G1), and 7 S2-sublineage viruses (3 G3, 1 G4. and 3 G5). G5 ICVs were detected in both time periods in Hong Kong, possibly indicative of better fitness compared to some other reassortant genotypes. With the caveat that only 168 full genomes were available for reassortment analysis, it is notable that three of the genotypes occurred in older viruses, G1 in C/India/P135047/2013, G2 in ICVs detected in Japan and Scotland with collection dates going back to 2005, and G5 in ICVs detected in Japan in 2014. Overall, our Bayesian analyses support previous work that showed ICV gene reassortment to play a significant role in the generation of viruses with specific gene constellations, thereby increasing genetic diversity, but with many genotypes possibly having relatively short life spans due to further reassortment (12, 17).

### Virus persistence.

Another factor that could be involved in recurrence at regular intervals is virus persistence, whereby a virus is not cleared but remains in specific cells of an infected individual, with the possibility of recrudescence (71). Such persistence can be established by modulation of virus and/or cellular gene expression, together with modification of the host immune response, and often involves periods of silent and productive infection, neither of which causes excessive damage to host cells.

Given the high seroprevalence rate in adults, it has been suggested that most ICV infections in adults are due to reinfection (72, 73). However, rather than reinfection, ICV persistence may play a role. In this context, a recent longitudinal study of anti-ICV antibodies in a group of healthy adults in Sendai, Japan, covering the period 2011 to 2016 showed viruses of the K- and S-lineages to have been circulating (as we observed in Hong Kong), with 10/57 (17.5%) volunteers showing signs of ICV infection over this period (74). One individual, subsequently studied over the period 2007 to 2019, showed persistently elevated antibody titers from 2010 to 2019 inclusive, 16- to 32-fold and 4- to 8-fold, respectively, against representative viruses of K- and S-lineages compared to titers measured between 2007 and 2009, which could be related to persistent ICV infection rather than many reinfection events. Indeed, reinfection of an individual with established ICV persistence could have played a role in generating the high level of ICV genome reassortment reported in Japan at a time when A-, Y-, and M-lineage viruses were cocirculating (26), as identified more recently (12, 17) and as detected here (Fig. 5).

Experiments conducted with volunteers showed that ICV can infect humans and generally cause common cold-like symptoms (2), though severe acute respiratory infection can sometimes occur (9), while studies of ICV outbreaks in a children’s home showed that virus shedding can persist for over 3 weeks (75). Further, seroepidemiologic studies have shown high seroprevalence among older children through to elderly adults, indicative of intense circulation of ICV among humans (76, 77), but with only those cases showing more severe symptoms seeking medical assistance and few of these being tested for the virus (19). This fact, linked with low ICV isolation rates, has resulted in there being few full-genome sequences available for analysis (21), highlighting the significance of the 43 full-genome sequences generated in the course of the present study. While reports of ICV isolations during an outbreak of IAV and IBV, and during an A(H3N2) epidemic, have been published (78, 79), we showed previously that ICV detections tend to occur early in influenza seasons in Hong Kong but are then dwarfed by epidemics of IAV and IBV (13), as we observed here for the 2019–2020 outbreak (Fig. 1). Like the previous two outbreaks in Hong Kong, HE gene sequences clustered in the K- and S-lineages, with the majority (60%) falling in the S1-sublineage (Table 3).

In support of the observations of infected individuals, it has been shown that ICV (A/Ann/Arbor/1/50) can establish persistence in culture that involves downregulation of the ICV receptor and alteration of the HEF affinity for the receptor, with the persistent virus being dominant in coinfection experiments (80–82). More recent studies have shown that ICV receptors, 9-*O*-acetylated sialic acids, are widely but variably expressed across cell lines and tissues from a variety of potential host species and, in all but the mouse, most are concentrated in the cells’ Golgi complexes, with only low-level expression at cell surfaces (83–85). Such concentration of ICV receptors in a cell’s pathway for processing and packaging of proteins and lipid molecules, notably, proteins destined for export from the cell, such as HEF, could play a role in establishing an ICV persistent state.

Extensive studies have shown that IAV and IBV generally cause acute infection with limited virus diversity (86), though higher diversity has been observed when primary infection is caused by multiple related strains (87, 88). However, chronic infection can be established in immunocompromised individuals with virus shedding occurring over extended periods (89), during which time extensive virus evolution and emergence of antigenic variants can be observed (90, 91).

### Viral interference/enhancement.

Three of the ICV infection cases studied here showed coinfection with an IAV, but we do not know if ICV infection occurred first and thus do not know if this represents interference or enhancement or was simply coincidence (Table S1). Coinfection of the respiratory tract with at least two viruses is a common finding in hospitalized patients, and modeling has shown that one virus can block another, so-called viral interference, either by being the first to infect available host cells or by having a faster replication rate than the other virus; e.g., rhinovirus can out-compete a number of slower-replicating viruses (92). Alternatively, infection with one virus might promote coinfection with another; e.g., infection of mice with IAV leads to enhanced expression of the cellular receptor angiotensin-converting enzyme 2 (ACE2) and increased susceptibility to SARS-CoV-2 infection (93).

A modeling study based on 44,230 cases of respiratory illness that were tested for 11 different viruses has provided evidence for the role of innate immunity in driving asynchronous circulation of IAV and rhinovirus (94). Indeed, through stimulation of antiviral defenses in the airway mucosa, rhinoviruses have been proposed to have disrupted the 2009 IAV pandemic in Europe (95). Further, surveillance data in Hong Kong indicated viral interference during epidemics of common respiratory viruses that affect the timing and duration of subsequent epidemics caused by other viruses; notably, a rhinovirus epidemic tended to shift subsequent epidemics of other viruses (96). In the context of the current COVID-19 pandemic, it has been shown that rhinovirus triggers an innate immune response that prevents SARS-CoV-2 replication in human respiratory epithelium (97).

Given our observation that all three ICV outbreaks in Hong Kong since 2014 have been swamped by subsequent IAV and IBV epidemics (Fig. 1B and C; reference 13), it is possible that IAV/IVB might impose viral interference on ICV. However, there may be another factor at play in the suppression of ICV; the receptor-destroying activity of ICV acetylesterase, removal of the *O*-acetyl group from 9-*O*-acetyl-*N*-acetylneuraminic acid, abolished ICV hemagglutination of erythrocytes and enhanced hemagglutination by IAV and IBV (98), while ICV esterase treatment of substrates containing 9-*O*-acetyl-*N*-acetylneuraminic acid (e.g., possible inhibitors of ICV in mucin and saliva) resulted in increased IAV sialidase activity on the deacetylated substrates (99, 100). These observations suggest that ICV acetylesterase might contribute to unmasking of new receptors for IAV and IBV on cell surfaces, thereby facilitating superinfection by these faster-replicating virus types which often result in more severe disease symptoms than those caused by ICV.

ICV surveillance in Hong Kong was stopped at the same time that SARS-CoV-2 struck, and measures introduced to limit its transmission will have had effects on transmission of other respiratory disease agents (101), including influenza (102). It is probable that viral interference caused by SARS-CoV-2 infection has also played a role, as supported by the lack of coinfections in COVID-19 patients (103, 104). For such viral interference to have occurred at a population level during the COVID-19 pandemic, high levels of infection must have occurred, and estimates of asymptomatic SARS-CoV-2 infection have ranged from 18 to 81% (105, 106), with high levels in health care workers (107). Many of these asymptomatic infectees have virus loads comparable to those reporting symptoms (108) and can shed virus for longer periods than those with symptoms (109). Given the high proportion of asymptomatic infectees among SARS-CoV-2-positive persons, their high virus loads, and extended periods of virus shedding, the potential of them being transmitters is substantial (110). It is unfortunate that surveillance of ICV in Hong Kong ceased at the time when SARS-CoV-2 started spreading worldwide; it would have been of interest to see how two viruses considered to cause widespread asymptomatic infection interacted.

### Conclusion.

To understand fully the epidemiology and virology of ICV infections, continued surveillance (i.e., testing of clinical specimens for the presence of ICV) is required in humans and pigs, with interspecies transmission possibly occurring in both directions (111), to provide samples for molecular analysis, together with renewed attempts at ICV isolation in culture systems and/or rescue via reverse genetics that will provide high titers of the viruses for phenotypic characterization. Only by such means can the outstanding questions of the interactions between ICV and its hosts be addressed in a material way.

## MATERIALS AND METHODS

### Respiratory sample collection and screening in Hong Kong SAR, China.

The surveillance system operating until week 07/2020 has been described previously (13). For the subsequent period considered here, up to week 52/2020, surveillance for influenza types A and B only together with SARS-CoV-2 has been reported. Weekly influenza surveillance has continued to be reported in Flu Express (https://www.chp.gov.hk/en/resources/29/304.html), and data relating to the numbers of COVID-19 tests performed and laboratory-confirmed cases of SARS-CoV-2 infection can be found, respectively, on the following websites: https://www.chp.gov.hk/files/pdf/statistics_on_covid_19_testing.pdf and https://www.info.gov.hk/gia/general/today.htm. All COVID-19 test results are fed back to, and positive SARS-CoV-2 detections followed up by, the Department of Health in Hong Kong.

### Samples shared with WHO CC, London, for ICV characterization.

Of archived ICV-positive clinical specimens, with collection dates from week 21/2019 to week 07/2020, those with threshold cycle (*C_T_*) values of ≤30 (*n* = 110) were shared as part of ongoing influenza surveillance conducted under the WHO Global Influenza Surveillance and Response System (GISRS). Brief details of the clinical specimens and those (*n* = 75) that yielded a full ORF sequence for at least one ICV gene are listed in Table S1. The samples shared to enable this study were clinical specimens which had completed their diagnostic purpose, rendering them surplus materials. Patient confidentiality was maintained throughout to ensure no infringement on patient privacy and protection of personal data.

### ICV gene sequencing.

All protocols relating to extraction of RNA from clinical specimens and generation of ICV-specific RT-PCR products, using highly conserved primer mixes, for Sanger sequencing of HE genes and next-generation sequencing (NGS) of whole genomes were published recently (13).

### Sequence assembly and curation.

HE gene sequences emerging from Sanger sequencing were assembled using the Staden package (http://staden.sourceforge.net/), and all sequences generated by NGS were assembled, and variants identified, using the pipeline described previously (13).

### Phylogenetic analyses.

Nucleotide alignments for complete ORFs of all gene products (HE, PB2, PB1, P3, NP, CM1, CM2, NS1, NS2), except for the HE gene, where the signal-peptide coding sequences were removed to give mature HEF amino acid numbering, were generated using BioEdit. These alignments were used to build maximum likelihood (ML) phylogenetic trees, with RAxML v8.2X (https://cme.h-its.org/exelixis/software.html), which were annotated with amino acid substitutions defining nodes and individual virus gene products using treesub (https://github.com/tamuri/treesub/blob/master/README.md) as described previously (13). Trees were rooted using sequences from C/Taylor/1233/47, except for NP, where the sequence from A/Ann Arbor/1/50 was used, and visualized using FigTree v1.4.4 (https://github.com/rambaut/figtree/releases).

Unrooted RAxML-generated ML trees, with strong bootstrap/statistical support, were used to check for temporal signal using TempEst (112) prior to BEAST analysis. All trees showed good correlation between diversity and time of sampling, with correlation coefficients of >0.89 for polymerase and NP segments and >0.75 for CM1 and NS1; CM2 and NS2 showed nontemporal signal and negative coefficients and were therefore excluded from the analysis.

Selection of clock and nucleotide substitution models was conducted using the nested sampling algorithm implemented in BEAST v2.5.2 (113). Based on these results, Bayesian phylogenetic analysis was subsequently estimated with a strict molecular clock, the Hasegawa-Kishino-Yano (HKY) model with gamma-distributed rates among sites with four rate categories and the Bayesian skyline plot as coalescent prior. Three independent Markov chain Monte Carlo (MCMC) chains were run for 50 million generations with sampling every 5,000 steps and were combined using LogCombiner within the BEAST package. Convergence was assessed based on effective sampling sizes (ESS) of >200 with a 10% burn-in using Tracer v1.7.1 (114). Nucleotide substitution rates were also generated as part of the BEAST analysis. Time-scaled maximum clade credibility (MCC) trees were generated using TreeAnnotator in BEAST and visualized using FigTree v1.4.4. Uncertainty in the estimates was indicated by 95% highest posterior density (95% HPD) intervals.

### Evolutionary selection analysis.

For each ORF, nucleotide substitution rates were calculated using BEAST. Associated estimations of *dN/dS* ratios were performed with Datamonkey (https://www.datamonkey.org/) using the algorithms SLAC (single-likelihood ancestor counting) and FEL (fixed effects likelihood) to evaluate the degree of selection pressure on each gene. Positive selection at a site was considered significant when a *P* value of <0.05 was obtained.

### Reassortment analysis.

RAxML-generated ML trees were colored based on the HE lineage, and pairwise incongruence was examined by constructing tanglegrams between the HE coding region versus all the internal gene ORF sequences using the dendextend R package (115), with the twines also colored by HE lineage. The program GiRaF (116) was employed to identify phylogenetic discordances across the data and produce, as output, a catalogue of reassorted segments. As input, 2,000 MrBayes-generated trees from each ORF were inferred with the GTR+Γ4+I substitution model and 1,000,000 generations with sampling every 500 iterations. These trees were used in GiRaF with burnin = 500 and default parameters. The default confidence threshold was 0.7, although all the events reported from our data had confidence levels of >0.9. Four independent runs were generated, where two did not include the HE gene to independently evaluate the reassortment relationships between internal gene ORFs.

### Data availability.

The accession numbers generated by the EpiFlu database of GISAID for all sequence data generated during this study (Table S1) and downloaded from GISAID for the generation of phylogenies (Table S4) are given in the supplemental material.
