# Supplementary material for: Temporal and Gene Reassortment Analysis of Influenza C Virus Outbreaks in Hong Kong, SAR, China
Source: J Virol. 2022 Feb 9;96(3):e01928-21. doi: 10.1128/jvi.01928-21 (PMC8826914; doi:10.1128/jvi.01928-21)
Supplement: Supplemental file 1 — Fig. S1 and S2 and Tables S1 to S4. Download jvi.01928-21-s0001.pdf, PDF file, 4.7 MB [file jvi.01928-21-s0001.pdf]

Figure S1. RaxML phylogenetic analysis of the coding regions of all seven ICV gene fragments

The phylogenies were generated as described in Materials and Methods and are zoomable. Viruses from the 2015-16, 2017-18 and 2019-20 outbreaks in Hong Kong are shown in orange, green and pink respectively. Group defining amino acid substitutions for all ORF coding sequences are shown on nodes and virus specific substitutions are shown after virus names. For all gene products alignments of full-length coding sequences, based on the following numbers of nucleotides, were used: PB2 2322, PB1 2262, P3 2127, HE 1923, NP 1695, CM1 726, CM2 417, NS1 738 and NS2 546. Where known, the HE gene lineage is indicated after the virus names in panels B-I, based on the lineage assignments in panel A: **T** C/Taylor/1233/47, **M** C/Mississippi/80, **Y** C/Yamagata/26/81, **A** C/Aichi/1/81, **K** C/Kanagawa/1/76 and **S** C/Sao Paulo/378/82 (**S1** C/Sao Paulo/378/82-S1, **S2** C/Sao Paulo/378/82-S2). The bar indicates the proportion of nucleotide changes between sequences. We gratefully acknowledge the authors, originating and submitting laboratories of the sequences from the EpiFlu^{TM} database of GISAID which were downloaded for use in the preparation of this manuscript (all submitters of data may be contacted directly via the GISAID website and the relevant sequence accession numbers are given in Table S4).

Figure S2. BEAST phylogenetic analysis of the coding regions of all seven ICV gene fragments

The phylogenies were generated as described in Materials and Methods, based on the same alignment files as those used to generate Figure S1, and are zoomable. Trees are shown only for the ORFs that showed good temporal signals (i.e. those for CM2 and NS2 are omitted – see

Materials and Methods). Virus names are given and HE lineages/sublineages are defined by colour as indicated in keys. For each ORF coding sequence tree amino acid substitutions defining nodes in the tree are shown. Nodes with ≥90% bootstrap support are indicated (●). For internal gene ORF trees (panels **B-G**) the two major lineages described by Matsuzaki et al are indicated (1).

1. Matsuzaki Y, Sugawara K, Furuse Y, Shimotai Y, Hongo S, Oshitani H, Mizuta K, Nishimura H. 2016. Genetic Lineage and Reassortment of Influenza C Viruses Circulating between 1947 and 2014. J Virol 90:8251-65.

Figure S1
A: HE

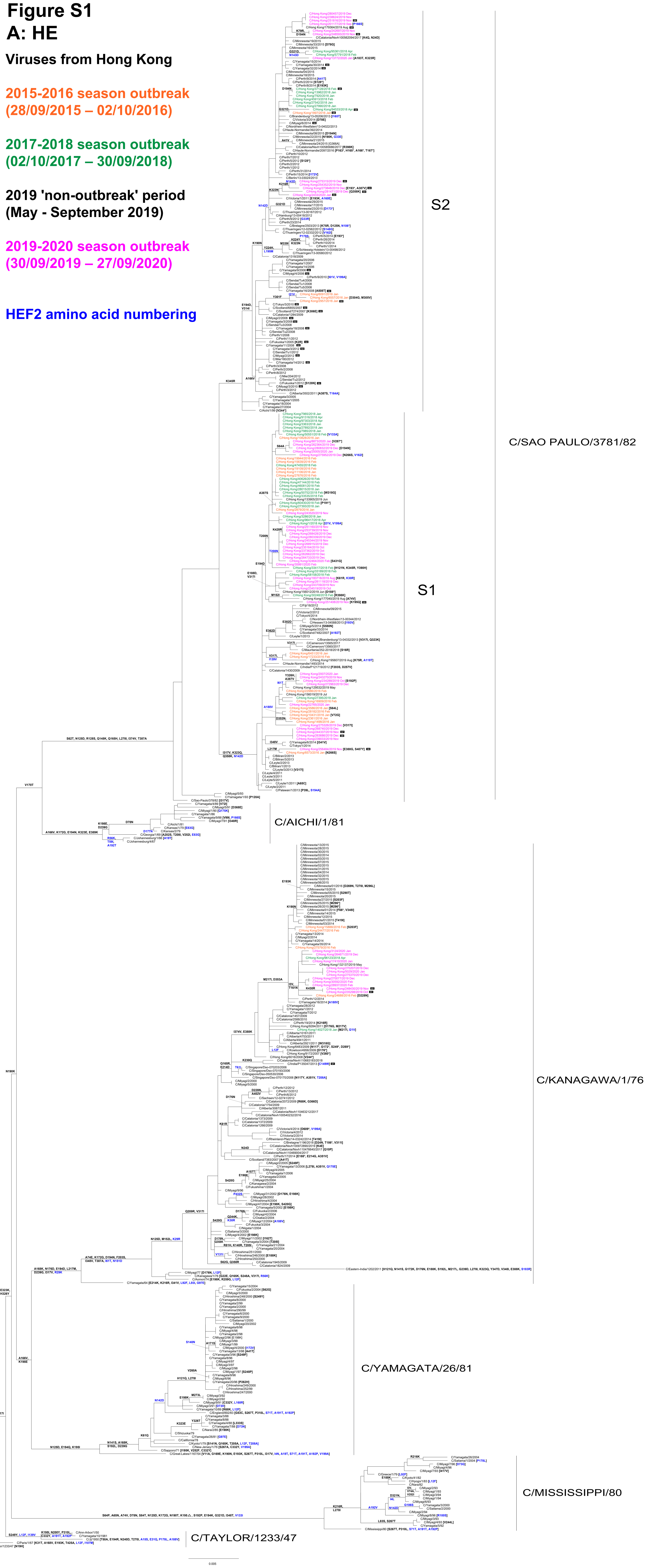

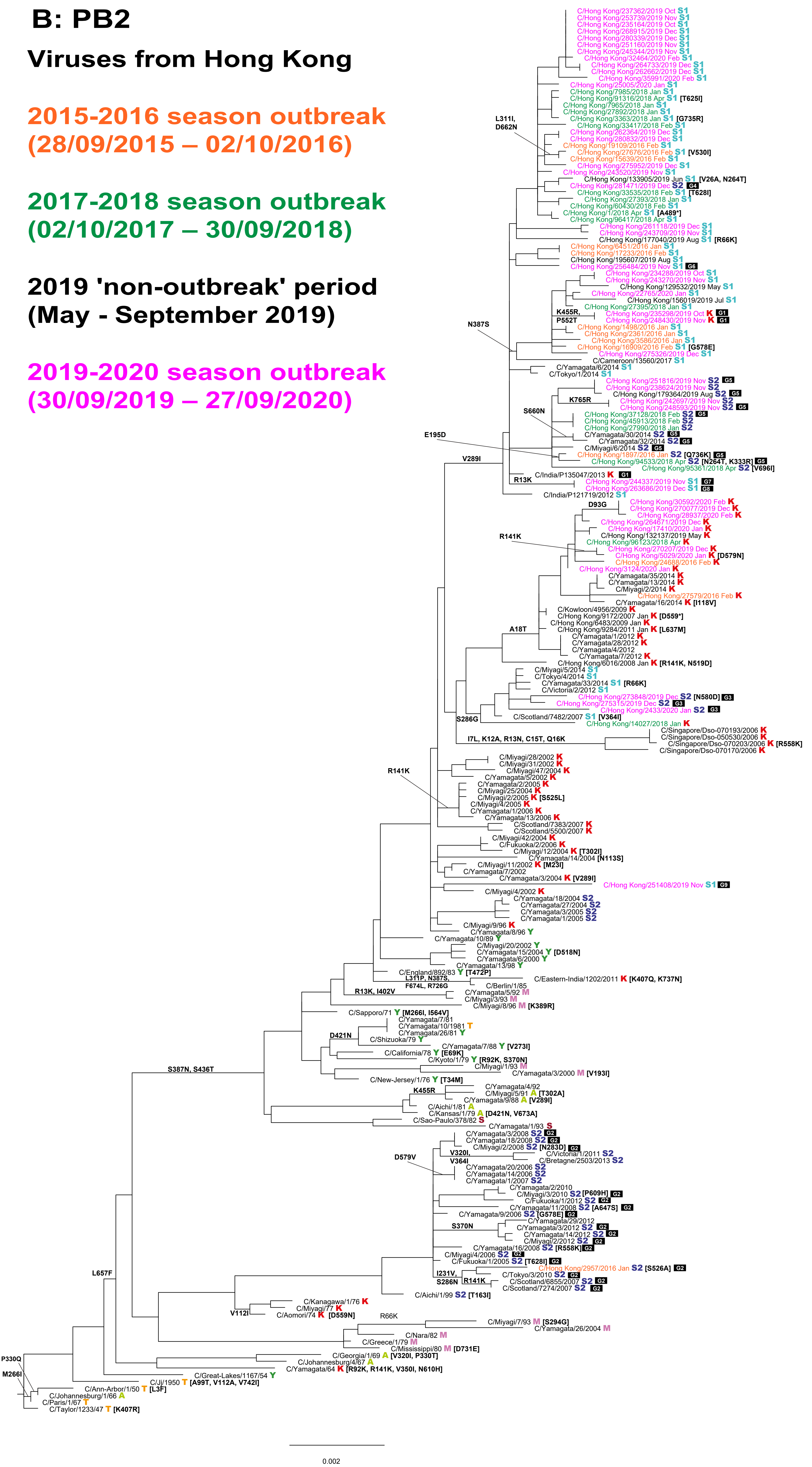

B: PB2
Viruses from Hong Kong
2015-2016 season outbreak
(28/09/2015 – 02/10/2016)
2017-2018 season outbreak
(02/10/2017 – 30/09/2018)
2019 'non-outbreak' period
(May - September 2019)
2019-2020 season outbreak
(30/09/2019 – 27/09/2020)
C/Hong Kong/237362/2019 Oct S1
C/Hong Kong/253739/2019 Nov S1
C/Hong Kong/235164/2019 Oct S1
C/Hong Kong/268915/2019 Dec S1
C/Hong Kong/280339/2019 Dec S1
C/Hong Kong/251160/2019 Nov S1
C/Hong Kong/245344/2019 Nov S1
C/Hong Kong/32464/2020 Feb S1
C/Hong Kong/264733/2019 Dec S1
C/Hong Kong/262662/2019 Dec S1
C/Hong Kong/35991/2020 Feb S1
C/Hong Kong/25005/2020 Jan S1
C/Hong Kong/7985/2018 Jan S1
C/Hong Kong/91316/2018 Apr S1 [T625I]
C/Hong Kong/7965/2018 Jan S1
C/Hong Kong/27892/2018 Jan S1
C/Hong Kong/3363/2018 Jan S1 [G735R]
C/Hong Kong/33417/2018 Feb S1
L311I, D662N
C/Hong Kong/262364/2019 Dec S1
C/Hong Kong/280832/2019 Dec S1
C/Hong Kong/19109/2016 Feb S1
C/Hong Kong/27676/2016 Feb S1 [V530I]
C/Hong Kong/15639/2016 Feb S1
C/Hong Kong/275952/2019 Dec S1
C/Hong Kong/243520/2019 Nov S1
C/Hong Kong/133905/2019 Jun S1 [V26A, N264T]
C/Hong Kong/281471/2019 Dec S2 G4
C/Hong Kong/33535/2018 Feb S1 [T628I]
C/Hong Kong/27393/2018 Jan S1
C/Hong Kong/60430/2018 Feb S1
C/Hong Kong/1/2018 Apr S1 [A489*]
C/Hong Kong/96417/2018 Apr S1
C/Hong Kong/261118/2019 Dec S1
C/Hong Kong/243709/2019 Nov S1
C/Hong Kong/177040/2019 Aug S1 [R66K]
C/Hong Kong/6451/2016 Jan S1
C/Hong Kong/17233/2016 Feb S1
C/Hong Kong/195607/2019 Aug S1
C/Hong Kong/256484/2019 Nov S1 G6
C/Hong Kong/234288/2019 Oct S1
C/Hong Kong/243270/2019 Nov S1
C/Hong Kong/129532/2019 May S1
C/Hong Kong/22765/2020 Jan S1
C/Hong Kong/156019/2019 Jul S1
C/Hong Kong/27395/2018 Jan S1
K455R, P552T
C/Hong Kong/235298/2019 Oct K G1
C/Hong Kong/248430/2019 Nov K G1
N387S
C/Hong Kong/1498/2016 Jan S1
C/Hong Kong/2361/2016 Jan S1
C/Hong Kong/3586/2016 Jan S1
C/Hong Kong/16909/2016 Feb S1 [G578E]
C/Hong Kong/275326/2019 Dec S1
C/Cameroon/13560/2017 S1
C/Yamagata/6/2014 S1
C/Tokyo/1/2014 S1
C/Hong Kong/251816/2019 Nov S2 G5
C/Hong Kong/238624/2019 Nov S2
C/Hong Kong/179364/2019 Aug S2 G5
K765R
C/Hong Kong/242697/2019 Nov S2 G5
C/Hong Kong/248593/2019 Nov S2 G5
S660N
C/Hong Kong/37128/2018 Feb S2 G5
C/Hong Kong/45913/2018 Feb S2
C/Hong Kong/27990/2018 Jan S2
C/Yamagata/30/2014 S2 G5
C/Yamagata/32/2014 S2 G5
E195D
C/Miyagi/6/2014 S2 G5
C/Hong Kong/1897/2016 Jan S2 [Q736K] G5
V289I
C/Hong Kong/94533/2018 Apr S2 [N264T, K333R] G5
C/Hong Kong/95361/2018 Apr S2 [V696I]
C/India/P135047/2013 K G1
R13K
C/Hong Kong/244337/2019 Nov S1 G7
C/Hong Kong/263686/2019 Dec S1 G8
C/India/P121719/2012 S1
D93G
C/Hong Kong/30592/2020 Feb K
C/Hong Kong/270077/2019 Dec K
C/Hong Kong/28937/2020 Feb K
C/Hong Kong/264671/2019 Dec K
R141K
C/Hong Kong/17410/2020 Jan K
C/Hong Kong/132137/2019 May K
C/Hong Kong/96123/2018 Apr K
C/Hong Kong/270207/2019 Dec K
C/Hong Kong/5029/2020 Jan K [D579N]
C/Hong Kong/24688/2016 Feb K
C/Hong Kong/3124/2020 Jan K
C/Yamagata/35/2014 K
C/Yamagata/13/2014 K
C/Miyagi/2/2014 K
C/Hong Kong/27579/2016 Feb K
C/Yamagata/16/2014 K [I118V]
C/Kowloon/4956/2009 K
C/Hong Kong/9172/2007 Jan K [D559*]
C/Hong Kong/6483/2009 Jan K
A18T
C/Hong Kong/9284/2011 Jan K [L637M]
C/Yamagata/1/2012 K
C/Yamagata/28/2012 K
C/Yamagata/4/2012
C/Yamagata/7/2012 K
C/Hong Kong/6016/2008 Jan K [R141K, N519D]
C/Miyagi/5/2014 S1
C/Tokyo/4/2014 S1
C/Yamagata/33/2014 S1 [R66K]
C/Victoria/2/2012 S1
C/Hong Kong/273848/2019 Dec S2 [N580D] G3
C/Hong Kong/275315/2019 Dec S2 G3
C/Hong Kong/2433/2020 Jan S2 G3
S286G
C/Scotland/7482/2007 S1 [V364I]
C/Hong Kong/14027/2018 Jan K
C/Singapore/Dso-070193/2006 K
C/Singapore/Dso-050530/2006 K
I7L, K12A, R13N, C15T, Q16K
C/Singapore/Dso-070203/2006 K [R558K]
C/Singapore/Dso-070170/2006 K
C/Miyagi/28/2002 K
C/Miyagi/31/2002 K
C/Miyagi/47/2004 K
R141K
C/Yamagata/5/2002 K
C/Yamagata/2/2005 K
C/Miyagi/25/2004 K
C/Miyagi/2/2005 K [S525L]
C/Miyagi/4/2005 K
C/Yamagata/1/2006 K
C/Yamagata/13/2006 K
C/Scotland/7383/2007 K
C/Scotland/5500/2007 K
C/Miyagi/42/2004 K
C/Fukuoka/2/2006 K
C/Miyagi/12/2004 K [T302I]
C/Yamagata/14/2004 [N113S]
C/Miyagi/11/2002 K [M23I]
C/Yamagata/7/2002
C/Yamagata/3/2004 K [V289I]
C/Hong Kong/251408/2019 Nov S1 G9
C/Miyagi/4/2002 K
C/Yamagata/18/2004 S2
C/Yamagata/27/2004 S2
C/Yamagata/3/2005 S2
C/Yamagata/1/2005 S2
C/Miyagi/9/96 K
C/Yamagata/8/96 Y
C/Yamagata/10/89 Y
C/Miyagi/20/2002 Y
C/Yamagata/15/2004 Y [D518N]
C/Yamagata/6/2000 Y
C/Yamagata/13/98 Y
C/England/892/83 Y [T472P]
L311P, N387S, F674L, R726G
C/Eastern-India/1202/2011 K [K407Q, K737N]
C/Berlin/1/85
R13K, I402V
C/Yamagata/5/92 M
C/Miyagi/3/93 M
C/Miyagi/8/96 M [K389R]
C/Sapporo/71 Y [M266I, I564V]
C/Yamagata/7/81
C/Yamagata/10/1981 T
D421N
C/Yamagata/26/81 Y
C/Shizuoka/79 Y
C/Yamagata/7/88 Y [V273I]
C/California/78 Y [E69K]
C/Kyoto/1/79 Y [R92K, S370N]
S387N, S436T
C/Miyagi/1/93 M
C/Yamagata/3/2000 M [V193I]
C/New-Jersey/1/76 Y [T34M]
C/Yamagata/4/92
K455R
C/Miyagi/5/91 A [T302A]
C/Yamagata/9/88 A [V289I]
C/Aichi/1/81 A
C/Kansas/1/79 A [D421N, V673A]
C/Sao-Paulo/378/82 S
C/Yamagata/1/93 S
C/Yamagata/3/2008 S2 G2
C/Yamagata/18/2008 S2 G2
C/Miyagi/2/2008 S2 [N283D] G2
V320I, V364I
C/Victoria/1/2011 S2
D579V
C/Bretagne/2503/2013 S2
C/Yamagata/20/2006 S2
C/Yamagata/14/2006 S2
C/Yamagata/1/2007 S2
C/Yamagata/2/2010
C/Miyagi/3/2010 S2 [P609H] G2
C/Fukuoka/1/2012 S2 G2
C/Yamagata/11/2008 S2 [A647S] G2
C/Yamagata/9/2006 S2 [G578E] G2
C/Yamagata/29/2012
S370N
C/Yamagata/3/2012 S2 G2
C/Yamagata/14/2012 S2 G2
C/Miyagi/2/2012 S2 G2
C/Yamagata/16/2008 S2 [R558K] G2
C/Miyagi/4/2006 S2 G2
C/Fukuoka/1/2005 S2 [T628I] G2
C/Hong Kong/2957/2016 Jan S2 [S526A] G2
L657F
I231V, S286N
R141K
C/Tokyo/3/2010 S2 G2
C/Scotland/6855/2007 S2 G2
C/Scotland/7274/2007 S2 G2
C/Aichi/1/99 S2 [T163I]
C/Kanagawa/1/76 K
C/Miyagi/77 K
V112I
C/Aomori/74 K [D559N]
R66K
C/Miyagi/7/93 M [S294G]
C/Yamagata/26/2004 M
C/Nara/82 M
C/Greece/1/79 M
C/Mississippi/80 M [D731E]
P330Q
C/Georgia/1/69 A [V320I, P330T]
C/Johannesburg/4/67 A
C/Yamagata/64 K [R92K, R141K, V350I, N610H]
M266I
C/Great-Lakes/1167/54 Y
C/JJ/1950 T [A99T, V112A, V742I]
C/Ann-Arbor/1/50 T [L3F]
C/Johannesburg/1/66 A
C/Paris/1/67 T
C/Taylor/1233/47 T [K407R]
0.002

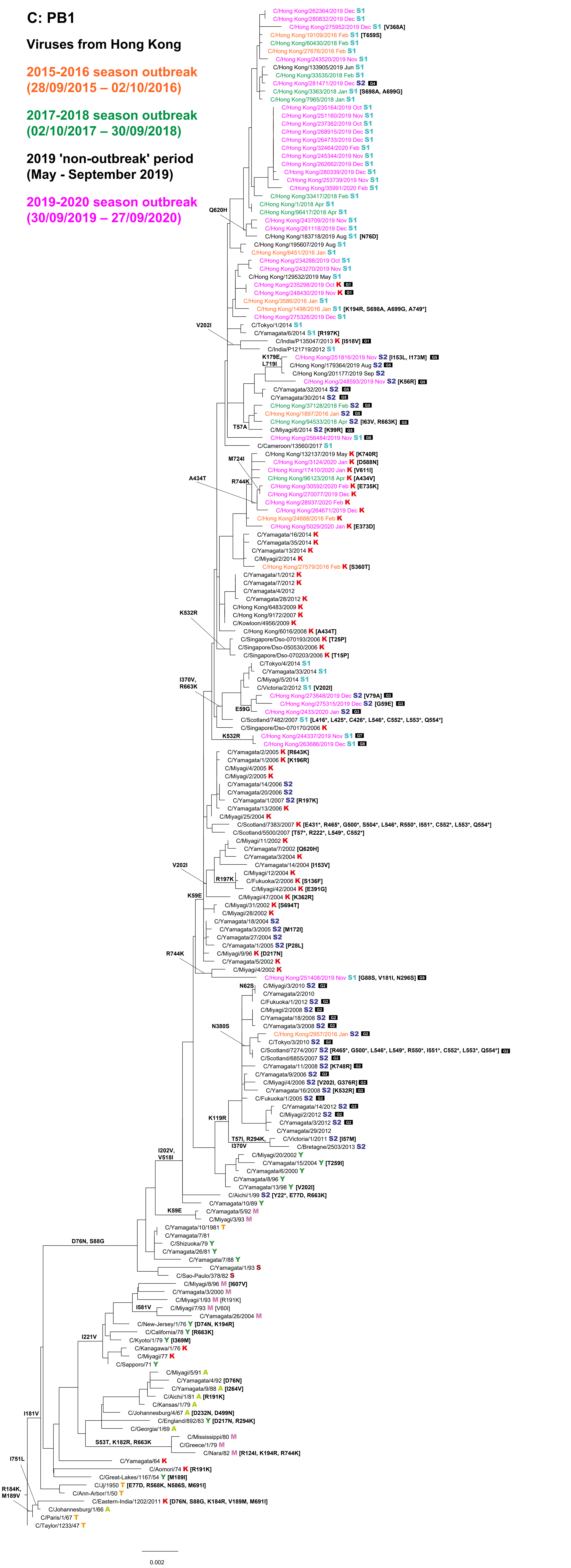
C: PB1
Viruses from Hong Kong
2015-2016 season outbreak
(28/09/2015 – 02/10/2016)
2017-2018 season outbreak
(02/10/2017 – 30/09/2018)
2019 'non-outbreak' period
(May - September 2019)
2019-2020 season outbreak
(30/09/2019 – 27/09/2020)
C/Hong Kong/262364/2019 Dec S1
C/Hong Kong/280832/2019 Dec S1
C/Hong Kong/275952/2019 Dec S1 [V368A]
C/Hong Kong/19109/2016 Feb S1 [T659S]
C/Hong Kong/60430/2018 Feb S1
C/Hong Kong/27876/2016 Feb S1
C/Hong Kong/243520/2019 Nov S1
C/Hong Kong/133905/2019 Jun S1
C/Hong Kong/33535/2018 Feb S1
C/Hong Kong/281471/2019 Dec S2 G4
C/Hong Kong/3363/2018 Jan S1 [S698A, A699G]
C/Hong Kong/7965/2018 Jan S1
C/Hong Kong/235164/2019 Oct S1
C/Hong Kong/251160/2019 Nov S1
C/Hong Kong/237362/2019 Oct S1
C/Hong Kong/268915/2019 Dec S1
C/Hong Kong/264733/2019 Dec S1
C/Hong Kong/32464/2020 Feb S1
C/Hong Kong/245344/2019 Nov S1
C/Hong Kong/262662/2019 Dec S1
C/Hong Kong/280339/2019 Dec S1
C/Hong Kong/253739/2019 Nov S1
C/Hong Kong/35991/2020 Feb S1
C/Hong Kong/33417/2018 Feb S1
Q620H
C/Hong Kong/1/2018 Apr S1
C/Hong Kong/96417/2018 Apr S1
C/Hong Kong/243709/2019 Nov S1
C/Hong Kong/261118/2019 Dec S1
C/Hong Kong/183718/2019 Aug S1 [N76D]
C/Hong Kong/195607/2019 Aug S1
C/Hong Kong/6451/2016 Jan S1
C/Hong Kong/234288/2019 Oct S1
C/Hong Kong/243270/2019 Nov S1
C/Hong Kong/129532/2019 May S1
C/Hong Kong/235298/2019 Oct K G1
C/Hong Kong/248430/2019 Nov K G1
C/Hong Kong/3586/2016 Jan S1
C/Hong Kong/1498/2016 Jan S1 [K194R, S698A, A699G, A749*]
C/Hong Kong/275326/2019 Dec S1
V202I
C/Tokyo/1/2014 S1
C/Yamagata/6/2014 S1 [R197K]
C/India/P135047/2013 K [I518V] G1
C/India/P121719/2012 S1
K179E
C/Hong Kong/251816/2019 Nov S2 [I153L, I173M] G5
L719I
C/Hong Kong/179364/2019 Aug S2 G5
C/Hong Kong/201177/2019 Sep S2
C/Hong Kong/248593/2019 Nov S2 [K56R] G5
C/Yamagata/32/2014 S2 G5
C/Yamagata/30/2014 S2 G5
C/Hong Kong/37128/2018 Feb S2 G5
C/Hong Kong/1897/2016 Jan S2 G5
C/Hong Kong/94533/2018 Apr S2 [I63V, R663K] G5
T57A
C/Miyagi/6/2014 S2 [K99R] G5
C/Hong Kong/256484/2019 Nov S1 G6
C/Cameroon/13560/2017 S1
M724I
C/Hong Kong/132137/2019 May K [K740R]
C/Hong Kong/3124/2020 Jan K [D588N]
A434T
C/Hong Kong/17410/2020 Jan K [V611I]
C/Hong Kong/96123/2018 Apr K [A434V]
R744K
C/Hong Kong/30592/2020 Feb K [E735K]
C/Hong Kong/270077/2019 Dec K
C/Hong Kong/28937/2020 Feb K
C/Hong Kong/264671/2019 Dec K
C/Hong Kong/24688/2016 Feb K
C/Hong Kong/5029/2020 Jan K [E373D]
C/Yamagata/16/2014 K
C/Yamagata/35/2014 K
C/Yamagata/13/2014 K
C/Miyagi/2/2014 K
C/Hong Kong/27579/2016 Feb K [S360T]
C/Yamagata/1/2012 K
C/Yamagata/7/2012 K
C/Yamagata/4/2012
C/Yamagata/28/2012 K
C/Hong Kong/6483/2009 K
C/Hong Kong/9172/2007 K
K532R
C/Kowloon/4956/2009 K
C/Hong Kong/6016/2008 K [A434T]
C/Singapore/Dso-070193/2006 K [T25P]
C/Singapore/Dso-050530/2006 K
C/Singapore/Dso-070203/2006 K [T15P]
C/Tokyo/4/2014 S1
C/Yamagata/33/2014 S1
I370V,
R663K
C/Miyagi/5/2014 S1
C/Victoria/2/2012 S1 [V202I]
C/Hong Kong/273848/2019 Dec S2 [V79A] G3
C/Hong Kong/275315/2019 Dec S2 [G59E] G3
E59G
C/Hong Kong/2433/2020 Jan S2 G3
C/Scotland/7482/2007 S1 [L416*, L425*, C426*, L546*, C552*, L553*, Q554*]
C/Singapore/Dso-070170/2006 K
K532R
C/Hong Kong/244337/2019 Nov S1 G7
C/Hong Kong/263686/2019 Dec S1 G8
C/Yamagata/2/2005 K [R643K]
C/Yamagata/1/2006 K [K196R]
C/Miyagi/4/2005 K
C/Miyagi/2/2005 K
C/Yamagata/14/2006 S2
C/Yamagata/20/2006 S2
C/Yamagata/1/2007 S2 [R197K]
C/Yamagata/13/2006 K
C/Miyagi/25/2004 K
C/Scotland/7383/2007 K [E431*, R465*, G500*, S504*, L546*, R550*, I551*, C552*, L553*, Q554*]
C/Scotland/5500/2007 [T57*, R222*, L549*, C552*]
C/Miyagi/11/2002 K
C/Yamagata/7/2002 [Q620H]
C/Yamagata/3/2004 K
C/Yamagata/14/2004 [I153V]
V202I
C/Miyagi/12/2004 K
R197K
C/Fukuoka/2/2006 K [S136F]
C/Miyagi/42/2004 K [E391G]
K59E
C/Miyagi/47/2004 K [K362R]
C/Miyagi/31/2002 K [S694T]
C/Miyagi/28/2002 K
C/Yamagata/18/2004 S2
C/Yamagata/3/2005 S2 [M172I]
C/Yamagata/27/2004 S2
C/Yamagata/1/2005 S2 [P28L]
R744K
C/Miyagi/9/96 K [D217N]
C/Yamagata/5/2002 K
C/Miyagi/4/2002 K
C/Hong Kong/251408/2019 Nov S1 [G88S, V181I, N296S] G9
N62S
C/Miyagi/3/2010 S2 G2
C/Yamagata/2/2010
C/Fukuoka/1/2012 S2 G2
C/Miyagi/2/2008 S2 G2
N380S
C/Yamagata/18/2008 S2 G2
C/Yamagata/3/2008 S2 G2
C/Hong Kong/2957/2016 Jan S2 G2
C/Tokyo/3/2010 S2 G2
C/Scotland/7274/2007 S2 [R465*, G500*, L546*, L549*, R550*, I551*, C552*, L553*, Q554*] G2
C/Scotland/6855/2007 S2 G2
C/Yamagata/11/2008 S2 [K748R] G2
C/Yamagata/9/2006 S2 G2
C/Miyagi/4/2006 S2 [V202I, G376R] G2
C/Yamagata/16/2008 S2 [K532R] G2
C/Fukuoka/1/2005 S2 G2
C/Yamagata/14/2012 S2 G2
K119R
C/Miyagi/2/2012 S2 G2
C/Yamagata/3/2012 S2 G2
C/Yamagata/29/2012
T57I, R294K,
I370V
C/Victoria/1/2011 S2 [I57M]
C/Bretagne/2503/2013 S2
I202V,
V518I
C/Miyagi/20/2002 Y
C/Yamagata/15/2004 Y [T259I]
C/Yamagata/6/2000 Y
C/Yamagata/8/96 Y
C/Yamagata/13/98 Y [V202I]
C/Aichi/1/99 S2 [Y22*, E77D, R663K]
C/Yamagata/10/89 Y
K59E
C/Yamagata/5/92 M
C/Miyagi/3/93 M
C/Yamagata/10/1981 T
C/Yamagata/7/81
D76N, S88G
C/Shizuoka/79 Y
C/Yamagata/26/81 Y
C/Yamagata/7/88 Y
C/Yamagata/1/93 S
C/Sao-Paulo/378/82 S
C/Miyagi/8/96 M [I607V]
C/Yamagata/3/2000 M
C/Miyagi/1/93 M [R191K]
I581V
C/Miyagi/7/93 M [V60I]
C/Yamagata/26/2004 M
C/New-Jersey/1/76 Y [D74N, K194R]
I221V
C/California/78 Y [R663K]
C/Kyoto/1/79 Y [I369M]
C/Kanagawa/1/76 K
C/Miyagi/77 K
C/Sapporo/71 Y
C/Miyagi/5/91 A
C/Yamagata/4/92 [D76N]
C/Yamagata/9/88 A [I264V]
C/Aichi/1/81 A [R191K]
C/Kansas/1/79 A
I181V
C/Johannesburg/4/67 A [D232N, D499N]
C/England/892/83 Y [D217N, R294K]
C/Georgia/1/69 A
C/Mississippi/80 M
S53T, K182R, R663K
C/Greece/1/79 M
C/Nara/82 M [R124I, K194R, R744K]
I751L
C/Yamagata/64 K
C/Aomori/74 K [R191K]
C/Great-Lakes/1167/54 Y [M189I]
C/Jj/1950 T [E77D, R568K, N586S, M691I]
C/Ann-Arbor/1/50 T
R184K,
M189V
C/Eastern-India/1202/2011 K [D76N, S88G, K184R, V189M, M691I]
C/Johannesburg/1/66 A
C/Paris/1/67 K
C/Taylor/1233/47 T
0.002

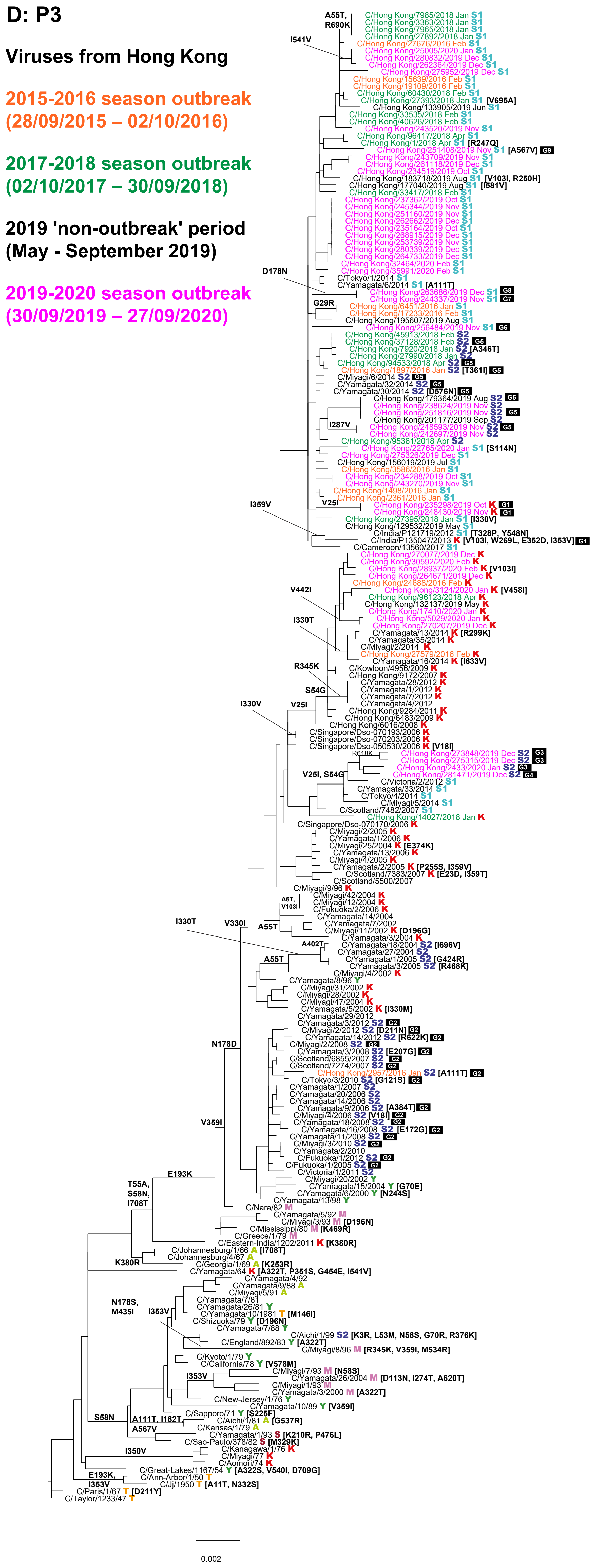
D: P3
Viruses from Hong Kong
2015-2016 season outbreak
(28/09/2015 – 02/10/2016)
2017-2018 season outbreak
(02/10/2017 – 30/09/2018)
2019 'non-outbreak' period
(May - September 2019)
2019-2020 season outbreak
(30/09/2019 – 27/09/2020)
A55T, R690K
I541V
C/Hong Kong/7985/2018 Jan S1
C/Hong Kong/3363/2018 Jan S1
C/Hong Kong/7965/2018 Jan S1
C/Hong Kong/27892/2018 Jan S1
C/Hong Kong/27676/2016 Feb S1
C/Hong Kong/25005/2020 Jan S1
C/Hong Kong/280832/2019 Dec S1
C/Hong Kong/262364/2019 Dec S1
C/Hong Kong/275952/2019 Dec S1
C/Hong Kong/15639/2016 Feb S1
C/Hong Kong/19109/2016 Feb S1
C/Hong Kong/60430/2018 Feb S1
C/Hong Kong/27393/2018 Jan S1 [V695A]
C/Hong Kong/133905/2019 Jun S1
C/Hong Kong/33535/2018 Feb S1
C/Hong Kong/40626/2018 Feb S1
C/Hong Kong/243520/2019 Nov S1
C/Hong Kong/96417/2018 Apr S1
C/Hong Kong/1/2018 Apr S1 [R247Q]
C/Hong Kong/251408/2019 Nov S1 [A567V] G9
C/Hong Kong/243709/2019 Nov S1
C/Hong Kong/261118/2019 Dec S1
C/Hong Kong/234519/2019 Oct S1
C/Hong Kong/183718/2019 Aug S1 [V103I, R250H]
C/Hong Kong/177040/2019 Aug S1 [I581V]
C/Hong Kong/33417/2018 Feb S1
C/Hong Kong/237362/2019 Oct S1
C/Hong Kong/245344/2019 Nov S1
C/Hong Kong/251160/2019 Nov S1
C/Hong Kong/262662/2019 Dec S1
C/Hong Kong/235164/2019 Oct S1
C/Hong Kong/268915/2019 Dec S1
C/Hong Kong/253739/2019 Nov S1
C/Hong Kong/280339/2019 Dec S1
C/Hong Kong/264733/2019 Dec S1
C/Hong Kong/32464/2020 Feb S1
C/Hong Kong/35991/2020 Feb S1
D178N
C/Tokyo/1/2014 S1
C/Yamagata/6/2014 S1 [A111T]
C/Hong Kong/263686/2019 Dec S1 G8
C/Hong Kong/244337/2019 Nov S1 G7
G29R
C/Hong Kong/6451/2016 Jan S1
C/Hong Kong/17233/2016 Feb S1
C/Hong Kong/195607/2019 Aug S1
C/Hong Kong/256484/2019 Nov S1 G6
C/Hong Kong/45913/2018 Feb S2
C/Hong Kong/37128/2018 Feb S2 G5
C/Hong Kong/7920/2018 Jan S2 [A346T]
C/Hong Kong/27990/2018 Jan S2
C/Hong Kong/94533/2018 Apr S2 G5
C/Hong Kong/1897/2016 Jan S2 [T361I] G5
C/Miyagi/6/2014 S2 G5
C/Yamagata/32/2014 S2 G5
C/Yamagata/30/2014 S2 [D576N] G5
C/Hong Kong/179364/2019 Aug S2 G5
C/Hong Kong/238624/2019 Nov S2
C/Hong Kong/251816/2019 Nov S2 G5
C/Hong Kong/201177/2019 Sep S2
I287V
C/Hong Kong/248593/2019 Nov S2 G5
C/Hong Kong/242697/2019 Nov S2
C/Hong Kong/95361/2018 Apr S2
C/Hong Kong/22765/2020 Jan S1 [S114N]
C/Hong Kong/275326/2019 Dec S1
C/Hong Kong/156019/2019 Jul S1
C/Hong Kong/3586/2016 Jan S1
C/Hong Kong/234288/2019 Oct S1
C/Hong Kong/243270/2019 Nov S1
C/Hong Kong/1498/2016 Jan S1
C/Hong Kong/2361/2016 Jan S1
V25I
C/Hong Kong/235298/2019 Oct K G1
C/Hong Kong/248430/2019 Nov K G1
I359V
C/Hong Kong/27395/2018 Jan S1 [I330V]
C/Hong Kong/129532/2019 May S1
C/India/P121719/2012 S1 [T328P, Y548N]
C/India/P135047/2013 K [V103I, W269L, E352D, I353V] G1
C/Cameroon/13560/2017 S1
C/Hong Kong/270077/2019 Dec K
C/Hong Kong/30592/2020 Feb K
C/Hong Kong/28937/2020 Feb K [V103I]
C/Hong Kong/264671/2019 Dec K
C/Hong Kong/24688/2016 Feb K
V442I
C/Hong Kong/3124/2020 Jan K [V458I]
C/Hong Kong/96123/2018 Apr K
C/Hong Kong/132137/2019 May K
C/Hong Kong/17410/2020 Jan K
I330T
C/Hong Kong/5029/2020 Jan K
C/Hong Kong/270207/2019 Dec K
C/Yamagata/13/2014 K [R299K]
C/Yamagata/35/2014 K
C/Miyagi/2/2014 K
C/Hong Kong/27579/2016 Feb K
C/Yamagata/16/2014 K [I633V]
R345K
C/Kowloon/4956/2009 K
C/Hong Kong/9172/2007 K
C/Yamagata/28/2012 K
S54G
C/Yamagata/1/2012 K
C/Yamagata/7/2012 K
I330V
V25I
C/Yamagata/4/2012
C/Hong Kong/9284/2011 K
C/Hong Kong/6483/2009 K
C/Hong Kong/6016/2008 K
C/Singapore/Dso-070193/2006 K
C/Singapore/Dso-070203/2006 K
C/Singapore/Dso-050530/2006 K [V18I]
R618K
C/Hong Kong/273848/2019 Dec S2 G3
C/Hong Kong/275315/2019 Dec S2 G3
C/Hong Kong/2433/2020 Jan S2 G3
C/Hong Kong/281471/2019 Dec S2 G4
V25I, S54G
C/Victoria/2/2012 S1
C/Yamagata/33/2014 S1
C/Tokyo/4/2014 S1
C/Miyagi/5/2014 S1
C/Scotland/7482/2007 S1
C/Hong Kong/14027/2018 Jan K
C/Singapore/Dso-070170/2006 K
C/Miyagi/2/2005 K
C/Yamagata/1/2006 K
C/Miyagi/25/2004 K [E374K]
C/Yamagata/13/2006 K
C/Miyagi/4/2005 K
C/Yamagata/2/2005 K [P255S, I359V]
C/Scotland/7383/2007 K [E23D, I359T]
C/Scotland/5500/2007
C/Miyagi/9/96 K
A6T, V103I
C/Miyagi/42/2004 K
C/Miyagi/12/2004 K
C/Fukuoka/2/2006 K
I330T
V330I
A55T
C/Yamagata/14/2004
C/Yamagata/7/2002
C/Miyagi/11/2002 K [D196G]
A402T
C/Yamagata/3/2004 K
C/Yamagata/18/2004 S2 [I696V]
A55T
C/Yamagata/27/2004 S2
C/Yamagata/1/2005 S2 [G424R]
C/Yamagata/3/2005 S2 [R468K]
C/Miyagi/4/2002 K
C/Yamagata/8/96 Y
C/Miyagi/31/2002 K
C/Miyagi/28/2002 K
C/Miyagi/47/2004 K
C/Yamagata/5/2002 K [I330M]
C/Yamagata/29/2012
C/Yamagata/3/2012 S2 G2
C/Miyagi/2/2012 S2 [D211N] G2
C/Yamagata/14/2012 S2 [R622K] G2
N178D
C/Miyagi/2/2008 S2 G2
C/Yamagata/3/2008 S2 [E207G] G2
C/Scotland/6855/2007 S2 G2
C/Scotland/7274/2007 S2 G2
C/Hong Kong/2957/2016 Jan S2 [A111T] G2
C/Tokyo/3/2010 S2 [G121S] G2
C/Yamagata/1/2007 S2
C/Yamagata/20/2006 S2
C/Yamagata/14/2006 S2
C/Yamagata/9/2006 S2 [A384T] G2
C/Miyagi/4/2006 S2 [V18I] G2
V359I
C/Yamagata/18/2008 S2 G2
C/Yamagata/16/2008 S2 [E172G] G2
C/Yamagata/11/2008 S2 G2
C/Miyagi/3/2010 S2 G2
C/Yamagata/2/2010
C/Fukuoka/1/2012 S2 G2
C/Fukuoka/1/2005 S2 G2
E193K
C/Victoria/1/2011 S2
T55A, S58N, I708T
C/Miyagi/20/2002 Y
C/Yamagata/15/2004 Y [G70E]
C/Yamagata/6/2000 Y [N244S]
C/Yamagata/13/98 Y
C/Nara/82 M
C/Yamagata/5/92 M
C/Miyagi/3/93 M [D196N]
C/Mississippi/80 M [K469R]
C/Greece/1/79 M
C/Eastern-India/1202/2011 K [K380R]
C/Johannesburg/1/66 A [I708T]
C/Johannesburg/4/67 A
K380R
C/Georgia/1/69 A [K253R]
C/Yamagata/64 K [A322T, P351S, G454E, I541V]
C/Yamagata/4/92
C/Yamagata/9/88 A
C/Miyagi/5/91 A
N178S, M435I
I353V
C/Yamagata/7/81
C/Yamagata/26/81 Y
C/Yamagata/10/1981 T [M146I]
C/Shizuoka/79 Y [D196N]
C/Yamagata/7/88 Y
C/Aichi/1/99 S2 [K3R, L53M, N58S, G70R, R376K]
C/England/892/83 Y [A322T]
C/Miyagi/8/96 M [R345K, V359I, M534R]
C/Kyoto/1/79 Y
C/California/78 Y [V578M]
I353V
C/Miyagi/7/93 M [N58S]
C/Yamagata/26/2004 M [D113N, I274T, A620T]
C/Miyagi/1/93 M
C/Yamagata/3/2000 M [A322T]
C/New-Jersey/1/76 Y
C/Yamagata/10/89 Y [V359I]
S58N
A111T, I182T
C/Sapporo/71 Y [S225F]
A567V
C/Aichi/1/81 A [G537R]
C/Kansas/1/79 A
C/Yamagata/1/93 S [K210R, P476L]
C/Sao-Paulo/378/82 S [M329K]
I350V
C/Kanagawa/1/76 K
C/Miyagi/77 K
C/Aomori/74 K
E193K
C/Great-Lakes/1167/54 Y [A322S, V540I, D709G]
C/Ann-Arbor/1/50 T
I353V
C/Jj/1950 T [A11T, N332S]
C/Paris/1/67 T [D211Y]
C/Taylor/1233/47 T
0.002

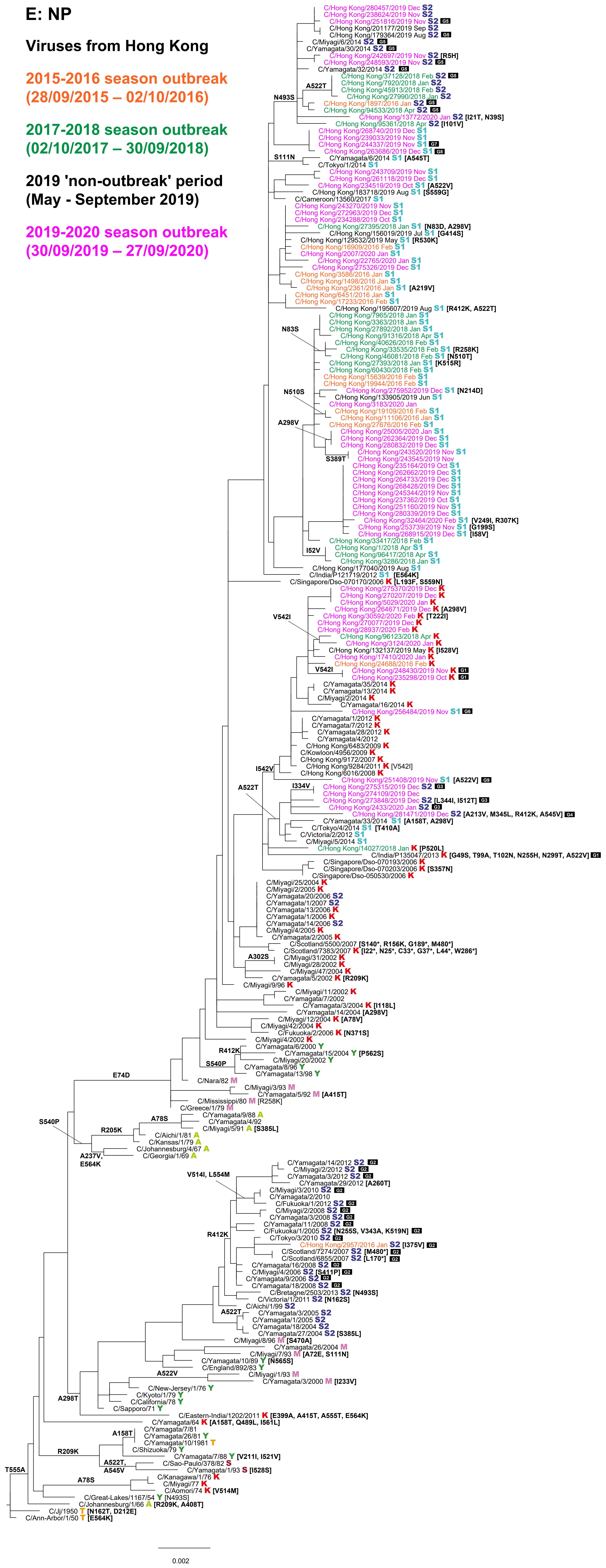
E: NP
Viruses from Hong Kong
2015-2016 season outbreak
(28/09/2015 – 02/10/2016)
2017-2018 season outbreak
(02/10/2017 – 30/09/2018)
2019 'non-outbreak' period
(May - September 2019)
2019-2020 season outbreak
(30/09/2019 – 27/09/2020)
0.002

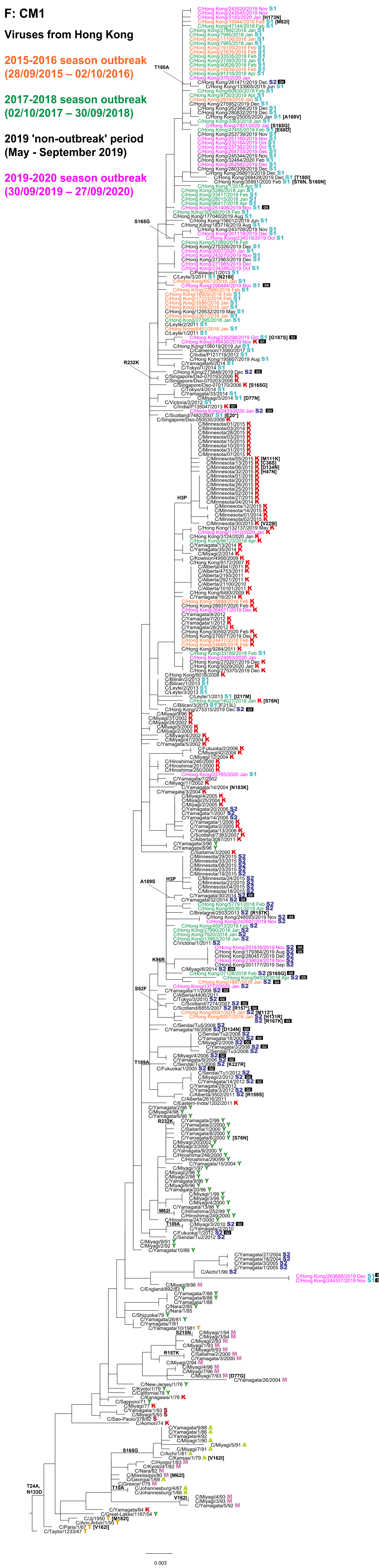
F: CM1
Viruses from Hong Kong
2015-2016 season outbreak
(28/09/2015 – 02/10/2016)
2017-2018 season outbreak
(02/10/2017 – 30/09/2018)
2019 'non-outbreak' period
(May - September 2019)
2019-2020 season outbreak
(30/09/2019 – 27/09/2020)
0.003

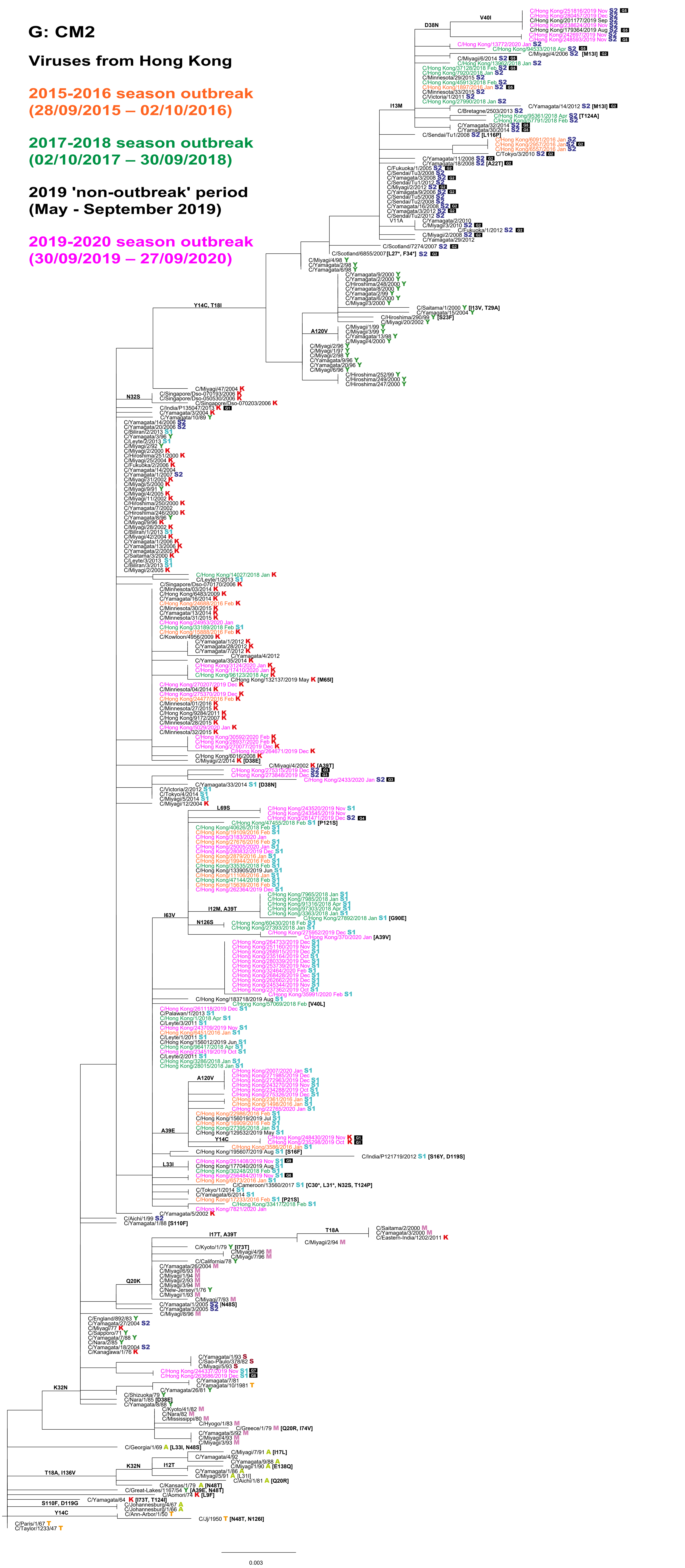
G: CM2
Viruses from Hong Kong
2015-2016 season outbreak
(28/09/2015 – 02/10/2016)
2017-2018 season outbreak
(02/10/2017 – 30/09/2018)
2019 'non-outbreak' period
(May - September 2019)
2019-2020 season outbreak
(30/09/2019 – 27/09/2020)
0.003

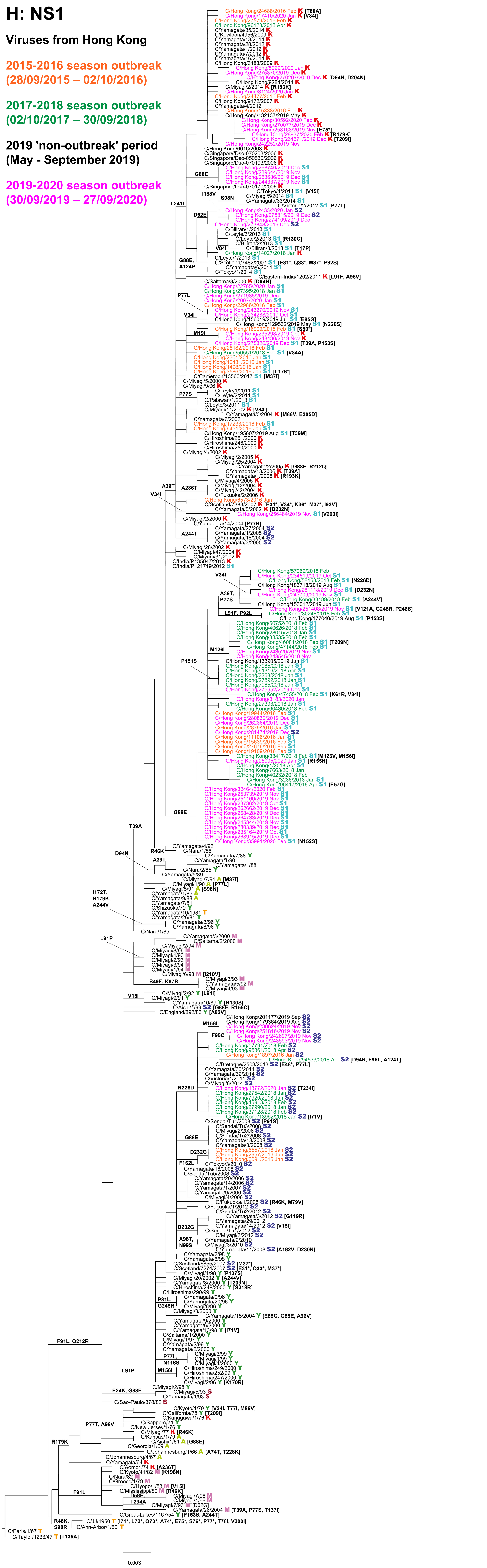

H: NS1
Viruses from Hong Kong
2015-2016 season outbreak
(28/09/2015 – 02/10/2016)
2017-2018 season outbreak
(02/10/2017 – 30/09/2018)
2019 'non-outbreak' period
(May - September 2019)
2019-2020 season outbreak
(30/09/2019 – 27/09/2020)
0.003

I: NS2

Viruses from Hong Kong

2015-2016 season outbreak
(28/09/2015 – 02/10/2016)

2017-2018 season outbreak
(02/10/2017 – 30/09/2018)

2019 'non-outbreak' period
(May - September 2019)

2019-2020 season outbreak
(30/09/2019 – 27/09/2020)

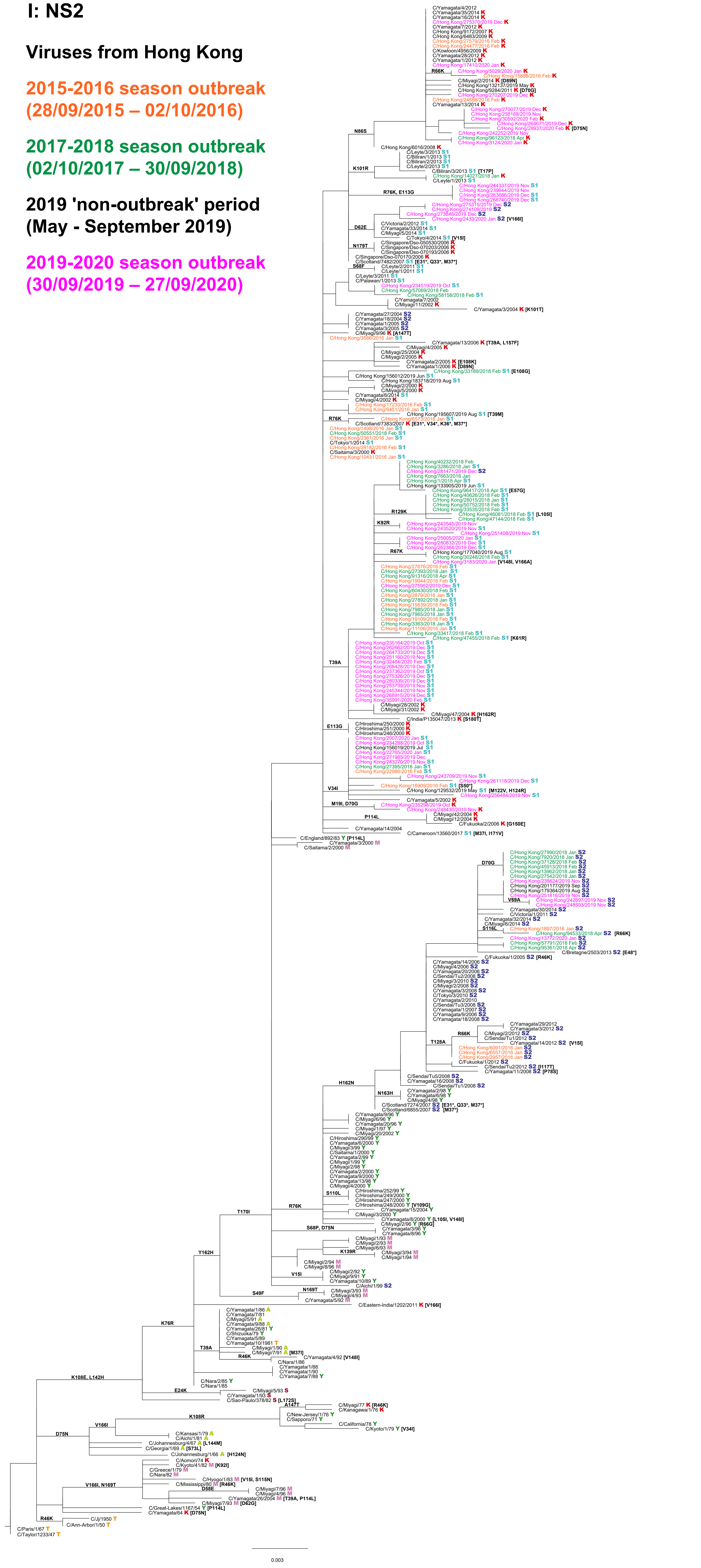

Figure S2

A:HE

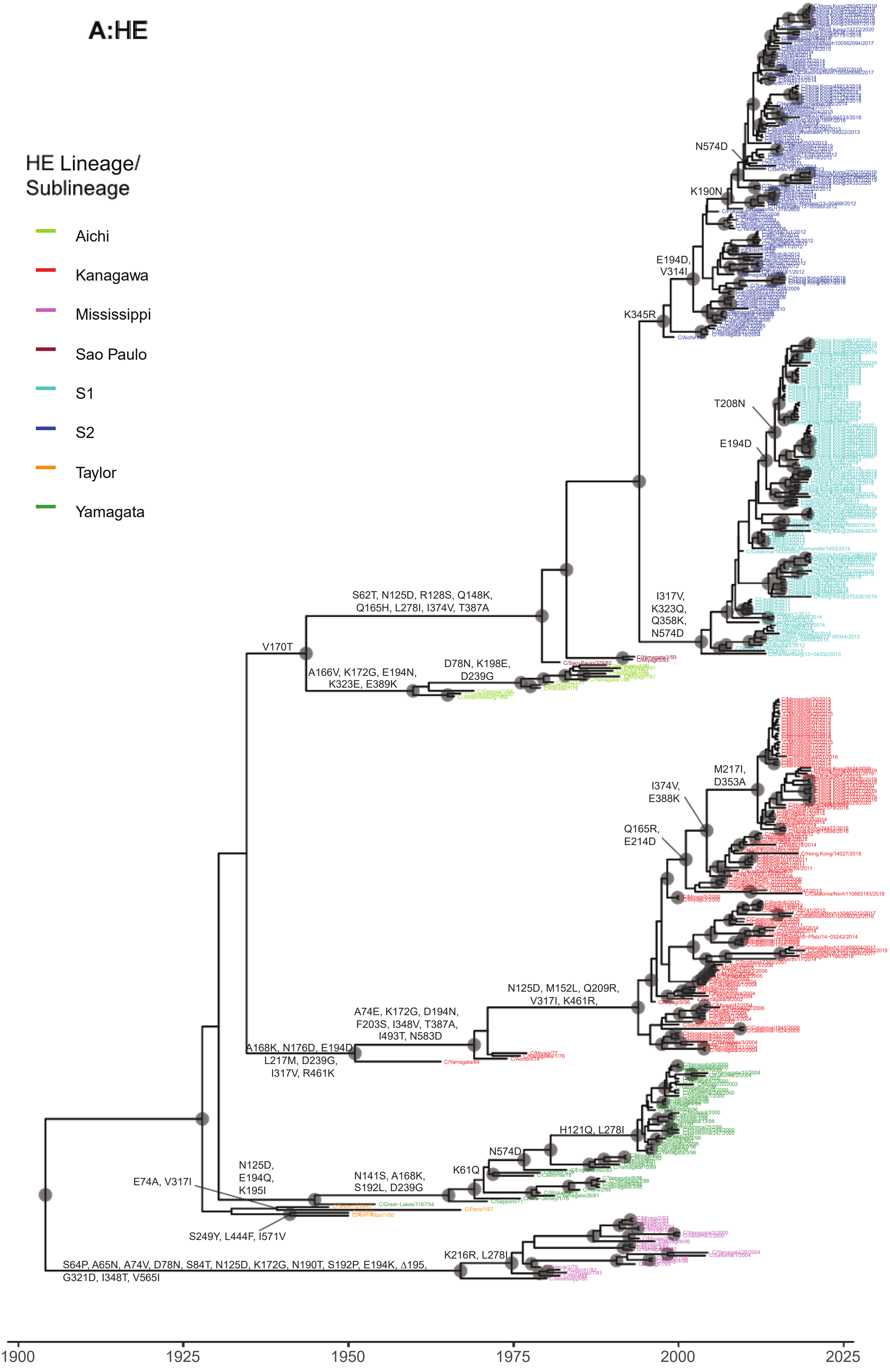

B: PB2

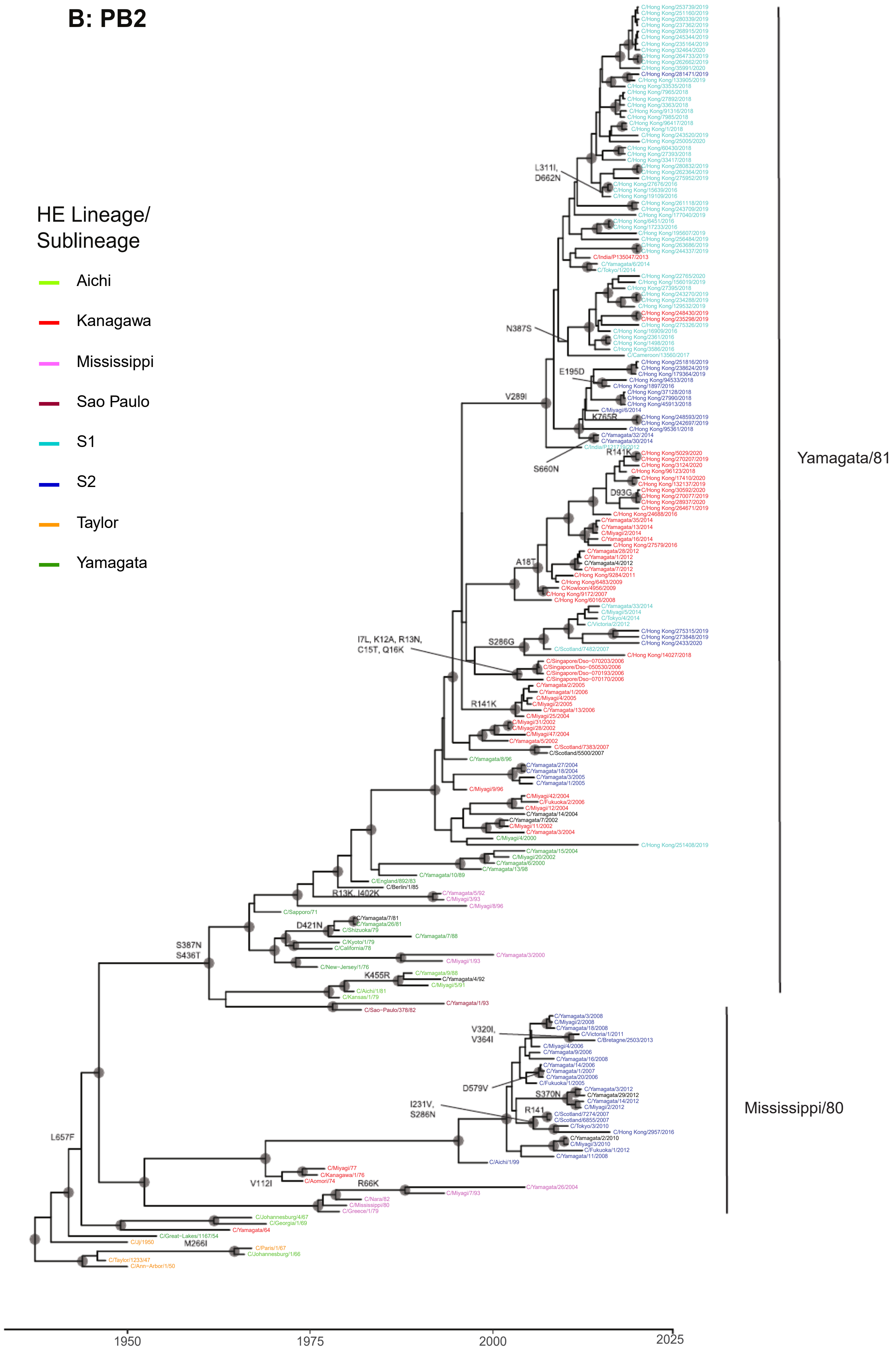
HE Lineage/
Sublineage
Aichi
Kanagawa
Mississippi
Sao Paulo
S1
S2
Taylor
Yamagata
L311I,
D662N
N387S
E195D
K765R
V289I
S660N
R141K
D93G
A18T
S286G
I7L, K12A, R13N,
C15T, Q16K
R141K
R13K, I402K
D421N
S387N
S436T
K455R
V320I,
V364I
D579V
S370N
I231V,
S286N
R141
L657F
V112I
R66K
M266I
Yamagata/81
Mississippi/80
1950
1975
2000
2025

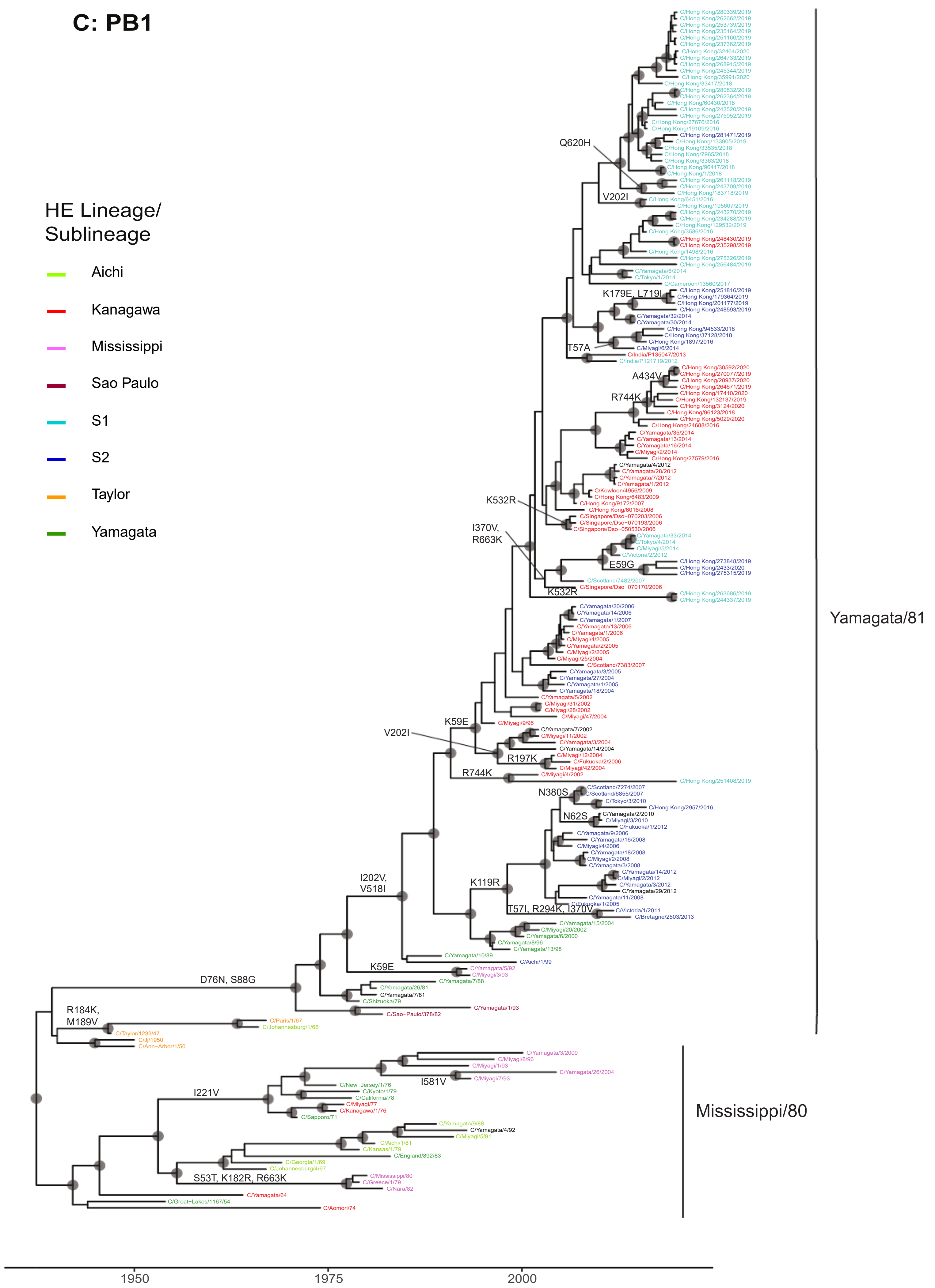

C: PB1
HE Lineage/ Sublineage
Aichi
Kanagawa
Mississippi
Sao Paulo
S1
S2
Taylor
Yamagata
Q620H
V202I
K179E, L719I
T57A
A434V
R744K
K532R
I370V, R663K
E59G
K532R
K59E
V202I
R197K
R744K
N380S
N62S
I202V, V518I
K119R
T57I, R294K, I370V
K59E
D76N, S88G
R184K, M189V
I221V
I581V
S53T, K182R, R663K
Yamagata/81
Mississippi/80
1950
1975
2000

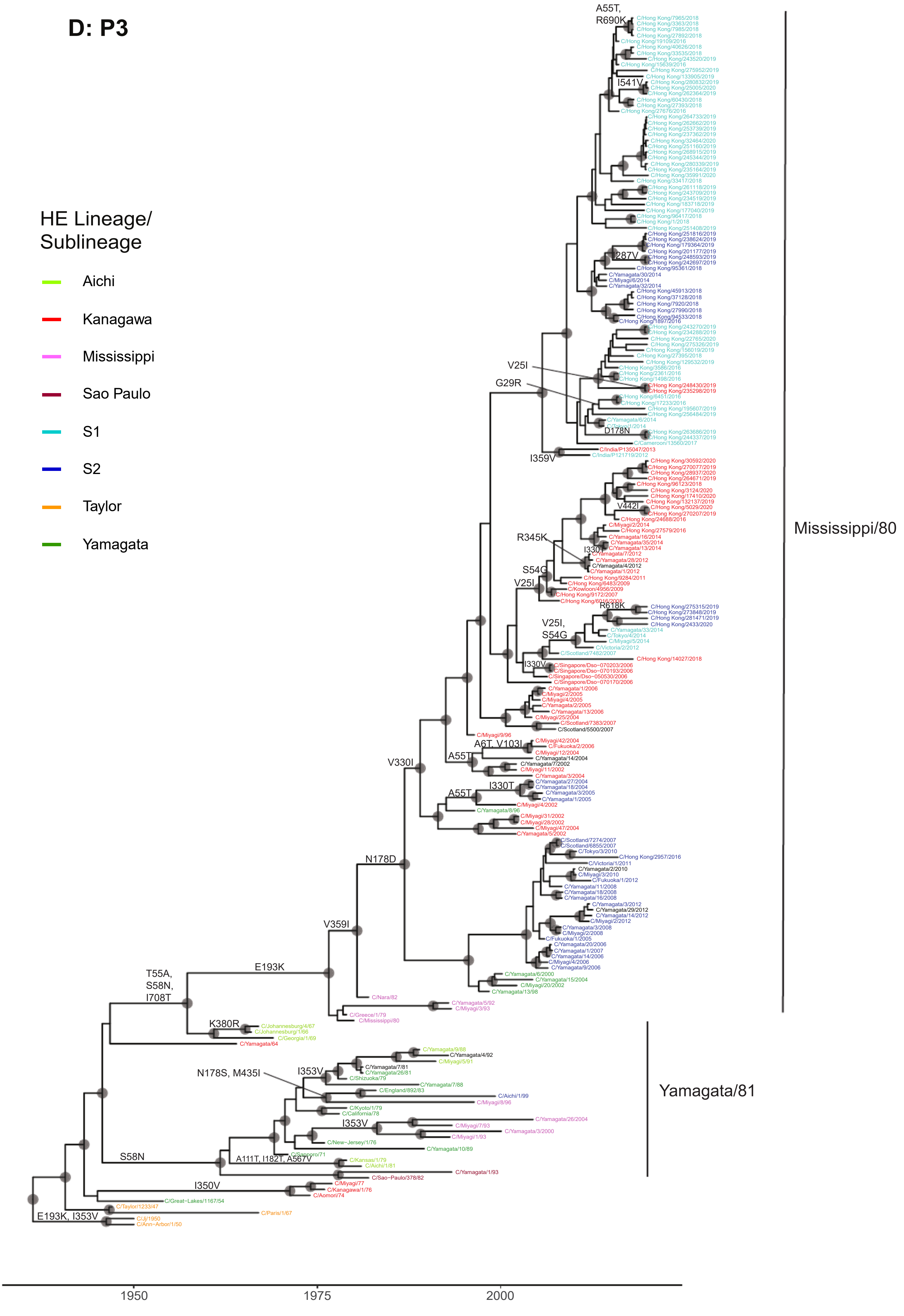
D: P3
HE Lineage/
Sublineage
Aichi
Kanagawa
Mississippi
Sao Paulo
S1
S2
Taylor
Yamagata
Mississippi/80
Yamagata/81
A55T,
R690K
I541V
I287V
V25I
G29R
D178N
I359V
V442I
R345K
I330T
S54G
V25I
R618K
V25I,
S54G
I330V
A6T, V103I
A55T
V330I
A55T
I330T
N178D
V359I
E193K
T55A,
S58N,
I708T
K380R
N178S, M435I
I353V
I353V
S58N
A111T, I182T, A567V
I350V
E193K, I353V
1950
1975
2000

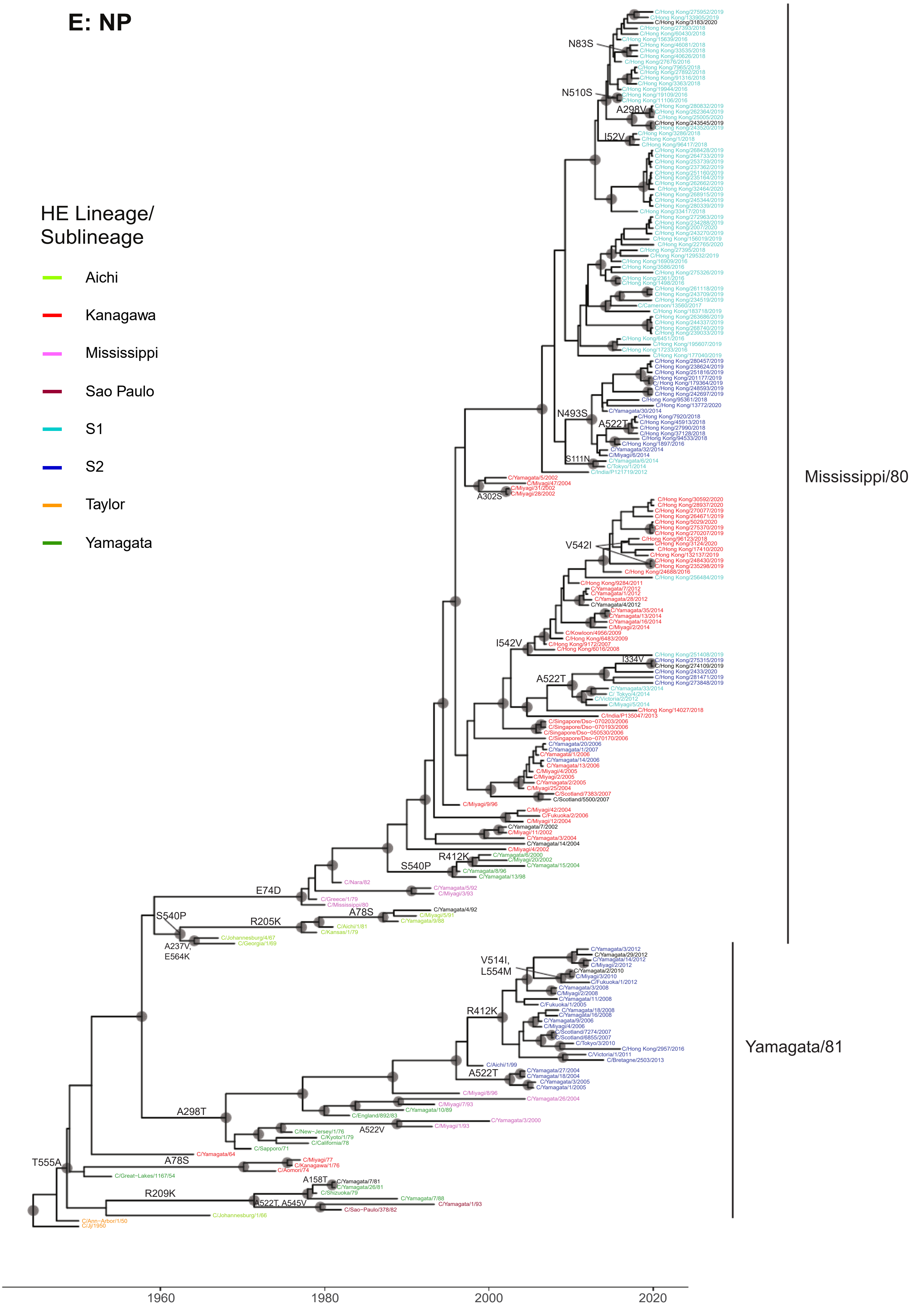
E: NP
HE Lineage/ Sublineage
Aichi
Kanagawa
Mississippi
Sao Paulo
S1
S2
Taylor
Yamagata
Mississippi/80
Yamagata/81
N83S
N510S
A298V
I52V
N493S
A522T
S111N
A302S
V542I
I542V
I334V
A522T
R412K
S540P
E74D
S540P
R205K
A78S
A237V, E564K
V514I, L554M
R412K
A522T
A298T
A522V
T555A
A78S
A158T
R209K
A522T, A545V
1960
1980
2000
2020

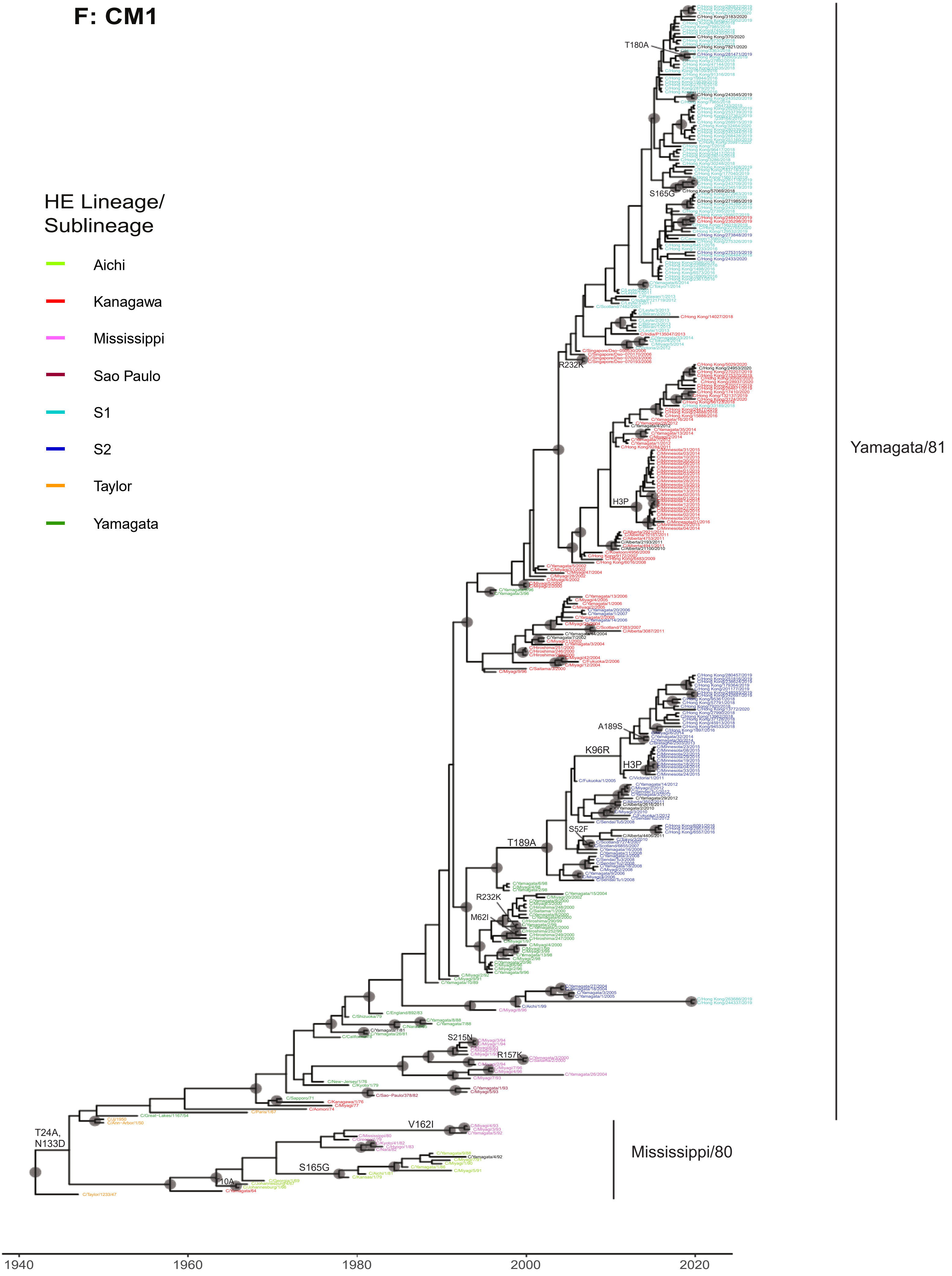
F: CM1
HE Lineage/ Sublineage
Aichi
Kanagawa
Mississippi
Sao Paulo
S1
S2
Taylor
Yamagata
Yamagata/81
Mississippi/80
T180A
S165G
R232K
H3P
A189S
K96R
H3P
S52F
T189A
R232K
M62I
S215N
R157K
V162I
S165G
T24A,
N133D
10A
1940
1960
1980
2000
2020

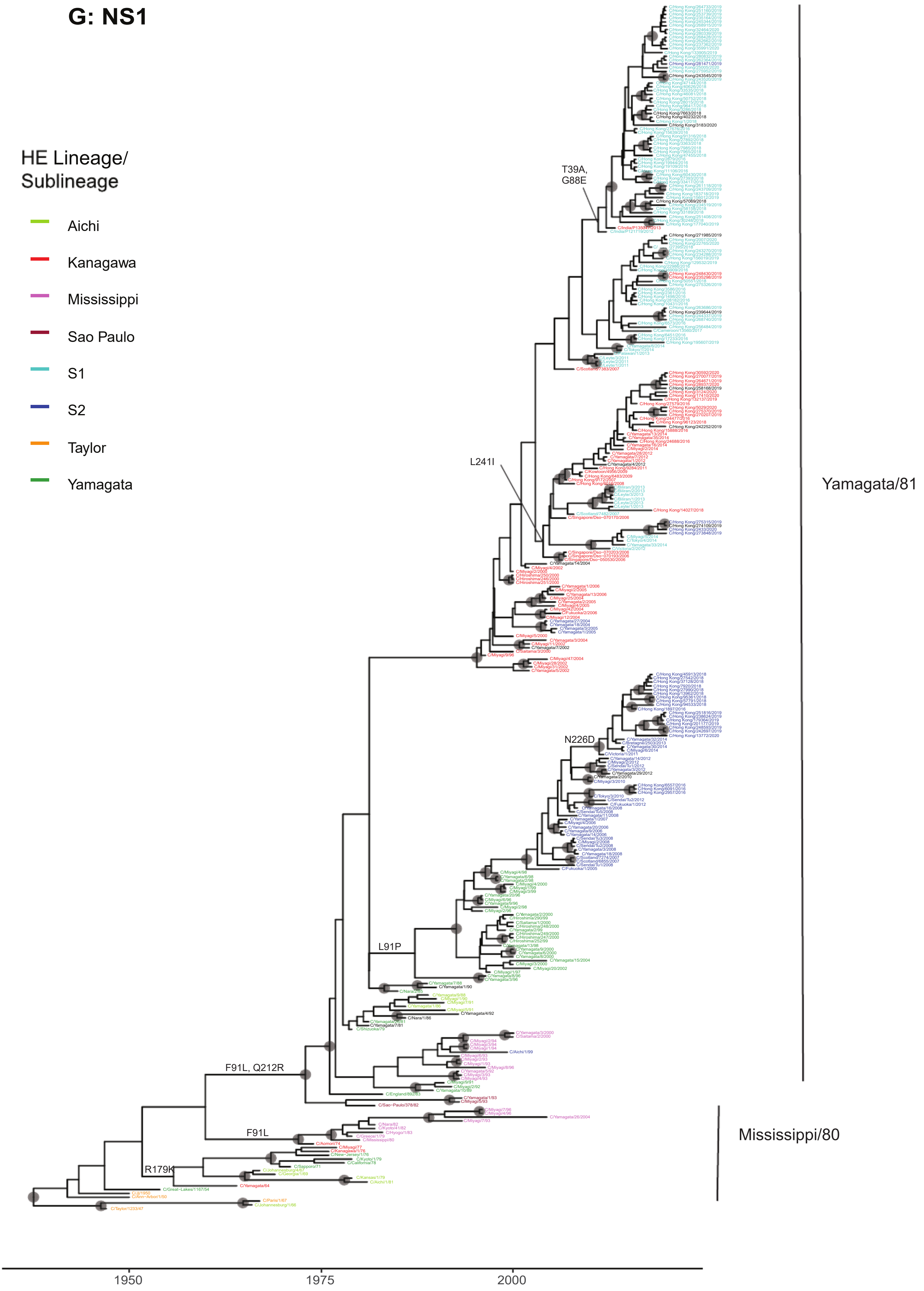
G: NS1
HE Lineage/
Sublineage
Aichi
Kanagawa
Mississippi
Sao Paulo
S1
S2
Taylor
Yamagata
T39A,
G88E
L241I
N226D
L91P
F91L, Q212R
F91L
R179K
Yamagata/81
Mississippi/80
1950
1975
2000

Table S1. ICV-positive clinical specimens from Hong Kong 2019-20 outbreak

Virus	Collection Date	Sample Type[a]	Patient and clinical details				Complete ORF sequence[f]							GISAID isolate accession no[g]	Period[h]
			Age[b]	Sex[c]	On Admission[d]	Co-infection[e]	HE	PB2	PB1	P3	NP	CM	NS		
C/Hong Kong/129110/2019	2019-05-26	NPA	4	F	Mycoplasma bronchitis	ND									
C/Hong Kong/129532/2019	2019-05-27	NS	6	M		ND	Y	Y	Y	Y	Y	Y	Y	EPI_ISL_574705	
C/Hong Kong/132137/2019	2019-05-29	NPS	88	F	Congestive heart failure	ND	Y	Y	Y	Y	Y	Y	Y	EPI_ISL_574706	**Weeks 21-26**
C/Hong Kong/133905/2019	2019-06-02	NPA	5	F	Pneumonia / ILI with travel history	ND	Y	Y	Y	Y	Y	Y	Y	EPI_ISL_574707	
C/Hong Kong/156012/2019	2019-06-30	NPA	1	M	Pre-surgical infection testing	PIV3	Y					Y	Y	EPI_ISL_574708	
C/Hong Kong/156019/2019	2019-07-01	NPA	75	M	Pneumonia	ND	Y	Y		Y	Y	Y	Y	EPI_ISL_574709	
C/Hong Kong/177040/2019	2019-08-01	NPS	35	M	URTI	ND	Y	Y		Y	Y	Y	Y	EPI_ISL_574711	
C/Hong Kong/179364/2019	2019-08-03	NPS	74	F	Fever	ND	Y	Y	Y	Y	Y	Y	Y	EPI_ISL_574712	
C/Hong Kong/183718/2019	2019-08-10	NPS	9	M	Fever	ND	Y		Y	Y	Y	Y	Y	EPI_ISL_574713	
C/Hong Kong/195607/2019	2019-08-29	NPA	9	F	Pneumonia / ILI with travel history	ND	Y	Y	Y	Y	Y	Y	Y	EPI_ISL_574714	
C/Hong Kong/200475/2019	2019-09-05	NPS	62	M	Intracranial haemorrhage	ND									
C/Hong Kong/201177/2019	2019-09-06	NPA	3	M	URTI	ND	Y		Y	Y	Y	Y	Y	EPI_ISL_574716	**Weeks 27-43**
C/Hong Kong/208097/2019	2019-09-18	NPS	1	F	URTI	AV									
C/Hong Kong/210837/2019	2019-09-22	NPS	67	F	Post-chemotheraoy fever	ND									
C/Hong Kong/232479/2019	2019-10-24	NPA	4	F	Viral illness	ND									
C/Hong Kong/234288/2019	2019-10-26	NPS	21	M	SOB	ND	Y	Y	Y	Y	Y	Y	Y	EPI_ISL_574718	
C/Hong Kong/234519/2019	2019-10-26	NPA	3	F	URTI	ND	Y			Y	Y	Y	Y	EPI_ISL_574719	
C/Hong Kong/235164/2019	2019-10-27	NPS	1	M	convulsion	ND	Y	Y	Y	Y	Y	Y	Y	EPI_ISL_574720	
C/Hong Kong/235298/2019	2019-10-28	NPS	3	F	CAP	ND	Y	Y	Y	Y	Y	Y	Y	EPI_ISL_574721	
C/Hong Kong/237362/2019	2019-10-30	NPS	2	M	Pneumonia	PIV1	Y	Y	Y	Y	Y	Y	Y	EPI_ISL_574722	
C/Hong Kong/237392/2019	2019-10-31	TS	2	M	Pneumonia	PIV1									
C/Hong Kong/239033/2019	2019-11-02	NPA	4	M	URTI	ND	Y				Y			EPI_ISL_574724	
C/Hong Kong/238624/2019	2019-11-03	NPS	<1	M	URTI	ND	Y	Y		Y	Y	Y	Y	EPI_ISL_574723	
C/Hong Kong/239644/2019	2019-11-04	NPA	4	M		ND							Y	EPI_ISL_574777	
C/Hong Kong/242252/2019	2019-11-07	NPS	2	M	GE	ND							Y	EPI_ISL_574778	
C/Hong Kong/242697/2019	2019-11-08	NPA	1	F	Fever convulsion, URTI	ND	Y	Y		Y	Y	Y	Y	EPI_ISL_574725	
C/Hong Kong/243709/2019	2019-11-09	NPA	1	M	URTI	ND	Y	Y	Y	Y	Y	Y	Y	EPI_ISL_574729	
C/Hong Kong/243270/2019	2019-11-10	NPS	2	F	Fever	ND	Y	Y	Y	Y	Y	Y	Y	EPI_ISL_574726	
C/Hong Kong/243520/2019	2019-11-10	NPS	1	M	Mild croup	ND	Y	Y	Y	Y	Y	Y	Y	EPI_ISL_574728	
C/Hong Kong/243545/2019	2019-11-11	NPS	4	F	Fever, URTI	ND					Y	Y	Y	EPI_ISL_574770	
C/Hong Kong/243548/2019	2019-11-11	NPS	5	M	Cough, hemoptysis	ND									
C/Hong Kong/244337/2019	2019-11-11	NPA	1	M	URTI	PV	Y	Y	Y	Y	Y	Y	Y	EPI_ISL_574730	**Weeks 44-52**
C/Hong Kong/245344/2019	2019-11-11	NPA	4	M	Fever	ND	Y	Y	Y	Y	Y	Y	Y	EPI_ISL_574731	
C/Hong Kong/244683/2019	2019-11-12	NPS	4	M	URTI	ND									
C/Hong Kong/247568/2019	2019-11-15	NPA	3	M	Wheezy attack	ND									
C/Hong Kong/248593/2019	2019-11-16	NPA	4	F	Viral illness	ND	Y	Y	Y	Y	Y	Y	Y	EPI_ISL_574733	
C/Hong Kong/248430/2019	2019-11-17	NPS	87	M	Fever, decreased general conditon	ND	Y	Y	Y	Y	Y	Y	Y	EPI_ISL_574732	
C/Hong Kong/248667/2019	2019-11-17	NPS	3	F	Fever	AH1									
C/Hong Kong/251160/2019	2019-11-21	NPS	1	M	Fever	PV	Y	Y	Y	Y	Y	Y	Y	EPI_ISL_574735	
C/Hong Kong/251408/2019	2019-11-20	NPA	43	M	Fever	ND	Y	Y	Y	Y	Y	Y	Y	EPI_ISL_574736	
C/Hong Kong/251816/2019	2019-11-22	NPS	2	M	Febrile convulsion	ND	Y	Y	Y	Y	Y	Y	Y	EPI_ISL_574737	
C/Hong Kong/253739/2019	2019-11-25	NPA	3	M	Febrile convulsion	ND	Y	Y	Y	Y	Y	Y	Y	EPI_ISL_574738	
C/Hong Kong/254352/2019	2019-11-25	NPA	<1	M	URTI	AV	Y							EPI_ISL_574739	
C/Hong Kong/254368/2019	2019-11-25	NPA	4	M	SVT	PV									
C/Hong Kong/254524/2019	2019-11-25	NPA	3	M	Febrile convulsion	ND									
C/Hong Kong/256484/2019	2019-11-28	NPS	94	F	Symptomatic hyponatremia	ND	Y	Y	Y	Y	Y	Y	Y	EPI_ISL_574740	

Virus	Collection Date	Sample Type[a]	Patient and clinical details				Complete ORF sequence[f]							GISAID isolate accession no[g]	Period[h]
			Age[b]	Sex[c]	On Admission[d]	Co-infection[e]	HE	PB2	PB1	P3	NP	CM	NS		
C/Hong Kong/258168/2019	2019-11-30	NPA	1	F	Aspiration pneumonia	ND							Y	EPI_ISL_574864	
C/Hong Kong/261118/2019	2019-12-04	NPS	46	F	URTI	ND	Y	Y	Y	Y	Y	Y	Y	EPI_ISL_574741	
C/Hong Kong/262364/2019	2019-12-07	NPS	4	M	Fever	ND	Y	Y	Y	Y	Y	Y	Y	EPI_ISL_574742	
C/Hong Kong/262662/2019	2019-12-07	NPA	2	M	Viral illness	PV	Y	Y	Y	Y	Y	Y	Y	EPI_ISL_574743	
C/Hong Kong/263686/2019	2019-12-09	NPA	1	F	Fever	ND	Y	Y	Y	Y	Y	Y	Y	EPI_ISL_574744	
C/Hong Kong/264671/2019	2019-12-10	NPA	3	M	Pneumonia	ND	Y	Y	Y	Y	Y	Y	Y	EPI_ISL_574745	
C/Hong Kong/264733/2019	2019-12-10	NPA	6	F	Fever	ND	Y	Y	Y	Y	Y	Y	Y	EPI_ISL_574746	
C/Hong Kong/268428/2019	2019-12-15	NPS	3	F	URTI	ND	Y				Y	Y	Y	EPI_ISL_574747	
C/Hong Kong/268740/2019	2019-12-15	NPA	4	M	Haematochezia	ND	Y				Y		Y	EPI_ISL_574748	
C/Hong Kong/268915/2019	2019-12-15	NPA	4	F	Pneumonia / ILI	ND	Y	Y	Y	Y	Y	Y	Y	EPI_ISL_574749	
C/Hong Kong/269640/2019	2019-12-16	NPA	4	M	Fever	ND									
C/Hong Kong/270077/2019	2019-12-17	NPS	31	F	Fever	ND	Y	Y	Y	Y	Y	Y	Y	EPI_ISL_574750	
C/Hong Kong/270207/2019	2019-12-17	NPS	5	M	Fever	ND	Y	Y		Y	Y	Y	Y	EPI_ISL_574751	
C/Hong Kong/271723/2019	2019-12-17	NPA	3	F	Fever	ND									
C/Hong Kong/271985/2019	2019-12-18	NPA	4	M	Chest infection	ND						Y	Y	EPI_ISL_574772	
C/Hong Kong/272831/2019	2019-12-19	NPS	1	F	Left bloody otorrhoea	ND									
C/Hong Kong/272963/2019	2019-12-19	NPA	3	F	Pneumonia	ND	Y				Y	Y		EPI_ISL_574752	
C/Hong Kong/273848/2019	2019-12-20	NPS	4	F	Hyperkalemia	PV	Y	Y	Y	Y	Y	Y	Y	EPI_ISL_574753	
C/Hong Kong/274109/2019	2019-12-22	NPS	3	F	URTI	ND					Y		Y	EPI_ISL_574776	
C/Hong Kong/275115/2019	2019-12-21	NPS	47	F	Ovarian cancer	ND									
C/Hong Kong/275315/2019	2019-12-22	NPA	5	M	Febrile convulsion	ND	Y	Y	Y	Y	Y	Y	Y	EPI_ISL_574754	
C/Hong Kong/275326/2019	2019-12-23	NPA	2	F	Febrile convulsion	ND	Y	Y	Y	Y	Y	Y	Y	EPI_ISL_574755	
C/Hong Kong/275370/2019	2019-12-23	NPS	94	M	Dizziness	ND	Y				Y	Y	Y	EPI_ISL_574756	
C/Hong Kong/275699/2019	2019-12-23	NPA	5	F	URTI / fever	ND									
C/Hong Kong/275952/2019	2019-12-23	NPA	3	M	Fever	AV	Y	Y	Y	Y	Y	Y	Y	EPI_ISL_574757	
C/Hong Kong/276129/2019	2019-12-23	NPA	4	M	Fever	ND									
C/Hong Kong/277010/2019	2019-12-26	NPA	3	M	URTI	ND									
C/Hong Kong/280339/2019	2019-12-29	NPS	5	F	Febrile convulsion	ND	Y	Y	Y	Y	Y	Y	Y	EPI_ISL_574758	
C/Hong Kong/280457/2019	2019-12-29	NPS	19	F	Desaturation (oxygen)	ND	Y				Y	Y		EPI_ISL_574759	
C/Hong Kong/280832/2019	2019-12-29	NPS	37	F	URTI	ND	Y	Y	Y	Y	Y	Y	Y	EPI_ISL_574760	
C/Hong Kong/281471/2019	2019-12-31	NPS	3	M	ILI	ND	Y	Y	Y	Y	Y	Y	Y	EPI_ISL_574761	
C/Hong Kong/359/2020	2020-01-02	NPS	1	F	URTI	ND									
C/Hong Kong/370/2020	2020-01-02	NPS	1	M	Fever	ND						Y		EPI_ISL_574774	
C/Hong Kong/466/2020	2020-01-01	NPA	2	M	Febrile convulsion	ND									
C/Hong Kong/1478/2020	2019-12-31	NPS	4	M	Preseptal cellulitis	ND									
C/Hong Kong/2007/2020	2020-01-03	NPA	3	M	Generalized lymphadenopathy	ND	Y				Y	Y	Y	EPI_ISL_574715	
C/Hong Kong/2433/2020	2020-01-03	TS	2	F	Acute tonsilitis	AV	Y	Y	Y	Y	Y	Y	Y	EPI_ISL_574727	
C/Hong Kong/2763/2020	2020-01-04	NPS	18	F	Asthma	ND									
C/Hong Kong/3124/2020	2020-01-03	NPS	4	F	Influenza	ND	Y	Y	Y	Y	Y	Y	Y	EPI_ISL_574764	
C/Hong Kong/3183/2020	2020-01-05	NPS	1	M	Erythema multiforme (allergy)	ND					Y	Y	Y	EPI_ISL_574773	
C/Hong Kong/5029/2020	2020-01-06	NPS	18	M	Seizure	ND	Y	Y	Y	Y	Y	Y	Y	EPI_ISL_574767	**Weeks 1-7**
C/Hong Kong/7558/2020	2020-01-09	NPS	46	M		ND									
C/Hong Kong/7821/2020	2020-01-09	TS	3	F		RSV						Y		EPI_ISL_574775	
C/Hong Kong/8873/2020	2020-01-10	NPS	7	M	Fever	ND	Y							EPI_ISL_574768	
C/Hong Kong/8928/2020	2020-01-10	NPS	2	M	GE	PIV2									
C/Hong Kong/8880/2020	2020-01-10	NPS	43	F	Pneumonia	AH3									
C/Hong Kong/13772/2020	2020-01-16	NPS	51	F	Anaemia	ND	Y				Y	Y	Y	EPI_ISL_574769	
C/Hong Kong/17410/2020	2020-01-20	NPS	60	F	Fever	ND	Y	Y	Y	Y	Y	Y	Y	EPI_ISL_574710	
C/Hong Kong/18808/2020	2020-01-23	NPA+TS	2	F		RSV									
C/Hong Kong/19349/2020	2020-01-22	TS	1	F	Febrile convulsion, viral illness	ND									
C/Hong Kong/19921/2020	2020-01-23	NPS	32	F	URTI	ND									

Virus	Collection Date	Sample Type[a]	Patient and clinical details				Complete ORF sequence[f]							GISAID isolate accession no[g]	Period[h]
			Age[b]	Sex[c]	On Admission[d]	Co-infection[e]	HE	PB2	PB1	P3	NP	CM	NS		
C/Hong Kong/20723/2020	2020-01-24	NPS	33	M	Bronchitis	ND									
C/Hong Kong/22765/2020	2020-01-28	NPS	75	F	Sepsis / pneumonia / tachycardia	ND	Y	Y		Y	Y	Y	Y	EPI_ISL_574717	
C/Hong Kong/22490/2020	2020-01-26	NPA+TS	2	F		PIV2, RSV, PV									
C/Hong Kong/24953/2020	2020-01-30	NPA+TS	33	M		ND						Y		EPI_ISL_574771	
C/Hong Kong/25005/2020	2020-01-29	NPS+TS	32	M	URTI	ND	Y	Y		Y	Y	Y	Y	EPI_ISL_574734	
C/Hong Kong/25366/2020	2020-01-30	TS	9	F	URTI	ND									
C/Hong Kong/22575/2020	2020-01-27	NPA+TS	5	M		PV									
C/Hong Kong/26566/2020	2020-01-30	NPA+TS	60	M	Fever	ND									
C/Hong Kong/25988/2020	2020-01-30	Sputum	56	M	Pneumonia	AH1									
C/Hong Kong/28937/2020	2020-02-03	NPS	31	F		ND	Y	Y	Y	Y	Y	Y	Y	EPI_ISL_574762	
C/Hong Kong/30592/2020	2020-02-05	NPS	80	F	COPD	ND	Y	Y	Y	Y	Y	Y	Y	EPI_ISL_574763	
C/Hong Kong/32464/2020	2020-02-08	NPS	63	M	Pneumonia	ND	Y	Y	Y	Y	Y	Y	Y	EPI_ISL_574765	
C/Hong Kong/35991/2020	2020-02-12	NPS	38	F	ILI	ND	Y	Y	Y	Y		Y	Y	EPI_ISL_574766	
**Number of complete ORF sequences determined/number of clinical specimens yielding at least one complete ORF sequence**							65	51	46	54	64	67	67	75	
**Percentage (of 110 clinical specimens) yielding complete ORFs by gene segment**							59	46	42	49	58	61	61		

[a]All samples were provided as clinical specimens with rt-RTPCR Ct values <30 (NPA = Nasal/Pharyngeal Aspirate, NPS = Nasal/Pharyngeal Swab, NS = Nasal Swab, TS = Throat Swab). [b]Age given in years. [c]Sex, M = Male, F = Female. [d]CAP = Community acquired pneumonia; COPD = Chronic Obstructive Pulmonary Disease; ILI = Influenza Like Illness; GE = Gastroenteritis; SOB = Shortness of breath; SVT = Supraventricular tachycardia; URTI = Upper respiratory tract infection. [e]AH1 = Influenza A(H1N1)pdm09; AH3 = Influenza A(H3N2); AV = Adenovirus; PIV = Parainfluenza virus (types 1, 2, 3, 4); PV = Picornavirus; RSV = Respiratory syncytial virus; ND = None Detected (of those that were screened for). [f]For each sample the recovery of at least one gene sequence encompassing complete open reading frame(s) is indicated (Y). [g]Corresponding EpiFlu database (GISAID) isolate accession numbers are given. [h]Periods by week range in 2019 and 2020 are given. No sequence recovered. Full genomes recovered.

Table S2. Minority variants within genes of ICVs from Hong Kong collected in 2019-20

Virus name[a]	Clade[b]	Gene/ORF[c]	Nucleotide number[d]	Total Reads	Concensus nucleotide[e]	Alternate nucleotide[e]	Supporting Reads	Frequency of alternate (%)	Amino acid position[f]	Codon position[g]	S/NS[h]	Amino acid substitution[i]	Double nucleotide substitution[j]
C/Hong/Kong/129532/2019	S1	HE/HEF2	1870	12342	C	T	141	1.14	610	1	NS	P-S	
C/Hong/Kong/129532/2019	S1	PB1	2096	1152	C	G	14	1.21	699	2	NS	A-G	
C/Hong/Kong/129532/2019	S1	NP	600	14056	A	G	395	2.81	200	3	S	L	
C/Hong/Kong/129532/2019	S1	CM/CM1	28	4189	A	G	44	1.05	10	1	NS	T-A	
C/Hong/Kong/129532/2019	S1	CM/CM1	32	4226	A	G	43	1.02	11	2	NS	E-G	
C/Hong/Kong/132137/2019	S1	PB2	1729	5647	A	G	60	1.06	577	1	NS	T-A	
C/Hong/Kong/132137/2019	S1	PB1	1843	1166	G	GA	12	1.03	615	1ins		fs	
C/Hong/Kong/132137/2019	S1	PB1	2092	968	T	G	16	1.65	698	1	NS	S-A	
C/Hong/Kong/132137/2019	S1	PB1	2096	694	C	G	12	1.72	699	2	NS	A-G	
C/Hong/Kong/133905/2019	S1	PB2	1729	3114	A	G	77	2.47	577	1	NS	T-A	
C/Hong/Kong/133905/2019	S1	PB2	1732	3698	G	A	58	1.57	578	1	NS	G-R	K
C/Hong/Kong/133905/2019	S1	PB2	1733	2959	G	A	102	3.45	578	2	NS	G-E	
C/Hong/Kong/133905/2019	S1	PB1	2092	1029	T	G	18	1.75	698	1	NS	S-A	
C/Hong/Kong/133905/2019	S1	PB1	2096	778	C	G	23	2.93	699	2	NS	A-G	
C/Hong/Kong/133905/2019	S1	P3	1271	2497	G	A	88	3.52	424	2	NS	G-E	
C/Hong/Kong/133905/2019	S1	NP	626	13361	G	A	2360	17.66	209	2	NS	R-K	
C/Hong/Kong/156019/2019	S1	HE/HEF1	373	148	GT	G	14	9.46	111	1del		fs	
C/Hong/Kong/156019/2019	S1	HE/HEF1	594	212	G	A	13	6.13	184	3	S	L	
C/Hong/Kong/156019/2019	S1	HE/HEF1	1227	252	AT	A	10	3.97	395	1del		fs	
C/Hong/Kong/156019/2019	S1	HE/HEF2	1399	530	A	G	84	15.85	453	1	NS	I-V	
C/Hong/Kong/156019/2019	S1	HE/HEF2	1480	697	A	G	11	1.58	480	1	NS	I-V	
C/Hong/Kong/156019/2019	S1	HE/HEF2	1719	747	A	G	13	1.74	559	3	S	G	
C/Hong/Kong/156019/2019	S1	PB2	1119	59	A	G	11	18.64	373	3	S	E	
C/Hong/Kong/156019/2019	S1	CM/CM1	303	199	T	C	13	6.53	101	3	S	A	
C/Hong/Kong/177040/2019	S1	HE/HEF1	1323	20004	C	T	412	2.06	427	3	S	T	
C/Hong/Kong/177040/2019	S1	PB2	1729	9759	A	G	106	1.09	577	1	NS	T-A	
C/Hong/Kong/177040/2019	S1	PB2	1733	6340	G	A	181	2.85	578	2	NS	G-E	
C/Hong/Kong/177040/2019	S1	P3	1271	3860	G	A	189	4.90	424	2	NS	G-E	
C/Hong/Kong/177040/2019	S1	CM/CM1	28	3953	A	G	42	1.06	10	1	NS	T-A	
C/Hong/Kong/179364/2019	S2	HE/HEF1	578	4392	T	TA	49	1.12	179	1ins		fs	
C/Hong/Kong/179364/2019	S2	HE/HEF1	852	3015	C	T	33	1.09	270	3	S	T	
C/Hong/Kong/179364/2019	S2	HE/HEF2	1629	3790	G	A	52	1.37	529	3	S	G	
C/Hong/Kong/179364/2019	S2	HE/HEF2	1728	2523	G	A	33	1.31	562	3	S	A	
C/Hong/Kong/179364/2019	S2	HE/HEF2	1893	1836	G	A	20	1.09	617	3	S	L	
C/Hong/Kong/179364/2019	S2	PB2	862	600	C	A	10	1.67	288	1	NS	L-M	
C/Hong/Kong/179364/2019	S2	PB2	1617	979	C	T	18	1.84	539	3	S	D	
C/Hong/Kong/179364/2019	S2	PB2	1729	1026	A	G	11	1.07	577	1	NS	T-A	
C/Hong/Kong/179364/2019	S2	PB2	1794	814	G	A	10	1.23	598	3	S	P	
C/Hong/Kong/179364/2019	S2	PB2	1800	1310	A	G	26	1.98	600	3	S	V	
C/Hong/Kong/179364/2019	S2	PB2	1806	1367	G	A	14	1.02	602	3	S	L-M	
C/Hong/Kong/179364/2019	S2	PB2	1815	1370	T	C	14	1.02	605	3	S	P	
C/Hong/Kong/179364/2019	S2	PB2	1924	1081	C	T	61	5.64	642	1	NS	P-S	

Virus name[a]	Clade[b]	Gene/ORF[c]	Nucleotide number[d]	Total Reads	Concensus nucleotide[e]	Alternate nucleotide[e]	Supporting Reads	Frequency of alternate (%)	Amino acid position[f]	Codon position[g]	S/NS[h]	Amino acid substitution[i]	Double nucleotide substitution[j]
C/Hong/Kong/179364/2019	S2	P3	73	458	G	A	10	2.18	25	1	NS	V-I	
C/Hong/Kong/179364/2019	S2	P3	159	503	G	A	11	2.19	53	3	S	L	
C/Hong/Kong/179364/2019	S2	P3	160	509	A	G	10	1.96	54	1	NS	S-G	
C/Hong/Kong/179364/2019	S2	P3	177	499	C	T	11	2.20	59	3	S	D	
C/Hong/Kong/179364/2019	S2	P3	441	382	G	A	17	4.45	147	3	S	E	
C/Hong/Kong/179364/2019	S2	P3	912	445	C	T	15	3.37	304	3	S	P	
C/Hong/Kong/179364/2019	S2	P3	975	328	C	T	11	3.35	325	3	S	A	
C/Hong/Kong/179364/2019	S2	P3	1014	603	A	G	11	1.82	338	3	S	E	
C/Hong/Kong/179364/2019	S2	P3	1271	492	G	A	13	2.64	424	2	NS	G-E	
C/Hong/Kong/179364/2019	S2	P3	2047	666	A	G	17	2.55	683	1	NS	M-V	
C/Hong/Kong/179364/2019	S2	P3	2052	421	C	T	20	4.75	684	3	S	C	
C/Hong/Kong/179364/2019	S2	P3	2080	636	C	T	11	1.73	694	1	NS	L-F	
C/Hong/Kong/179364/2019	S2	NP	235	1519	G	A	24	1.58	79	1	NS	D-N	
C/Hong/Kong/179364/2019	S2	NP	741	3239	A	G	51	1.57	247	3	S	R	
C/Hong/Kong/179364/2019	S2	NP	1029	3080	A	G	31	1.01	343	3	S	V	
C/Hong/Kong/179364/2019	S2	CM/CM1	28	824	A	G	15	1.82	10	1	NS	T-A	
C/Hong/Kong/179364/2019	S2	CM/CM1	85	897	A	G	12	1.34	29	1	NS	I-V	E
C/Hong/Kong/179364/2019	S2	CM/CM1	86	879	T	A	11	1.25	29	2	NS	I-K	
C/Hong/Kong/179364/2019	S2	CM/CM1	88	908	A	G	15	1.65	30	1	NS	T-A	
C/Hong/Kong/179364/2019	S2	CM/CM1	93	813	T	G	11	1.35	31	3	S	G	
C/Hong/Kong/179364/2019	S2	CM/CM1	138	970	G	A	11	1.13	46	3	S	E	
C/Hong/Kong/179364/2019	S2	CM/CM1	165	740	C	A	10	1.35	55	3	S	A	
C/Hong/Kong/179364/2019	S2	CM/CM1	210	1036	G	A	13	1.25	70	3	S	K	
C/Hong/Kong/179364/2019	S2	CM/CM1	282	1040	T	C	12	1.15	94	3	S	I	
C/Hong/Kong/179364/2019	S2	CM/CM1	287	818	G	A	13	1.59	96	2	NS	R-K	
C/Hong/Kong/179364/2019	S2	CM/CM1	384	1184	C	A	12	1.01	128	3	S	V	
C/Hong/Kong/179364/2019	S2	CM/CM1	478	1296	A	G	13	1.00	160	1	NS	S-G	
C/Hong/Kong/179364/2019	S2	CM/CM1	690	982	G	A	11	1.12	230	3	S	E	
C/Hong/Kong/179364/2019	S2	CM/CM2	773	1131	C	T	24	2.12	23	2	NS	S-F	
C/Hong/Kong/179364/2019	S2	CM/CM2	817	912	A	G	10	1.10	38	1	NS	N-D	
C/Hong/Kong/179364/2019	S2	CM/CM2	837	905	C	T	10	1.10	44	3	S	C	
C/Hong/Kong/179364/2019	S2	NS/NS1	456	769	C	T	11	1.43	152	3	S	N	
C/Hong/Kong/179364/2019	S2	NS/NS1	468	965	A	G	14	1.45	156	3	NS	I-M	
C/Hong/Kong/179364/2019	S2	NS/NS1	540	950	A	G	11	1.16	180	3	S	Q	
C/Hong/Kong/179364/2019	S2	NS/NS2	540	950	A	G	11	1.16	76	2	NS	K-R	
C/Hong/Kong/183718/2019	S1	HE/HEF2	1867	7180	G	T	957	13.33	609	1	NS	D-Y	
C/Hong/Kong/183718/2019	S1	NP	1184	5818	C	T	91	1.56	395	2	NS	T-I	
C/Hong/Kong/195607/2019	S1	HE/HEF2	1437	4169	A	G	44	1.06	465	3	S	G	
C/Hong/Kong/195607/2019	S1	PB2	540	860	T	C	17	1.98	180	3	S	P	
C/Hong/Kong/195607/2019	S1	PB2	763	817	G	A	11	1.35	255	1	NS	G-R	K
C/Hong/Kong/195607/2019	S1	PB2	764	797	G	A	14	1.76	255	2	NS	G-E	
C/Hong/Kong/195607/2019	S1	PB2	1118	937	A	G	15	1.60	373	2	NS	E-G	
C/Hong/Kong/195607/2019	S1	PB2	1119	959	A	G	30	3.13	373	3	S	E	
C/Hong/Kong/195607/2019	S1	PB1	2043	679	A	G	10	1.47	681	3	S	E	

Virus name[a]	Clade[b]	Gene/ORF[c]	Nucleotide number[d]	Total Reads	Concensus nucleotide[e]	Alternate nucleotide[e]	Supporting Reads	Frequency of alternate (%)	Amino acid position[f]	Codon position[g]	S/NS[h]	Amino acid substitution[i]	Double nucleotide substitution[j]
C/Hong/Kong/195607/2019	S1	PB1	2096	555	C	G	41	7.31	699	2	NS	A-G	
C/Hong/Kong/195607/2019	S1	P3	1271	767	G	A	39	5.08	424	2	NS	G-E	
C/Hong/Kong/195607/2019	S1	NP	1274	2653	T	G	474	17.87	425	2	NS	V-G	
C/Hong/Kong/195607/2019	S1	CM/CM1	32	789	A	G	13	1.65	11	2	NS	E-G	
C/Hong/Kong/195607/2019	S1	CM/CM2	952	1028	G	A	11	1.07	83	1	NS	G-S	
C/Hong/Kong/195607/2019	S1	CM/CM2	959	1033	G	GCA	11	1.06	85	2ins		fs	
C/Hong/Kong/195607/2019	S1	NS/NS1	224	981	A	G	33	3.36	75	2	NS	E-G	
C/Hong/Kong/234288/2019	S1	HE/HEF1	246	4745	G	A	61	1.29	68	3	S	R	
C/Hong/Kong/234288/2019	S1	HE/HEF1	616	7524	C	T	860	11.43	192	1	NS	P-S	
C/Hong/Kong/234288/2019	S1	PB2	1018	1088	A	T	21	1.93	340	1	NS	M-L	
C/Hong/Kong/234288/2019	S1	PB2	1116	1235	A	G	13	1.05	372	3	S	Q	
C/Hong/Kong/234288/2019	S1	PB2	1729	5313	A	G	75	1.41	577	1	NS	T-A	
C/Hong/Kong/234288/2019	S1	PB2	1733	4577	G	A	104	2.27	578	2	NS	G-E	
C/Hong/Kong/234288/2019	S1	PB1	2096	431	C	G	74	16.89	699	2	NS	A-G	
C/Hong/Kong/234288/2019	S1	P3	1271	1303	G	A	63	4.83	424	2	NS	G-E	
C/Hong/Kong/234288/2019	S1	CM/CM1	16	2965	T	G	158	5.33	6	1	NS	L-V	
C/Hong/Kong/234288/2019	S1	CM/CM1	21	2890	C	CA	278	9.52	7	1ins		fs	
C/Hong/Kong/234288/2019	S1	CM/CM1	24	3159	T	A	310	9.81	8	3	S	A	
C/Hong/Kong/234288/2019	S1	CM/CM1	32	3533	A	G	397	11.22	11	2	NS	E-G	
C/Hong/Kong/234288/2019	S1	CM/CM1	85	5028	A	G	69	1.37	29	1	NS	I-V	E
C/Hong/Kong/234288/2019	S1	CM/CM1	86	4993	T	A	74	1.48	29	2	NS	I-K	
C/Hong/Kong/234288/2019	S1	CM/CM1	88	5032	A	G	91	1.81	30	1	NS	T-A	
C/Hong/Kong/234288/2019	S1	CM/CM1	93	4791	T	G	54	1.12	31	3	S	G	
C/Hong/Kong/234288/2019	S1	NS/NS1	679	3741	G	A	309	8.26	227	1	NS	E-K	
C/Hong/Kong/234288/2019	S1	NS/NS2	679	3741	G	A	309	8.26	122	3	NS	M-I	
C/Hong/Kong/234519/2019	S1	HE/HEF2	1399	1048	A	G	54	5.15	453	1	NS	I-V	
C/Hong/Kong/234519/2019	S1	HE/HEF2	1442	1111	G	T	64	5.76	467	2	NS	G-V	
C/Hong/Kong/234519/2019	S1	PB2	1733	982	G	A	86	8.76	578	2	NS	G-E	
C/Hong/Kong/235164/2019	S1	PB2	679	2817	G	A	36	1.28	227	1	NS	V-I	
C/Hong/Kong/235164/2019	S1	PB2	1729	3534	A	G	38	1.07	577	1	NS	T-A	
C/Hong/Kong/235164/2019	S1	PB2	1733	3088	G	A	136	4.40	578	2	NS	G-E	
C/Hong/Kong/235164/2019	S1	PB2	2205	2567	G	A	28	1.09	735	3	S	G	
C/Hong/Kong/235164/2019	S1	PB1	2096	1024	C	G	13	1.27	699	2	NS	A-G	
C/Hong/Kong/235164/2019	S1	P3	1271	3776	G	A	93	2.46	424	2	NS	G-E	
C/Hong/Kong/235164/2019	S1	NP	518	5746	G	A	110	1.91	173	2	NS	G-E	
C/Hong/Kong/235164/2019	S1	CM/CM1	28	2234	A	G	54	2.42	10	1	NS	T-A	
C/Hong/Kong/235164/2019	S1	CM/CM1	32	2240	A	G	37	1.65	11	2	NS	E-G	
C/Hong/Kong/235164/2019	S1	NS/NS1	21	1416	A	G	35	2.47	7	3	S	K	
C/Hong/Kong/235164/2019	S1	NS/NS1	121	1785	A	G	39	2.18	41	1	NS	T-A	
C/Hong/Kong/235164/2019	S1	NS/NS1	124	1808	A	G	30	1.66	42	1	NS	K-E	
C/Hong/Kong/235164/2019	S1	NS/NS1	129	1778	T	A	45	2.53	43	3	S	A	
C/Hong/Kong/235164/2019	S1	NS/NS1	130	1780	A	G	42	2.36	44	1	NS	R-G	
C/Hong/Kong/235164/2019	S1	NS/NS1	532	1747	G	A	18	1.03	178	1	NS	A-T	
C/Hong/Kong/235164/2019	S1	NS/NS2	532	1747	G	A	18	1.03	73	3	S	S	

Virus name[a]	Clade[b]	Gene/ORF[c]	Nucleotide number[d]	Total Reads	Concensus nucleotide[e]	Alternate nucleotide[e]	Supporting Reads	Frequency of alternate (%)	Amino acid position[f]	Codon position[g]	S/NS[h]	Amino acid substitution[i]	Double nucleotide substitution[j]
C/Hong/Kong/235298/2019	K	PB2	951	5236	A	T	160	3.06	317	3	S	S	
C/Hong/Kong/235298/2019	K	PB1	69	1143	CTA	C	12	1.05	23-24	2del		fs	
C/Hong/Kong/235298/2019	K	PB1	1203	1502	C	T	19	1.26	401	3	S	V	
C/Hong/Kong/235298/2019	K	PB1	1205	1500	G	GT	16	1.06	402	1ins		fs	
C/Hong/Kong/235298/2019	K	P3	404	4673	A	C	86	1.84	135	2	NS	D-G	
C/Hong/Kong/235298/2019	K	P3	406	4578	G	A	87	1.90	136	1	NS	G-R	
C/Hong/Kong/235298/2019	K	NP	270	10175	A	G	651	6.40	90	3	S	R	
C/Hong/Kong/235298/2019	K	NP	1655	5836	A	G	207	3.55	552	2	NS	D-G	
C/Hong/Kong/235298/2019	K	CM/CM1	28	3903	A	G	96	2.46	10	1	NS	T-A	
C/Hong/Kong/235298/2019	K	CM/CM1	32	3954	A	G	79	2.00	11	2	NS	E-G	
C/Hong/Kong/237362/2019	S1	PB2	1729	1198	A	G	16	1.34	577	1	NS	T-A	
C/Hong/Kong/237362/2019	S1	PB2	1733	1255	G	A	42	3.34	578	2	NS	G-E	
C/Hong/Kong/237362/2019	S1	PB1	1509	409	G	A	11	2.69	503	3	S	V	
C/Hong/Kong/237362/2019	S1	P3	1271	909	G	A	12	1.32	424	2	NS	G-E	
C/Hong/Kong/237362/2019	S1	NP	1565	2949	C	A	32	1.08	522	2	NS	A-E	
C/Hong/Kong/237362/2019	S1	CM/CM1	28	942	A	G	36	3.82	10	1	NS	T-A	
C/Hong/Kong/237362/2019	S1	CM/CM1	32	952	A	G	30	3.15	11	2	NS	E-G	
C/Hong/Kong/237362/2019	S1	CM/CM1	478	1765	A	G	32	1.81	160	1	NS	S-G	
C/Hong/Kong/237362/2019	S1	NS/NS1	121	676	A	G	11	1.63	41	1	NS	T-A	
C/Hong/Kong/237362/2019	S1	NS/NS1	129	677	T	A	11	1.62	43	3	S	A	
C/Hong/Kong/237362/2019	S1	NS/NS1	130	674	A	G	10	1.48	44	1	NS	R-G	
C/Hong/Kong/238624/2019	S2	HE/HEF2	1398	775	A	G	27	3.48	452	3	S	G	
C/Hong/Kong/238624/2019	S2	HE/HEF2	1399	831	A	G	18	2.17	453	1	NS	I-V	
C/Hong/Kong/238624/2019	S2	P3	1271	61	G	A	11	18.03	424	2	NS	G-E	
C/Hong/Kong/242697/2019	S2	HE/HEF2	1398	1468	A	G	56	3.81	452	3	S	G	
C/Hong/Kong/242697/2019	S2	HE/HEF2	1399	1480	A	G	119	8.04	453	1	NS	I-V	
C/Hong/Kong/243709/2019	S1	HE/HEF2	1388	5747	T	C	67	1.17	449	2	NS	V-A	
C/Hong/Kong/243709/2019	S1	HE/HEF2	1389	5749	T	A	86	1.50	449	3	S	V	
C/Hong/Kong/243709/2019	S1	PB2	1727	5502	G	A	66	1.20	576	2	NS	G-E	
C/Hong/Kong/243709/2019	S1	PB2	1729	5536	A	G	171	3.09	577	1	NS	T-A	
C/Hong/Kong/243709/2019	S1	PB1	2096	407	C	G	71	17.44	699	2	NS	A-G	
C/Hong/Kong/243709/2019	S1	NS/NS1	224	3388	A	G	53	1.56	75	2	NS	E-G	
C/Hong/Kong/243520/2019	S1	HE/HEF1	654	3584	C	T	71	1.98	204	3	S	F	
C/Hong/Kong/243520/2019	S1	PB2	1729	1790	A	G	90	5.03	577	1	NS	T-A	
C/Hong/Kong/243520/2019	S1	PB2	1732	3260	G	A	56	1.72	578	1	NS	G-R	
C/Hong/Kong/243520/2019	S1	CM/CM1	486	3810	C	T	100	2.62	162	3	S	V	
C/Hong/Kong/243270/2019	S1	CM/CM1	24	1084	T	A	56	5.17	8	3	S	A	
C/Hong/Kong/243270/2019	S1	CM/CM1	28	1222	A	G	182	14.89	10	1	NS	T-A	
C/Hong/Kong/243270/2019	S1	CM/CM1	32	1282	A	G	157	12.25	11	2	NS	E-G	
C/Hong/Kong/244337/2019	S1	HE/HEF2	1428	4406	A	G	61	1.38	462	3	S	K	
C/Hong/Kong/244337/2019	S1	PB2	1729	1066	A	G	17	1.59	577	1	NS	T-A	
C/Hong/Kong/244337/2019	S1	PB2	1732	1247	G	A	13	1.04	578	1	NS	G-R	K
C/Hong/Kong/244337/2019	S1	PB2	1733	1248	G	A	15	1.20	578	2	NS	G-E	
C/Hong/Kong/244337/2019	S1	PB2	1886	1417	C	T	35	2.47	629	2	NS	T-I	

Virus name[a]	Clade[b]	Gene/ORF[c]	Nucleotide number[d]	Total Reads	Concensus nucleotide[e]	Alternate nucleotide[e]	Supporting Reads	Frequency of alternate (%)	Amino acid position[f]	Codon position[g]	S/NS[h]	Amino acid substitution[i]	Double nucleotide substitution[j]
C/Hong/Kong/244337/2019	S1	P3	18	178	C	T	23	12.85	6	3	S	A	
C/Hong/Kong/244337/2019	S1	CM/CM1	85	740	A	G	16	2.16	29	1	NS	I-V	E
C/Hong/Kong/244337/2019	S1	CM/CM1	86	747	T	A	16	2.14	29	2	NS	I-K	
C/Hong/Kong/244337/2019	S1	CM/CM1	88	745	A	G	20	2.68	30	1	NS	T-A	
C/Hong/Kong/244337/2019	S1	CM/CM1	93	716	T	G	16	2.23	31	3	S	G	
C/Hong/Kong/244337/2019	S1	CM/CM2	773	830	C	T	15	1.81	23	2	NS	S-F	
C/Hong/Kong/244337/2019	S1	NS/NS1	133	455	C	T	10	2.20	45	1	S	L	
C/Hong/Kong/245344/2019	S1	HE/HEF1	485	6272	A	G	65	1.04	148	2	NS	K-R	
C/Hong/Kong/245344/2019	S1	HE/HEF1	540	6335	G	T	64	1.01	166	3	S	A	
C/Hong/Kong/245344/2019	S1	HE/HEF1	554	6493	C	CA	76	1.17	171	1ins		fs	
C/Hong/Kong/245344/2019	S1	HE/HEF1	1202	6613	C	T	72	1.09	387	2	NS	A-V	
C/Hong/Kong/245344/2019	S1	HE/HEF1	1308	6382	T	C	70	1.10	422	3	S	T	
C/Hong/Kong/245344/2019	S1	HE/HEF1	1317	6340	T	C	72	1.14	425	3	S	T	
C/Hong/Kong/245344/2019	S1	HE/HEF1	1333	6394	A	G	146	2.28	431	1	NS	S-G	
C/Hong/Kong/245344/2019	S1	HE/HEF2	1342	6568	T	C	79	1.20	434	1	NS	F-L	P
C/Hong/Kong/245344/2019	S1	HE/HEF2	1343	6581	T	C	80	1.22	434	2	NS	F-S	
C/Hong/Kong/245344/2019	S1	HE/HEF2	1375	6339	T	C	87	1.37	445	1	NS	F-L	P
C/Hong/Kong/245344/2019	S1	HE/HEF2	1376	6354	T	C	87	1.37	445	2	NS	F-S	
C/Hong/Kong/245344/2019	S1	HE/HEF2	1377	6334	T	C	86	1.36	445	3	S	F	
C/Hong/Kong/245344/2019	S1	HE/HEF2	1379	6302	T	C	88	1.40	446	2	NS	V-A	
C/Hong/Kong/245344/2019	S1	HE/HEF2	1666	10170	G	A	196	1.93	542	1	NS	G-R	
C/Hong/Kong/245344/2019	S1	PB2	1729	2191	A	G	75	3.42	577	1	NS	T-A	
C/Hong/Kong/245344/2019	S1	PB2	2218	2960	C	T	153	5.17	740	1	NS	L-F	
C/Hong/Kong/245344/2019	S1	NP	444	5300	A	G	119	2.25	148	3	S	E	
C/Hong/Kong/245344/2019	S1	NP	1101	6798	T	C	94	1.38	367	3	S	L	
C/Hong/Kong/245344/2019	S1	NP	1231	6542	T	C	103	1.57	411	1	NS	S-P	
C/Hong/Kong/245344/2019	S1	NP	1354	6809	C	T	93	1.37	452	1	NS	*	
C/Hong/Kong/245344/2019	S1	NP	1375	6636	A	G	103	1.55	459	1	NS	R-G	
C/Hong/Kong/245344/2019	S1	CM/CM1	28	2576	A	G	161	6.25	10	1	NS	T-A	
C/Hong/Kong/245344/2019	S1	CM/CM1	32	2608	A	G	103	3.95	11	2	NS	E-G	
C/Hong/Kong/245344/2019	S1	CM/CM1	85	3258	A	G	57	1.75	29	1	NS	I-V	
C/Hong/Kong/245344/2019	S1	CM/CM1	88	3263	A	G	65	1.99	30	1	NS	T-A	
C/Hong/Kong/245344/2019	S1	CM/CM2	774	5722	A	T	195	3.41	23	3	S	S	
C/Hong/Kong/245344/2019	S1	CM/CM2	1040	2954	A	T	83	2.81	112	2	NS	E-V	
C/Hong/Kong/245344/2019	S1	NS/NS1	524	1151	A	G	191	16.59	175	2	NS	Y-C	
C/Hong/Kong/245344/2019	S1	NS/NS2	524	1151	A	G	191	16.59	71	1	NS	T-A	
C/Hong/Kong/248593/2019	S2	PB2	1729	7384	A	G	83	1.12	577	1	NS	T-A	
C/Hong/Kong/248593/2019	S2	PB1	2092	813	T	G	128	15.74	698	1	NS	S-A	
C/Hong/Kong/248593/2019	S2	PB1	2096	853	C	G	161	18.87	699	2	NS	A-G	
C/Hong/Kong/248593/2019	S2	CM/CM2	773	4075	C	T	73	1.79	23	2	NS	S-F	
C/Hong/Kong/248430/2019	K	HE/HEF2	1500	14536	C	T	214	1.47	486	3	S	I	
C/Hong/Kong/248430/2019	K	PB2	792	3153	C	A	77	2.44	264	3	NS	N-K	
C/Hong/Kong/248430/2019	K	PB1	2096	715	C	G	12	1.68	699	2	NS	A-G	
C/Hong/Kong/248430/2019	K	P3	29	1957	A	G	22	1.12	10	2	NS	E-G	

Virus name[a]	Clade[b]	Gene/ORF[c]	Nucleotide number[d]	Total Reads	Concensus nucleotide[e]	Alternate nucleotide[e]	Supporting Reads	Frequency of alternate (%)	Amino acid position[f]	Codon position[g]	S/NS[h]	Amino acid substitution[i]	Double nucleotide substitution[j]
C/Hong/Kong/248430/2019	K	P3	636	3697	T	C	83	2.25	212	3	S	Y	
C/Hong/Kong/248430/2019	K	P3	1271	3332	G	A	41	1.23	424	2	NS	G-E	
C/Hong/Kong/248430/2019	K	NP	468	9919	A	G	1066	10.74	156	3	S	R	
C/Hong/Kong/248430/2019	K	CM/CM1	28	3642	A	G	68	1.87	10	1	NS	T-A	
C/Hong/Kong/248430/2019	K	CM/CM1	32	3669	A	G	55	1.50	11	2	NS	E-G	
C/Hong/Kong/251408/2019	S1	HE/HEF1	1207	4610	G	A	131	2.84	389	1	NS	E-K	
C/Hong/Kong/251408/2019	S1	PB2	822	704	T	C	14	1.99	274	3	S	C	
C/Hong/Kong/251408/2019	S1	PB2	1118	731	A	G	11	1.50	373	2	NS	E-G	
C/Hong/Kong/251408/2019	S1	PB2	1119	730	A	G	15	2.05	373	3	S	E	
C/Hong/Kong/251408/2019	S1	PB2	1729	1586	A	G	19	1.20	577	1	NS	T-A	
C/Hong/Kong/251408/2019	S1	PB2	1938	1674	C	T	20	1.19	646	3	S	F	
C/Hong/Kong/251408/2019	S1	PB2	2115	1686	G	A	29	1.72	705	3	S	S	
C/Hong/Kong/251408/2019	S1	P3	1271	721	G	A	12	1.66	424	2	NS	G-E	
C/Hong/Kong/251408/2019	S1	P3	1581	857	T	A	10	1.17	527	3	S	A	
C/Hong/Kong/251408/2019	S1	NP	733	1842	C	G	26	1.41	245	1	NS	L-V	
C/Hong/Kong/251408/2019	S1	NP	742	1866	A	G	20	1.07	248	1	NS	R-G	
C/Hong/Kong/251408/2019	S1	CM/CM1	28	1071	A	G	16	1.49	10	1	NS	T-A	
C/Hong/Kong/251408/2019	S1	CM/CM1	32	1075	A	G	15	1.40	11	2	NS	E-G	
C/Hong/Kong/251408/2019	S1	CM/CM1	413	1808	A	G	20	1.11	138	2	NS	D-G	
C/Hong/Kong/251408/2019	S1	CM/CM1	478	1885	A	G	36	1.91	160	1	NS	S-G	
C/Hong/Kong/251408/2019	S1	CM/CM1	630	1942	TC	T	20	1.03	210	1del		fs	
C/Hong/Kong/251408/2019	S1	CM/CM2	1095	1140	T	C	13	1.14	130	3	S	P	
C/Hong/Kong/251408/2019	S1	CM/CM2	1118	1111	A	G	12	1.08	138	2	NS	E-G	
C/Hong/Kong/251408/2019	S1	NS/NS1	320	704	C	T	12	1.70	107	2	NS	P-L	
C/Hong/Kong/251408/2019	S1	NS/NS1	610	545	G	A	12	2.20	204	1	NS	D-N	
C/Hong/Kong/251408/2019	S1	NS/NS2	610	545	G	A	12	2.20	99	3	S	P	
C/Hong/Kong/251408/2019	S1	NS/NS1	647	804	C	T	10	1.24	216	2	NS	S-L	
C/Hong/Kong/251408/2019	S1	NS/NS2	647	804	C	T	10	1.24	112	1	NS	*	
C/Hong/Kong/251160/2019	S1	HE/HEF2	1948	4467	G	A	723	16.19	636	1	NS	A-T	
C/Hong/Kong/251160/2019	S1	PB2	1729	2380	A	G	26	1.09	577	1	NS	T-A	
C/Hong/Kong/251160/2019	S1	NP	1138	7432	G	A	1169	15.73	380	1	NS	V-I	
C/Hong/Kong/251160/2019	S1	CM/CM1	28	1887	A	G	46	2.44	10	1	NS	T-A	
C/Hong/Kong/251160/2019	S1	CM/CM1	32	1884	A	G	41	2.18	11	2	NS	E-G	
C/Hong/Kong/251160/2019	S1	NS/NS1	121	1126	A	G	17	1.51	41	1	NS	T-A	
C/Hong/Kong/251160/2019	S1	NS/NS1	124	1143	A	G	15	1.31	42	1	NS	K-E	
C/Hong/Kong/251160/2019	S1	NS/NS1	129	1094	T	A	18	1.64	43	3	S	A	
C/Hong/Kong/251160/2019	S1	NS/NS1	130	1111	A	G	16	1.44	44	1	NS	R-G	
C/Hong/Kong/251816/2019	S2	HE/HEF1	1077	15151	G	A	191	1.26	345	3	S	K	
C/Hong/Kong/251816/2019	S2	PB2	542	7372	T	C	84	1.14	181	2	NS	L-P	
C/Hong/Kong/251816/2019	S2	PB2	1016	4418	C	T	157	3.55	339	2	NS	P-L	
C/Hong/Kong/251816/2019	S2	PB2	1018	6371	A	T	489	7.46	340	1	NS	M-L	
C/Hong/Kong/251816/2019	S2	PB2	1733	6342	G	A	203	3.20	578	2	NS	G-E	
C/Hong/Kong/251816/2019	S2	PB1	1843	2045	G	GA	29	1.42	615	1ins		fs	
C/Hong/Kong/251816/2019	S2	PB1	2096	1400	C	G	60	4.20	699	2	NS	A-G	

Virus name[a]	Clade[b]	Gene/ORF[c]	Nucleotide number[d]	Total Reads	Concensus nucleotide[e]	Alternate nucleotide[e]	Supporting Reads	Frequency of alternate (%)	Amino acid position[f]	Codon position[g]	S/NS[h]	Amino acid substitution[i]	Double nucleotide substitution[j]
C/Hong/Kong/251816/2019	S2	P3	174	3882	T	C	198	5.10	58	3	S	N	
C/Hong/Kong/251816/2019	S2	P3	1271	4739	G	A	177	3.73	424	2	NS	G-E	
C/Hong/Kong/251816/2019	S2	NP	518	9294	G	A	121	1.30	173	2	NS	G-E	
C/Hong/Kong/253739/2019	S1	PB2	1729	7113	A	G	79	1.11	577	1	NS	T-A	
C/Hong/Kong/253739/2019	S1	NS/NS1	121	2414	A	G	71	2.94	41	1	NS	T-A	
C/Hong/Kong/253739/2019	S1	NS/NS1	129	2439	T	A	69	2.83	43	3	S	A	
C/Hong/Kong/253739/2019	S1	NS/NS1	130	2455	A	G	68	2.77	44	1	NS	R-G	
C/Hong/Kong/256484/2019	S1	PB1	216	1230	TG	T	14	1.14	72-73	1del		fs	
C/Hong/Kong/256484/2019	S1	PB1	2063	1254	GGTTTGCGACAT	G	24	1.91	688-690	11del		fs	
C/Hong/Kong/256484/2019	S1	P3	29	1671	A	G	22	1.32	10	2	NS	E-G	
C/Hong/Kong/256484/2019	S1	P3	174	2123	TGACA	T	27	1.27	58-60	4del		fs	
C/Hong/Kong/256484/2019	S1	P3	474	2642	A	T	37	1.40	158	3	S	A	
C/Hong/Kong/258168/2019	?	NS/NS1	556	205	A	G	34	16.59	186	1	NS	T-A	
C/Hong/Kong/258168/2019	?	NS/NS2	556	205	A	G	34	16.59	81	3	S	K	
C/Hong/Kong/258168/2019	?	NS/NS1	646	226	T	G	10	4.42	216	1	NS	S-A	
C/Hong/Kong/258168/2019	?	NS/NS2	646	226	T	G	10	4.42	111	3	NS	N-K	
C/Hong/Kong/261118/2019	S1	PB2	763	3362	G	A	34	1.01	255	1	NS	G-R	
C/Hong/Kong/261118/2019	S1	PB2	1729	10169	A	G	233	2.29	577	1	NS	T-A	
C/Hong/Kong/261118/2019	S1	PB2	1732	12995	G	A	130	1.00	578	1	NS	G-R	K
C/Hong/Kong/261118/2019	S1	PB2	1733	13005	G	A	137	1.05	578	2	NS	G-E	
C/Hong/Kong/261118/2019	S1	PB1	2096	886	C	G	33	3.71	699	2	NS	A-G	
C/Hong/Kong/261118/2019	S1	NS/NS1	224	3311	A	G	80	2.42	75	2	NS	E-G	
C/Hong/Kong/262662/2019	S1	PB2	1729	6347	A	G	68	1.07	577	1	NS	T-A	
C/Hong/Kong/262662/2019	S1	PB2	1733	5199	G	A	219	4.21	578	2	NS	G-E	
C/Hong/Kong/262662/2019	S1	PB1	2092	2210	T	G	30	1.36	698	1	NS	S-A	
C/Hong/Kong/262662/2019	S1	PB1	2096	1565	C	G	22	1.40	699	2	NS	A-G	
C/Hong/Kong/262662/2019	S1	P3	1271	5548	G	A	145	2.61	424	2	NS	G-E	
C/Hong/Kong/262662/2019	S1	NP	518	8199	G	A	138	1.68	173	2	NS	G-E	
C/Hong/Kong/262662/2019	S1	CM/CM1	28	3129	A	G	44	1.41	10	1	NS	T-A	
C/Hong/Kong/262662/2019	S1	NS/NS1	121	3047	A	G	58	1.90	41	1	NS	T-A	
C/Hong/Kong/262662/2019	S1	NS/NS1	124	3118	A	G	50	1.60	42	1	NS	K-E	
C/Hong/Kong/262662/2019	S1	NS/NS1	129	2990	T	A	72	2.41	43	3	S	A	
C/Hong/Kong/262662/2019	S1	NS/NS1	130	3010	A	G	70	2.32	44	1	NS	R-G	
C/Hong/Kong/263686/2019	S1	PB1	2096	1525	C	G	26	1.70	699	2	NS	A-G	
C/Hong/Kong/263686/2019	S1	P3	1271	6476	G	A	94	1.45	424	2	NS	G-E	
C/Hong/Kong/263686/2019	S1	CM/CM1	85	5491	A	G	161	2.93	29	1	NS	I-V	E
C/Hong/Kong/263686/2019	S1	CM/CM1	86	5490	T	A	179	3.26	29	2	NS	I-K	
C/Hong/Kong/263686/2019	S1	CM/CM1	88	5539	A	G	207	3.74	30	1	NS	T-A	
C/Hong/Kong/263686/2019	S1	CM/CM1	93	5461	T	G	183	3.34	31	3	S	G	
C/Hong/Kong/263686/2019	S1	NS/NS1	650	7084	G	A	81	1.14	217	2	NS	G-E	
C/Hong/Kong/263686/2019	S1	NS/NS2	650	7084	G	A	81	1.14	113	1	NS	G-R	
C/Hong/Kong/264671/2019	K	PB1	1872	2220	G	A	40	1.80	624	3	S	R	
C/Hong/Kong/264671/2019	K	PB1	2096	1413	C	G	27	1.89	699	2	NS	A-G	
C/Hong/Kong/264671/2019	K	P3	1404	7665	G	A	79	1.03	468	3	S	R	

Virus name[a]	Clade[b]	Gene/ORF[c]	Nucleotide number[d]	Total Reads	Concensus nucleotide[e]	Alternate nucleotide[e]	Supporting Reads	Frequency of alternate (%)	Amino acid position[f]	Codon position[g]	S/NS[h]	Amino acid substitution[i]	Double nucleotide substitution[j]
C/Hong/Kong/264671/2019	K	CM/CM1	414	18553	C	T	295	1.59	138	3	S	D	
C/Hong/Kong/264733/2019	S1	HE/HEF1	852	11079	C	T	235	2.12	270	3	S	T	
C/Hong/Kong/264733/2019	S1	HE/HEF2	1690	15479	C	T	1911	12.35	550	1	S	L	
C/Hong/Kong/264733/2019	S1	PB2	1688	2795	C	G	35	1.25	563	2	NS	A-G	
C/Hong/Kong/264733/2019	S1	PB2	1711	2966	C	A	39	1.31	571	1	NS	L-I	
C/Hong/Kong/264733/2019	S1	PB2	1727	2959	G	A	44	1.48	576	2	NS	G-E	
C/Hong/Kong/264733/2019	S1	PB2	1729	3082	A	G	75	2.43	577	1	NS	T-A	
C/Hong/Kong/264733/2019	S1	PB2	1732	3837	G	A	89	2.32	578	1	NS	G-R	K
C/Hong/Kong/264733/2019	S1	PB2	1733	3915	G	A	84	2.15	578	2	NS	G-E	
C/Hong/Kong/264733/2019	S1	PB1	2092	1274	T	G	17	1.33	698	1	NS	S-A	
C/Hong/Kong/264733/2019	S1	PB1	2096	1015	C	G	23	2.26	699	2	NS	A-G	
C/Hong/Kong/264733/2019	S1	P3	1271	3780	G	A	42	1.11	424	2	NS	G-E	
C/Hong/Kong/264733/2019	S1	P3	1908	3716	C	T	49	1.32	636	3	S	D	
C/Hong/Kong/264733/2019	S1	CM/CM1	85	2880	A	G	82	2.85	29	1	NS	I-V	E
C/Hong/Kong/264733/2019	S1	CM/CM1	86	2895	T	A	72	2.49	29	2	NS	I-K	
C/Hong/Kong/264733/2019	S1	CM/CM1	88	2910	A	G	90	3.09	30	1	NS	T-A	
C/Hong/Kong/264733/2019	S1	CM/CM1	93	2899	T	G	54	1.85	31	3	S	G	
C/Hong/Kong/264733/2019	S1	CM/CM2	913	4719	G	A	119	2.52	70	1	NS	V-I	
C/Hong/Kong/264733/2019	S1	NS/NS1	121	1689	A	G	25	1.48	41	1	NS	T-A	
C/Hong/Kong/264733/2019	S1	NS/NS1	129	1684	T	A	19	1.13	43	3	S	A	
C/Hong/Kong/264733/2019	S1	NS/NS1	138	1781	A	G	24	1.35	46	3	S	R	
C/Hong/Kong/264733/2019	S1	NS/NS1	151	1851	T	C	58	3.13	51	1	NS	F-L	
C/Hong/Kong/264733/2019	S1	NS/NS1	195	1970	T	C	64	3.25	65	3	S	L	
C/Hong/Kong/264733/2019	S1	NS/NS1	197	1958	T	C	47	2.40	66	2	NS	L-P	
C/Hong/Kong/268915/2019	S1	HE/HEF2	1886	13417	G	A	139	1.04	615	2	NS	S-N	
C/Hong/Kong/268915/2019	S1	PB2	1729	4544	A	G	94	2.07	577	1	NS	T-A	
C/Hong/Kong/268915/2019	S1	PB2	1733	6937	G	A	100	1.44	578	2	NS	G-E	
C/Hong/Kong/268915/2019	S1	PB2	2075	8477	G	A	1074	12.67	692	2	NS	R-K	
C/Hong/Kong/268915/2019	S1	P3	1338	4830	G	T	51	1.06	446	3	NS	M-I	
C/Hong/Kong/268915/2019	S1	CM/CM1	28	2843	A	G	61	2.15	10	1	NS	T-A	
C/Hong/Kong/268915/2019	S1	CM/CM1	85	3753	A	G	57	1.52	29	1	NS	I-V	E
C/Hong/Kong/268915/2019	S1	CM/CM1	86	3758	T	A	61	1.62	29	2	NS	I-K	
C/Hong/Kong/268915/2019	S1	CM/CM1	88	3784	A	G	72	1.90	30	1	NS	T-A	
C/Hong/Kong/268915/2019	S1	CM/CM1	93	3878	T	G	75	1.93	31	3	S	G	
C/Hong/Kong/268915/2019	S1	NS/NS1	121	2899	A	G	149	5.14	41	1	NS	T-A	
C/Hong/Kong/268915/2019	S1	NS/NS1	124	2931	A	G	117	3.99	42	1	NS	K-E	
C/Hong/Kong/268915/2019	S1	NS/NS1	129	2937	T	A	160	5.45	43	3	S	A	
C/Hong/Kong/268915/2019	S1	NS/NS1	130	2957	A	G	150	5.07	44	1	NS	R-G	
C/Hong/Kong/268428/2019	S1	HE/HEF2	1398	1505	A	G	163	10.83	452	3	S	G	
C/Hong/Kong/268428/2019	S1	HE/HEF2	1399	1506	A	G	116	7.70	453	1	NS	I-V	
C/Hong/Kong/268428/2019	S1	HE/HEF2	1432	1763	T	G	95	5.39	464	1	NS	S-A	
C/Hong/Kong/268428/2019	S1	HE/HEF2	1437	1803	A	G	55	3.05	465	3	S	G	
C/Hong/Kong/270077/2019	K	PB2	1729	4513	A	G	55	1.22	577	1	NS	T-A	
C/Hong/Kong/270077/2019	K	P3	1923	1428	C	T	81	5.67	641	3	S	G	

Virus name[a]	Clade[b]	Gene/ORF[c]	Nucleotide number[d]	Total Reads	Concensus nucleotide[e]	Alternate nucleotide[e]	Supporting Reads	Frequency of alternate (%)	Amino acid position[f]	Codon position[g]	S/NS[h]	Amino acid substitution[i]	Double nucleotide substitution[j]
C/Hong/Kong/271985/2019	?	CM/CM2	958	300	A	AGCAG	58	19.33	85	4ins		fs	
C/Hong/Kong/273848/2019	S2	HE/HEF1	604	1086	G	A	55	5.06	188	1	NS	E-K	
C/Hong/Kong/275315/2019	S2	CM/CM2	802	1359	C	A	93	6.84	33	1	NS	L-I	
C/Hong/Kong/275952/2019	S1	HE/HEF1	426	3586	T	A	60	1.67	128	3	S	S	
C/Hong/Kong/280832/2019	S1	NP	733	4054	C	G	60	1.48	245	1	NS	L-V	
C/Hong/Kong/280832/2019	S1	NP	1274	4661	T	G	806	17.29	425	2	NS	V-G	
C/Hong/Kong/280339/2019	S1	PB2	300	1887	T	C	58	3.07	100	3	S	S	
C/Hong/Kong/280339/2019	S1	PB2	1729	4964	A	G	119	2.40	577	1	NS	T-A	
C/Hong/Kong/280339/2019	S1	PB1	2092	1742	T	G	149	8.55	698	1	NS	S-A	
C/Hong/Kong/280339/2019	S1	PB1	2096	1770	C	G	174	9.83	699	2	NS	A-G	
C/Hong/Kong/280339/2019	S1	NP	1576	8869	T	C	1520	17.14	526	1	NS	S-P	
C/Hong/Kong/280339/2019	S1	CM/CM1	85	4372	A	G	73	1.67	29	1	NS	I-V	E
C/Hong/Kong/280339/2019	S1	CM/CM1	86	4382	T	A	73	1.67	29	2	NS	I-K	
C/Hong/Kong/280339/2019	S1	CM/CM1	88	4399	A	G	85	1.93	30	1	NS	T-A	
C/Hong/Kong/280339/2019	S1	CM/CM1	93	4505	T	G	86	1.91	31	3	S	G	
C/Hong/Kong/280339/2019	S1	CM/CM2	952	6305	G	A	69	1.09	83	2	NS	G-D	
C/Hong/Kong/280339/2019	S1	CM/CM2	958	6166	T	A	63	1.02	85	1	NS	W-R	
C/Hong/Kong/280339/2019	S1	NS/NS1	121	2761	A	G	95	3.44	41	1	NS	T-A	
C/Hong/Kong/280339/2019	S1	NS/NS1	124	2786	A	G	79	2.84	42	1	NS	K-E	
C/Hong/Kong/280339/2019	S1	NS/NS1	129	2779	T	A	102	3.67	43	3	S	A	
C/Hong/Kong/280339/2019	S1	NS/NS1	130	2800	A	G	95	3.39	44	1	NS	R-G	
C/Hong/Kong/280339/2019	S1	NS/NS1	224	3436	A	G	51	1.48	75	2	NS	E-G	
C/Hong/Kong/281471/2019	S2	HE/HEF2	1690	11167	C	T	1966	17.61	550	1	S	L	
C/Hong/Kong/281471/2019	S2	PB2	1729	3284	A	G	53	1.61	577	1	NS	T-A	
C/Hong/Kong/281471/2019	S2	NP	711	9219	G	A	1829	19.84	237	3	S	A	
C/Hong/Kong/281471/2019	S2	CM/CM1	28	2867	A	G	67	2.34	10	1	NS	T-A	
C/Hong/Kong/281471/2019	S2	CM/CM1	32	2876	A	G	52	1.81	11	2	NS	E-G	
C/Hong/Kong/281471/2019	S2	CM/CM1	88	3609	A	G	51	1.41	30	1	NS	T-A	
C/Hong/Kong/281471/2019	S2	CM/CM1	93	3681	T	G	55	1.49	31	3	S	G	
C/Hong/Kong/3124/2020	K	HE/HEF2	1845	4024	C	A	163	4.05	601	3	NS	D-E	
C/Hong/Kong/2433/2020	S2	HE/HEF2	1432	4728	T	G	69	1.46	464	1	NS	S-A	
C/Hong/Kong/5029/2020	K	HE/HEF2	1459	17873	G	T	262	1.47	473	1	NS	A-S	
C/Hong/Kong/5029/2020	K	PB1	2092	787	T	G	144	18.30	698	1	NS	S-A	
C/Hong/Kong/5029/2020	K	CM/CM1	28	4240	A	G	74	1.75	10	1	NS	T-A	
C/Hong/Kong/5029/2020	K	CM/CM1	32	4287	A	G	55	1.28	11	2	NS	E-G	
C/Hong/Kong/5029/2020	K	CM/CM1	85	6059	A	G	71	1.17	29	1	NS	I-V	E
C/Hong/Kong/5029/2020	K	CM/CM1	86	6066	T	A	73	1.20	29	2	NS	I-K	
C/Hong/Kong/5029/2020	K	CM/CM1	88	6111	A	G	85	1.39	30	1	NS	T-A	
C/Hong/Kong/5029/2020	K	CM/CM1	93	6279	T	G	89	1.42	31	3	S	G	
C/Hong/Kong/5029/2020	K	CM/CM2	773	11337	C	T	157	1.38	23	2	NS	S-F	
C/Hong/Kong/17410/2020	K	HE/HEF2	1388	2314	T	C	53	2.29	449	2	NS	V-A	
C/Hong/Kong/17410/2020	K	HE/HEF2	1389	2335	T	A	66	2.83	449	3	S	V	
C/Hong/Kong/17410/2020	K	HE/HEF2	1432	6316	T	G	490	7.76	464	1	NS	S-A	
C/Hong/Kong/17410/2020	K	HE/HEF2	1437	6580	A	G	257	3.91	453	3	S	G	

Virus name[a]	Clade[b]	Gene/ORF[c]	Nucleotide number[d]	Total Reads	Concensus nucleotide[e]	Alternate nucleotide[e]	Supporting Reads	Frequency of alternate (%)	Amino acid position[f]	Codon position[g]	S/NS[h]	Amino acid substitution[i]	Double nucleotide substitution[j]
C/Hong/Kong/17410/2020	K	HE/HEF2	1461	7351	T	A	204	2.78	473	3	S	A	
C/Hong/Kong/17410/2020	K	HE/HEF2	1465	7423	A	G	196	2.64	475	1	NS	K-E	A
C/Hong/Kong/17410/2020	K	HE/HEF2	1466	7443	A	C	201	2.70	475	2	NS	K-T	
C/Hong/Kong/17410/2020	K	PB2	540	973	T	C	85	8.74	180	3	S	P	
C/Hong/Kong/17410/2020	K	PB2	1018	1314	A	T	141	10.73	340	1	NS	M-L	
C/Hong/Kong/17410/2020	K	P3	1271	536	G	A	62	11.57	424	2	NS	G-E	
C/Hong/Kong/17410/2020	K	CM/CM1	86	1512	T	A	53	3.51	29	2	NS	I-K	
C/Hong/Kong/17410/2020	K	CM/CM1	88	1509	A	G	56	3.71	30	1	NS	T-A	
C/Hong/Kong/17410/2020	K	CM/CM2	773	2445	C	T	51	2.09	23	2	NS	S-F	
C/Hong/Kong/25005/2020	S1	HE/HEF1	482	16217	C	T	357	2.20	147	2	NS	S-F	
C/Hong/Kong/25005/2020	S1	HE/HEF2	1840	12109	C	A	275	2.27	600	1	NS	L-I	
C/Hong/Kong/25005/2020	S1	PB2	722	3390	C	T	53	1.56	241	2	NS	A-V	
C/Hong/Kong/25005/2020	S1	P3	1676	4709	T	C	77	1.64	559	2	NS	I-T	
C/Hong/Kong/25005/2020	S1	NP	282	7127	A	G	113	1.59	94	3	S	R	
C/Hong/Kong/25005/2020	S1	CM/CM1	223	5687	G	A	156	2.74	75	1	NS	A-T	
C/Hong/Kong/25005/2020	S1	CM/CM1	271	6021	G	A	1148	19.07	91	1	NS	G-R	
C/Hong/Kong/25005/2020	S1	NS/NS2	846	1364	G	A	83	6.09	178	2	NS	C-Y	
C/Hong/Kong/32464/2020	S1	HE/HEF1	291	9196	G	A	464	5.05	83	3	S	L	
C/Hong/Kong/35991/2020	S1	HE/HEF2	1389	3092	T	A	106	3.43	449	3	S	V-I	
C/Hong/Kong/35991/2020	S1	HE/HEF2	1432	7182	T	G	391	5.44	464	1	NS	S-A	
C/Hong/Kong/35991/2020	S1	HE/HEF2	1437	7402	A	G	278	3.76	465	3	S	G	
C/Hong/Kong/35991/2020	S1	PB2	1018	780	A	T	61	7.82	340	1	NS	M-L	
C/Hong/Kong/35991/2020	S1	PB2	1729	7288	A	G	76	1.04	577	1	NS	T-A	
C/Hong/Kong/35991/2020	S1	CM/CM1	28	2636	A	G	118	4.48	10	1	NS	T-A	
C/Hong/Kong/35991/2020	S1	CM/CM1	32	2670	A	G	107	4.01	11	2	NS	E-G	
C/Hong/Kong/35991/2020	S1	CM/CM1	85	3596	A	G	131	3.64	29	1	NS	I-V	E
C/Hong/Kong/35991/2020	S1	CM/CM1	86	3609	T	A	165	4.57	29	2	NS	I-K	
C/Hong/Kong/35991/2020	S1	CM/CM1	88	3613	A	G	175	4.84	30	1	NS	T-A	
C/Hong/Kong/35991/2020	S1	CM/CM1	93	3664	T	G	156	4.26	31	3	S	G	
C/Hong/Kong/35991/2020	S1	NS/NS1	121	3112	A	G	238	7.65	41	1	NS	T-A	
C/Hong/Kong/35991/2020	S1	NS/NS1	124	3145	A	G	180	5.72	42	1	NS	K-E	
C/Hong/Kong/35991/2020	S1	NS/NS1	129	3144	T	A	239	7.60	43	3	S	A	
C/Hong/Kong/35991/2020	S1	NS/NS1	130	3217	A	G	240	7.46	44	1	NS	R-G	
C/Hong/Kong/35991/2020	S1	NS/NS1	224	4208	A	G	151	3.59	75	2	NS	E-G	

Results of Varscan/bcftools analyses are shown for alternate nucleotides supported by at least 10 reads. a) Virus names are based the clinical specimen codes. b) Virus clade is indicated: Kanagawa (K), São Paulo 1 (S1) and São Paulo 2 (S2). c) The gene is indicated in all instances and for HEF the glycoprotein segment is indicated (HEF1/2) and for CM and NS the individual ORFs are indicated (CM1/2 and NS1/2). d) Nucleotide number relates to the complete ORF (inclusive of signal peptide for HEF). e) Nucleotide numbering relates to the first nucleotide where there are insertions or deletions in some reads. f) Amino acid position numbering relates to mature HEF (i.e. signal peptide removed) and numbers above 432 are in HEF2; numbering is specific for all other protein products. g) Where nucleotide insertions (ins) or deletions (del) were seen the number of nucleotides is given before the abbreviation. h) Mutations resulting in synonymous (S) or non-synonymous (NS) amino acid substitutions and indicated. i) Amino acid substitutions, with generation of stop codons (*), and reading-frame shifts (fs) caused by indels are indicated. j) Potential amino acid substitutions caused by mutations at two positions within the same codon are indicated.

Table S3. Summary of variant calls in the internal genes of ICVs from Hong Kong collected in 2019-20

Protein ORF	Number of variant calls	Number of viruses affected	Number of nucleotide positions involved	Amino acid substitution[a]	Range of variant levels (%)	Number of viruses with variant	Amino acids at the corresponding positions in viruses represented in the phylogenies presented in Figure S1[b]
PB2	80	31	34	M340L	1.9 - 10.9	4	All M
				T577A	1.0 - 5.0	23	All T
				G578R	1.0 - 2.3	5	No R
				G578E	1.1-8.8	12	2E - C/Yamagata/9/2006, C/Hong Kong/16909/2016
PB1	33	20	11	S698A	1.3-18.3	7	2A - C/Hong Kong/1498/2016, C/Hong Kong/3363/2018
				A699G	1.2-18.9	16	2G - C/Hong Kong/1498/2016, C/Hong Kong/3363/2018
P3	41	22	25	G424E	1.3-18.0	15	1R - C/Yamagata/1/2005
NP	27	18	23	G173E	1.3-1.9	3	All G
CM1	93	27	24	T10A	1.1-14.9	16	2A - C/Johannesburg/1/66, C/Johannesburg/4/67
				E11G	1.0 - 12.3	14	All E
				I29V	1.2-3.6	10	All I
				I29K	1.2-4.6	10	All I
				T30A	1.4-4.8	12	All T
				S160G	1.0 - 1.9	3	1N - C/Hong Kong/35991/2020
CM2	18	12	12	S23F	1.4-2.1	5	1F - C/Hiroshima/290/99
NS1	55	19	23	T41A	1.5-7.7	9	All T
				K42E	1.3-5.7	6	All K
				R44G	1.4-7.5	8	All R
				E75G	1.5-3.6	5	1Deleted[c] - C/JJ/1950, 1X[d] - C/Hong Kong/258168/2019
NS2	10	8	10				

Data for the internal genes presented in Table S2 is summarized. [a]Encoded amino acid substitutions are indicated when they occurred in at least three ICVs. Most variant calls were synonymous (i.e. did not cause amino acid substitution). [b]For the amino acid positions identified in column 5, numbers of amino acid substitutions among the sequences from ICVs used to generate the phylogenies in Figure S1 are shown: All = all contain the consensus amino acid; No = none of the amino acid indicated; numbers of sequences carrying a particular substitution are indicated followed by the ICV names; [c]C/JJ/1950 contains a seven amino acid deletion (residues 71-77); [d]C/Hong Kong/258168/2019 contained E75E/G polymorphism (E45:G55).

Table S4. GISAID accession numbers for gene sequences downloaded and used in phylogeny

Segment ID	Segment	Country	Collection date	Isolate-ID	Isolate name	Originating Lab	Submitting Lab	Authors
EPI231537	HE	Japan	1981-Jan-01	EPI_ISL_66336	C/Aichi/1/81		Import from public-domain	Matsuzaki,Y.; Sugawara,K.; Furuse,Y.; Shimotai,Y.; Hongo,S.; Oshitani,H.; Mizuta,K.; Nishimura,H.
EPI232028	MP	Japan	1981-Jan-01	EPI_ISL_66336	C/Aichi/1/81		Import from public-domain	Matsuzaki,Y.; Sugawara,K.; Furuse,Y.; Shimotai,Y.; Hongo,S.; Oshitani,H.; Mizuta,K.; Nishimura,H.
EPI816583	NP	Japan	1981-Jan-01	EPI_ISL_66336	C/Aichi/1/81		Import from public-domain	Matsuzaki,Y.; Sugawara,K.; Furuse,Y.; Shimotai,Y.; Hongo,S.; Oshitani,H.; Mizuta,K.; Nishimura,H.
EPI231542	NS	Japan	1981-Jan-01	EPI_ISL_66336	C/Aichi/1/81		Import from public-domain	Matsuzaki,Y.; Sugawara,K.; Furuse,Y.; Shimotai,Y.; Hongo,S.; Oshitani,H.; Mizuta,K.; Nishimura,H.
EPI816484	P3	Japan	1981-Jan-01	EPI_ISL_66336	C/Aichi/1/81		Import from public-domain	Matsuzaki,Y.; Sugawara,K.; Furuse,Y.; Shimotai,Y.; Hongo,S.; Oshitani,H.; Mizuta,K.; Nishimura,H.
EPI813815	PB1	Japan	1981-Jan-01	EPI_ISL_66336	C/Aichi/1/81		Import from public-domain	Matsuzaki,Y.; Sugawara,K.; Furuse,Y.; Shimotai,Y.; Hongo,S.; Oshitani,H.; Mizuta,K.; Nishimura,H.
EPI814538	PB2	Japan	1981-Jan-01	EPI_ISL_66336	C/Aichi/1/81		Import from public-domain	Matsuzaki,Y.; Sugawara,K.; Furuse,Y.; Shimotai,Y.; Hongo,S.; Oshitani,H.; Mizuta,K.; Nishimura,H.
EPI231580	HE	Japan	1999-Apr-07	EPI_ISL_66363	C/Aichi/1/99		Import from public-domain	Matsuzaki,Y.; Sugawara,K.; Furuse,Y.; Shimotai,Y.; Hongo,S.; Oshitani,H.; Mizuta,K.; Nishimura,H.
EPI231585	MP	Japan	1999-Apr-07	EPI_ISL_66363	C/Aichi/1/99		Import from public-domain	Matsuzaki,Y.; Sugawara,K.; Furuse,Y.; Shimotai,Y.; Hongo,S.; Oshitani,H.; Mizuta,K.; Nishimura,H.
EPI816611	NP	Japan	1999-Apr-07	EPI_ISL_66363	C/Aichi/1/99		Import from public-domain	Matsuzaki,Y.; Sugawara,K.; Furuse,Y.; Shimotai,Y.; Hongo,S.; Oshitani,H.; Mizuta,K.; Nishimura,H.
EPI231586	NS	Japan	1999-Apr-07	EPI_ISL_66363	C/Aichi/1/99		Import from public-domain	Matsuzaki,Y.; Sugawara,K.; Furuse,Y.; Shimotai,Y.; Hongo,S.; Oshitani,H.; Mizuta,K.; Nishimura,H.
EPI816506	P3	Japan	1999-Apr-07	EPI_ISL_66363	C/Aichi/1/99		Import from public-domain	Matsuzaki,Y.; Sugawara,K.; Furuse,Y.; Shimotai,Y.; Hongo,S.; Oshitani,H.; Mizuta,K.; Nishimura,H.
EPI816423	PB1	Japan	1999-Apr-07	EPI_ISL_66363	C/Aichi/1/99		Import from public-domain	Matsuzaki,Y.; Sugawara,K.; Furuse,Y.; Shimotai,Y.; Hongo,S.; Oshitani,H.; Mizuta,K.; Nishimura,H.
EPI813742	PB2	Japan	1999-Apr-07	EPI_ISL_66363	C/Aichi/1/99		Import from public-domain	Matsuzaki,Y.; Sugawara,K.; Furuse,Y.; Shimotai,Y.; Hongo,S.; Oshitani,H.; Mizuta,K.; Nishimura,H.
EPI403452	HE	Canada	2011-Apr-15	EPI_ISL_131508	C/Alberta/10161/2011		Import from public-domain	Pabbaraju,K.; Wong,S.; Wong,A.; May-Hadford,J.; Tellier,R.; Fonseca,K.
EPI403467	MP	Canada	2011-Apr-15	EPI_ISL_131508	C/Alberta/10161/2011		Import from public-domain	Pabbaraju,K.; Wong,S.; Wong,A.; May-Hadford,J.; Tellier,R.; Fonseca,K.
EPI403464	MP	Canada	2010-Dec-21	EPI_ISL_131515	C/Alberta/21100/2010		Import from public-domain	Pabbaraju,K.; Wong,S.; Wong,A.; May-Hadford,J.; Tellier,R.; Fonseca,K.
EPI403465	MP	Canada	2011-Jan-24	EPI_ISL_131516	C/Alberta/2193/2011		Import from public-domain	Pabbaraju,K.; Wong,S.; Wong,A.; May-Hadford,J.; Tellier,R.; Fonseca,K.
EPI403458	MP	Canada	2011-Feb-25	EPI_ISL_131513	C/Alberta/2616/2011		Import from public-domain	Pabbaraju,K.; Wong,S.; Wong,A.; May-Hadford,J.; Tellier,R.; Fonseca,K.
EPI403447	HE	Canada	2011-Mar-04	EPI_ISL_131503	C/Alberta/2921/2011		Import from public-domain	Pabbaraju,K.; Wong,S.; Wong,A.; May-Hadford,J.; Tellier,R.; Fonseca,K.
EPI403459	MP	Canada	2011-Mar-04	EPI_ISL_131503	C/Alberta/2921/2011		Import from public-domain	Pabbaraju,K.; Wong,S.; Wong,A.; May-Hadford,J.; Tellier,R.; Fonseca,K.
EPI403448	HE	Canada	2011-Mar-04	EPI_ISL_131504	C/Alberta/3087/2011		Import from public-domain	Pabbaraju,K.; Wong,S.; Wong,A.; May-Hadford,J.; Tellier,R.; Fonseca,K.
EPI403460	MP	Canada	2011-Mar-04	EPI_ISL_131504	C/Alberta/3087/2011		Import from public-domain	Pabbaraju,K.; Wong,S.; Wong,A.; May-Hadford,J.; Tellier,R.; Fonseca,K.
EPI403449	HE	Canada	2011-Mar-18	EPI_ISL_131505	C/Alberta/3502/2011		Import from public-domain	Pabbaraju,K.; Wong,S.; Wong,A.; May-Hadford,J.; Tellier,R.; Fonseca,K.
EPI403461	MP	Canada	2011-Mar-18	EPI_ISL_131505	C/Alberta/3502/2011		Import from public-domain	Pabbaraju,K.; Wong,S.; Wong,A.; May-Hadford,J.; Tellier,R.; Fonseca,K.
EPI403462	MP	Canada	2011-Apr-08	EPI_ISL_131514	C/Alberta/4406/2011		Import from public-domain	Pabbaraju,K.; Wong,S.; Wong,A.; May-Hadford,J.; Tellier,R.; Fonseca,K.
EPI403450	HE	Canada	2011-Feb-19	EPI_ISL_131506	C/Alberta/4753/2011		Import from public-domain	Pabbaraju,K.; Wong,S.; Wong,A.; May-Hadford,J.; Tellier,R.; Fonseca,K.
EPI403463	MP	Canada	2011-Feb-19	EPI_ISL_131506	C/Alberta/4753/2011		Import from public-domain	Pabbaraju,K.; Wong,S.; Wong,A.; May-Hadford,J.; Tellier,R.; Fonseca,K.
EPI403451	HE	Canada	2011-Feb-22	EPI_ISL_131507	C/Alberta/4941/2011		Import from public-domain	Pabbaraju,K.; Wong,S.; Wong,A.; May-Hadford,J.; Tellier,R.; Fonseca,K.
EPI403466	MP	Canada	2011-Feb-22	EPI_ISL_131507	C/Alberta/4941/2011		Import from public-domain	Pabbaraju,K.; Wong,S.; Wong,A.; May-Hadford,J.; Tellier,R.; Fonseca,K.
EPI232040	HE	United States	1950-Jan-01	EPI_ISL_66438	C/Ann Arbor/1/50		Import from public-domain	Muraki,Y.; Washioka,H.; Sugawara,K.; Matsuzaki,Y.; Takashita,E.; Hongo,S.
EPI232042	MP	United States	1950-Jan-01	EPI_ISL_66438	C/Ann Arbor/1/50		Import from public-domain	Muraki,Y.; Washioka,H.; Sugawara,K.; Matsuzaki,Y.; Takashita,E.; Hongo,S.
EPI232041	NP	United States	1950-Jan-01	EPI_ISL_66438	C/Ann Arbor/1/50		Import from public-domain	Muraki,Y.; Washioka,H.; Sugawara,K.; Matsuzaki,Y.; Takashita,E.; Hongo,S.
EPI232043	NS	United States	1950-Jan-01	EPI_ISL_66438	C/Ann Arbor/1/50		Import from public-domain	Muraki,Y.; Washioka,H.; Sugawara,K.; Matsuzaki,Y.; Takashita,E.; Hongo,S.
EPI232039	P3	United States	1950-Jan-01	EPI_ISL_66438	C/Ann Arbor/1/50		Import from public-domain	Muraki,Y.; Washioka,H.; Sugawara,K.; Matsuzaki,Y.; Takashita,E.; Hongo,S.
EPI232038	PB1	United States	1950-Jan-01	EPI_ISL_66438	C/Ann Arbor/1/50		Import from public-domain	Muraki,Y.; Washioka,H.; Sugawara,K.; Matsuzaki,Y.; Takashita,E.; Hongo,S.
EPI231512	PB2	United States	1950-Jan-01	EPI_ISL_66315	C/Ann Arbor/1/50		Import from public-domain	Lapatschek,M.S.
EPI232045	HE	Japan	1974-Jan-01	EPI_ISL_66326	C/Aomori/74		Import from public-domain	Matsuzaki,Y.; Sugawara,K.; Furuse,Y.; Shimotai,Y.; Hongo,S.; Oshitani,H.; Mizuta,K.; Nishimura,H.
EPI232044	MP	Japan	1974-Jan-01	EPI_ISL_66326	C/Aomori/74		Import from public-domain	Matsuzaki,Y.; Sugawara,K.; Furuse,Y.; Shimotai,Y.; Hongo,S.; Oshitani,H.; Mizuta,K.; Nishimura,H.
EPI816575	NP	Japan	1974-Jan-01	EPI_ISL_66326	C/Aomori/74		Import from public-domain	Matsuzaki,Y.; Sugawara,K.; Furuse,Y.; Shimotai,Y.; Hongo,S.; Oshitani,H.; Mizuta,K.; Nishimura,H.
EPI231527	NS	Japan	1974-Jan-01	EPI_ISL_66326	C/Aomori/74		Import from public-domain	Matsuzaki,Y.; Sugawara,K.; Furuse,Y.; Shimotai,Y.; Hongo,S.; Oshitani,H.; Mizuta,K.; Nishimura,H.
EPI816465	P3	Japan	1974-Jan-01	EPI_ISL_66326	C/Aomori/74		Import from public-domain	Matsuzaki,Y.; Sugawara,K.; Furuse,Y.; Shimotai,Y.; Hongo,S.; Oshitani,H.; Mizuta,K.; Nishimura,H.
EPI813785	PB1	Japan	1974-Jan-01	EPI_ISL_66326	C/Aomori/74		Import from public-domain	Matsuzaki,Y.; Sugawara,K.; Furuse,Y.; Shimotai,Y.; Hongo,S.; Oshitani,H.; Mizuta,K.; Nishimura,H.
EPI814893	PB2	Japan	1974-Jan-01	EPI_ISL_66326	C/Aomori/74		Import from public-domain	Matsuzaki,Y.; Sugawara,K.; Furuse,Y.; Shimotai,Y.; Hongo,S.; Oshitani,H.; Mizuta,K.; Nishimura,H.
EPI231592	PB2	Germany	1985-Jan-01	EPI_ISL_66364	C/Berlin/1/85		Import from public-domain	Santibanez Koref,M.F.
EPI1183991	HE	Germany	2013-Feb-11	EPI_ISL_300539	C/Berlin/13-03024/2013		Robert Koch Institute Nationales Referenzzentrum für Influenza	Biere, B.; Fritsch, A.; Schweiger, B.
EPI581546	HE	Philippines	2013-Jan-01	EPI_ISL_176781	C/Biliran/1/2013		Import from public-domain	Odagiri,T.; Matsuzaki,Y.; Okamoto,M.; Hongo,S.; Oshitani,H.
EPI621713	MP	Philippines	2013-Jan-01	EPI_ISL_176781	C/Biliran/1/2013		Import from public-domain	Odagiri,T.; Matsuzaki,Y.; Okamoto,M.; Hongo,S.; Oshitani,H.
EPI621697	NS	Philippines	2013-Jan-01	EPI_ISL_176781	C/Biliran/1/2013		Import from public-domain	Odagiri,T.; Matsuzaki,Y.; Okamoto,M.; Hongo,S.; Oshitani,H.
EPI581547	HE	Philippines	2013-Jan-01	EPI_ISL_176782	C/Biliran/2/2013		Import from public-domain	Odagiri,T.; Matsuzaki,Y.; Okamoto,M.; Hongo,S.; Oshitani,H.
EPI621716	MP	Philippines	2013-Jan-01	EPI_ISL_176782	C/Biliran/2/2013		Import from public-domain	Odagiri,T.; Matsuzaki,Y.; Okamoto,M.; Hongo,S.; Oshitani,H.
EPI621698	NS	Philippines	2013-Jan-01	EPI_ISL_176782	C/Biliran/2/2013		Import from public-domain	Odagiri,T.; Matsuzaki,Y.; Okamoto,M.; Hongo,S.; Oshitani,H.
EPI581548	HE	Philippines	2013-Jan-01	EPI_ISL_176783	C/Biliran/3/2013		Import from public-domain	Odagiri,T.; Matsuzaki,Y.; Okamoto,M.; Hongo,S.; Oshitani,H.
EPI621715	MP	Philippines	2013-Jan-01	EPI_ISL_176783	C/Biliran/3/2013		Import from public-domain	Odagiri,T.; Matsuzaki,Y.; Okamoto,M.; Hongo,S.; Oshitani,H.
EPI621699	NS	Philippines	2013-Jan-01	EPI_ISL_176783	C/Biliran/3/2013		Import from public-domain	Odagiri,T.; Matsuzaki,Y.; Okamoto,M.; Hongo,S.; Oshitani,H.
EPI1183993	HE	Germany	2013-Mar-04	EPI_ISL_300541	C/Brandenburg/13-04332/2013		Robert Koch Institute Nationales Referenzzentrum für Influenza	Biere, B.; Fritsch, A.; Schweiger, B.
EPI1183996	HE	Germany	2013-Mar-25	EPI_ISL_300544	C/Brandenburg/13-05206/2013		Robert Koch Institute Nationales Referenzzentrum für Influenza	Biere, B.; Fritsch, A.; Schweiger, B.
EPI1432119	HE	France	2018-Mar-05	EPI_ISL_354111	C/Bretagne/1196/2018	Institut Pasteur	Institut Pasteur	Behillil,S
EPI1432103	HE	France	2013-Dec-23	EPI_ISL_354105	C/Bretagne/2503/2013	Institut Pasteur	Institut Pasteur	
EPI1432107	MP	France	2013-Dec-23	EPI_ISL_354105	C/Bretagne/2503/2013	Institut Pasteur	Institut Pasteur	
EPI1432106	NP	France	2013-Dec-23	EPI_ISL_354105	C/Bretagne/2503/2013	Institut Pasteur	Institut Pasteur	
EPI1432108	NS	France	2013-Dec-23	EPI_ISL_354105	C/Bretagne/2503/2013	Institut Pasteur	Institut Pasteur	
EPI1432105	PB1	France	2013-Dec-23	EPI_ISL_354105	C/Bretagne/2503/2013	Institut Pasteur	Institut Pasteur	
EPI1432104	PB2	France	2013-Dec-23	EPI_ISL_354105	C/Bretagne/2503/2013	Institut Pasteur	Institut Pasteur	
EPI814531	HE	United States	1978-Aug-01	EPI_ISL_230379	C/California/78		Import from public-domain	Matsuzaki,Y.; Sugawara,K.; Furuse,Y.; Shimotai,Y.; Hongo,S.; Oshitani,H.; Mizuta,K.; Nishimura,H.
EPI231593	MP	United States	1978-Jan-01	EPI_ISL_66365	C/California/78		Import from public-domain	Tada,Y.
EPI816578	NP	United States	1978-Aug-01	EPI_ISL_230379	C/California/78		Import from public-domain	Matsuzaki,Y.; Sugawara,K.; Furuse,Y.; Shimotai,Y.; Hongo,S.; Oshitani,H.; Mizuta,K.; Nishimura,H.
EPI232049	NS	United States	1978-Jan-01	EPI_ISL_66365	C/California/78		Import from public-domain	Tada,Y.
EPI816469	P3	United States	1978-Aug-01	EPI_ISL_230379	C/California/78		Import from public-domain	Matsuzaki,Y.; Sugawara,K.; Furuse,Y.; Shimotai,Y.; Hongo,S.; Oshitani,H.; Mizuta,K.; Nishimura,H.
EPI815406	PB1	United States	1978-Aug-01	EPI_ISL_230379	C/California/78		Import from public-domain	Matsuzaki,Y.; Sugawara,K.; Furuse,Y.; Shimotai,Y.; Hongo,S.; Oshitani,H.; Mizuta,K.; Nishimura,H.
EPI814532	PB2	United States	1978-Aug-01	EPI_ISL_230379	C/California/78		Import from public-domain	Matsuzaki,Y.; Sugawara,K.; Furuse,Y.; Shimotai,Y.; Hongo,S.; Oshitani,H.; Mizuta,K.; Nishimura,H.
EPI1259829	HE	Cameroon	2017-Dec-27	EPI_ISL_315877	C/Cameroon/13560/2017	Centre Pasteur du Cameroun	Crick Worldwide Influenza Centre	
EPI1259832	MP	Cameroon	2017-Dec-27	EPI_ISL_315877	C/Cameroon/13560/2017	Centre Pasteur du Cameroun	Crick Worldwide Influenza Centre	
EPI1259830	NP	Cameroon	2017-Dec-27	EPI_ISL_315877	C/Cameroon/13560/2017	Centre Pasteur du Cameroun	Crick Worldwide Influenza Centre	
EPI1259831	NS	Cameroon	2017-Dec-27	EPI_ISL_315877	C/Cameroon/13560/2017	Centre Pasteur du Cameroun	Crick Worldwide Influenza Centre	
EPI1259828	P3	Cameroon	2017-Dec-27	EPI_ISL_315877	C/Cameroon/13560/2017	Centre Pasteur du Cameroun	Crick Worldwide Influenza Centre	
EPI1259834	PB1	Cameroon	2017-Dec-27	EPI_ISL_315877	C/Cameroon/13560/2017	Centre Pasteur du Cameroun	Crick Worldwide Influenza Centre	
EPI1259833	PB2	Cameroon	2017-Dec-27	EPI_ISL_315877	C/Cameroon/13560/2017	Centre Pasteur du Cameroun	Crick Worldwide Influenza Centre	
EPI1259835	HE	Cameroon	2017-Dec-27	EPI_ISL_315878	C/Cameroon/13565/2017	Centre Pasteur du Cameroun	Crick Worldwide Influenza Centre	
EPI272316	HE	Spain	2009-Aug-25	EPI_ISL_77214	C/Catalonia/1266/2009		Import from public-domain	Anton,A.; de Molina,P.; Marcos,M.A.; Martinez,A.; Cardenosa,N.; Godoy,P.; Torner,N.; Martinez,M.J.; Ramon,S.; Tudo,G.; Isanta,R.; Gonzalo,V.; Jimenez de Anta,M.T.; Pumarola,T.
EPI272317	HE	Spain	2009-Aug-26	EPI_ISL_77215	C/Catalonia/1284/2009		Import from public-domain	Anton,A.; de Molina,P.; Marcos,M.A.; Martinez,A.; Cardenosa,N.; Godoy,P.; Torner,N.; Martinez,M.J.; Ramon,S.; Tudo,G.; Isanta,R.; Gonzalo,V.; Jimenez de Anta,M.T.; Pumarola,T.
EPI272318	HE	Spain	2009-Sep-01	EPI_ISL_77216	C/Catalonia/1318/2009		Import from public-domain	Anton,A.; de Molina,P.; Marcos,M.A.; Martinez,A.; Cardenosa,N.; Godoy,P.; Torner,N.; Martinez,M.J.; Ramon,S.; Tudo,G.; Isanta,R.; Gonzalo,V.; Jimenez de Anta,M.T.; Pumarola,T.
EPI272319	HE	Spain	2009-Sep-14	EPI_ISL_77217	C/Catalonia/1372/2009		Import from public-domain	Anton,A.; de Molina,P.; Marcos,M.A.; Martinez,A.; Cardenosa,N.; Godoy,P.; Torner,N.; Martinez,M.J.; Ramon,S.; Tudo,G.; Isanta,R.; Gonzalo,V.; Jimenez de Anta,M.T.; Pumarola,T.
EPI272320	HE	Spain	2009-Sep-09	EPI_ISL_77218	C/Catalonia/1373/2009		Import from public-domain	Anton,A.; de Molina,P.; Marcos,M.A.; Martinez,A.; Cardenosa,N.; Godoy,P.; Torner,N.; Martinez,M.J.; Ramon,S.; Tudo,G.; Isanta,R.; Gonzalo,V.; Jimenez de Anta,M.T.; Pumarola,T.
EPI272321	HE	Spain	2009-Sep-28	EPI_ISL_77219	C/Catalonia/1430/2009		Import from public-domain	Anton,A.; de Molina,P.; Marcos,M.A.; Martinez,A.; Cardenosa,N.; Godoy,P.; Torner,N.; Martinez,M.J.; Ramon,S.; Tudo,G.; Isanta,R.; Gonzalo,V.; Jimenez de Anta,M.T.; Pumarola,T.
EPI272322	HE	Spain	2009-Sep-29	EPI_ISL_77220	C/Catalonia/1457/2009		Import from public-domain	Anton,A.; de Molina,P.; Marcos,M.A.; Martinez,A.; Cardenosa,N.; Godoy,P.; Torner,N.; Martinez,M.J.; Ramon,S.; Tudo,G.; Isanta,R.; Gonzalo,V.; Jimenez de Anta,M.T.; Pumarola,T.
EPI272323	HE	Spain	2009-Nov-09	EPI_ISL_77221	C/Catalonia/1754/2009		Import from public-domain	Anton,A.; de Molina,P.; Marcos,M.A.; Martinez,A.; Cardenosa,N.; Godoy,P.; Torner,N.; Martinez,M.J.; Ramon,S.; Tudo,G.; Isanta,R.; Gonzalo,V.; Jimenez de Anta,M.T.; Pumarola,T.
EPI272324	HE	Spain	2009-Nov-11	EPI_ISL_77222	C/Catalonia/1824/2009		Import from public-domain	Anton,A.; de Molina,P.; Marcos,M.A.; Martinez,A.; Cardenosa,N.; Godoy,P.; Torner,N.; Martinez,M.J.; Ramon,S.; Tudo,G.; Isanta,R.; Gonzalo,V.; Jimenez de Anta,M.T.; Pumarola,T.
EPI272325	HE	Spain	2009-Nov-23	EPI_ISL_77223	C/Catalonia/1945/2009		Import from public-domain	Anton,A.; de Molina,P.; Marcos,M.A.; Martinez,A.; Cardenosa,N.; Godoy,P.; Torner,N.; Martinez,M.J.; Ramon,S.; Tudo,G.; Isanta,R.; Gonzalo,V.; Jimenez de Anta,M.T.; Pumarola,T.
EPI272326	HE	Spain	2009-Dec-09	EPI_ISL_77224	C/Catalonia/2072/2009		Import from public-domain	Anton,A.; de Molina,P.; Marcos,M.A.; Martinez,A.; Cardenosa,N.; Godoy,P.; Torner,N.; Martinez,M.J.; Ramon,S.; Tudo,G.; Isanta,R.; Gonzalo,V.; Jimenez de Anta,M.T.; Pumarola,T.
EPI272327	HE	Spain	2010-Apr-13	EPI_ISL_77225	C/Catalonia/2588/2010		Import from public-domain	Anton,A.; de Molina,P.; Marcos,M.A.; Martinez,A.; Cardenosa,N.; Godoy,P.; Torner,N.; Martinez,M.J.; Ramon,S.; Tudo,G.; Isanta,R.; Gonzalo,V.; Jimenez de Anta,M.T.; Pumarola,T.
EPI1345812	HE	Spain	2016-Dec-25	EPI_ISL_336014	C/Catalonia/NSVH100540232/2016	Hospital Universitari Vall d'Hebron	Hospital Universitari Vall d'Hebron	
EPI1345816	HE	Spain	2017-Jan-28	EPI_ISL_336018	C/Catalonia/NSVH100562094/2017	Hospital Universitari Vall d'Hebron	Hospital Universitari Vall d'Hebron	
EPI1345814	HE	Spain	2017-Mar-10	EPI_ISL_336016	C/Catalonia/NSVH100585686/2017	Hospital Universitari Vall d'Hebron	Hospital Universitari Vall d'Hebron	
EPI1440611	HE	Spain	2019-Jan-26	EPI_ISL_355939	C/Catalonia/NSVH100972890/2019	Hospital Universitari Vall d'Hebron	Hospital Universitari Vall d'Hebron	
EPI1345818	HE	Spain	2017-Apr-07	EPI_ISL_336020	C/Catalonia/NSVH110463212/2017	Hospital Universitari Vall d'Hebron	Hospital Universitari Vall d'Hebron	
EPI1345815	HE	Spain	2017-Apr-23	EPI_ISL_336017	C/Catalonia/NSVH110469004/2017	Hospital Universitari Vall d'Hebron	Hospital Universitari Vall d'Hebron	
EPI1345813	HE	Spain	2017-Jun-10	EPI_ISL_336015	C/Catalonia/NSVH110476640/2017	Hospital Universitari Vall d'Hebron	Hospital Universitari Vall d'Hebron	
EPI1324929	HE	Spain	2018-Aug-25	EPI_ISL_332166	C/Catalonia/NSVH110683183/2018	Hospital Universitari Vall d'Hebron	Hospital Universitari Vall d'Hebron	
EPI463757	HE	India	2011-Jul-15	EPI_ISL_144547	C/Eastern India/1202/2011		Import from public-domain	Roy Mukherjee,T.; Chawla-Sarkar,M.
EPI463762	MP	India	2011-Jul-15	EPI_ISL_144547	C/Eastern India/1202/2011		Import from public-domain	Roy Mukherjee,T.; Chawla-Sarkar,M.
EPI463758	NP	India	2011-Jul-15	EPI_ISL_144547	C/Eastern India/1202/2011		Import from public-domain	Roy Mukherjee,T.; Chawla-Sarkar,M.
EPI463777	NS	India	2011-Jul-15	EPI_ISL_144547	C/Eastern India/1202/2011		Import from public-domain	Roy Mukherjee,T.; Chawla-Sarkar,M.
EPI463759	P3	India	2011-Jul-15	EPI_ISL_144547	C/Eastern India/1202/2011		Import from public-domain	Roy Mukherjee,T.; Chawla-Sarkar,M.
EPI463760	PB1	India	2011-Jul-15	EPI_ISL_144547	C/Eastern India/1202/2011		Import from public-domain	Roy Mukherjee,T.; Chawla-Sarkar,M.
EPI463761	PB2	India	2011-Jul-15	EPI_ISL_144547	C/Eastern India/1202/2011		Import from public-domain	Roy Mukherjee,T.; Chawla-Sarkar,M.
EPI232051	MP	United Kingdom	1983-Jan-01	EPI_ISL_66366	C/England/83		Import from public-domain	Gao,P.
EPI816590	NP	United Kingdom	1983-Aug-01	EPI_ISL_230236	C/England/892/1983		Import from public-domain	Matsuzaki,Y.; Sugawara,K.; Furuse,Y.; Shimotai,Y.; Hongo,S.; Oshitani,H.; Mizuta,K.; Nishimura,H.
EPI816491	P3	United Kingdom	1983-Aug-01	EPI_ISL_230236	C/England/892/1983		Import from public-domain	Matsuzaki,Y.; Sugawara,K.; Furuse,Y.; Shimotai,Y.; Hongo,S.; Oshitani,H.; Mizuta,K.; Nishimura,H.
EPI813794	PB1	United Kingdom	1983-Aug-01	EPI_ISL_230236	C/England/892/1983		Import from public-domain	Matsuzaki,Y.; Sugawara,K.; Furuse,Y.; Shimotai,Y.; Hongo,S.; Oshitani,H.; Mizuta,K.; Nishimura,H.
EPI813697	PB2	United Kingdom	1983-Aug-01	EPI_ISL_230236	C/England/892/1983		Import from public-domain	Matsuzaki,Y.; Sugawara,K.; Furuse,Y.; Shimotai,Y.; Hongo,S.; Oshitani,H.; Mizuta,K.; Nishimura,H.
EPI231554	HE	United Kingdom	1983-Jan-01	EPI_ISL_66345	C/England/892/83		Import from public-domain	Buonagurio,D.A.; Nakada,S.; Desselberger,U.; Krystal,M.; Palese,P.
EPI816743	NS	United Kingdom	1983-Jan-01	EPI_ISL_230236	C/England/892/83		Import from public-domain	Matsuzaki,Y.; Sugawara,K.; Furuse,Y.; Shimotai,Y.; Hongo,S.; Oshitani,H.; Mizuta,K.; Nishimura,H.
EPI711153	HE	Fiji	2012-Jul-10	EPI_ISL_212091	C/Fiji/18/2012	National Centre for Scientific Services for Virology and Vector Borne Diseases	WHO Collaborating Centre for Reference and Research on Influenza	
EPI813667	HE	Japan	2005-Dec-28	EPI_ISL_230252	C/Fukuoka/1/2005		Import from public-domain	Matsuzaki,Y.; Sugawara,K.; Furuse,Y.; Shimotai,Y.; Hongo,S.; Oshitani,H.; Mizuta,K.; Nishimura,H.
EPI816698	MP	Japan	2005-Dec-28	EPI_ISL_230252	C/Fukuoka/1/2005		Import from public-domain	Matsuzaki,Y.; Sugawara,K.; Furuse,Y.; Shimotai,Y.; Hongo,S.; Oshitani,H.; Mizuta,K.; Nishimura,H.
EPI816632	NP	Japan	2005-Dec-28	EPI_ISL_230252	C/Fukuoka/1/2005		Import from public-domain	Matsuzaki,Y.; Sugawara,K.; Furuse,Y.; Shimotai,Y.; Hongo,S.; Oshitani,H.; Mizuta,K.; Nishimura,H.
EPI816767	NS	Japan	2005-Dec-28	EPI_ISL_230252	C/Fukuoka/1/2005		Import from public-domain	Matsuzaki,Y.; Sugawara,K.; Furuse,Y.; Shimotai,Y.; Hongo,S.; Oshitani,H.; Mizuta,K.; Nishimura,H.
EPI816531	P3	Japan	2005-Dec-28	EPI_ISL_230252	C/Fukuoka/1/2005		Import from public-domain	Matsuzaki,Y.; Sugawara,K.; Furuse,Y.; Shimotai,Y.; Hongo,S.; Oshitani,H.; Mizuta,K.; Nishimura,H.
EPI813813	PB1	Japan	2005-Dec-28	EPI_ISL_230252	C/Fukuoka/1/2005		Import from public-domain	Matsuzaki,Y.; Sugawara,K.; Furuse,Y.; Shimotai,Y.; Hongo,S.; Oshitani,H.; Mizuta,K.; Nishimura,H.
EPI813716	PB2	Japan	2005-Dec-28	EPI_ISL_230252	C/Fukuoka/1/2005		Import from public-domain	Matsuzaki,Y.; Sugawara,K.; Furuse,Y.; Shimotai,Y.; Hongo,S.; Oshitani,H.; Mizuta,K.; Nishimura,H.
EPI813687	HE	Japan	2012-Apr-11	EPI_ISL_230272	C/Fukuoka/1/2012		Import from public-domain	Matsuzaki,Y.; Sugawara,K.; Furuse,Y.; Shimotai,Y.; Hongo,S.; Oshitani,H.; Mizuta,K.; Nishimura,H.
EPI816723	MP	Japan	2012-Apr-11	EPI_ISL_230272	C/Fukuoka/1/2012		Import from public-domain	Matsuzaki,Y.; Sugawara,K.; Furuse,Y.; Shimotai,Y.; Hongo,S.; Oshitani,H.; Mizuta,K.; Nishimura,H.
EPI816655	NP	Japan	2012-Apr-11	EPI_ISL_230272	C/Fukuoka/1/2012		Import from public-domain	Matsuzaki,Y.; Sugawara,K.; Furuse,Y.; Shimotai,Y.; Hongo,S.; Oshitani,H.; Mizuta,K.; Nishimura,H.
EPI816792	NS	Japan	2012-Apr-11	EPI_ISL_230272	C/Fukuoka/1/2012		Import from public-domain	Matsuzaki,Y.; Sugawara,K.; Furuse,Y.; Shimotai,Y.; Hongo,S.; Oshitani,H.; Mizuta,K.; Nishimura,H.
EPI816556	P3	Japan	2012-Apr-11	EPI_ISL_230272	C/Fukuoka/1/2012		Import from public-domain	Matsuzaki,Y.; Sugawara,K.; Furuse,Y.; Shimotai,Y.; Hongo,S.; Oshitani,H.; Mizuta,K.; Nishimura,H.
EPI816452	PB1	Japan	2012-Apr-11	EPI_ISL_230272	C/Fukuoka/1/2012		Import from public-domain	Matsuzaki,Y.; Sugawara,K.; Furuse,Y.; Shimotai,Y.; Hongo,S.; Oshitani,H.; Mizuta,K.; Nishimura,H.

Segment ID	Segment	Country	Collection date	Isolate-ID	Isolate name	Originating Lab	Submitting Lab	Authors
EPI813762	PB2	Japan	2012-Apr-11	EPI_ISL_230272	C/Fukuoka/1/2012		Import from public-domain	Matsuzaki,Y.; Sugawara,K.; Furuse,Y.; Shimotai,Y.; Hongo,S.; Oshitani,H.; Mizuta,K.; Nishimura,H.
EPI228330	HE	Japan	2004-Jan-01	EPI_ISL_65166	C/Fukuoka/2/2004		Import from public-domain	Matsuzaki,Y.
EPI813668	HE	Japan	2006-Mar-08	EPI_ISL_230253	C/Fukuoka/2/2006		Import from public-domain	Matsuzaki,Y.; Sugawara,K.; Furuse,Y.; Shimotai,Y.; Hongo,S.; Oshitani,H.; Mizuta,K.; Nishimura,H.
EPI816699	MP	Japan	2006-Mar-08	EPI_ISL_230253	C/Fukuoka/2/2006		Import from public-domain	Matsuzaki,Y.; Sugawara,K.; Furuse,Y.; Shimotai,Y.; Hongo,S.; Oshitani,H.; Mizuta,K.; Nishimura,H.
EPI816633	NP	Japan	2006-Mar-08	EPI_ISL_230253	C/Fukuoka/2/2006		Import from public-domain	Matsuzaki,Y.; Sugawara,K.; Furuse,Y.; Shimotai,Y.; Hongo,S.; Oshitani,H.; Mizuta,K.; Nishimura,H.
EPI816768	NS	Japan	2006-Mar-08	EPI_ISL_230253	C/Fukuoka/2/2006		Import from public-domain	Matsuzaki,Y.; Sugawara,K.; Furuse,Y.; Shimotai,Y.; Hongo,S.; Oshitani,H.; Mizuta,K.; Nishimura,H.
EPI816532	P3	Japan	2006-Mar-08	EPI_ISL_230253	C/Fukuoka/2/2006		Import from public-domain	Matsuzaki,Y.; Sugawara,K.; Furuse,Y.; Shimotai,Y.; Hongo,S.; Oshitani,H.; Mizuta,K.; Nishimura,H.
EPI813814	PB1	Japan	2006-Mar-08	EPI_ISL_230253	C/Fukuoka/2/2006		Import from public-domain	Matsuzaki,Y.; Sugawara,K.; Furuse,Y.; Shimotai,Y.; Hongo,S.; Oshitani,H.; Mizuta,K.; Nishimura,H.
EPI813717	PB2	Japan	2006-Mar-08	EPI_ISL_230253	C/Fukuoka/2/2006		Import from public-domain	Matsuzaki,Y.; Sugawara,K.; Furuse,Y.; Shimotai,Y.; Hongo,S.; Oshitani,H.; Mizuta,K.; Nishimura,H.
EPI228331	HE	Japan	2004-Jan-01	EPI_ISL_65167	C/Fukuoka/3/2004		Import from public-domain	Matsuzaki,Y.
EPI228325	HE	Japan	2004-Jan-01	EPI_ISL_65161	C/Fukushima/1/2004		Import from public-domain	Matsuzaki,Y.
EPI231525	HE	United States	1969-Jan-01	EPI_ISL_66324	C/Georgia/1/69		Import from public-domain	Matsuzaki,Y.; Sugawara,K.; Furuse,Y.; Shimotai,Y.; Hongo,S.; Oshitani,H.; Mizuta,K.; Nishimura,H.
EPI816671	MP	United States	1969-Jan-01	EPI_ISL_66324	C/Georgia/1/69		Import from public-domain	Matsuzaki,Y.; Sugawara,K.; Furuse,Y.; Shimotai,Y.; Hongo,S.; Oshitani,H.; Mizuta,K.; Nishimura,H.
EPI816573	NP	United States	1969-Jan-01	EPI_ISL_66324	C/Georgia/1/69		Import from public-domain	Matsuzaki,Y.; Sugawara,K.; Furuse,Y.; Shimotai,Y.; Hongo,S.; Oshitani,H.; Mizuta,K.; Nishimura,H.
EPI816739	NS	United States	1969-Jan-01	EPI_ISL_66324	C/Georgia/1/69		Import from public-domain	Matsuzaki,Y.; Sugawara,K.; Furuse,Y.; Shimotai,Y.; Hongo,S.; Oshitani,H.; Mizuta,K.; Nishimura,H.
EPI816463	P3	United States	1969-Jan-01	EPI_ISL_66324	C/Georgia/1/69		Import from public-domain	Matsuzaki,Y.; Sugawara,K.; Furuse,Y.; Shimotai,Y.; Hongo,S.; Oshitani,H.; Mizuta,K.; Nishimura,H.
EPI813783	PB1	United States	1969-Jan-01	EPI_ISL_66324	C/Georgia/1/69		Import from public-domain	Matsuzaki,Y.; Sugawara,K.; Furuse,Y.; Shimotai,Y.; Hongo,S.; Oshitani,H.; Mizuta,K.; Nishimura,H.
EPI814812	PB2	United States	1969-Jan-01	EPI_ISL_66324	C/Georgia/1/69		Import from public-domain	Matsuzaki,Y.; Sugawara,K.; Furuse,Y.; Shimotai,Y.; Hongo,S.; Oshitani,H.; Mizuta,K.; Nishimura,H.
EPI816668	MP	United States	1954-Aug-01	EPI_ISL_230235	C/Great Lakes/1167/1954		Import from public-domain	Matsuzaki,Y.; Sugawara,K.; Furuse,Y.; Shimotai,Y.; Hongo,S.; Oshitani,H.; Mizuta,K.; Nishimura,H.
EPI816570	NP	United States	1954-Aug-01	EPI_ISL_230235	C/Great Lakes/1167/1954		Import from public-domain	Matsuzaki,Y.; Sugawara,K.; Furuse,Y.; Shimotai,Y.; Hongo,S.; Oshitani,H.; Mizuta,K.; Nishimura,H.
EPI816737	NS	United States	1954-Aug-01	EPI_ISL_230235	C/Great Lakes/1167/1954		Import from public-domain	Matsuzaki,Y.; Sugawara,K.; Furuse,Y.; Shimotai,Y.; Hongo,S.; Oshitani,H.; Mizuta,K.; Nishimura,H.
EPI816459	P3	United States	1954-Aug-01	EPI_ISL_230235	C/Great Lakes/1167/1954		Import from public-domain	Matsuzaki,Y.; Sugawara,K.; Furuse,Y.; Shimotai,Y.; Hongo,S.; Oshitani,H.; Mizuta,K.; Nishimura,H.
EPI813769	PB1	United States	1954-Aug-01	EPI_ISL_230235	C/Great Lakes/1167/1954		Import from public-domain	Matsuzaki,Y.; Sugawara,K.; Furuse,Y.; Shimotai,Y.; Hongo,S.; Oshitani,H.; Mizuta,K.; Nishimura,H.
EPI813694	PB2	United States	1954-Aug-01	EPI_ISL_230235	C/Great Lakes/1167/1954		Import from public-domain	Matsuzaki,Y.; Sugawara,K.; Furuse,Y.; Shimotai,Y.; Hongo,S.; Oshitani,H.; Mizuta,K.; Nishimura,H.
EPI231516	HE	United States	1954-Jan-01	EPI_ISL_66318	C/Great Lakes/1167/54		Import from public-domain	Buonagurio,D.A.; Nakada,S.; Desselberger,U.; Krystal,M.; Palese,P.
EPI231534	HE	Greece	1979-Jan-01	EPI_ISL_66333	C/Greece/1/79		Import from public-domain	Matsuzaki,Y.; Sugawara,K.; Furuse,Y.; Shimotai,Y.; Hongo,S.; Oshitani,H.; Mizuta,K.; Nishimura,H.
EPI816471	P3	Greece	1979-Jan-01	EPI_ISL_66333	C/Greece/1/79		Import from public-domain	Matsuzaki,Y.; Sugawara,K.; Furuse,Y.; Shimotai,Y.; Hongo,S.; Oshitani,H.; Mizuta,K.; Nishimura,H.
EPI813789	PB1	Greece	1979-Jan-01	EPI_ISL_66333	C/Greece/1/79		Import from public-domain	Matsuzaki,Y.; Sugawara,K.; Furuse,Y.; Shimotai,Y.; Hongo,S.; Oshitani,H.; Mizuta,K.; Nishimura,H.
EPI814534	PB2	Greece	1979-Jan-01	EPI_ISL_66333	C/Greece/1/79		Import from public-domain	Matsuzaki,Y.; Sugawara,K.; Furuse,Y.; Shimotai,Y.; Hongo,S.; Oshitani,H.; Mizuta,K.; Nishimura,H.
EPI231604	MP	Greece	1979-Jan-01	EPI_ISL_66367	C/Greece/79		Import from public-domain	Matsuzaki,Y.
EPI369796	NP	Greece	1979-Jan-01	EPI_ISL_118431	C/Greece/79		Import from public-domain	Matsuzaki,Y.; Ikeda,T.; Abiko,C.; Aoki,Y.; Mizuta,K.; Shimotai,Y.; Sugawara,K.; Hongo,S.
EPI231600	NS	Greece	1979-Jan-01	EPI_ISL_66367	C/Greece/79		Import from public-domain	Matsuzaki,Y.
EPI1183987	HE	Germany	2012-Nov-21	EPI_ISL_300535	C/Hamburg/13-00418/2012		Robert Koch Institute Nationales Referenzzentrum für Influenza	Biere, B.; Fritsch, A.; Schweiger, B.
EPI1432114	HE	France	2014-Apr-14	EPI_ISL_354108	C/Haute Normandie/1493/2014	Institut Pasteur	Institut Pasteur	Behillil,S
EPI1432117	HE	France	2016-Apr-01	EPI_ISL_354109	C/Haute normandie/2097/2016	Institut Pasteur	Institut Pasteur	Behillil,S
EPI1432112	HE	France	2014-Jan-30	EPI_ISL_354107	C/Haute Normandie/392/2014	Institut Pasteur	Institut Pasteur	Behillil,S
EPI1183994	HE	Germany	2013-Mar-11	EPI_ISL_300542	C/Hessen/13-04588/2013		Robert Koch Institute Nationales Referenzzentrum für Influenza	Biere, B.; Fritsch, A.; Schweiger, B.
EPI232052	HE	Japan	2000-Jan-01	EPI_ISL_66439	C/Hiroshima/246/2000		Import from public-domain	Matsuzaki,Y.
EPI232058	MP	Japan	2000-Jan-01	EPI_ISL_66439	C/Hiroshima/246/2000		Import from public-domain	Matsuzaki,Y.
EPI232057	NS	Japan	2000-Jan-01	EPI_ISL_66439	C/Hiroshima/246/2000		Import from public-domain	Matsuzaki,Y.
EPI231612	HE	Japan	2000-Jan-01	EPI_ISL_66369	C/Hiroshima/247/2000		Import from public-domain	Matsuzaki,Y.
EPI231606	MP	Japan	2000-Jan-01	EPI_ISL_66369	C/Hiroshima/247/2000		Import from public-domain	Matsuzaki,Y.
EPI231607	NS	Japan	2000-Jan-01	EPI_ISL_66369	C/Hiroshima/247/2000		Import from public-domain	Matsuzaki,Y.
EPI232060	HE	Japan	2000-Jan-01	EPI_ISL_66441	C/Hiroshima/248/2000		Import from public-domain	Matsuzaki,Y.
EPI232066	MP	Japan	2000-Jan-01	EPI_ISL_66441	C/Hiroshima/248/2000		Import from public-domain	Matsuzaki,Y.
EPI232065	NS	Japan	2000-Jan-01	EPI_ISL_66441	C/Hiroshima/248/2000		Import from public-domain	Matsuzaki,Y.
EPI231619	HE	Japan	2000-Jan-01	EPI_ISL_66371	C/Hiroshima/249/2000		Import from public-domain	Matsuzaki,Y.
EPI231615	MP	Japan	2000-Jan-01	EPI_ISL_66371	C/Hiroshima/249/2000		Import from public-domain	Matsuzaki,Y.
EPI231616	NS	Japan	2000-Jan-01	EPI_ISL_66371	C/Hiroshima/249/2000		Import from public-domain	Matsuzaki,Y.
EPI232068	HE	Japan	2000-Jan-01	EPI_ISL_66443	C/Hiroshima/250/2000		Import from public-domain	Matsuzaki,Y.
EPI232074	MP	Japan	2000-Jan-01	EPI_ISL_66443	C/Hiroshima/250/2000		Import from public-domain	Matsuzaki,Y.
EPI232073	NS	Japan	2000-Jan-01	EPI_ISL_66443	C/Hiroshima/250/2000		Import from public-domain	Matsuzaki,Y.
EPI231628	HE	Japan	2000-Jan-01	EPI_ISL_66373	C/Hiroshima/251/2000		Import from public-domain	Matsuzaki,Y.
EPI231622	MP	Japan	2000-Jan-01	EPI_ISL_66373	C/Hiroshima/251/2000		Import from public-domain	Matsuzaki,Y.
EPI231623	NS	Japan	2000-Jan-01	EPI_ISL_66373	C/Hiroshima/251/2000		Import from public-domain	Matsuzaki,Y.
EPI231630	HE	Japan	1999-Jan-01	EPI_ISL_66375	C/Hiroshima/252/99		Import from public-domain	Matsuzaki,Y.
EPI231635	MP	Japan	1999-Jan-01	EPI_ISL_66375	C/Hiroshima/252/99		Import from public-domain	Matsuzaki,Y.
EPI231636	NS	Japan	1999-Jan-01	EPI_ISL_66375	C/Hiroshima/252/99		Import from public-domain	Matsuzaki,Y.
EPI231643	HE	Japan	1999-Jan-01	EPI_ISL_66376	C/Hiroshima/290/99		Import from public-domain	Matsuzaki,Y.
EPI231639	MP	Japan	1999-Jan-01	EPI_ISL_66376	C/Hiroshima/290/99		Import from public-domain	Matsuzaki,Y.
EPI231637	NS	Japan	1999-Jan-01	EPI_ISL_66376	C/Hiroshima/290/99		Import from public-domain	Matsuzaki,Y.
EPI228329	HE	Japan	2004-Jan-01	EPI_ISL_65165	C/Hiroshima/4/2004		Import from public-domain	Matsuzaki,Y.
EPI1564306	HE	Hong Kong (SAR)	2018-Apr-29	EPI_ISL_380253	C/Hong Kong/1/2018	Government Virus Unit	Crick Worldwide Influenza Centre	
EPI1595479	MP	Hong Kong (SAR)	2018-Apr-29	EPI_ISL_380253	C/Hong Kong/1/2018	Government Virus Unit	Crick Worldwide Influenza Centre	
EPI1558362	NP	Hong Kong (SAR)	2018-Apr-29	EPI_ISL_380253	C/Hong Kong/1/2018	Government Virus Unit	Crick Worldwide Influenza Centre	
EPI1607314	NS	Hong Kong (SAR)	2018-Apr-29	EPI_ISL_380253	C/Hong Kong/1/2018	Government Virus Unit	Crick Worldwide Influenza Centre	
EPI1566812	P3	Hong Kong (SAR)	2018-Apr-29	EPI_ISL_380253	C/Hong Kong/1/2018	Government Virus Unit	Crick Worldwide Influenza Centre	
EPI1567406	PB1	Hong Kong (SAR)	2018-Apr-29	EPI_ISL_380253	C/Hong Kong/1/2018	Government Virus Unit	Crick Worldwide Influenza Centre	
EPI1590421	PB2	Hong Kong (SAR)	2018-Apr-29	EPI_ISL_380253	C/Hong Kong/1/2018	Government Virus Unit	Crick Worldwide Influenza Centre	
EPI1564309	HE	Hong Kong (SAR)	2016-Jan-23	EPI_ISL_381012	C/Hong Kong/10431/2016	Government Virus Unit	Crick Worldwide Influenza Centre	
EPI1607372	NS	Hong Kong (SAR)	2016-Jan-23	EPI_ISL_381012	C/Hong Kong/10431/2016	Government Virus Unit	Crick Worldwide Influenza Centre	
EPI1564310	HE	Hong Kong (SAR)	2016-Jan-22	EPI_ISL_381013	C/Hong Kong/10828/2016	Government Virus Unit	Crick Worldwide Influenza Centre	
EPI1564311	HE	Hong Kong (SAR)	2016-Jan-25	EPI_ISL_380240	C/Hong Kong/11106/2016	Government Virus Unit	Crick Worldwide Influenza Centre	
EPI1595480	MP	Hong Kong (SAR)	2016-Jan-25	EPI_ISL_380240	C/Hong Kong/11106/2016	Government Virus Unit	Crick Worldwide Influenza Centre	
EPI1558349	NP	Hong Kong (SAR)	2016-Jan-25	EPI_ISL_380240	C/Hong Kong/11106/2016	Government Virus Unit	Crick Worldwide Influenza Centre	
EPI1607373	NS	Hong Kong (SAR)	2016-Jan-25	EPI_ISL_380240	C/Hong Kong/11106/2016	Government Virus Unit	Crick Worldwide Influenza Centre	
EPI1805210	HE	Hong Kong (SAR)	2019-May-27	EPI_ISL_574705	C/Hong Kong/129532/2019	Government Virus Unit	Crick Worldwide Influenza Centre	
EPI1805485	MP	Hong Kong (SAR)	2019-May-27	EPI_ISL_574705	C/Hong Kong/129532/2019	Government Virus Unit	Crick Worldwide Influenza Centre	
EPI1805509	NP	Hong Kong (SAR)	2019-May-27	EPI_ISL_574705	C/Hong Kong/129532/2019	Government Virus Unit	Crick Worldwide Influenza Centre	
EPI1805532	NS	Hong Kong (SAR)	2019-May-27	EPI_ISL_574705	C/Hong Kong/129532/2019	Government Virus Unit	Crick Worldwide Influenza Centre	
EPI1805557	P3	Hong Kong (SAR)	2019-May-27	EPI_ISL_574705	C/Hong Kong/129532/2019	Government Virus Unit	Crick Worldwide Influenza Centre	
EPI1805580	PB1	Hong Kong (SAR)	2019-May-27	EPI_ISL_574705	C/Hong Kong/129532/2019	Government Virus Unit	Crick Worldwide Influenza Centre	
EPI1805600	PB2	Hong Kong (SAR)	2019-May-27	EPI_ISL_574705	C/Hong Kong/129532/2019	Government Virus Unit	Crick Worldwide Influenza Centre	
EPI1805211	HE	Hong Kong (SAR)	2019-May-29	EPI_ISL_574706	C/Hong Kong/132137/2019	Government Virus Unit	Crick Worldwide Influenza Centre	
EPI1805486	MP	Hong Kong (SAR)	2019-May-29	EPI_ISL_574706	C/Hong Kong/132137/2019	Government Virus Unit	Crick Worldwide Influenza Centre	
EPI1805510	NP	Hong Kong (SAR)	2019-May-29	EPI_ISL_574706	C/Hong Kong/132137/2019	Government Virus Unit	Crick Worldwide Influenza Centre	
EPI1805533	NS	Hong Kong (SAR)	2019-May-29	EPI_ISL_574706	C/Hong Kong/132137/2019	Government Virus Unit	Crick Worldwide Influenza Centre	
EPI1805558	P3	Hong Kong (SAR)	2019-May-29	EPI_ISL_574706	C/Hong Kong/132137/2019	Government Virus Unit	Crick Worldwide Influenza Centre	
EPI1805581	PB1	Hong Kong (SAR)	2019-May-29	EPI_ISL_574706	C/Hong Kong/132137/2019	Government Virus Unit	Crick Worldwide Influenza Centre	
EPI1805601	PB2	Hong Kong (SAR)	2019-May-29	EPI_ISL_574706	C/Hong Kong/132137/2019	Government Virus Unit	Crick Worldwide Influenza Centre	
EPI1805212	HE	Hong Kong (SAR)	2019-Jun-02	EPI_ISL_574707	C/Hong Kong/133905/2019	Government Virus Unit	Crick Worldwide Influenza Centre	
EPI1805487	MP	Hong Kong (SAR)	2019-Jun-02	EPI_ISL_574707	C/Hong Kong/133905/2019	Government Virus Unit	Crick Worldwide Influenza Centre	
EPI1805511	NP	Hong Kong (SAR)	2019-Jun-02	EPI_ISL_574707	C/Hong Kong/133905/2019	Government Virus Unit	Crick Worldwide Influenza Centre	
EPI1805534	NS	Hong Kong (SAR)	2019-Jun-02	EPI_ISL_574707	C/Hong Kong/133905/2019	Government Virus Unit	Crick Worldwide Influenza Centre	
EPI1805559	P3	Hong Kong (SAR)	2019-Jun-02	EPI_ISL_574707	C/Hong Kong/133905/2019	Government Virus Unit	Crick Worldwide Influenza Centre	
EPI1805582	PB1	Hong Kong (SAR)	2019-Jun-02	EPI_ISL_574707	C/Hong Kong/133905/2019	Government Virus Unit	Crick Worldwide Influenza Centre	
EPI1805602	PB2	Hong Kong (SAR)	2019-Jun-02	EPI_ISL_574707	C/Hong Kong/133905/2019	Government Virus Unit	Crick Worldwide Influenza Centre	
EPI1811582	HE	Hong Kong (SAR)	2020-Jan-16	EPI_ISL_574769	C/Hong Kong/13772/2020	Government Virus Unit	Crick Worldwide Influenza Centre	
EPI1805274	MP	Hong Kong (SAR)	2020-Jan-16	EPI_ISL_574769	C/Hong Kong/13772/2020	Government Virus Unit	Crick Worldwide Influenza Centre	
EPI1805317	NP	Hong Kong (SAR)	2020-Jan-16	EPI_ISL_574769	C/Hong Kong/13772/2020	Government Virus Unit	Crick Worldwide Influenza Centre	
EPI1805358	NS	Hong Kong (SAR)	2020-Jan-16	EPI_ISL_574769	C/Hong Kong/13772/2020	Government Virus Unit	Crick Worldwide Influenza Centre	
EPI1564312	HE	Hong Kong (SAR)	2018-Jan-17	EPI_ISL_381014	C/Hong Kong/13962/2018	Government Virus Unit	Crick Worldwide Influenza Centre	
EPI1595481	MP	Hong Kong (SAR)	2018-Jan-17	EPI_ISL_381014	C/Hong Kong/13962/2018	Government Virus Unit	Crick Worldwide Influenza Centre	
EPI1607315	NS	Hong Kong (SAR)	2018-Jan-17	EPI_ISL_381014	C/Hong Kong/13962/2018	Government Virus Unit	Crick Worldwide Influenza Centre	
EPI1564313	HE	Hong Kong (SAR)	2018-Jan-17	EPI_ISL_380221	C/Hong Kong/14027/2018	Government Virus Unit	Crick Worldwide Influenza Centre	
EPI1595482	MP	Hong Kong (SAR)	2018-Jan-17	EPI_ISL_380221	C/Hong Kong/14027/2018	Government Virus Unit	Crick Worldwide Influenza Centre	
EPI1558330	NP	Hong Kong (SAR)	2018-Jan-17	EPI_ISL_380221	C/Hong Kong/14027/2018	Government Virus Unit	Crick Worldwide Influenza Centre	
EPI1607316	NS	Hong Kong (SAR)	2018-Jan-17	EPI_ISL_380221	C/Hong Kong/14027/2018	Government Virus Unit	Crick Worldwide Influenza Centre	
EPI1566791	P3	Hong Kong (SAR)	2018-Jan-17	EPI_ISL_380221	C/Hong Kong/14027/2018	Government Virus Unit	Crick Worldwide Influenza Centre	
EPI1590423	PB2	Hong Kong (SAR)	2018-Jan-17	EPI_ISL_380221	C/Hong Kong/14027/2018	Government Virus Unit	Crick Worldwide Influenza Centre	
EPI1564314	HE	Hong Kong (SAR)	2016-Jan-03	EPI_ISL_380247	C/Hong Kong/1498/2016	Government Virus Unit	Crick Worldwide Influenza Centre	
EPI1595483	MP	Hong Kong (SAR)	2016-Jan-03	EPI_ISL_380247	C/Hong Kong/1498/2016	Government Virus Unit	Crick Worldwide Influenza Centre	
EPI1558356	NP	Hong Kong (SAR)	2016-Jan-03	EPI_ISL_380247	C/Hong Kong/1498/2016	Government Virus Unit	Crick Worldwide Influenza Centre	
EPI1607374	NS	Hong Kong (SAR)	2016-Jan-03	EPI_ISL_380247	C/Hong Kong/1498/2016	Government Virus Unit	Crick Worldwide Influenza Centre	
EPI1566807	P3	Hong Kong (SAR)	2016-Jan-03	EPI_ISL_380247	C/Hong Kong/1498/2016	Government Virus Unit	Crick Worldwide Influenza Centre	
EPI1567401	PB1	Hong Kong (SAR)	2016-Jan-03	EPI_ISL_380247	C/Hong Kong/1498/2016	Government Virus Unit	Crick Worldwide Influenza Centre	
EPI1590424	PB2	Hong Kong (SAR)	2016-Jan-03	EPI_ISL_380247	C/Hong Kong/1498/2016	Government Virus Unit	Crick Worldwide Influenza Centre	
EPI1805213	HE	Hong Kong (SAR)	2019-Jun-30	EPI_ISL_574708	C/Hong Kong/156012/2019	Government Virus Unit	Crick Worldwide Influenza Centre	
EPI1805488	MP	Hong Kong (SAR)	2019-Jun-30	EPI_ISL_574708	C/Hong Kong/156012/2019	Government Virus Unit	Crick Worldwide Influenza Centre	
EPI1805535	NS	Hong Kong (SAR)	2019-Jun-30	EPI_ISL_574708	C/Hong Kong/156012/2019	Government Virus Unit	Crick Worldwide Influenza Centre	
EPI1805214	HE	Hong Kong (SAR)	2019-Jul-01	EPI_ISL_574709	C/Hong Kong/156019/2019	Government Virus Unit	Crick Worldwide Influenza Centre	
EPI1805489	MP	Hong Kong (SAR)	2019-Jul-01	EPI_ISL_574709	C/Hong Kong/156019/2019	Government Virus Unit	Crick Worldwide Influenza Centre	
EPI1805512	NP	Hong Kong (SAR)	2019-Jul-01	EPI_ISL_574709	C/Hong Kong/156019/2019	Government Virus Unit	Crick Worldwide Influenza Centre	
EPI1805536	NS	Hong Kong (SAR)	2019-Jul-01	EPI_ISL_574709	C/Hong Kong/156019/2019	Government Virus Unit	Crick Worldwide Influenza Centre	
EPI1805560	P3	Hong Kong (SAR)	2019-Jul-01	EPI_ISL_574709	C/Hong Kong/156019/2019	Government Virus Unit	Crick Worldwide Influenza Centre	
EPI1805603	PB2	Hong Kong (SAR)	2019-Jul-01	EPI_ISL_574709	C/Hong Kong/156019/2019	Government Virus Unit	Crick Worldwide Influenza Centre	
EPI1564315	HE	Hong Kong (SAR)	2016-Feb-01	EPI_ISL_380239	C/Hong Kong/15639/2016	Government Virus Unit	Crick Worldwide Influenza Centre	
EPI1595484	MP	Hong Kong (SAR)	2016-Feb-01	EPI_ISL_380239	C/Hong Kong/15639/2016	Government Virus Unit	Crick Worldwide Influenza Centre	
EPI1558348	NP	Hong Kong (SAR)	2016-Feb-01	EPI_ISL_380239	C/Hong Kong/15639/2016	Government Virus Unit	Crick Worldwide Influenza Centre	
EPI1607375	NS	Hong Kong (SAR)	2016-Feb-01	EPI_ISL_380239	C/Hong Kong/15639/2016	Government Virus Unit	Crick Worldwide Influenza Centre	
EPI1566801	P3	Hong Kong (SAR)	2016-Feb-01	EPI_ISL_380239	C/Hong Kong/15639/2016	Government Virus Unit	Crick Worldwide Influenza Centre	
EPI1590425	PB2	Hong Kong (SAR)	2016-Feb-01	EPI_ISL_380239	C/Hong Kong/15639/2016	Government Virus Unit	Crick Worldwide Influenza Centre	
EPI1564316	HE	Hong Kong (SAR)	2016-Feb-03	EPI_ISL_381015	C/Hong Kong/15888/2016	Government Virus Unit	Crick Worldwide Influenza Centre	
EPI1595485	MP	Hong Kong (SAR)	2016-Feb-03	EPI_ISL_381015	C/Hong Kong/15888/2016	Government Virus Unit	Crick Worldwide Influenza Centre	
EPI1607376	NS	Hong Kong (SAR)	2016-Feb-03	EPI_ISL_381015	C/Hong Kong/15888/2016	Government Virus Unit	Crick Worldwide Influenza Centre	
EPI1564317	HE	Hong Kong (SAR)	2016-Feb-04	EPI_ISL_380237	C/Hong Kong/16909/2016	Government Virus Unit	Crick Worldwide Influenza Centre	
EPI1595486	MP	Hong Kong (SAR)	2016-Feb-04	EPI_ISL_380237	C/Hong Kong/16909/2016	Government Virus Unit	Crick Worldwide Influenza Centre	
EPI1558346	NP	Hong Kong (SAR)	2016-Feb-04	EPI_ISL_380237	C/Hong Kong/16909/2016	Government Virus Unit	Crick Worldwide Influenza Centre	
EPI1607317	NS	Hong Kong (SAR)	2016-Feb-04	EPI_ISL_380237	C/Hong Kong/16909/2016	Government Virus Unit	Crick Worldwide Influenza Centre	
EPI1590426	PB2	Hong Kong (SAR)	2016-Feb-04	EPI_ISL_380237	C/Hong Kong/16909/2016	Government Virus Unit	Crick Worldwide Influenza Centre	
EPI1564318	HE	Hong Kong (SAR)	2016-Feb-05	EPI_ISL_380236	C/Hong Kong/17233/2016	Government Virus Unit	Crick Worldwide Influenza Centre	
EPI1595487	MP	Hong Kong (SAR)	2016-Feb-05	EPI_ISL_380236	C/Hong Kong/17233/2016	Government Virus Unit	Crick Worldwide Influenza Centre	
EPI1558345	NP	Hong Kong (SAR)	2016-Feb-05	EPI_ISL_380236	C/Hong Kong/17233/2016	Government Virus Unit	Crick Worldwide Influenza Centre	
EPI1607318	NS	Hong Kong (SAR)	2016-Feb-05	EPI_ISL_380236	C/Hong Kong/17233/2016	Government Virus Unit	Crick Worldwide Influenza Centre	
EPI1566800	P3	Hong Kong (SAR)	2016-Feb-05	EPI_ISL_380236	C/Hong Kong/17233/2016	Government Virus Unit	Crick Worldwide Influenza Centre	
EPI1590427	PB2	Hong Kong (SAR)	2016-Feb-05	EPI_ISL_380236	C/Hong Kong/17233/2016	Government Virus Unit	Crick Worldwide Influenza Centre	
EPI1805215	HE	Hong Kong (SAR)	2020-Jan-20	EPI_ISL_574710	C/Hong Kong/17410/2020	Government Virus Unit	Crick Worldwide Influenza Centre	
EPI1805275	MP	Hong Kong (SAR)	2020-Jan-20	EPI_ISL_574710	C/Hong Kong/17410/2020	Government Virus Unit	Crick Worldwide Influenza Centre	
EPI1805318	NP	Hong Kong (SAR)	2020-Jan-20	EPI_ISL_574710	C/Hong Kong/17410/2020	Government Virus Unit	Crick Worldwide Influenza Centre	
EPI1805359	NS	Hong Kong (SAR)	2020-Jan-20	EPI_ISL_574710	C/Hong Kong/17410/2020	Government Virus Unit	Crick Worldwide Influenza Centre	
EPI1805400	P3	Hong Kong (SAR)	2020-Jan-20	EPI_ISL_574710	C/Hong Kong/17410/2020	Government Virus Unit	Crick Worldwide Influenza Centre	
EPI1805431	PB1	Hong Kong (SAR)	2020-Jan-20	EPI_ISL_574710	C/Hong Kong/17410/2020	Government Virus Unit	Crick Worldwide Influenza Centre	
EPI1805457	PB2	Hong Kong (SAR)	2020-Jan-20	EPI_ISL_574710	C/Hong Kong/17410/2020	Government Virus Unit	Crick Worldwide Influenza Centre	
EPI1805216	HE	Hong Kong (SAR)	2019-Aug-01	EPI_ISL_574711	C/Hong Kong/177040/2019	Government Virus Unit	Crick Worldwide Influenza Centre	
EPI1805490	MP	Hong Kong (SAR)	2019-Aug-01	EPI_ISL_574711	C/Hong Kong/177040/2019	Government Virus Unit	Crick Worldwide Influenza Centre	

Segment ID	Segment	Country	Collection date	Isolate-ID	Isolate name	Originating Lab	Submitting Lab	Authors
EPI1805513	NP	Hong Kong (SAR)	2019-Aug-01	EPI_ISL_574711	C/Hong Kong/177040/2019	Government Virus Unit	Crick Worldwide Influenza Centre	
EPI1805537	NS	Hong Kong (SAR)	2019-Aug-01	EPI_ISL_574711	C/Hong Kong/177040/2019	Government Virus Unit	Crick Worldwide Influenza Centre	
EPI1805561	P3	Hong Kong (SAR)	2019-Aug-01	EPI_ISL_574711	C/Hong Kong/177040/2019	Government Virus Unit	Crick Worldwide Influenza Centre	
EPI1805604	PB2	Hong Kong (SAR)	2019-Aug-01	EPI_ISL_574711	C/Hong Kong/177040/2019	Government Virus Unit	Crick Worldwide Influenza Centre	
EPI1805217	HE	Hong Kong (SAR)	2019-Aug-03	EPI_ISL_574712	C/Hong Kong/179364/2019	Government Virus Unit	Crick Worldwide Influenza Centre	
EPI1805491	MP	Hong Kong (SAR)	2019-Aug-03	EPI_ISL_574712	C/Hong Kong/179364/2019	Government Virus Unit	Crick Worldwide Influenza Centre	
EPI1805514	NP	Hong Kong (SAR)	2019-Aug-03	EPI_ISL_574712	C/Hong Kong/179364/2019	Government Virus Unit	Crick Worldwide Influenza Centre	
EPI1805538	NS	Hong Kong (SAR)	2019-Aug-03	EPI_ISL_574712	C/Hong Kong/179364/2019	Government Virus Unit	Crick Worldwide Influenza Centre	
EPI1805562	P3	Hong Kong (SAR)	2019-Aug-03	EPI_ISL_574712	C/Hong Kong/179364/2019	Government Virus Unit	Crick Worldwide Influenza Centre	
EPI1805583	PB1	Hong Kong (SAR)	2019-Aug-03	EPI_ISL_574712	C/Hong Kong/179364/2019	Government Virus Unit	Crick Worldwide Influenza Centre	
EPI1805605	PB2	Hong Kong (SAR)	2019-Aug-03	EPI_ISL_574712	C/Hong Kong/179364/2019	Government Virus Unit	Crick Worldwide Influenza Centre	
EPI1805218	HE	Hong Kong (SAR)	2019-Aug-10	EPI_ISL_574713	C/Hong Kong/183718/2019	Government Virus Unit	Crick Worldwide Influenza Centre	
EPI1805276	MP	Hong Kong (SAR)	2019-Aug-10	EPI_ISL_574713	C/Hong Kong/183718/2019	Government Virus Unit	Crick Worldwide Influenza Centre	
EPI1805319	NP	Hong Kong (SAR)	2019-Aug-10	EPI_ISL_574713	C/Hong Kong/183718/2019	Government Virus Unit	Crick Worldwide Influenza Centre	
EPI1805360	NS	Hong Kong (SAR)	2019-Aug-10	EPI_ISL_574713	C/Hong Kong/183718/2019	Government Virus Unit	Crick Worldwide Influenza Centre	
EPI1805401	P3	Hong Kong (SAR)	2019-Aug-10	EPI_ISL_574713	C/Hong Kong/183718/2019	Government Virus Unit	Crick Worldwide Influenza Centre	
EPI1805432	PB1	Hong Kong (SAR)	2019-Aug-10	EPI_ISL_574713	C/Hong Kong/183718/2019	Government Virus Unit	Crick Worldwide Influenza Centre	
EPI1564319	HE	Hong Kong (SAR)	2016-Jan-05	EPI_ISL_380246	C/Hong Kong/1897/2016	Government Virus Unit	Crick Worldwide Influenza Centre	
EPI1595488	MP	Hong Kong (SAR)	2016-Jan-05	EPI_ISL_380246	C/Hong Kong/1897/2016	Government Virus Unit	Crick Worldwide Influenza Centre	
EPI1558355	NP	Hong Kong (SAR)	2016-Jan-05	EPI_ISL_380246	C/Hong Kong/1897/2016	Government Virus Unit	Crick Worldwide Influenza Centre	
EPI1607377	NS	Hong Kong (SAR)	2016-Jan-05	EPI_ISL_380246	C/Hong Kong/1897/2016	Government Virus Unit	Crick Worldwide Influenza Centre	
EPI1566806	P3	Hong Kong (SAR)	2016-Jan-05	EPI_ISL_380246	C/Hong Kong/1897/2016	Government Virus Unit	Crick Worldwide Influenza Centre	
EPI1567400	PB1	Hong Kong (SAR)	2016-Jan-05	EPI_ISL_380246	C/Hong Kong/1897/2016	Government Virus Unit	Crick Worldwide Influenza Centre	
EPI1590428	PB2	Hong Kong (SAR)	2016-Jan-05	EPI_ISL_380246	C/Hong Kong/1897/2016	Government Virus Unit	Crick Worldwide Influenza Centre	
EPI1564320	HE	Hong Kong (SAR)	2016-Feb-10	EPI_ISL_380232	C/Hong Kong/19109/2016	Government Virus Unit	Crick Worldwide Influenza Centre	
EPI1595489	MP	Hong Kong (SAR)	2016-Feb-10	EPI_ISL_380232	C/Hong Kong/19109/2016	Government Virus Unit	Crick Worldwide Influenza Centre	
EPI1558341	NP	Hong Kong (SAR)	2016-Feb-10	EPI_ISL_380232	C/Hong Kong/19109/2016	Government Virus Unit	Crick Worldwide Influenza Centre	
EPI1607319	NS	Hong Kong (SAR)	2016-Feb-10	EPI_ISL_380232	C/Hong Kong/19109/2016	Government Virus Unit	Crick Worldwide Influenza Centre	
EPI1566799	P3	Hong Kong (SAR)	2016-Feb-10	EPI_ISL_380232	C/Hong Kong/19109/2016	Government Virus Unit	Crick Worldwide Influenza Centre	
EPI1567391	PB1	Hong Kong (SAR)	2016-Feb-10	EPI_ISL_380232	C/Hong Kong/19109/2016	Government Virus Unit	Crick Worldwide Influenza Centre	
EPI1590429	PB2	Hong Kong (SAR)	2016-Feb-10	EPI_ISL_380232	C/Hong Kong/19109/2016	Government Virus Unit	Crick Worldwide Influenza Centre	
EPI1805219	HE	Hong Kong (SAR)	2019-Aug-29	EPI_ISL_574714	C/Hong Kong/195607/2019	Government Virus Unit	Crick Worldwide Influenza Centre	
EPI1805492	MP	Hong Kong (SAR)	2019-Aug-29	EPI_ISL_574714	C/Hong Kong/195607/2019	Government Virus Unit	Crick Worldwide Influenza Centre	
EPI1805515	NP	Hong Kong (SAR)	2019-Aug-29	EPI_ISL_574714	C/Hong Kong/195607/2019	Government Virus Unit	Crick Worldwide Influenza Centre	
EPI1805539	NS	Hong Kong (SAR)	2019-Aug-29	EPI_ISL_574714	C/Hong Kong/195607/2019	Government Virus Unit	Crick Worldwide Influenza Centre	
EPI1805563	P3	Hong Kong (SAR)	2019-Aug-29	EPI_ISL_574714	C/Hong Kong/195607/2019	Government Virus Unit	Crick Worldwide Influenza Centre	
EPI1805584	PB1	Hong Kong (SAR)	2019-Aug-29	EPI_ISL_574714	C/Hong Kong/195607/2019	Government Virus Unit	Crick Worldwide Influenza Centre	
EPI1805606	PB2	Hong Kong (SAR)	2019-Aug-29	EPI_ISL_574714	C/Hong Kong/195607/2019	Government Virus Unit	Crick Worldwide Influenza Centre	
EPI1564321	HE	Hong Kong (SAR)	2016-Feb-08	EPI_ISL_380234	C/Hong Kong/19944/2016	Government Virus Unit	Crick Worldwide Influenza Centre	
EPI1595490	MP	Hong Kong (SAR)	2016-Feb-08	EPI_ISL_380234	C/Hong Kong/19944/2016	Government Virus Unit	Crick Worldwide Influenza Centre	
EPI1558343	NP	Hong Kong (SAR)	2016-Feb-08	EPI_ISL_380234	C/Hong Kong/19944/2016	Government Virus Unit	Crick Worldwide Influenza Centre	
EPI1607320	NS	Hong Kong (SAR)	2016-Feb-08	EPI_ISL_380234	C/Hong Kong/19944/2016	Government Virus Unit	Crick Worldwide Influenza Centre	
EPI1805220	HE	Hong Kong (SAR)	2020-Jan-03	EPI_ISL_574715	C/Hong Kong/2007/2020	Government Virus Unit	Crick Worldwide Influenza Centre	
EPI1805277	MP	Hong Kong (SAR)	2020-Jan-03	EPI_ISL_574715	C/Hong Kong/2007/2020	Government Virus Unit	Crick Worldwide Influenza Centre	
EPI1805320	NP	Hong Kong (SAR)	2020-Jan-03	EPI_ISL_574715	C/Hong Kong/2007/2020	Government Virus Unit	Crick Worldwide Influenza Centre	
EPI1805361	NS	Hong Kong (SAR)	2020-Jan-03	EPI_ISL_574715	C/Hong Kong/2007/2020	Government Virus Unit	Crick Worldwide Influenza Centre	
EPI1805221	HE	Hong Kong (SAR)	2019-Sep-06	EPI_ISL_574716	C/Hong Kong/201177/2019	Government Virus Unit	Crick Worldwide Influenza Centre	
EPI1805278	MP	Hong Kong (SAR)	2019-Sep-06	EPI_ISL_574716	C/Hong Kong/201177/2019	Government Virus Unit	Crick Worldwide Influenza Centre	
EPI1805321	NP	Hong Kong (SAR)	2019-Sep-06	EPI_ISL_574716	C/Hong Kong/201177/2019	Government Virus Unit	Crick Worldwide Influenza Centre	
EPI1805362	NS	Hong Kong (SAR)	2019-Sep-06	EPI_ISL_574716	C/Hong Kong/201177/2019	Government Virus Unit	Crick Worldwide Influenza Centre	
EPI1805402	P3	Hong Kong (SAR)	2019-Sep-06	EPI_ISL_574716	C/Hong Kong/201177/2019	Government Virus Unit	Crick Worldwide Influenza Centre	
EPI1805433	PB1	Hong Kong (SAR)	2019-Sep-06	EPI_ISL_574716	C/Hong Kong/201177/2019	Government Virus Unit	Crick Worldwide Influenza Centre	
EPI1805222	HE	Hong Kong (SAR)	2020-Jan-28	EPI_ISL_574717	C/Hong Kong/22765/2020	Government Virus Unit	Crick Worldwide Influenza Centre	
EPI1805279	MP	Hong Kong (SAR)	2020-Jan-28	EPI_ISL_574717	C/Hong Kong/22765/2020	Government Virus Unit	Crick Worldwide Influenza Centre	
EPI1805322	NP	Hong Kong (SAR)	2020-Jan-28	EPI_ISL_574717	C/Hong Kong/22765/2020	Government Virus Unit	Crick Worldwide Influenza Centre	
EPI1805363	NS	Hong Kong (SAR)	2020-Jan-28	EPI_ISL_574717	C/Hong Kong/22765/2020	Government Virus Unit	Crick Worldwide Influenza Centre	
EPI1805403	P3	Hong Kong (SAR)	2020-Jan-28	EPI_ISL_574717	C/Hong Kong/22765/2020	Government Virus Unit	Crick Worldwide Influenza Centre	
EPI1805458	PB2	Hong Kong (SAR)	2020-Jan-28	EPI_ISL_574717	C/Hong Kong/22765/2020	Government Virus Unit	Crick Worldwide Influenza Centre	
EPI1564322	HE	Hong Kong (SAR)	2016-Feb-14	EPI_ISL_381016	C/Hong Kong/22986/2016	Government Virus Unit	Crick Worldwide Influenza Centre	
EPI1595491	MP	Hong Kong (SAR)	2016-Feb-14	EPI_ISL_381016	C/Hong Kong/22986/2016	Government Virus Unit	Crick Worldwide Influenza Centre	
EPI1607378	NS	Hong Kong (SAR)	2016-Feb-14	EPI_ISL_381016	C/Hong Kong/22986/2016	Government Virus Unit	Crick Worldwide Influenza Centre	
EPI1805223	HE	Hong Kong (SAR)	2019-Oct-26	EPI_ISL_574718	C/Hong Kong/234288/2019	Government Virus Unit	Crick Worldwide Influenza Centre	
EPI1805493	MP	Hong Kong (SAR)	2019-Oct-26	EPI_ISL_574718	C/Hong Kong/234288/2019	Government Virus Unit	Crick Worldwide Influenza Centre	
EPI1805516	NP	Hong Kong (SAR)	2019-Oct-26	EPI_ISL_574718	C/Hong Kong/234288/2019	Government Virus Unit	Crick Worldwide Influenza Centre	
EPI1805540	NS	Hong Kong (SAR)	2019-Oct-26	EPI_ISL_574718	C/Hong Kong/234288/2019	Government Virus Unit	Crick Worldwide Influenza Centre	
EPI1805564	P3	Hong Kong (SAR)	2019-Oct-26	EPI_ISL_574718	C/Hong Kong/234288/2019	Government Virus Unit	Crick Worldwide Influenza Centre	
EPI1805585	PB1	Hong Kong (SAR)	2019-Oct-26	EPI_ISL_574718	C/Hong Kong/234288/2019	Government Virus Unit	Crick Worldwide Influenza Centre	
EPI1805607	PB2	Hong Kong (SAR)	2019-Oct-26	EPI_ISL_574718	C/Hong Kong/234288/2019	Government Virus Unit	Crick Worldwide Influenza Centre	
EPI1805224	HE	Hong Kong (SAR)	2019-Oct-26	EPI_ISL_574719	C/Hong Kong/234519/2019	Government Virus Unit	Crick Worldwide Influenza Centre	
EPI1805280	MP	Hong Kong (SAR)	2019-Oct-26	EPI_ISL_574719	C/Hong Kong/234519/2019	Government Virus Unit	Crick Worldwide Influenza Centre	
EPI1805323	NP	Hong Kong (SAR)	2019-Oct-26	EPI_ISL_574719	C/Hong Kong/234519/2019	Government Virus Unit	Crick Worldwide Influenza Centre	
EPI1805364	NS	Hong Kong (SAR)	2019-Oct-26	EPI_ISL_574719	C/Hong Kong/234519/2019	Government Virus Unit	Crick Worldwide Influenza Centre	
EPI1805404	P3	Hong Kong (SAR)	2019-Oct-26	EPI_ISL_574719	C/Hong Kong/234519/2019	Government Virus Unit	Crick Worldwide Influenza Centre	
EPI1805225	HE	Hong Kong (SAR)	2019-Oct-27	EPI_ISL_574720	C/Hong Kong/235164/2019	Government Virus Unit	Crick Worldwide Influenza Centre	
EPI1805494	MP	Hong Kong (SAR)	2019-Oct-27	EPI_ISL_574720	C/Hong Kong/235164/2019	Government Virus Unit	Crick Worldwide Influenza Centre	
EPI1805517	NP	Hong Kong (SAR)	2019-Oct-27	EPI_ISL_574720	C/Hong Kong/235164/2019	Government Virus Unit	Crick Worldwide Influenza Centre	
EPI1805541	NS	Hong Kong (SAR)	2019-Oct-27	EPI_ISL_574720	C/Hong Kong/235164/2019	Government Virus Unit	Crick Worldwide Influenza Centre	
EPI1805565	P3	Hong Kong (SAR)	2019-Oct-27	EPI_ISL_574720	C/Hong Kong/235164/2019	Government Virus Unit	Crick Worldwide Influenza Centre	
EPI1805586	PB1	Hong Kong (SAR)	2019-Oct-27	EPI_ISL_574720	C/Hong Kong/235164/2019	Government Virus Unit	Crick Worldwide Influenza Centre	
EPI1805608	PB2	Hong Kong (SAR)	2019-Oct-27	EPI_ISL_574720	C/Hong Kong/235164/2019	Government Virus Unit	Crick Worldwide Influenza Centre	
EPI1805226	HE	Hong Kong (SAR)	2019-Oct-28	EPI_ISL_574721	C/Hong Kong/235298/2019	Government Virus Unit	Crick Worldwide Influenza Centre	
EPI1805495	MP	Hong Kong (SAR)	2019-Oct-28	EPI_ISL_574721	C/Hong Kong/235298/2019	Government Virus Unit	Crick Worldwide Influenza Centre	
EPI1805518	NP	Hong Kong (SAR)	2019-Oct-28	EPI_ISL_574721	C/Hong Kong/235298/2019	Government Virus Unit	Crick Worldwide Influenza Centre	
EPI1805542	NS	Hong Kong (SAR)	2019-Oct-28	EPI_ISL_574721	C/Hong Kong/235298/2019	Government Virus Unit	Crick Worldwide Influenza Centre	
EPI1805566	P3	Hong Kong (SAR)	2019-Oct-28	EPI_ISL_574721	C/Hong Kong/235298/2019	Government Virus Unit	Crick Worldwide Influenza Centre	
EPI1805587	PB1	Hong Kong (SAR)	2019-Oct-28	EPI_ISL_574721	C/Hong Kong/235298/2019	Government Virus Unit	Crick Worldwide Influenza Centre	
EPI1805609	PB2	Hong Kong (SAR)	2019-Oct-28	EPI_ISL_574721	C/Hong Kong/235298/2019	Government Virus Unit	Crick Worldwide Influenza Centre	
EPI1564323	HE	Hong Kong (SAR)	2016-Jan-05	EPI_ISL_380245	C/Hong Kong/2361/2016	Government Virus Unit	Crick Worldwide Influenza Centre	
EPI1595492	MP	Hong Kong (SAR)	2016-Jan-05	EPI_ISL_380245	C/Hong Kong/2361/2016	Government Virus Unit	Crick Worldwide Influenza Centre	
EPI1558354	NP	Hong Kong (SAR)	2016-Jan-05	EPI_ISL_380245	C/Hong Kong/2361/2016	Government Virus Unit	Crick Worldwide Influenza Centre	
EPI1607379	NS	Hong Kong (SAR)	2016-Jan-05	EPI_ISL_380245	C/Hong Kong/2361/2016	Government Virus Unit	Crick Worldwide Influenza Centre	
EPI1566805	P3	Hong Kong (SAR)	2016-Jan-05	EPI_ISL_380245	C/Hong Kong/2361/2016	Government Virus Unit	Crick Worldwide Influenza Centre	
EPI1590430	PB2	Hong Kong (SAR)	2016-Jan-05	EPI_ISL_380245	C/Hong Kong/2361/2016	Government Virus Unit	Crick Worldwide Influenza Centre	
EPI1805227	HE	Hong Kong (SAR)	2019-Oct-30	EPI_ISL_574722	C/Hong Kong/237362/2019	Government Virus Unit	Crick Worldwide Influenza Centre	
EPI1805496	MP	Hong Kong (SAR)	2019-Oct-30	EPI_ISL_574722	C/Hong Kong/237362/2019	Government Virus Unit	Crick Worldwide Influenza Centre	
EPI1805519	NP	Hong Kong (SAR)	2019-Oct-30	EPI_ISL_574722	C/Hong Kong/237362/2019	Government Virus Unit	Crick Worldwide Influenza Centre	
EPI1805543	NS	Hong Kong (SAR)	2019-Oct-30	EPI_ISL_574722	C/Hong Kong/237362/2019	Government Virus Unit	Crick Worldwide Influenza Centre	
EPI1805567	P3	Hong Kong (SAR)	2019-Oct-30	EPI_ISL_574722	C/Hong Kong/237362/2019	Government Virus Unit	Crick Worldwide Influenza Centre	
EPI1805588	PB1	Hong Kong (SAR)	2019-Oct-30	EPI_ISL_574722	C/Hong Kong/237362/2019	Government Virus Unit	Crick Worldwide Influenza Centre	
EPI1805610	PB2	Hong Kong (SAR)	2019-Oct-30	EPI_ISL_574722	C/Hong Kong/237362/2019	Government Virus Unit	Crick Worldwide Influenza Centre	
EPI1805228	HE	Hong Kong (SAR)	2019-Nov-03	EPI_ISL_574723	C/Hong Kong/238624/2019	Government Virus Unit	Crick Worldwide Influenza Centre	
EPI1805497	MP	Hong Kong (SAR)	2019-Nov-03	EPI_ISL_574723	C/Hong Kong/238624/2019	Government Virus Unit	Crick Worldwide Influenza Centre	
EPI1805520	NP	Hong Kong (SAR)	2019-Nov-03	EPI_ISL_574723	C/Hong Kong/238624/2019	Government Virus Unit	Crick Worldwide Influenza Centre	
EPI1805544	NS	Hong Kong (SAR)	2019-Nov-03	EPI_ISL_574723	C/Hong Kong/238624/2019	Government Virus Unit	Crick Worldwide Influenza Centre	
EPI1805568	P3	Hong Kong (SAR)	2019-Nov-03	EPI_ISL_574723	C/Hong Kong/238624/2019	Government Virus Unit	Crick Worldwide Influenza Centre	
EPI1805611	PB2	Hong Kong (SAR)	2019-Nov-03	EPI_ISL_574723	C/Hong Kong/238624/2019	Government Virus Unit	Crick Worldwide Influenza Centre	
EPI1805229	HE	Hong Kong (SAR)	2019-Nov-02	EPI_ISL_574724	C/Hong Kong/239033/2019	Government Virus Unit	Crick Worldwide Influenza Centre	
EPI1805324	NP	Hong Kong (SAR)	2019-Nov-02	EPI_ISL_574724	C/Hong Kong/239033/2019	Government Virus Unit	Crick Worldwide Influenza Centre	
EPI1805365	NS	Hong Kong (SAR)	2019-Nov-04	EPI_ISL_574777	C/Hong Kong/239644/2019	Government Virus Unit	Crick Worldwide Influenza Centre	
EPI1805366	NS	Hong Kong (SAR)	2019-Nov-07	EPI_ISL_574778	C/Hong Kong/242252/2019	Government Virus Unit	Crick Worldwide Influenza Centre	
EPI1805230	HE	Hong Kong (SAR)	2019-Nov-08	EPI_ISL_574725	C/Hong Kong/242697/2019	Government Virus Unit	Crick Worldwide Influenza Centre	
EPI1805281	MP	Hong Kong (SAR)	2019-Nov-08	EPI_ISL_574725	C/Hong Kong/242697/2019	Government Virus Unit	Crick Worldwide Influenza Centre	
EPI1805325	NP	Hong Kong (SAR)	2019-Nov-08	EPI_ISL_574725	C/Hong Kong/242697/2019	Government Virus Unit	Crick Worldwide Influenza Centre	
EPI1805367	NS	Hong Kong (SAR)	2019-Nov-08	EPI_ISL_574725	C/Hong Kong/242697/2019	Government Virus Unit	Crick Worldwide Influenza Centre	
EPI1805405	P3	Hong Kong (SAR)	2019-Nov-08	EPI_ISL_574725	C/Hong Kong/242697/2019	Government Virus Unit	Crick Worldwide Influenza Centre	
EPI1805459	PB2	Hong Kong (SAR)	2019-Nov-08	EPI_ISL_574725	C/Hong Kong/242697/2019	Government Virus Unit	Crick Worldwide Influenza Centre	
EPI1805231	HE	Hong Kong (SAR)	2019-Nov-10	EPI_ISL_574726	C/Hong Kong/243270/2019	Government Virus Unit	Crick Worldwide Influenza Centre	
EPI1805282	MP	Hong Kong (SAR)	2019-Nov-10	EPI_ISL_574726	C/Hong Kong/243270/2019	Government Virus Unit	Crick Worldwide Influenza Centre	
EPI1805326	NP	Hong Kong (SAR)	2019-Nov-10	EPI_ISL_574726	C/Hong Kong/243270/2019	Government Virus Unit	Crick Worldwide Influenza Centre	
EPI1805368	NS	Hong Kong (SAR)	2019-Nov-10	EPI_ISL_574726	C/Hong Kong/243270/2019	Government Virus Unit	Crick Worldwide Influenza Centre	
EPI1805406	P3	Hong Kong (SAR)	2019-Nov-10	EPI_ISL_574726	C/Hong Kong/243270/2019	Government Virus Unit	Crick Worldwide Influenza Centre	
EPI1805434	PB1	Hong Kong (SAR)	2019-Nov-10	EPI_ISL_574726	C/Hong Kong/243270/2019	Government Virus Unit	Crick Worldwide Influenza Centre	
EPI1805460	PB2	Hong Kong (SAR)	2019-Nov-10	EPI_ISL_574726	C/Hong Kong/243270/2019	Government Virus Unit	Crick Worldwide Influenza Centre	
EPI1805232	HE	Hong Kong (SAR)	2020-Jan-03	EPI_ISL_574727	C/Hong Kong/2433/2020	Government Virus Unit	Crick Worldwide Influenza Centre	
EPI1805283	MP	Hong Kong (SAR)	2020-Jan-03	EPI_ISL_574727	C/Hong Kong/2433/2020	Government Virus Unit	Crick Worldwide Influenza Centre	
EPI1805327	NP	Hong Kong (SAR)	2020-Jan-03	EPI_ISL_574727	C/Hong Kong/2433/2020	Government Virus Unit	Crick Worldwide Influenza Centre	
EPI1805369	NS	Hong Kong (SAR)	2020-Jan-03	EPI_ISL_574727	C/Hong Kong/2433/2020	Government Virus Unit	Crick Worldwide Influenza Centre	
EPI1805407	P3	Hong Kong (SAR)	2020-Jan-03	EPI_ISL_574727	C/Hong Kong/2433/2020	Government Virus Unit	Crick Worldwide Influenza Centre	
EPI1805435	PB1	Hong Kong (SAR)	2020-Jan-03	EPI_ISL_574727	C/Hong Kong/2433/2020	Government Virus Unit	Crick Worldwide Influenza Centre	
EPI1805461	PB2	Hong Kong (SAR)	2020-Jan-03	EPI_ISL_574727	C/Hong Kong/2433/2020	Government Virus Unit	Crick Worldwide Influenza Centre	
EPI1805233	HE	Hong Kong (SAR)	2019-Nov-10	EPI_ISL_574728	C/Hong Kong/243520/2019	Government Virus Unit	Crick Worldwide Influenza Centre	
EPI1805284	MP	Hong Kong (SAR)	2019-Nov-10	EPI_ISL_574728	C/Hong Kong/243520/2019	Government Virus Unit	Crick Worldwide Influenza Centre	
EPI1805328	NP	Hong Kong (SAR)	2019-Nov-10	EPI_ISL_574728	C/Hong Kong/243520/2019	Government Virus Unit	Crick Worldwide Influenza Centre	
EPI1805370	NS	Hong Kong (SAR)	2019-Nov-10	EPI_ISL_574728	C/Hong Kong/243520/2019	Government Virus Unit	Crick Worldwide Influenza Centre	
EPI1805408	P3	Hong Kong (SAR)	2019-Nov-10	EPI_ISL_574728	C/Hong Kong/243520/2019	Government Virus Unit	Crick Worldwide Influenza Centre	
EPI1805436	PB1	Hong Kong (SAR)	2019-Nov-10	EPI_ISL_574728	C/Hong Kong/243520/2019	Government Virus Unit	Crick Worldwide Influenza Centre	
EPI1805462	PB2	Hong Kong (SAR)	2019-Nov-10	EPI_ISL_574728	C/Hong Kong/243520/2019	Government Virus Unit	Crick Worldwide Influenza Centre	
EPI1805285	MP	Hong Kong (SAR)	2019-Nov-11	EPI_ISL_574770	C/Hong Kong/243545/2019	Government Virus Unit	Crick Worldwide Influenza Centre	
EPI1805329	NP	Hong Kong (SAR)	2019-Nov-11	EPI_ISL_574770	C/Hong Kong/243545/2019	Government Virus Unit	Crick Worldwide Influenza Centre	
EPI1805371	NS	Hong Kong (SAR)	2019-Nov-11	EPI_ISL_574770	C/Hong Kong/243545/2019	Government Virus Unit	Crick Worldwide Influenza Centre	
EPI1805234	HE	Hong Kong (SAR)	2019-Nov-09	EPI_ISL_574729	C/Hong Kong/243709/2019	Government Virus Unit	Crick Worldwide Influenza Centre	
EPI1805286	MP	Hong Kong (SAR)	2019-Nov-09	EPI_ISL_574729	C/Hong Kong/243709/2019	Government Virus Unit	Crick Worldwide Influenza Centre	
EPI1805330	NP	Hong Kong (SAR)	2019-Nov-09	EPI_ISL_574729	C/Hong Kong/243709/2019	Government Virus Unit	Crick Worldwide Influenza Centre	
EPI1805372	NS	Hong Kong (SAR)	2019-Nov-09	EPI_ISL_574729	C/Hong Kong/243709/2019	Government Virus Unit	Crick Worldwide Influenza Centre	
EPI1805409	P3	Hong Kong (SAR)	2019-Nov-09	EPI_ISL_574729	C/Hong Kong/243709/2019	Government Virus Unit	Crick Worldwide Influenza Centre	
EPI1805437	PB1	Hong Kong (SAR)	2019-Nov-09	EPI_ISL_574729	C/Hong Kong/243709/2019	Government Virus Unit	Crick Worldwide Influenza Centre	
EPI1805463	PB2	Hong Kong (SAR)	2019-Nov-09	EPI_ISL_574729	C/Hong Kong/243709/2019	Government Virus Unit	Crick Worldwide Influenza Centre	
EPI1805235	HE	Hong Kong (SAR)	2019-Nov-11	EPI_ISL_574730	C/Hong Kong/244337/2019	Government Virus Unit	Crick Worldwide Influenza Centre	
EPI1805498	MP	Hong Kong (SAR)	2019-Nov-11	EPI_ISL_574730	C/Hong Kong/244337/2019	Government Virus Unit	Crick Worldwide Influenza Centre	
EPI1805521	NP	Hong Kong (SAR)	2019-Nov-11	EPI_ISL_574730	C/Hong Kong/244337/2019	Government Virus Unit	Crick Worldwide Influenza Centre	
EPI1805545	NS	Hong Kong (SAR)	2019-Nov-11	EPI_ISL_574730	C/Hong Kong/244337/2019	Government Virus Unit	Crick Worldwide Influenza Centre	
EPI1805569	P3	Hong Kong (SAR)	2019-Nov-11	EPI_ISL_574730	C/Hong Kong/244337/2019	Government Virus Unit	Crick Worldwide Influenza Centre	
EPI1805589	PB1	Hong Kong (SAR)	2019-Nov-11	EPI_ISL_574730	C/Hong Kong/244337/2019	Government Virus Unit	Crick Worldwide Influenza Centre	
EPI1805612	PB2	Hong Kong (SAR)	2019-Nov-11	EPI_ISL_574730	C/Hong Kong/244337/2019	Government Virus Unit	Crick Worldwide Influenza Centre	
EPI1564324	HE	Hong Kong (SAR)	2016-Feb-15	EPI_ISL_381017	C/Hong Kong/24477/2016	Government Virus Unit	Crick Worldwide Influenza Centre	
EPI1595493	MP	Hong Kong (SAR)	2016-Feb-15	EPI_ISL_381017	C/Hong Kong/24477/2016	Government Virus Unit	Crick Worldwide Influenza Centre	
EPI1607321	NS	Hong Kong (SAR)	2016-Feb-15	EPI_ISL_381017	C/Hong Kong/24477/2016	Government Virus Unit	Crick Worldwide Influenza Centre	
EPI1805236	HE	Hong Kong (SAR)	2019-Nov-11	EPI_ISL_574731	C/Hong Kong/245344/2019	Government Virus Unit	Crick Worldwide Influenza Centre	
EPI1805287	MP	Hong Kong (SAR)	2019-Nov-11	EPI_ISL_574731	C/Hong Kong/245344/2019	Government Virus Unit	Crick Worldwide Influenza Centre	
EPI1805331	NP	Hong Kong (SAR)	2019-Nov-11	EPI_ISL_574731	C/Hong Kong/245344/2019	Government Virus Unit	Crick Worldwide Influenza Centre	
EPI1805373	NS	Hong Kong (SAR)	2019-Nov-11	EPI_ISL_574731	C/Hong Kong/245344/2019	Government Virus Unit	Crick Worldwide Influenza Centre	
EPI1805410	P3	Hong Kong (SAR)	2019-Nov-11	EPI_ISL_574731	C/Hong Kong/245344/2019	Government Virus Unit	Crick Worldwide Influenza Centre	
EPI1805438	PB1	Hong Kong (SAR)	2019-Nov-11	EPI_ISL_574731	C/Hong Kong/245344/2019	Government Virus Unit	Crick Worldwide Influenza Centre	
EPI1805464	PB2	Hong Kong (SAR)	2019-Nov-11	EPI_ISL_574731	C/Hong Kong/245344/2019	Government Virus Unit	Crick Worldwide Influenza Centre	
EPI1564325	HE	Hong Kong (SAR)	2016-Feb-17	EPI_ISL_380227	C/Hong Kong/24688/2016	Government Virus Unit	Crick Worldwide Influenza Centre	
EPI1595494	MP	Hong Kong (SAR)	2016-Feb-17	EPI_ISL_380227	C/Hong Kong/24688/2016	Government Virus Unit	Crick Worldwide Influenza Centre	
EPI1558336	NP	Hong Kong (SAR)	2016-Feb-17	EPI_ISL_380227	C/Hong Kong/24688/2016	Government Virus Unit	Crick Worldwide Influenza Centre	
EPI1607322	NS	Hong Kong (SAR)	2016-Feb-17	EPI_ISL_380227	C/Hong Kong/24688/2016	Government Virus Unit	Crick Worldwide Influenza Centre	
EPI1566798	P3	Hong Kong (SAR)	2016-Feb-17	EPI_ISL_380227	C/Hong Kong/24688/2016	Government Virus Unit	Crick Worldwide Influenza Centre	
EPI1567388	PB1	Hong Kong (SAR)	2016-Feb-17	EPI_ISL_380227	C/Hong Kong/24688/2016	Government Virus Unit	Crick Worldwide Influenza Centre	
EPI1590431	PB2	Hong Kong (SAR)	2016-Feb-17	EPI_ISL_380227	C/Hong Kong/24688/2016	Government Virus Unit	Crick Worldwide Influenza Centre	
EPI1805237	HE	Hong Kong (SAR)	2019-Nov-17	EPI_ISL_574732	C/Hong Kong/248430/2019	Government Virus Unit	Crick Worldwide Influenza Centre	
EPI1805499	MP	Hong Kong (SAR)	2019-Nov-17	EPI_ISL_574732	C/Hong Kong/248430/2019	Government Virus Unit	Crick Worldwide Influenza Centre	
EPI1805522	NP	Hong Kong (SAR)	2019-Nov-17	EPI_ISL_574732	C/Hong Kong/248430/2019	Government Virus Unit	Crick Worldwide Influenza Centre	
EPI1805546	NS	Hong Kong (SAR)	2019-Nov-17	EPI_ISL_574732	C/Hong Kong/248430/2019	Government Virus Unit	Crick Worldwide Influenza Centre	
EPI1805570	P3	Hong Kong (SAR)	2019-Nov-17	EPI_ISL_574732	C/Hong Kong/248430/2019	Government Virus Unit	Crick Worldwide Influenza Centre	

Segment ID	Segment	Country	Collection date	Isolate-ID	Isolate name	Originating Lab	Submitting Lab	Authors
EPI1805590	PB1	Hong Kong (SAR)	2019-Nov-17	EPI_ISL_574732	C/Hong Kong/248430/2019	Government Virus Unit	Crick Worldwide Influenza Centre	
EPI1805613	PB2	Hong Kong (SAR)	2019-Nov-17	EPI_ISL_574732	C/Hong Kong/248430/2019	Government Virus Unit	Crick Worldwide Influenza Centre	
EPI1805238	HE	Hong Kong (SAR)	2019-Nov-16	EPI_ISL_574733	C/Hong Kong/248593/2019	Government Virus Unit	Crick Worldwide Influenza Centre	
EPI1805288	MP	Hong Kong (SAR)	2019-Nov-16	EPI_ISL_574733	C/Hong Kong/248593/2019	Government Virus Unit	Crick Worldwide Influenza Centre	
EPI1805332	NP	Hong Kong (SAR)	2019-Nov-16	EPI_ISL_574733	C/Hong Kong/248593/2019	Government Virus Unit	Crick Worldwide Influenza Centre	
EPI1805374	NS	Hong Kong (SAR)	2019-Nov-16	EPI_ISL_574733	C/Hong Kong/248593/2019	Government Virus Unit	Crick Worldwide Influenza Centre	
EPI1805411	P3	Hong Kong (SAR)	2019-Nov-16	EPI_ISL_574733	C/Hong Kong/248593/2019	Government Virus Unit	Crick Worldwide Influenza Centre	
EPI1805439	PB1	Hong Kong (SAR)	2019-Nov-16	EPI_ISL_574733	C/Hong Kong/248593/2019	Government Virus Unit	Crick Worldwide Influenza Centre	
EPI1805465	PB2	Hong Kong (SAR)	2019-Nov-16	EPI_ISL_574733	C/Hong Kong/248593/2019	Government Virus Unit	Crick Worldwide Influenza Centre	
EPI1805289	MP	Hong Kong (SAR)	2020-Jan-30	EPI_ISL_574771	C/Hong Kong/24953/2020	Government Virus Unit	Crick Worldwide Influenza Centre	
EPI1805239	HE	Hong Kong (SAR)	2020-Jan-29	EPI_ISL_574734	C/Hong Kong/25005/2020	Government Virus Unit	Crick Worldwide Influenza Centre	
EPI1805290	MP	Hong Kong (SAR)	2020-Jan-29	EPI_ISL_574734	C/Hong Kong/25005/2020	Government Virus Unit	Crick Worldwide Influenza Centre	
EPI1805333	NP	Hong Kong (SAR)	2020-Jan-29	EPI_ISL_574734	C/Hong Kong/25005/2020	Government Virus Unit	Crick Worldwide Influenza Centre	
EPI1805375	NS	Hong Kong (SAR)	2020-Jan-29	EPI_ISL_574734	C/Hong Kong/25005/2020	Government Virus Unit	Crick Worldwide Influenza Centre	
EPI1805412	P3	Hong Kong (SAR)	2020-Jan-29	EPI_ISL_574734	C/Hong Kong/25005/2020	Government Virus Unit	Crick Worldwide Influenza Centre	
EPI1805466	PB2	Hong Kong (SAR)	2020-Jan-29	EPI_ISL_574734	C/Hong Kong/25005/2020	Government Virus Unit	Crick Worldwide Influenza Centre	
EPI1805240	HE	Hong Kong (SAR)	2019-Nov-21	EPI_ISL_574735	C/Hong Kong/251160/2019	Government Virus Unit	Crick Worldwide Influenza Centre	
EPI1805500	MP	Hong Kong (SAR)	2019-Nov-21	EPI_ISL_574735	C/Hong Kong/251160/2019	Government Virus Unit	Crick Worldwide Influenza Centre	
EPI1805523	NP	Hong Kong (SAR)	2019-Nov-21	EPI_ISL_574735	C/Hong Kong/251160/2019	Government Virus Unit	Crick Worldwide Influenza Centre	
EPI1805547	NS	Hong Kong (SAR)	2019-Nov-21	EPI_ISL_574735	C/Hong Kong/251160/2019	Government Virus Unit	Crick Worldwide Influenza Centre	
EPI1805571	P3	Hong Kong (SAR)	2019-Nov-21	EPI_ISL_574735	C/Hong Kong/251160/2019	Government Virus Unit	Crick Worldwide Influenza Centre	
EPI1805591	PB1	Hong Kong (SAR)	2019-Nov-21	EPI_ISL_574735	C/Hong Kong/251160/2019	Government Virus Unit	Crick Worldwide Influenza Centre	
EPI1805614	PB2	Hong Kong (SAR)	2019-Nov-21	EPI_ISL_574735	C/Hong Kong/251160/2019	Government Virus Unit	Crick Worldwide Influenza Centre	
EPI1805241	HE	Hong Kong (SAR)	2019-Nov-20	EPI_ISL_574736	C/Hong Kong/251408/2019	Government Virus Unit	Crick Worldwide Influenza Centre	
EPI1805501	MP	Hong Kong (SAR)	2019-Nov-20	EPI_ISL_574736	C/Hong Kong/251408/2019	Government Virus Unit	Crick Worldwide Influenza Centre	
EPI1805524	NP	Hong Kong (SAR)	2019-Nov-20	EPI_ISL_574736	C/Hong Kong/251408/2019	Government Virus Unit	Crick Worldwide Influenza Centre	
EPI1805548	NS	Hong Kong (SAR)	2019-Nov-20	EPI_ISL_574736	C/Hong Kong/251408/2019	Government Virus Unit	Crick Worldwide Influenza Centre	
EPI1805572	P3	Hong Kong (SAR)	2019-Nov-20	EPI_ISL_574736	C/Hong Kong/251408/2019	Government Virus Unit	Crick Worldwide Influenza Centre	
EPI1805592	PB1	Hong Kong (SAR)	2019-Nov-20	EPI_ISL_574736	C/Hong Kong/251408/2019	Government Virus Unit	Crick Worldwide Influenza Centre	
EPI1805615	PB2	Hong Kong (SAR)	2019-Nov-20	EPI_ISL_574736	C/Hong Kong/251408/2019	Government Virus Unit	Crick Worldwide Influenza Centre	
EPI1805242	HE	Hong Kong (SAR)	2019-Nov-22	EPI_ISL_574737	C/Hong Kong/251816/2019	Government Virus Unit	Crick Worldwide Influenza Centre	
EPI1805502	MP	Hong Kong (SAR)	2019-Nov-22	EPI_ISL_574737	C/Hong Kong/251816/2019	Government Virus Unit	Crick Worldwide Influenza Centre	
EPI1805525	NP	Hong Kong (SAR)	2019-Nov-22	EPI_ISL_574737	C/Hong Kong/251816/2019	Government Virus Unit	Crick Worldwide Influenza Centre	
EPI1805549	NS	Hong Kong (SAR)	2019-Nov-22	EPI_ISL_574737	C/Hong Kong/251816/2019	Government Virus Unit	Crick Worldwide Influenza Centre	
EPI1805573	P3	Hong Kong (SAR)	2019-Nov-22	EPI_ISL_574737	C/Hong Kong/251816/2019	Government Virus Unit	Crick Worldwide Influenza Centre	
EPI1805593	PB1	Hong Kong (SAR)	2019-Nov-22	EPI_ISL_574737	C/Hong Kong/251816/2019	Government Virus Unit	Crick Worldwide Influenza Centre	
EPI1805616	PB2	Hong Kong (SAR)	2019-Nov-22	EPI_ISL_574737	C/Hong Kong/251816/2019	Government Virus Unit	Crick Worldwide Influenza Centre	
EPI1805243	HE	Hong Kong (SAR)	2019-Nov-25	EPI_ISL_574738	C/Hong Kong/253739/2019	Government Virus Unit	Crick Worldwide Influenza Centre	
EPI1805291	MP	Hong Kong (SAR)	2019-Nov-25	EPI_ISL_574738	C/Hong Kong/253739/2019	Government Virus Unit	Crick Worldwide Influenza Centre	
EPI1805334	NP	Hong Kong (SAR)	2019-Nov-25	EPI_ISL_574738	C/Hong Kong/253739/2019	Government Virus Unit	Crick Worldwide Influenza Centre	
EPI1805376	NS	Hong Kong (SAR)	2019-Nov-25	EPI_ISL_574738	C/Hong Kong/253739/2019	Government Virus Unit	Crick Worldwide Influenza Centre	
EPI1805413	P3	Hong Kong (SAR)	2019-Nov-25	EPI_ISL_574738	C/Hong Kong/253739/2019	Government Virus Unit	Crick Worldwide Influenza Centre	
EPI1805440	PB1	Hong Kong (SAR)	2019-Nov-25	EPI_ISL_574738	C/Hong Kong/253739/2019	Government Virus Unit	Crick Worldwide Influenza Centre	
EPI1805467	PB2	Hong Kong (SAR)	2019-Nov-25	EPI_ISL_574738	C/Hong Kong/253739/2019	Government Virus Unit	Crick Worldwide Influenza Centre	
EPI1805244	HE	Hong Kong (SAR)	2019-Nov-25	EPI_ISL_574739	C/Hong Kong/254352/2019	Government Virus Unit	Crick Worldwide Influenza Centre	
EPI1805245	HE	Hong Kong (SAR)	2019-Nov-28	EPI_ISL_574740	C/Hong Kong/256484/2019	Government Virus Unit	Crick Worldwide Influenza Centre	
EPI1805503	MP	Hong Kong (SAR)	2019-Nov-28	EPI_ISL_574740	C/Hong Kong/256484/2019	Government Virus Unit	Crick Worldwide Influenza Centre	
EPI1805526	NP	Hong Kong (SAR)	2019-Nov-28	EPI_ISL_574740	C/Hong Kong/256484/2019	Government Virus Unit	Crick Worldwide Influenza Centre	
EPI1805550	NS	Hong Kong (SAR)	2019-Nov-28	EPI_ISL_574740	C/Hong Kong/256484/2019	Government Virus Unit	Crick Worldwide Influenza Centre	
EPI1805574	P3	Hong Kong (SAR)	2019-Nov-28	EPI_ISL_574740	C/Hong Kong/256484/2019	Government Virus Unit	Crick Worldwide Influenza Centre	
EPI1805594	PB1	Hong Kong (SAR)	2019-Nov-28	EPI_ISL_574740	C/Hong Kong/256484/2019	Government Virus Unit	Crick Worldwide Influenza Centre	
EPI1805617	PB2	Hong Kong (SAR)	2019-Nov-28	EPI_ISL_574740	C/Hong Kong/256484/2019	Government Virus Unit	Crick Worldwide Influenza Centre	
EPI1805551	NS	Hong Kong (SAR)	2019-Nov-30	EPI_ISL_574864	C/Hong Kong/258168/2019	Government Virus Unit	Crick Worldwide Influenza Centre	
EPI1805246	HE	Hong Kong (SAR)	2019-Dec-04	EPI_ISL_574741	C/Hong Kong/261118/2019	Government Virus Unit	Crick Worldwide Influenza Centre	
EPI1805504	MP	Hong Kong (SAR)	2019-Dec-04	EPI_ISL_574741	C/Hong Kong/261118/2019	Government Virus Unit	Crick Worldwide Influenza Centre	
EPI1805527	NP	Hong Kong (SAR)	2019-Dec-04	EPI_ISL_574741	C/Hong Kong/261118/2019	Government Virus Unit	Crick Worldwide Influenza Centre	
EPI1805552	NS	Hong Kong (SAR)	2019-Dec-04	EPI_ISL_574741	C/Hong Kong/261118/2019	Government Virus Unit	Crick Worldwide Influenza Centre	
EPI1805575	P3	Hong Kong (SAR)	2019-Dec-04	EPI_ISL_574741	C/Hong Kong/261118/2019	Government Virus Unit	Crick Worldwide Influenza Centre	
EPI1805595	PB1	Hong Kong (SAR)	2019-Dec-04	EPI_ISL_574741	C/Hong Kong/261118/2019	Government Virus Unit	Crick Worldwide Influenza Centre	
EPI1805618	PB2	Hong Kong (SAR)	2019-Dec-04	EPI_ISL_574741	C/Hong Kong/261118/2019	Government Virus Unit	Crick Worldwide Influenza Centre	
EPI1805247	HE	Hong Kong (SAR)	2019-Dec-07	EPI_ISL_574742	C/Hong Kong/262364/2019	Government Virus Unit	Crick Worldwide Influenza Centre	
EPI1805292	MP	Hong Kong (SAR)	2019-Dec-07	EPI_ISL_574742	C/Hong Kong/262364/2019	Government Virus Unit	Crick Worldwide Influenza Centre	
EPI1805335	NP	Hong Kong (SAR)	2019-Dec-07	EPI_ISL_574742	C/Hong Kong/262364/2019	Government Virus Unit	Crick Worldwide Influenza Centre	
EPI1805377	NS	Hong Kong (SAR)	2019-Dec-07	EPI_ISL_574742	C/Hong Kong/262364/2019	Government Virus Unit	Crick Worldwide Influenza Centre	
EPI1805414	P3	Hong Kong (SAR)	2019-Dec-07	EPI_ISL_574742	C/Hong Kong/262364/2019	Government Virus Unit	Crick Worldwide Influenza Centre	
EPI1805441	PB1	Hong Kong (SAR)	2019-Dec-07	EPI_ISL_574742	C/Hong Kong/262364/2019	Government Virus Unit	Crick Worldwide Influenza Centre	
EPI1805468	PB2	Hong Kong (SAR)	2019-Dec-07	EPI_ISL_574742	C/Hong Kong/262364/2019	Government Virus Unit	Crick Worldwide Influenza Centre	
EPI1805248	HE	Hong Kong (SAR)	2019-Dec-07	EPI_ISL_574743	C/Hong Kong/262662/2019	Government Virus Unit	Crick Worldwide Influenza Centre	
EPI1805505	MP	Hong Kong (SAR)	2019-Dec-07	EPI_ISL_574743	C/Hong Kong/262662/2019	Government Virus Unit	Crick Worldwide Influenza Centre	
EPI1805528	NP	Hong Kong (SAR)	2019-Dec-07	EPI_ISL_574743	C/Hong Kong/262662/2019	Government Virus Unit	Crick Worldwide Influenza Centre	
EPI1805553	NS	Hong Kong (SAR)	2019-Dec-07	EPI_ISL_574743	C/Hong Kong/262662/2019	Government Virus Unit	Crick Worldwide Influenza Centre	
EPI1805576	P3	Hong Kong (SAR)	2019-Dec-07	EPI_ISL_574743	C/Hong Kong/262662/2019	Government Virus Unit	Crick Worldwide Influenza Centre	
EPI1805596	PB1	Hong Kong (SAR)	2019-Dec-07	EPI_ISL_574743	C/Hong Kong/262662/2019	Government Virus Unit	Crick Worldwide Influenza Centre	
EPI1805619	PB2	Hong Kong (SAR)	2019-Dec-07	EPI_ISL_574743	C/Hong Kong/262662/2019	Government Virus Unit	Crick Worldwide Influenza Centre	
EPI1805249	HE	Hong Kong (SAR)	2019-Dec-09	EPI_ISL_574744	C/Hong Kong/263686/2019	Government Virus Unit	Crick Worldwide Influenza Centre	
EPI1805506	MP	Hong Kong (SAR)	2019-Dec-09	EPI_ISL_574744	C/Hong Kong/263686/2019	Government Virus Unit	Crick Worldwide Influenza Centre	
EPI1805529	NP	Hong Kong (SAR)	2019-Dec-09	EPI_ISL_574744	C/Hong Kong/263686/2019	Government Virus Unit	Crick Worldwide Influenza Centre	
EPI1805554	NS	Hong Kong (SAR)	2019-Dec-09	EPI_ISL_574744	C/Hong Kong/263686/2019	Government Virus Unit	Crick Worldwide Influenza Centre	
EPI1805577	P3	Hong Kong (SAR)	2019-Dec-09	EPI_ISL_574744	C/Hong Kong/263686/2019	Government Virus Unit	Crick Worldwide Influenza Centre	
EPI1805597	PB1	Hong Kong (SAR)	2019-Dec-09	EPI_ISL_574744	C/Hong Kong/263686/2019	Government Virus Unit	Crick Worldwide Influenza Centre	
EPI1805620	PB2	Hong Kong (SAR)	2019-Dec-09	EPI_ISL_574744	C/Hong Kong/263686/2019	Government Virus Unit	Crick Worldwide Influenza Centre	
EPI1805250	HE	Hong Kong (SAR)	2019-Dec-10	EPI_ISL_574745	C/Hong Kong/264671/2019	Government Virus Unit	Crick Worldwide Influenza Centre	
EPI1805507	MP	Hong Kong (SAR)	2019-Dec-10	EPI_ISL_574745	C/Hong Kong/264671/2019	Government Virus Unit	Crick Worldwide Influenza Centre	
EPI1805530	NP	Hong Kong (SAR)	2019-Dec-10	EPI_ISL_574745	C/Hong Kong/264671/2019	Government Virus Unit	Crick Worldwide Influenza Centre	
EPI1805555	NS	Hong Kong (SAR)	2019-Dec-10	EPI_ISL_574745	C/Hong Kong/264671/2019	Government Virus Unit	Crick Worldwide Influenza Centre	
EPI1805578	P3	Hong Kong (SAR)	2019-Dec-10	EPI_ISL_574745	C/Hong Kong/264671/2019	Government Virus Unit	Crick Worldwide Influenza Centre	
EPI1805598	PB1	Hong Kong (SAR)	2019-Dec-10	EPI_ISL_574745	C/Hong Kong/264671/2019	Government Virus Unit	Crick Worldwide Influenza Centre	
EPI1805621	PB2	Hong Kong (SAR)	2019-Dec-10	EPI_ISL_574745	C/Hong Kong/264671/2019	Government Virus Unit	Crick Worldwide Influenza Centre	
EPI1805251	HE	Hong Kong (SAR)	2019-Dec-10	EPI_ISL_574746	C/Hong Kong/264733/2019	Government Virus Unit	Crick Worldwide Influenza Centre	
EPI1805508	MP	Hong Kong (SAR)	2019-Dec-10	EPI_ISL_574746	C/Hong Kong/264733/2019	Government Virus Unit	Crick Worldwide Influenza Centre	
EPI1805531	NP	Hong Kong (SAR)	2019-Dec-10	EPI_ISL_574746	C/Hong Kong/264733/2019	Government Virus Unit	Crick Worldwide Influenza Centre	
EPI1805556	NS	Hong Kong (SAR)	2019-Dec-10	EPI_ISL_574746	C/Hong Kong/264733/2019	Government Virus Unit	Crick Worldwide Influenza Centre	
EPI1805579	P3	Hong Kong (SAR)	2019-Dec-10	EPI_ISL_574746	C/Hong Kong/264733/2019	Government Virus Unit	Crick Worldwide Influenza Centre	
EPI1805599	PB1	Hong Kong (SAR)	2019-Dec-10	EPI_ISL_574746	C/Hong Kong/264733/2019	Government Virus Unit	Crick Worldwide Influenza Centre	
EPI1805622	PB2	Hong Kong (SAR)	2019-Dec-10	EPI_ISL_574746	C/Hong Kong/264733/2019	Government Virus Unit	Crick Worldwide Influenza Centre	
EPI1805252	HE	Hong Kong (SAR)	2019-Dec-15	EPI_ISL_574747	C/Hong Kong/268428/2019	Government Virus Unit	Crick Worldwide Influenza Centre	
EPI1805293	MP	Hong Kong (SAR)	2019-Dec-15	EPI_ISL_574747	C/Hong Kong/268428/2019	Government Virus Unit	Crick Worldwide Influenza Centre	
EPI1805336	NP	Hong Kong (SAR)	2019-Dec-15	EPI_ISL_574747	C/Hong Kong/268428/2019	Government Virus Unit	Crick Worldwide Influenza Centre	
EPI1805378	NS	Hong Kong (SAR)	2019-Dec-15	EPI_ISL_574747	C/Hong Kong/268428/2019	Government Virus Unit	Crick Worldwide Influenza Centre	
EPI1805253	HE	Hong Kong (SAR)	2019-Dec-15	EPI_ISL_574748	C/Hong Kong/268740/2019	Government Virus Unit	Crick Worldwide Influenza Centre	
EPI1805337	NP	Hong Kong (SAR)	2019-Dec-15	EPI_ISL_574748	C/Hong Kong/268740/2019	Government Virus Unit	Crick Worldwide Influenza Centre	
EPI1805379	NS	Hong Kong (SAR)	2019-Dec-15	EPI_ISL_574748	C/Hong Kong/268740/2019	Government Virus Unit	Crick Worldwide Influenza Centre	
EPI1805254	HE	Hong Kong (SAR)	2019-Dec-15	EPI_ISL_574749	C/Hong Kong/268915/2019	Government Virus Unit	Crick Worldwide Influenza Centre	
EPI1805294	MP	Hong Kong (SAR)	2019-Dec-15	EPI_ISL_574749	C/Hong Kong/268915/2019	Government Virus Unit	Crick Worldwide Influenza Centre	
EPI1805338	NP	Hong Kong (SAR)	2019-Dec-15	EPI_ISL_574749	C/Hong Kong/268915/2019	Government Virus Unit	Crick Worldwide Influenza Centre	
EPI1805380	NS	Hong Kong (SAR)	2019-Dec-15	EPI_ISL_574749	C/Hong Kong/268915/2019	Government Virus Unit	Crick Worldwide Influenza Centre	
EPI1805415	P3	Hong Kong (SAR)	2019-Dec-15	EPI_ISL_574749	C/Hong Kong/268915/2019	Government Virus Unit	Crick Worldwide Influenza Centre	
EPI1805442	PB1	Hong Kong (SAR)	2019-Dec-15	EPI_ISL_574749	C/Hong Kong/268915/2019	Government Virus Unit	Crick Worldwide Influenza Centre	
EPI1805469	PB2	Hong Kong (SAR)	2019-Dec-15	EPI_ISL_574749	C/Hong Kong/268915/2019	Government Virus Unit	Crick Worldwide Influenza Centre	
EPI1805255	HE	Hong Kong (SAR)	2019-Dec-17	EPI_ISL_574750	C/Hong Kong/270077/2019	Government Virus Unit	Crick Worldwide Influenza Centre	
EPI1805295	MP	Hong Kong (SAR)	2019-Dec-17	EPI_ISL_574750	C/Hong Kong/270077/2019	Government Virus Unit	Crick Worldwide Influenza Centre	
EPI1805339	NP	Hong Kong (SAR)	2019-Dec-17	EPI_ISL_574750	C/Hong Kong/270077/2019	Government Virus Unit	Crick Worldwide Influenza Centre	
EPI1805381	NS	Hong Kong (SAR)	2019-Dec-17	EPI_ISL_574750	C/Hong Kong/270077/2019	Government Virus Unit	Crick Worldwide Influenza Centre	
EPI1805416	P3	Hong Kong (SAR)	2019-Dec-17	EPI_ISL_574750	C/Hong Kong/270077/2019	Government Virus Unit	Crick Worldwide Influenza Centre	
EPI1805443	PB1	Hong Kong (SAR)	2019-Dec-17	EPI_ISL_574750	C/Hong Kong/270077/2019	Government Virus Unit	Crick Worldwide Influenza Centre	
EPI1805470	PB2	Hong Kong (SAR)	2019-Dec-17	EPI_ISL_574750	C/Hong Kong/270077/2019	Government Virus Unit	Crick Worldwide Influenza Centre	
EPI1805256	HE	Hong Kong (SAR)	2019-Dec-17	EPI_ISL_574751	C/Hong Kong/270207/2019	Government Virus Unit	Crick Worldwide Influenza Centre	
EPI1805296	MP	Hong Kong (SAR)	2019-Dec-17	EPI_ISL_574751	C/Hong Kong/270207/2019	Government Virus Unit	Crick Worldwide Influenza Centre	
EPI1805340	NP	Hong Kong (SAR)	2019-Dec-17	EPI_ISL_574751	C/Hong Kong/270207/2019	Government Virus Unit	Crick Worldwide Influenza Centre	
EPI1805382	NS	Hong Kong (SAR)	2019-Dec-17	EPI_ISL_574751	C/Hong Kong/270207/2019	Government Virus Unit	Crick Worldwide Influenza Centre	
EPI1805417	P3	Hong Kong (SAR)	2019-Dec-17	EPI_ISL_574751	C/Hong Kong/270207/2019	Government Virus Unit	Crick Worldwide Influenza Centre	
EPI1805471	PB2	Hong Kong (SAR)	2019-Dec-17	EPI_ISL_574751	C/Hong Kong/270207/2019	Government Virus Unit	Crick Worldwide Influenza Centre	
EPI1805297	MP	Hong Kong (SAR)	2019-Dec-18	EPI_ISL_574772	C/Hong Kong/271985/2019	Government Virus Unit	Crick Worldwide Influenza Centre	
EPI1805383	NS	Hong Kong (SAR)	2019-Dec-18	EPI_ISL_574772	C/Hong Kong/271985/2019	Government Virus Unit	Crick Worldwide Influenza Centre	
EPI1805257	HE	Hong Kong (SAR)	2019-Dec-19	EPI_ISL_574752	C/Hong Kong/272963/2019	Government Virus Unit	Crick Worldwide Influenza Centre	
EPI1805298	MP	Hong Kong (SAR)	2019-Dec-19	EPI_ISL_574752	C/Hong Kong/272963/2019	Government Virus Unit	Crick Worldwide Influenza Centre	
EPI1805341	NP	Hong Kong (SAR)	2019-Dec-19	EPI_ISL_574752	C/Hong Kong/272963/2019	Government Virus Unit	Crick Worldwide Influenza Centre	
EPI1805258	HE	Hong Kong (SAR)	2019-Dec-20	EPI_ISL_574753	C/Hong Kong/273848/2019	Government Virus Unit	Crick Worldwide Influenza Centre	
EPI1805299	MP	Hong Kong (SAR)	2019-Dec-20	EPI_ISL_574753	C/Hong Kong/273848/2019	Government Virus Unit	Crick Worldwide Influenza Centre	
EPI1805342	NP	Hong Kong (SAR)	2019-Dec-20	EPI_ISL_574753	C/Hong Kong/273848/2019	Government Virus Unit	Crick Worldwide Influenza Centre	
EPI1805384	NS	Hong Kong (SAR)	2019-Dec-20	EPI_ISL_574753	C/Hong Kong/273848/2019	Government Virus Unit	Crick Worldwide Influenza Centre	
EPI1805418	P3	Hong Kong (SAR)	2019-Dec-20	EPI_ISL_574753	C/Hong Kong/273848/2019	Government Virus Unit	Crick Worldwide Influenza Centre	
EPI1805444	PB1	Hong Kong (SAR)	2019-Dec-20	EPI_ISL_574753	C/Hong Kong/273848/2019	Government Virus Unit	Crick Worldwide Influenza Centre	
EPI1805472	PB2	Hong Kong (SAR)	2019-Dec-20	EPI_ISL_574753	C/Hong Kong/273848/2019	Government Virus Unit	Crick Worldwide Influenza Centre	
EPI1564326	HE	Hong Kong (SAR)	2018-Jan-29	EPI_ISL_380220	C/Hong Kong/27393/2018	Government Virus Unit	Crick Worldwide Influenza Centre	
EPI1595495	MP	Hong Kong (SAR)	2018-Jan-29	EPI_ISL_380220	C/Hong Kong/27393/2018	Government Virus Unit	Crick Worldwide Influenza Centre	
EPI1558329	NP	Hong Kong (SAR)	2018-Jan-29	EPI_ISL_380220	C/Hong Kong/27393/2018	Government Virus Unit	Crick Worldwide Influenza Centre	
EPI1607323	NS	Hong Kong (SAR)	2018-Jan-29	EPI_ISL_380220	C/Hong Kong/27393/2018	Government Virus Unit	Crick Worldwide Influenza Centre	
EPI1566790	P3	Hong Kong (SAR)	2018-Jan-29	EPI_ISL_380220	C/Hong Kong/27393/2018	Government Virus Unit	Crick Worldwide Influenza Centre	
EPI1590432	PB2	Hong Kong (SAR)	2018-Jan-29	EPI_ISL_380220	C/Hong Kong/27393/2018	Government Virus Unit	Crick Worldwide Influenza Centre	
EPI1564327	HE	Hong Kong (SAR)	2018-Jan-29	EPI_ISL_380219	C/Hong Kong/27395/2018	Government Virus Unit	Crick Worldwide Influenza Centre	
EPI1595496	MP	Hong Kong (SAR)	2018-Jan-29	EPI_ISL_380219	C/Hong Kong/27395/2018	Government Virus Unit	Crick Worldwide Influenza Centre	
EPI1558328	NP	Hong Kong (SAR)	2018-Jan-29	EPI_ISL_380219	C/Hong Kong/27395/2018	Government Virus Unit	Crick Worldwide Influenza Centre	
EPI1607380	NS	Hong Kong (SAR)	2018-Jan-29	EPI_ISL_380219	C/Hong Kong/27395/2018	Government Virus Unit	Crick Worldwide Influenza Centre	
EPI1566789	P3	Hong Kong (SAR)	2018-Jan-29	EPI_ISL_380219	C/Hong Kong/27395/2018	Government Virus Unit	Crick Worldwide Influenza Centre	
EPI1590433	PB2	Hong Kong (SAR)	2018-Jan-29	EPI_ISL_380219	C/Hong Kong/27395/2018	Government Virus Unit	Crick Worldwide Influenza Centre	
EPI1805343	NP	Hong Kong (SAR)	2019-Dec-22	EPI_ISL_574776	C/Hong Kong/274109/2019	Government Virus Unit	Crick Worldwide Influenza Centre	
EPI1805385	NS	Hong Kong (SAR)	2019-Dec-22	EPI_ISL_574776	C/Hong Kong/274109/2019	Government Virus Unit	Crick Worldwide Influenza Centre	
EPI1805259	HE	Hong Kong (SAR)	2019-Dec-22	EPI_ISL_574754	C/Hong Kong/275315/2019	Government Virus Unit	Crick Worldwide Influenza Centre	
EPI1805300	MP	Hong Kong (SAR)	2019-Dec-22	EPI_ISL_574754	C/Hong Kong/275315/2019	Government Virus Unit	Crick Worldwide Influenza Centre	
EPI1805344	NP	Hong Kong (SAR)	2019-Dec-22	EPI_ISL_574754	C/Hong Kong/275315/2019	Government Virus Unit	Crick Worldwide Influenza Centre	
EPI1805386	NS	Hong Kong (SAR)	2019-Dec-22	EPI_ISL_574754	C/Hong Kong/275315/2019	Government Virus Unit	Crick Worldwide Influenza Centre	
EPI1805419	P3	Hong Kong (SAR)	2019-Dec-22	EPI_ISL_574754	C/Hong Kong/275315/2019	Government Virus Unit	Crick Worldwide Influenza Centre	
EPI1805445	PB1	Hong Kong (SAR)	2019-Dec-22	EPI_ISL_574754	C/Hong Kong/275315/2019	Government Virus Unit	Crick Worldwide Influenza Centre	
EPI1805473	PB2	Hong Kong (SAR)	2019-Dec-22	EPI_ISL_574754	C/Hong Kong/275315/2019	Government Virus Unit	Crick Worldwide Influenza Centre	
EPI1805260	HE	Hong Kong (SAR)	2019-Dec-23	EPI_ISL_574755	C/Hong Kong/275326/2019	Government Virus Unit	Crick Worldwide Influenza Centre	
EPI1805301	MP	Hong Kong (SAR)	2019-Dec-23	EPI_ISL_574755	C/Hong Kong/275326/2019	Government Virus Unit	Crick Worldwide Influenza Centre	
EPI1805345	NP	Hong Kong (SAR)	2019-Dec-23	EPI_ISL_574755	C/Hong Kong/275326/2019	Government Virus Unit	Crick Worldwide Influenza Centre	
EPI1805387	NS	Hong Kong (SAR)	2019-Dec-23	EPI_ISL_574755	C/Hong Kong/275326/2019	Government Virus Unit	Crick Worldwide Influenza Centre	
EPI1805420	P3	Hong Kong (SAR)	2019-Dec-23	EPI_ISL_574755	C/Hong Kong/275326/2019	Government Virus Unit	Crick Worldwide Influenza Centre	
EPI1805446	PB1	Hong Kong (SAR)	2019-Dec-23	EPI_ISL_574755	C/Hong Kong/275326/2019	Government Virus Unit	Crick Worldwide Influenza Centre	
EPI1805474	PB2	Hong Kong (SAR)	2019-Dec-23	EPI_ISL_574755	C/Hong Kong/275326/2019	Government Virus Unit	Crick Worldwide Influenza Centre	
EPI1805261	HE	Hong Kong (SAR)	2019-Dec-23	EPI_ISL_574756	C/Hong Kong/275370/2019	Government Virus Unit	Crick Worldwide Influenza Centre	
EPI1805302	MP	Hong Kong (SAR)	2019-Dec-23	EPI_ISL_574756	C/Hong Kong/275370/2019	Government Virus Unit	Crick Worldwide Influenza Centre	
EPI1805346	NP	Hong Kong (SAR)	2019-Dec-23	EPI_ISL_574756	C/Hong Kong/275370/2019	Government Virus Unit	Crick Worldwide Influenza Centre	
EPI1805388	NS	Hong Kong (SAR)	2019-Dec-23	EPI_ISL_574756	C/Hong Kong/275370/2019	Government Virus Unit	Crick Worldwide Influenza Centre	
EPI1564328	HE	Hong Kong (SAR)	2018-Jan-29	EPI_ISL_381018	C/Hong Kong/27542/2018	Government Virus Unit	Crick Worldwide Influenza Centre	
EPI1607324	NS	Hong Kong (SAR)	2018-Jan-29	EPI_ISL_381018	C/Hong Kong/27542/2018	Government Virus Unit	Crick Worldwide Influenza Centre	
EPI1564329	HE	Hong Kong (SAR)	2016-Feb-20	EPI_ISL_381019	C/Hong Kong/27579/2016	Government Virus Unit	Crick Worldwide Influenza Centre	
EPI1607325	NS	Hong Kong (SAR)	2016-Feb-20	EPI_ISL_381019	C/Hong Kong/27579/2016	Government Virus Unit	Crick Worldwide Influenza Centre	
EPI1566797	P3	Hong Kong (SAR)	2016-Feb-20	EPI_ISL_381019	C/Hong Kong/27579/2016	Government Virus Unit	Crick Worldwide Influenza Centre	
EPI1567387	PB1	Hong Kong (SAR)	2016-Feb-20	EPI_ISL_381019	C/Hong Kong/27579/2016	Government Virus Unit	Crick Worldwide Influenza Centre	
EPI1590434	PB2	Hong Kong (SAR)	2016-Feb-20	EPI_ISL_381019	C/Hong Kong/27579/2016	Government Virus Unit	Crick Worldwide Influenza Centre	
EPI1805262	HE	Hong Kong (SAR)	2019-Dec-23	EPI_ISL_574757	C/Hong Kong/275952/2019	Government Virus Unit	Crick Worldwide Influenza Centre	
EPI1805303	MP	Hong Kong (SAR)	2019-Dec-23	EPI_ISL_574757	C/Hong Kong/275952/2019	Government Virus Unit	Crick Worldwide Influenza Centre	
EPI1805347	NP	Hong Kong (SAR)	2019-Dec-23	EPI_ISL_574757	C/Hong Kong/275952/2019	Government Virus Unit	Crick Worldwide Influenza Centre	

Segment ID	Segment	Country	Collection date	Isolate-ID	Isolate name	Originating Lab	Submitting Lab	Authors
EPI1805389	NS	Hong Kong (SAR)	2019-Dec-23	EPI_ISL_574757	C/Hong Kong/275952/2019	Government Virus Unit	Crick Worldwide Influenza Centre	
EPI1805421	P3	Hong Kong (SAR)	2019-Dec-23	EPI_ISL_574757	C/Hong Kong/275952/2019	Government Virus Unit	Crick Worldwide Influenza Centre	
EPI1805447	PB1	Hong Kong (SAR)	2019-Dec-23	EPI_ISL_574757	C/Hong Kong/275952/2019	Government Virus Unit	Crick Worldwide Influenza Centre	
EPI1805475	PB2	Hong Kong (SAR)	2019-Dec-23	EPI_ISL_574757	C/Hong Kong/275952/2019	Government Virus Unit	Crick Worldwide Influenza Centre	
EPI1564330	HE	Hong Kong (SAR)	2016-Feb-20	EPI_ISL_380226	C/Hong Kong/27676/2016	Government Virus Unit	Crick Worldwide Influenza Centre	
EPI1595497	MP	Hong Kong (SAR)	2016-Feb-20	EPI_ISL_380226	C/Hong Kong/27676/2016	Government Virus Unit	Crick Worldwide Influenza Centre	
EPI1558335	NP	Hong Kong (SAR)	2016-Feb-20	EPI_ISL_380226	C/Hong Kong/27676/2016	Government Virus Unit	Crick Worldwide Influenza Centre	
EPI1607326	NS	Hong Kong (SAR)	2016-Feb-20	EPI_ISL_380226	C/Hong Kong/27676/2016	Government Virus Unit	Crick Worldwide Influenza Centre	
EPI1566796	P3	Hong Kong (SAR)	2016-Feb-20	EPI_ISL_380226	C/Hong Kong/27676/2016	Government Virus Unit	Crick Worldwide Influenza Centre	
EPI1567386	PB1	Hong Kong (SAR)	2016-Feb-20	EPI_ISL_380226	C/Hong Kong/27676/2016	Government Virus Unit	Crick Worldwide Influenza Centre	
EPI1590435	PB2	Hong Kong (SAR)	2016-Feb-20	EPI_ISL_380226	C/Hong Kong/27676/2016	Government Virus Unit	Crick Worldwide Influenza Centre	
EPI1564331	HE	Hong Kong (SAR)	2018-Jan-29	EPI_ISL_380218	C/Hong Kong/27892/2018	Government Virus Unit	Crick Worldwide Influenza Centre	
EPI1595498	MP	Hong Kong (SAR)	2018-Jan-29	EPI_ISL_380218	C/Hong Kong/27892/2018	Government Virus Unit	Crick Worldwide Influenza Centre	
EPI1558327	NP	Hong Kong (SAR)	2018-Jan-29	EPI_ISL_380218	C/Hong Kong/27892/2018	Government Virus Unit	Crick Worldwide Influenza Centre	
EPI1607327	NS	Hong Kong (SAR)	2018-Jan-29	EPI_ISL_380218	C/Hong Kong/27892/2018	Government Virus Unit	Crick Worldwide Influenza Centre	
EPI1566788	P3	Hong Kong (SAR)	2018-Jan-29	EPI_ISL_380218	C/Hong Kong/27892/2018	Government Virus Unit	Crick Worldwide Influenza Centre	
EPI1590436	PB2	Hong Kong (SAR)	2018-Jan-29	EPI_ISL_380218	C/Hong Kong/27892/2018	Government Virus Unit	Crick Worldwide Influenza Centre	
EPI1564332	HE	Hong Kong (SAR)	2018-Jan-29	EPI_ISL_380217	C/Hong Kong/27990/2018	Government Virus Unit	Crick Worldwide Influenza Centre	
EPI1595499	MP	Hong Kong (SAR)	2018-Jan-29	EPI_ISL_380217	C/Hong Kong/27990/2018	Government Virus Unit	Crick Worldwide Influenza Centre	
EPI1558326	NP	Hong Kong (SAR)	2018-Jan-29	EPI_ISL_380217	C/Hong Kong/27990/2018	Government Virus Unit	Crick Worldwide Influenza Centre	
EPI1607328	NS	Hong Kong (SAR)	2018-Jan-29	EPI_ISL_380217	C/Hong Kong/27990/2018	Government Virus Unit	Crick Worldwide Influenza Centre	
EPI1566787	P3	Hong Kong (SAR)	2018-Jan-29	EPI_ISL_380217	C/Hong Kong/27990/2018	Government Virus Unit	Crick Worldwide Influenza Centre	
EPI1590437	PB2	Hong Kong (SAR)	2018-Jan-29	EPI_ISL_380217	C/Hong Kong/27990/2018	Government Virus Unit	Crick Worldwide Influenza Centre	
EPI1564333	HE	Hong Kong (SAR)	2018-Jan-30	EPI_ISL_381020	C/Hong Kong/28015/2018	Government Virus Unit	Crick Worldwide Influenza Centre	
EPI1595500	MP	Hong Kong (SAR)	2018-Jan-30	EPI_ISL_381020	C/Hong Kong/28015/2018	Government Virus Unit	Crick Worldwide Influenza Centre	
EPI1607329	NS	Hong Kong (SAR)	2018-Jan-30	EPI_ISL_381020	C/Hong Kong/28015/2018	Government Virus Unit	Crick Worldwide Influenza Centre	
EPI1805263	HE	Hong Kong (SAR)	2019-Dec-29	EPI_ISL_574758	C/Hong Kong/280339/2019	Government Virus Unit	Crick Worldwide Influenza Centre	
EPI1805304	MP	Hong Kong (SAR)	2019-Dec-29	EPI_ISL_574758	C/Hong Kong/280339/2019	Government Virus Unit	Crick Worldwide Influenza Centre	
EPI1805348	NP	Hong Kong (SAR)	2019-Dec-29	EPI_ISL_574758	C/Hong Kong/280339/2019	Government Virus Unit	Crick Worldwide Influenza Centre	
EPI1805390	NS	Hong Kong (SAR)	2019-Dec-29	EPI_ISL_574758	C/Hong Kong/280339/2019	Government Virus Unit	Crick Worldwide Influenza Centre	
EPI1805422	P3	Hong Kong (SAR)	2019-Dec-29	EPI_ISL_574758	C/Hong Kong/280339/2019	Government Virus Unit	Crick Worldwide Influenza Centre	
EPI1805448	PB1	Hong Kong (SAR)	2019-Dec-29	EPI_ISL_574758	C/Hong Kong/280339/2019	Government Virus Unit	Crick Worldwide Influenza Centre	
EPI1805476	PB2	Hong Kong (SAR)	2019-Dec-29	EPI_ISL_574758	C/Hong Kong/280339/2019	Government Virus Unit	Crick Worldwide Influenza Centre	
EPI1805264	HE	Hong Kong (SAR)	2019-Dec-29	EPI_ISL_574759	C/Hong Kong/280457/2019	Government Virus Unit	Crick Worldwide Influenza Centre	
EPI1805305	MP	Hong Kong (SAR)	2019-Dec-29	EPI_ISL_574759	C/Hong Kong/280457/2019	Government Virus Unit	Crick Worldwide Influenza Centre	
EPI1805349	NP	Hong Kong (SAR)	2019-Dec-29	EPI_ISL_574759	C/Hong Kong/280457/2019	Government Virus Unit	Crick Worldwide Influenza Centre	
EPI1805265	HE	Hong Kong (SAR)	2019-Dec-29	EPI_ISL_574760	C/Hong Kong/280832/2019	Government Virus Unit	Crick Worldwide Influenza Centre	
EPI1805306	MP	Hong Kong (SAR)	2019-Dec-29	EPI_ISL_574760	C/Hong Kong/280832/2019	Government Virus Unit	Crick Worldwide Influenza Centre	
EPI1805350	NP	Hong Kong (SAR)	2019-Dec-29	EPI_ISL_574760	C/Hong Kong/280832/2019	Government Virus Unit	Crick Worldwide Influenza Centre	
EPI1805391	NS	Hong Kong (SAR)	2019-Dec-29	EPI_ISL_574760	C/Hong Kong/280832/2019	Government Virus Unit	Crick Worldwide Influenza Centre	
EPI1805423	P3	Hong Kong (SAR)	2019-Dec-29	EPI_ISL_574760	C/Hong Kong/280832/2019	Government Virus Unit	Crick Worldwide Influenza Centre	
EPI1805449	PB1	Hong Kong (SAR)	2019-Dec-29	EPI_ISL_574760	C/Hong Kong/280832/2019	Government Virus Unit	Crick Worldwide Influenza Centre	
EPI1805477	PB2	Hong Kong (SAR)	2019-Dec-29	EPI_ISL_574760	C/Hong Kong/280832/2019	Government Virus Unit	Crick Worldwide Influenza Centre	
EPI1805266	HE	Hong Kong (SAR)	2019-Dec-31	EPI_ISL_574761	C/Hong Kong/281471/2019	Government Virus Unit	Crick Worldwide Influenza Centre	
EPI1805307	MP	Hong Kong (SAR)	2019-Dec-31	EPI_ISL_574761	C/Hong Kong/281471/2019	Government Virus Unit	Crick Worldwide Influenza Centre	
EPI1805351	NP	Hong Kong (SAR)	2019-Dec-31	EPI_ISL_574761	C/Hong Kong/281471/2019	Government Virus Unit	Crick Worldwide Influenza Centre	
EPI1805392	NS	Hong Kong (SAR)	2019-Dec-31	EPI_ISL_574761	C/Hong Kong/281471/2019	Government Virus Unit	Crick Worldwide Influenza Centre	
EPI1805424	P3	Hong Kong (SAR)	2019-Dec-31	EPI_ISL_574761	C/Hong Kong/281471/2019	Government Virus Unit	Crick Worldwide Influenza Centre	
EPI1805450	PB1	Hong Kong (SAR)	2019-Dec-31	EPI_ISL_574761	C/Hong Kong/281471/2019	Government Virus Unit	Crick Worldwide Influenza Centre	
EPI1805478	PB2	Hong Kong (SAR)	2019-Dec-31	EPI_ISL_574761	C/Hong Kong/281471/2019	Government Virus Unit	Crick Worldwide Influenza Centre	
EPI1564334	HE	Hong Kong (SAR)	2016-Feb-20	EPI_ISL_381021	C/Hong Kong/28182/2016	Government Virus Unit	Crick Worldwide Influenza Centre	
EPI1607330	NS	Hong Kong (SAR)	2016-Feb-20	EPI_ISL_381021	C/Hong Kong/28182/2016	Government Virus Unit	Crick Worldwide Influenza Centre	
EPI1564335	HE	Hong Kong (SAR)	2016-Jan-06	EPI_ISL_381022	C/Hong Kong/2879/2016	Government Virus Unit	Crick Worldwide Influenza Centre	
EPI1595501	MP	Hong Kong (SAR)	2016-Jan-06	EPI_ISL_381022	C/Hong Kong/2879/2016	Government Virus Unit	Crick Worldwide Influenza Centre	
EPI1607331	NS	Hong Kong (SAR)	2016-Jan-06	EPI_ISL_381022	C/Hong Kong/2879/2016	Government Virus Unit	Crick Worldwide Influenza Centre	
EPI1805267	HE	Hong Kong (SAR)	2020-Feb-03	EPI_ISL_574762	C/Hong Kong/28937/2020	Government Virus Unit	Crick Worldwide Influenza Centre	
EPI1805308	MP	Hong Kong (SAR)	2020-Feb-03	EPI_ISL_574762	C/Hong Kong/28937/2020	Government Virus Unit	Crick Worldwide Influenza Centre	
EPI1805352	NP	Hong Kong (SAR)	2020-Feb-03	EPI_ISL_574762	C/Hong Kong/28937/2020	Government Virus Unit	Crick Worldwide Influenza Centre	
EPI1805393	NS	Hong Kong (SAR)	2020-Feb-03	EPI_ISL_574762	C/Hong Kong/28937/2020	Government Virus Unit	Crick Worldwide Influenza Centre	
EPI1805425	P3	Hong Kong (SAR)	2020-Feb-03	EPI_ISL_574762	C/Hong Kong/28937/2020	Government Virus Unit	Crick Worldwide Influenza Centre	
EPI1805451	PB1	Hong Kong (SAR)	2020-Feb-03	EPI_ISL_574762	C/Hong Kong/28937/2020	Government Virus Unit	Crick Worldwide Influenza Centre	
EPI1805479	PB2	Hong Kong (SAR)	2020-Feb-03	EPI_ISL_574762	C/Hong Kong/28937/2020	Government Virus Unit	Crick Worldwide Influenza Centre	
EPI1564337	HE	Hong Kong (SAR)	2016-Jan-07	EPI_ISL_380243	C/Hong Kong/2957/2016	Government Virus Unit	Crick Worldwide Influenza Centre	
EPI1595502	MP	Hong Kong (SAR)	2016-Jan-07	EPI_ISL_380243	C/Hong Kong/2957/2016	Government Virus Unit	Crick Worldwide Influenza Centre	
EPI1558352	NP	Hong Kong (SAR)	2016-Jan-07	EPI_ISL_380243	C/Hong Kong/2957/2016	Government Virus Unit	Crick Worldwide Influenza Centre	
EPI1607381	NS	Hong Kong (SAR)	2016-Jan-07	EPI_ISL_380243	C/Hong Kong/2957/2016	Government Virus Unit	Crick Worldwide Influenza Centre	
EPI1566804	P3	Hong Kong (SAR)	2016-Jan-07	EPI_ISL_380243	C/Hong Kong/2957/2016	Government Virus Unit	Crick Worldwide Influenza Centre	
EPI1567398	PB1	Hong Kong (SAR)	2016-Jan-07	EPI_ISL_380243	C/Hong Kong/2957/2016	Government Virus Unit	Crick Worldwide Influenza Centre	
EPI1590438	PB2	Hong Kong (SAR)	2016-Jan-07	EPI_ISL_380243	C/Hong Kong/2957/2016	Government Virus Unit	Crick Worldwide Influenza Centre	
EPI1564338	HE	Hong Kong (SAR)	2018-Feb-01	EPI_ISL_381023	C/Hong Kong/30248/2018	Government Virus Unit	Crick Worldwide Influenza Centre	
EPI1595504	MP	Hong Kong (SAR)	2018-Feb-01	EPI_ISL_381023	C/Hong Kong/30248/2018	Government Virus Unit	Crick Worldwide Influenza Centre	
EPI1607333	NS	Hong Kong (SAR)	2018-Feb-01	EPI_ISL_381023	C/Hong Kong/30248/2018	Government Virus Unit	Crick Worldwide Influenza Centre	
EPI1805268	HE	Hong Kong (SAR)	2020-Feb-05	EPI_ISL_574763	C/Hong Kong/30592/2020	Government Virus Unit	Crick Worldwide Influenza Centre	
EPI1805309	MP	Hong Kong (SAR)	2020-Feb-05	EPI_ISL_574763	C/Hong Kong/30592/2020	Government Virus Unit	Crick Worldwide Influenza Centre	
EPI1805353	NP	Hong Kong (SAR)	2020-Feb-05	EPI_ISL_574763	C/Hong Kong/30592/2020	Government Virus Unit	Crick Worldwide Influenza Centre	
EPI1805394	NS	Hong Kong (SAR)	2020-Feb-05	EPI_ISL_574763	C/Hong Kong/30592/2020	Government Virus Unit	Crick Worldwide Influenza Centre	
EPI1805426	P3	Hong Kong (SAR)	2020-Feb-05	EPI_ISL_574763	C/Hong Kong/30592/2020	Government Virus Unit	Crick Worldwide Influenza Centre	
EPI1805452	PB1	Hong Kong (SAR)	2020-Feb-05	EPI_ISL_574763	C/Hong Kong/30592/2020	Government Virus Unit	Crick Worldwide Influenza Centre	
EPI1805480	PB2	Hong Kong (SAR)	2020-Feb-05	EPI_ISL_574763	C/Hong Kong/30592/2020	Government Virus Unit	Crick Worldwide Influenza Centre	
EPI1805269	HE	Hong Kong (SAR)	2020-Jan-03	EPI_ISL_574764	C/Hong Kong/3124/2020	Government Virus Unit	Crick Worldwide Influenza Centre	
EPI1805310	MP	Hong Kong (SAR)	2020-Jan-03	EPI_ISL_574764	C/Hong Kong/3124/2020	Government Virus Unit	Crick Worldwide Influenza Centre	
EPI1805354	NP	Hong Kong (SAR)	2020-Jan-03	EPI_ISL_574764	C/Hong Kong/3124/2020	Government Virus Unit	Crick Worldwide Influenza Centre	
EPI1805395	NS	Hong Kong (SAR)	2020-Jan-03	EPI_ISL_574764	C/Hong Kong/3124/2020	Government Virus Unit	Crick Worldwide Influenza Centre	
EPI1805427	P3	Hong Kong (SAR)	2020-Jan-03	EPI_ISL_574764	C/Hong Kong/3124/2020	Government Virus Unit	Crick Worldwide Influenza Centre	
EPI1805453	PB1	Hong Kong (SAR)	2020-Jan-03	EPI_ISL_574764	C/Hong Kong/3124/2020	Government Virus Unit	Crick Worldwide Influenza Centre	
EPI1805481	PB2	Hong Kong (SAR)	2020-Jan-03	EPI_ISL_574764	C/Hong Kong/3124/2020	Government Virus Unit	Crick Worldwide Influenza Centre	
EPI1805311	MP	Hong Kong (SAR)	2020-Jan-05	EPI_ISL_574773	C/Hong Kong/3183/2020	Government Virus Unit	Crick Worldwide Influenza Centre	
EPI1805355	NP	Hong Kong (SAR)	2020-Jan-05	EPI_ISL_574773	C/Hong Kong/3183/2020	Government Virus Unit	Crick Worldwide Influenza Centre	
EPI1805396	NS	Hong Kong (SAR)	2020-Jan-05	EPI_ISL_574773	C/Hong Kong/3183/2020	Government Virus Unit	Crick Worldwide Influenza Centre	
EPI1805270	HE	Hong Kong (SAR)	2020-Feb-08	EPI_ISL_574765	C/Hong Kong/32464/2020	Government Virus Unit	Crick Worldwide Influenza Centre	
EPI1805312	MP	Hong Kong (SAR)	2020-Feb-08	EPI_ISL_574765	C/Hong Kong/32464/2020	Government Virus Unit	Crick Worldwide Influenza Centre	
EPI1805356	NP	Hong Kong (SAR)	2020-Feb-08	EPI_ISL_574765	C/Hong Kong/32464/2020	Government Virus Unit	Crick Worldwide Influenza Centre	
EPI1805397	NS	Hong Kong (SAR)	2020-Feb-08	EPI_ISL_574765	C/Hong Kong/32464/2020	Government Virus Unit	Crick Worldwide Influenza Centre	
EPI1805428	P3	Hong Kong (SAR)	2020-Feb-08	EPI_ISL_574765	C/Hong Kong/32464/2020	Government Virus Unit	Crick Worldwide Influenza Centre	
EPI1805454	PB1	Hong Kong (SAR)	2020-Feb-08	EPI_ISL_574765	C/Hong Kong/32464/2020	Government Virus Unit	Crick Worldwide Influenza Centre	
EPI1805482	PB2	Hong Kong (SAR)	2020-Feb-08	EPI_ISL_574765	C/Hong Kong/32464/2020	Government Virus Unit	Crick Worldwide Influenza Centre	
EPI1564339	HE	Hong Kong (SAR)	2018-Jan-03	EPI_ISL_380225	C/Hong Kong/3286/2018	Government Virus Unit	Crick Worldwide Influenza Centre	
EPI1595505	MP	Hong Kong (SAR)	2018-Jan-03	EPI_ISL_380225	C/Hong Kong/3286/2018	Government Virus Unit	Crick Worldwide Influenza Centre	
EPI1558334	NP	Hong Kong (SAR)	2018-Jan-03	EPI_ISL_380225	C/Hong Kong/3286/2018	Government Virus Unit	Crick Worldwide Influenza Centre	
EPI1607334	NS	Hong Kong (SAR)	2018-Jan-03	EPI_ISL_380225	C/Hong Kong/3286/2018	Government Virus Unit	Crick Worldwide Influenza Centre	
EPI1564340	HE	Hong Kong (SAR)	2018-Feb-04	EPI_ISL_381024	C/Hong Kong/33189/2018	Government Virus Unit	Crick Worldwide Influenza Centre	
EPI1595506	MP	Hong Kong (SAR)	2018-Feb-04	EPI_ISL_381024	C/Hong Kong/33189/2018	Government Virus Unit	Crick Worldwide Influenza Centre	
EPI1607335	NS	Hong Kong (SAR)	2018-Feb-04	EPI_ISL_381024	C/Hong Kong/33189/2018	Government Virus Unit	Crick Worldwide Influenza Centre	
EPI1564341	HE	Hong Kong (SAR)	2018-Feb-04	EPI_ISL_380216	C/Hong Kong/33417/2018	Government Virus Unit	Crick Worldwide Influenza Centre	
EPI1595507	MP	Hong Kong (SAR)	2018-Feb-04	EPI_ISL_380216	C/Hong Kong/33417/2018	Government Virus Unit	Crick Worldwide Influenza Centre	
EPI1558325	NP	Hong Kong (SAR)	2018-Feb-04	EPI_ISL_380216	C/Hong Kong/33417/2018	Government Virus Unit	Crick Worldwide Influenza Centre	
EPI1607336	NS	Hong Kong (SAR)	2018-Feb-04	EPI_ISL_380216	C/Hong Kong/33417/2018	Government Virus Unit	Crick Worldwide Influenza Centre	
EPI1566786	P3	Hong Kong (SAR)	2018-Feb-04	EPI_ISL_380216	C/Hong Kong/33417/2018	Government Virus Unit	Crick Worldwide Influenza Centre	
EPI1567383	PB1	Hong Kong (SAR)	2018-Feb-04	EPI_ISL_380216	C/Hong Kong/33417/2018	Government Virus Unit	Crick Worldwide Influenza Centre	
EPI1590440	PB2	Hong Kong (SAR)	2018-Feb-04	EPI_ISL_380216	C/Hong Kong/33417/2018	Government Virus Unit	Crick Worldwide Influenza Centre	
EPI1564342	HE	Hong Kong (SAR)	2018-Feb-04	EPI_ISL_380215	C/Hong Kong/33535/2018	Government Virus Unit	Crick Worldwide Influenza Centre	
EPI1595508	MP	Hong Kong (SAR)	2018-Feb-04	EPI_ISL_380215	C/Hong Kong/33535/2018	Government Virus Unit	Crick Worldwide Influenza Centre	
EPI1558324	NP	Hong Kong (SAR)	2018-Feb-04	EPI_ISL_380215	C/Hong Kong/33535/2018	Government Virus Unit	Crick Worldwide Influenza Centre	
EPI1607337	NS	Hong Kong (SAR)	2018-Feb-04	EPI_ISL_380215	C/Hong Kong/33535/2018	Government Virus Unit	Crick Worldwide Influenza Centre	
EPI1566785	P3	Hong Kong (SAR)	2018-Feb-04	EPI_ISL_380215	C/Hong Kong/33535/2018	Government Virus Unit	Crick Worldwide Influenza Centre	
EPI1567382	PB1	Hong Kong (SAR)	2018-Feb-04	EPI_ISL_380215	C/Hong Kong/33535/2018	Government Virus Unit	Crick Worldwide Influenza Centre	
EPI1590441	PB2	Hong Kong (SAR)	2018-Feb-04	EPI_ISL_380215	C/Hong Kong/33535/2018	Government Virus Unit	Crick Worldwide Influenza Centre	
EPI1564343	HE	Hong Kong (SAR)	2018-Jan-03	EPI_ISL_380224	C/Hong Kong/3363/2018	Government Virus Unit	Crick Worldwide Influenza Centre	
EPI1595509	MP	Hong Kong (SAR)	2018-Jan-03	EPI_ISL_380224	C/Hong Kong/3363/2018	Government Virus Unit	Crick Worldwide Influenza Centre	
EPI1558333	NP	Hong Kong (SAR)	2018-Jan-03	EPI_ISL_380224	C/Hong Kong/3363/2018	Government Virus Unit	Crick Worldwide Influenza Centre	
EPI1607338	NS	Hong Kong (SAR)	2018-Jan-03	EPI_ISL_380224	C/Hong Kong/3363/2018	Government Virus Unit	Crick Worldwide Influenza Centre	
EPI1566795	P3	Hong Kong (SAR)	2018-Jan-03	EPI_ISL_380224	C/Hong Kong/3363/2018	Government Virus Unit	Crick Worldwide Influenza Centre	
EPI1567385	PB1	Hong Kong (SAR)	2018-Jan-03	EPI_ISL_380224	C/Hong Kong/3363/2018	Government Virus Unit	Crick Worldwide Influenza Centre	
EPI1590442	PB2	Hong Kong (SAR)	2018-Jan-03	EPI_ISL_380224	C/Hong Kong/3363/2018	Government Virus Unit	Crick Worldwide Influenza Centre	
EPI1564344	HE	Hong Kong (SAR)	2016-Jan-08	EPI_ISL_380242	C/Hong Kong/3586/2016	Government Virus Unit	Crick Worldwide Influenza Centre	
EPI1595510	MP	Hong Kong (SAR)	2016-Jan-08	EPI_ISL_380242	C/Hong Kong/3586/2016	Government Virus Unit	Crick Worldwide Influenza Centre	
EPI1558351	NP	Hong Kong (SAR)	2016-Jan-08	EPI_ISL_380242	C/Hong Kong/3586/2016	Government Virus Unit	Crick Worldwide Influenza Centre	
EPI1607382	NS	Hong Kong (SAR)	2016-Jan-08	EPI_ISL_380242	C/Hong Kong/3586/2016	Government Virus Unit	Crick Worldwide Influenza Centre	
EPI1566803	P3	Hong Kong (SAR)	2016-Jan-08	EPI_ISL_380242	C/Hong Kong/3586/2016	Government Virus Unit	Crick Worldwide Influenza Centre	
EPI1567397	PB1	Hong Kong (SAR)	2016-Jan-08	EPI_ISL_380242	C/Hong Kong/3586/2016	Government Virus Unit	Crick Worldwide Influenza Centre	
EPI1590443	PB2	Hong Kong (SAR)	2016-Jan-08	EPI_ISL_380242	C/Hong Kong/3586/2016	Government Virus Unit	Crick Worldwide Influenza Centre	
EPI1805271	HE	Hong Kong (SAR)	2020-Feb-12	EPI_ISL_574766	C/Hong Kong/35991/2020	Government Virus Unit	Crick Worldwide Influenza Centre	
EPI1805313	MP	Hong Kong (SAR)	2020-Feb-12	EPI_ISL_574766	C/Hong Kong/35991/2020	Government Virus Unit	Crick Worldwide Influenza Centre	
EPI1805398	NS	Hong Kong (SAR)	2020-Feb-12	EPI_ISL_574766	C/Hong Kong/35991/2020	Government Virus Unit	Crick Worldwide Influenza Centre	
EPI1805429	P3	Hong Kong (SAR)	2020-Feb-12	EPI_ISL_574766	C/Hong Kong/35991/2020	Government Virus Unit	Crick Worldwide Influenza Centre	
EPI1805455	PB1	Hong Kong (SAR)	2020-Feb-12	EPI_ISL_574766	C/Hong Kong/35991/2020	Government Virus Unit	Crick Worldwide Influenza Centre	
EPI1805483	PB2	Hong Kong (SAR)	2020-Feb-12	EPI_ISL_574766	C/Hong Kong/35991/2020	Government Virus Unit	Crick Worldwide Influenza Centre	
EPI1805314	MP	Hong Kong (SAR)	2020-Jan-02	EPI_ISL_574774	C/Hong Kong/370/2020	Government Virus Unit	Crick Worldwide Influenza Centre	
EPI1564346	HE	Hong Kong (SAR)	2018-Feb-07	EPI_ISL_380213	C/Hong Kong/37128/2018	Government Virus Unit	Crick Worldwide Influenza Centre	
EPI1595512	MP	Hong Kong (SAR)	2018-Feb-07	EPI_ISL_380213	C/Hong Kong/37128/2018	Government Virus Unit	Crick Worldwide Influenza Centre	
EPI1558322	NP	Hong Kong (SAR)	2018-Feb-07	EPI_ISL_380213	C/Hong Kong/37128/2018	Government Virus Unit	Crick Worldwide Influenza Centre	
EPI1607340	NS	Hong Kong (SAR)	2018-Feb-07	EPI_ISL_380213	C/Hong Kong/37128/2018	Government Virus Unit	Crick Worldwide Influenza Centre	
EPI1566783	P3	Hong Kong (SAR)	2018-Feb-07	EPI_ISL_380213	C/Hong Kong/37128/2018	Government Virus Unit	Crick Worldwide Influenza Centre	
EPI1567380	PB1	Hong Kong (SAR)	2018-Feb-07	EPI_ISL_380213	C/Hong Kong/37128/2018	Government Virus Unit	Crick Worldwide Influenza Centre	
EPI1590444	PB2	Hong Kong (SAR)	2018-Feb-07	EPI_ISL_380213	C/Hong Kong/37128/2018	Government Virus Unit	Crick Worldwide Influenza Centre	
EPI1607341	NS	Hong Kong (SAR)	2018-Feb-10	EPI_ISL_395061	C/Hong Kong/40232/2018	Government Virus Unit	Crick Worldwide Influenza Centre	
EPI1564347	HE	Hong Kong (SAR)	2018-Feb-09	EPI_ISL_380212	C/Hong Kong/40626/2018	Government Virus Unit	Crick Worldwide Influenza Centre	
EPI1595513	MP	Hong Kong (SAR)	2018-Feb-09	EPI_ISL_380212	C/Hong Kong/40626/2018	Government Virus Unit	Crick Worldwide Influenza Centre	
EPI1558321	NP	Hong Kong (SAR)	2018-Feb-09	EPI_ISL_380212	C/Hong Kong/40626/2018	Government Virus Unit	Crick Worldwide Influenza Centre	
EPI1607342	NS	Hong Kong (SAR)	2018-Feb-09	EPI_ISL_380212	C/Hong Kong/40626/2018	Government Virus Unit	Crick Worldwide Influenza Centre	
EPI1566782	P3	Hong Kong (SAR)	2018-Feb-09	EPI_ISL_380212	C/Hong Kong/40626/2018	Government Virus Unit	Crick Worldwide Influenza Centre	
EPI1564348	HE	Hong Kong (SAR)	2018-Feb-14	EPI_ISL_380210	C/Hong Kong/45913/2018	Government Virus Unit	Crick Worldwide Influenza Centre	
EPI1595514	MP	Hong Kong (SAR)	2018-Feb-14	EPI_ISL_380210	C/Hong Kong/45913/2018	Government Virus Unit	Crick Worldwide Influenza Centre	
EPI1558319	NP	Hong Kong (SAR)	2018-Feb-14	EPI_ISL_380210	C/Hong Kong/45913/2018	Government Virus Unit	Crick Worldwide Influenza Centre	
EPI1607343	NS	Hong Kong (SAR)	2018-Feb-14	EPI_ISL_380210	C/Hong Kong/45913/2018	Government Virus Unit	Crick Worldwide Influenza Centre	
EPI1566781	P3	Hong Kong (SAR)	2018-Feb-14	EPI_ISL_380210	C/Hong Kong/45913/2018	Government Virus Unit	Crick Worldwide Influenza Centre	
EPI1590446	PB2	Hong Kong (SAR)	2018-Feb-14	EPI_ISL_380210	C/Hong Kong/45913/2018	Government Virus Unit	Crick Worldwide Influenza Centre	
EPI1564349	HE	Hong Kong (SAR)	2018-Feb-13	EPI_ISL_380211	C/Hong Kong/46081/2018	Government Virus Unit	Crick Worldwide Influenza Centre	
EPI1558320	NP	Hong Kong (SAR)	2018-Feb-13	EPI_ISL_380211	C/Hong Kong/46081/2018	Government Virus Unit	Crick Worldwide Influenza Centre	
EPI1607344	NS	Hong Kong (SAR)	2018-Feb-13	EPI_ISL_380211	C/Hong Kong/46081/2018	Government Virus Unit	Crick Worldwide Influenza Centre	
EPI1564350	HE	Hong Kong (SAR)	2018-Feb-17	EPI_ISL_381025	C/Hong Kong/47144/2018	Government Virus Unit	Crick Worldwide Influenza Centre	
EPI1595515	MP	Hong Kong (SAR)	2018-Feb-17	EPI_ISL_381025	C/Hong Kong/47144/2018	Government Virus Unit	Crick Worldwide Influenza Centre	
EPI1607345	NS	Hong Kong (SAR)	2018-Feb-17	EPI_ISL_381025	C/Hong Kong/47144/2018	Government Virus Unit	Crick Worldwide Influenza Centre	
EPI1564351	HE	Hong Kong (SAR)	2018-Feb-16	EPI_ISL_381026	C/Hong Kong/47455/2018	Government Virus Unit	Crick Worldwide Influenza Centre	
EPI1595516	MP	Hong Kong (SAR)	2018-Feb-16	EPI_ISL_381026	C/Hong Kong/47455/2018	Government Virus Unit	Crick Worldwide Influenza Centre	
EPI1607346	NS	Hong Kong (SAR)	2018-Feb-16	EPI_ISL_381026	C/Hong Kong/47455/2018	Government Virus Unit	Crick Worldwide Influenza Centre	
EPI1805272	HE	Hong Kong (SAR)	2020-Jan-06	EPI_ISL_574767	C/Hong Kong/5029/2020	Government Virus Unit	Crick Worldwide Influenza Centre	
EPI1805315	MP	Hong Kong (SAR)	2020-Jan-06	EPI_ISL_574767	C/Hong Kong/5029/2020	Government Virus Unit	Crick Worldwide Influenza Centre	
EPI1805357	NP	Hong Kong (SAR)	2020-Jan-06	EPI_ISL_574767	C/Hong Kong/5029/2020	Government Virus Unit	Crick Worldwide Influenza Centre	
EPI1805399	NS	Hong Kong (SAR)	2020-Jan-06	EPI_ISL_574767	C/Hong Kong/5029/2020	Government Virus Unit	Crick Worldwide Influenza Centre	
EPI1805430	P3	Hong Kong (SAR)	2020-Jan-06	EPI_ISL_574767	C/Hong Kong/5029/2020	Government Virus Unit	Crick Worldwide Influenza Centre	
EPI1805456	PB1	Hong Kong (SAR)	2020-Jan-06	EPI_ISL_574767	C/Hong Kong/5029/2020	Government Virus Unit	Crick Worldwide Influenza Centre	
EPI1805484	PB2	Hong Kong (SAR)	2020-Jan-06	EPI_ISL_574767	C/Hong Kong/5029/2020	Government Virus Unit	Crick Worldwide Influenza Centre	
EPI1564352	HE	Hong Kong (SAR)	2018-Feb-18	EPI_ISL_381027	C/Hong Kong/50551/2018	Government Virus Unit	Crick Worldwide Influenza Centre	
EPI1607347	NS	Hong Kong (SAR)	2018-Feb-18	EPI_ISL_381027	C/Hong Kong/50551/2018	Government Virus Unit	Crick Worldwide Influenza Centre	
EPI1564353	HE	Hong Kong (SAR)	2018-Feb-19	EPI_ISL_381028	C/Hong Kong/50752/2018	Government Virus Unit	Crick Worldwide Influenza Centre	

Segment ID	Segment	Country	Collection date	Isolate-ID	Isolate name	Originating Lab	Submitting Lab	Authors
EPI1607348	NS	Hong Kong (SAR)	2018-Feb-19	EPI_ISL_381028	C/Hong Kong/50752/2018	Government Virus Unit	Crick Worldwide Influenza Centre	
EPI1595517	MP	Hong Kong (SAR)	2018-Feb-25	EPI_ISL_391812	C/Hong Kong/57069/2018	Government Virus Unit	Crick Worldwide Influenza Centre	
EPI1607349	NS	Hong Kong (SAR)	2018-Feb-25	EPI_ISL_391812	C/Hong Kong/57069/2018	Government Virus Unit	Crick Worldwide Influenza Centre	
EPI1564354	HE	Hong Kong (SAR)	2018-Feb-26	EPI_ISL_381029	C/Hong Kong/57791/2018	Government Virus Unit	Crick Worldwide Influenza Centre	
EPI1595518	MP	Hong Kong (SAR)	2018-Feb-26	EPI_ISL_381029	C/Hong Kong/57791/2018	Government Virus Unit	Crick Worldwide Influenza Centre	
EPI1607350	NS	Hong Kong (SAR)	2018-Feb-26	EPI_ISL_381029	C/Hong Kong/57791/2018	Government Virus Unit	Crick Worldwide Influenza Centre	
EPI1564355	HE	Hong Kong (SAR)	2018-Feb-24	EPI_ISL_381030	C/Hong Kong/58158/2018	Government Virus Unit	Crick Worldwide Influenza Centre	
EPI1607351	NS	Hong Kong (SAR)	2018-Feb-24	EPI_ISL_381030	C/Hong Kong/58158/2018	Government Virus Unit	Crick Worldwide Influenza Centre	
EPI1564374	HE	Hong Kong (SAR)	2008-Jan-01	EPI_ISL_381036	C/Hong Kong/6016/2008	Government Virus Unit	Crick Worldwide Influenza Centre	
EPI1595519	MP	Hong Kong (SAR)	2008-Jan-01	EPI_ISL_381036	C/Hong Kong/6016/2008	Government Virus Unit	Crick Worldwide Influenza Centre	
EPI1564381	NP	Hong Kong (SAR)	2008-Jan-01	EPI_ISL_381036	C/Hong Kong/6016/2008	Government Virus Unit	Crick Worldwide Influenza Centre	
EPI1607352	NS	Hong Kong (SAR)	2008-Jan-01	EPI_ISL_381036	C/Hong Kong/6016/2008	Government Virus Unit	Crick Worldwide Influenza Centre	
EPI1566809	P3	Hong Kong (SAR)	2008-Jan-01	EPI_ISL_381036	C/Hong Kong/6016/2008	Government Virus Unit	Crick Worldwide Influenza Centre	
EPI1567393	PB1	Hong Kong (SAR)	2008-Jan-01	EPI_ISL_381036	C/Hong Kong/6016/2008	Government Virus Unit	Crick Worldwide Influenza Centre	
EPI1590447	PB2	Hong Kong (SAR)	2008-Jan-01	EPI_ISL_381036	C/Hong Kong/6016/2008	Government Virus Unit	Crick Worldwide Influenza Centre	
EPI1564356	HE	Hong Kong (SAR)	2018-Feb-28	EPI_ISL_380214	C/Hong Kong/60430/2018	Government Virus Unit	Crick Worldwide Influenza Centre	
EPI1595520	MP	Hong Kong (SAR)	2018-Feb-28	EPI_ISL_380214	C/Hong Kong/60430/2018	Government Virus Unit	Crick Worldwide Influenza Centre	
EPI1558323	NP	Hong Kong (SAR)	2018-Feb-28	EPI_ISL_380214	C/Hong Kong/60430/2018	Government Virus Unit	Crick Worldwide Influenza Centre	
EPI1607354	NS	Hong Kong (SAR)	2018-Feb-28	EPI_ISL_380214	C/Hong Kong/60430/2018	Government Virus Unit	Crick Worldwide Influenza Centre	
EPI1566784	P3	Hong Kong (SAR)	2018-Feb-28	EPI_ISL_380214	C/Hong Kong/60430/2018	Government Virus Unit	Crick Worldwide Influenza Centre	
EPI1567381	PB1	Hong Kong (SAR)	2018-Feb-28	EPI_ISL_380214	C/Hong Kong/60430/2018	Government Virus Unit	Crick Worldwide Influenza Centre	
EPI1590449	PB2	Hong Kong (SAR)	2018-Feb-28	EPI_ISL_380214	C/Hong Kong/60430/2018	Government Virus Unit	Crick Worldwide Influenza Centre	
EPI1564358	HE	Hong Kong (SAR)	2016-Jan-14	EPI_ISL_381031	C/Hong Kong/6091/2016	Government Virus Unit	Crick Worldwide Influenza Centre	
EPI1595522	MP	Hong Kong (SAR)	2016-Jan-14	EPI_ISL_381031	C/Hong Kong/6091/2016	Government Virus Unit	Crick Worldwide Influenza Centre	
EPI1607384	NS	Hong Kong (SAR)	2016-Jan-14	EPI_ISL_381031	C/Hong Kong/6091/2016	Government Virus Unit	Crick Worldwide Influenza Centre	
EPI1564360	HE	Hong Kong (SAR)	2016-Jan-14	EPI_ISL_380241	C/Hong Kong/6451/2016	Government Virus Unit	Crick Worldwide Influenza Centre	
EPI1595523	MP	Hong Kong (SAR)	2016-Jan-14	EPI_ISL_380241	C/Hong Kong/6451/2016	Government Virus Unit	Crick Worldwide Influenza Centre	
EPI1558350	NP	Hong Kong (SAR)	2016-Jan-14	EPI_ISL_380241	C/Hong Kong/6451/2016	Government Virus Unit	Crick Worldwide Influenza Centre	
EPI1607385	NS	Hong Kong (SAR)	2016-Jan-14	EPI_ISL_380241	C/Hong Kong/6451/2016	Government Virus Unit	Crick Worldwide Influenza Centre	
EPI1566802	P3	Hong Kong (SAR)	2016-Jan-14	EPI_ISL_380241	C/Hong Kong/6451/2016	Government Virus Unit	Crick Worldwide Influenza Centre	
EPI1567396	PB1	Hong Kong (SAR)	2016-Jan-14	EPI_ISL_380241	C/Hong Kong/6451/2016	Government Virus Unit	Crick Worldwide Influenza Centre	
EPI1590450	PB2	Hong Kong (SAR)	2016-Jan-14	EPI_ISL_380241	C/Hong Kong/6451/2016	Government Virus Unit	Crick Worldwide Influenza Centre	
EPI1564375	HE	Hong Kong (SAR)	2009-Jan-01	EPI_ISL_380254	C/Hong Kong/6483/2009	Government Virus Unit	Crick Worldwide Influenza Centre	
EPI1595525	MP	Hong Kong (SAR)	2009-Jan-01	EPI_ISL_380254	C/Hong Kong/6483/2009	Government Virus Unit	Crick Worldwide Influenza Centre	
EPI1558363	NP	Hong Kong (SAR)	2009-Jan-01	EPI_ISL_380254	C/Hong Kong/6483/2009	Government Virus Unit	Crick Worldwide Influenza Centre	
EPI1607356	NS	Hong Kong (SAR)	2009-Jan-01	EPI_ISL_380254	C/Hong Kong/6483/2009	Government Virus Unit	Crick Worldwide Influenza Centre	
EPI1566808	P3	Hong Kong (SAR)	2009-Jan-01	EPI_ISL_380254	C/Hong Kong/6483/2009	Government Virus Unit	Crick Worldwide Influenza Centre	
EPI1567407	PB1	Hong Kong (SAR)	2009-Jan-01	EPI_ISL_380254	C/Hong Kong/6483/2009	Government Virus Unit	Crick Worldwide Influenza Centre	
EPI1590452	PB2	Hong Kong (SAR)	2009-Jan-01	EPI_ISL_380254	C/Hong Kong/6483/2009	Government Virus Unit	Crick Worldwide Influenza Centre	
EPI1564361	HE	Hong Kong (SAR)	2016-Jan-14	EPI_ISL_381032	C/Hong Kong/6557/2016	Government Virus Unit	Crick Worldwide Influenza Centre	
EPI1595526	MP	Hong Kong (SAR)	2016-Jan-14	EPI_ISL_381032	C/Hong Kong/6557/2016	Government Virus Unit	Crick Worldwide Influenza Centre	
EPI1607386	NS	Hong Kong (SAR)	2016-Jan-14	EPI_ISL_381032	C/Hong Kong/6557/2016	Government Virus Unit	Crick Worldwide Influenza Centre	
EPI1564362	HE	Hong Kong (SAR)	2016-Jan-14	EPI_ISL_381033	C/Hong Kong/6573/2016	Government Virus Unit	Crick Worldwide Influenza Centre	
EPI1595527	MP	Hong Kong (SAR)	2016-Jan-14	EPI_ISL_381033	C/Hong Kong/6573/2016	Government Virus Unit	Crick Worldwide Influenza Centre	
EPI1607387	NS	Hong Kong (SAR)	2016-Jan-14	EPI_ISL_381033	C/Hong Kong/6573/2016	Government Virus Unit	Crick Worldwide Influenza Centre	
EPI1607357	NS	Hong Kong (SAR)	2018-Jan-10	EPI_ISL_395062	C/Hong Kong/7663/2018	Government Virus Unit	Crick Worldwide Influenza Centre	
EPI1805316	MP	Hong Kong (SAR)	2020-Jan-09	EPI_ISL_574775	C/Hong Kong/7821/2020	Government Virus Unit	Crick Worldwide Influenza Centre	
EPI1564363	HE	Hong Kong (SAR)	2018-Jan-09	EPI_ISL_380223	C/Hong Kong/7920/2018	Government Virus Unit	Crick Worldwide Influenza Centre	
EPI1595528	MP	Hong Kong (SAR)	2018-Jan-09	EPI_ISL_380223	C/Hong Kong/7920/2018	Government Virus Unit	Crick Worldwide Influenza Centre	
EPI1558332	NP	Hong Kong (SAR)	2018-Jan-09	EPI_ISL_380223	C/Hong Kong/7920/2018	Government Virus Unit	Crick Worldwide Influenza Centre	
EPI1607358	NS	Hong Kong (SAR)	2018-Jan-09	EPI_ISL_380223	C/Hong Kong/7920/2018	Government Virus Unit	Crick Worldwide Influenza Centre	
EPI1566794	P3	Hong Kong (SAR)	2018-Jan-09	EPI_ISL_380223	C/Hong Kong/7920/2018	Government Virus Unit	Crick Worldwide Influenza Centre	
EPI1564364	HE	Hong Kong (SAR)	2018-Jan-10	EPI_ISL_380222	C/Hong Kong/7965/2018	Government Virus Unit	Crick Worldwide Influenza Centre	
EPI1595529	MP	Hong Kong (SAR)	2018-Jan-10	EPI_ISL_380222	C/Hong Kong/7965/2018	Government Virus Unit	Crick Worldwide Influenza Centre	
EPI1558331	NP	Hong Kong (SAR)	2018-Jan-10	EPI_ISL_380222	C/Hong Kong/7965/2018	Government Virus Unit	Crick Worldwide Influenza Centre	
EPI1607359	NS	Hong Kong (SAR)	2018-Jan-10	EPI_ISL_380222	C/Hong Kong/7965/2018	Government Virus Unit	Crick Worldwide Influenza Centre	
EPI1566793	P3	Hong Kong (SAR)	2018-Jan-10	EPI_ISL_380222	C/Hong Kong/7965/2018	Government Virus Unit	Crick Worldwide Influenza Centre	
EPI1567384	PB1	Hong Kong (SAR)	2018-Jan-10	EPI_ISL_380222	C/Hong Kong/7965/2018	Government Virus Unit	Crick Worldwide Influenza Centre	
EPI1590453	PB2	Hong Kong (SAR)	2018-Jan-10	EPI_ISL_380222	C/Hong Kong/7965/2018	Government Virus Unit	Crick Worldwide Influenza Centre	
EPI1564365	HE	Hong Kong (SAR)	2018-Jan-10	EPI_ISL_381034	C/Hong Kong/7985/2018	Government Virus Unit	Crick Worldwide Influenza Centre	
EPI1595530	MP	Hong Kong (SAR)	2018-Jan-10	EPI_ISL_381034	C/Hong Kong/7985/2018	Government Virus Unit	Crick Worldwide Influenza Centre	
EPI1607360	NS	Hong Kong (SAR)	2018-Jan-10	EPI_ISL_381034	C/Hong Kong/7985/2018	Government Virus Unit	Crick Worldwide Influenza Centre	
EPI1566792	P3	Hong Kong (SAR)	2018-Jan-10	EPI_ISL_381034	C/Hong Kong/7985/2018	Government Virus Unit	Crick Worldwide Influenza Centre	
EPI1590454	PB2	Hong Kong (SAR)	2018-Jan-10	EPI_ISL_381034	C/Hong Kong/7985/2018	Government Virus Unit	Crick Worldwide Influenza Centre	
EPI1805273	HE	Hong Kong (SAR)	2020-Jan-10	EPI_ISL_574768	C/Hong Kong/8873/2020	Government Virus Unit	Crick Worldwide Influenza Centre	
EPI1564366	HE	Hong Kong (SAR)	2018-Apr-06	EPI_ISL_380228	C/Hong Kong/91316/2018	Government Virus Unit	Crick Worldwide Influenza Centre	
EPI1595531	MP	Hong Kong (SAR)	2018-Apr-06	EPI_ISL_380228	C/Hong Kong/91316/2018	Government Virus Unit	Crick Worldwide Influenza Centre	
EPI1558337	NP	Hong Kong (SAR)	2018-Apr-06	EPI_ISL_380228	C/Hong Kong/91316/2018	Government Virus Unit	Crick Worldwide Influenza Centre	
EPI1607361	NS	Hong Kong (SAR)	2018-Apr-06	EPI_ISL_380228	C/Hong Kong/91316/2018	Government Virus Unit	Crick Worldwide Influenza Centre	
EPI1590455	PB2	Hong Kong (SAR)	2018-Apr-06	EPI_ISL_380228	C/Hong Kong/91316/2018	Government Virus Unit	Crick Worldwide Influenza Centre	
EPI1564376	HE	United States	2007-Jan-01	EPI_ISL_380249	C/Hong Kong/9172/2007	St. Jude Children's Research Hospital	Crick Worldwide Influenza Centre	
EPI1595532	MP	United States	2007-Jan-01	EPI_ISL_380249	C/Hong Kong/9172/2007	St. Jude Children's Research Hospital	Crick Worldwide Influenza Centre	
EPI1558358	NP	United States	2007-Jan-01	EPI_ISL_380249	C/Hong Kong/9172/2007	St. Jude Children's Research Hospital	Crick Worldwide Influenza Centre	
EPI1607362	NS	United States	2007-Jan-01	EPI_ISL_380249	C/Hong Kong/9172/2007	St. Jude Children's Research Hospital	Crick Worldwide Influenza Centre	
EPI1566780	P3	United States	2007-Jan-01	EPI_ISL_380249	C/Hong Kong/9172/2007	St. Jude Children's Research Hospital	Crick Worldwide Influenza Centre	
EPI1567403	PB1	United States	2007-Jan-01	EPI_ISL_380249	C/Hong Kong/9172/2007	St. Jude Children's Research Hospital	Crick Worldwide Influenza Centre	
EPI1590456	PB2	United States	2007-Jan-01	EPI_ISL_380249	C/Hong Kong/9172/2007	St. Jude Children's Research Hospital	Crick Worldwide Influenza Centre	
EPI1564377	HE	Hong Kong (SAR)	2011-Jan-01	EPI_ISL_380255	C/Hong Kong/9284/2011	Government Virus Unit	Crick Worldwide Influenza Centre	
EPI1595533	MP	Hong Kong (SAR)	2011-Jan-01	EPI_ISL_380255	C/Hong Kong/9284/2011	Government Virus Unit	Crick Worldwide Influenza Centre	
EPI1558364	NP	Hong Kong (SAR)	2011-Jan-01	EPI_ISL_380255	C/Hong Kong/9284/2011	Government Virus Unit	Crick Worldwide Influenza Centre	
EPI1607363	NS	Hong Kong (SAR)	2011-Jan-01	EPI_ISL_380255	C/Hong Kong/9284/2011	Government Virus Unit	Crick Worldwide Influenza Centre	
EPI1566810	P3	Hong Kong (SAR)	2011-Jan-01	EPI_ISL_380255	C/Hong Kong/9284/2011	Government Virus Unit	Crick Worldwide Influenza Centre	
EPI1590457	PB2	Hong Kong (SAR)	2011-Jan-01	EPI_ISL_380255	C/Hong Kong/9284/2011	Government Virus Unit	Crick Worldwide Influenza Centre	
EPI1564367	HE	Hong Kong (SAR)	2018-Apr-12	EPI_ISL_380229	C/Hong Kong/94533/2018	Government Virus Unit	Crick Worldwide Influenza Centre	
EPI1595534	MP	Hong Kong (SAR)	2018-Apr-12	EPI_ISL_380229	C/Hong Kong/94533/2018	Government Virus Unit	Crick Worldwide Influenza Centre	
EPI1558338	NP	Hong Kong (SAR)	2018-Apr-12	EPI_ISL_380229	C/Hong Kong/94533/2018	Government Virus Unit	Crick Worldwide Influenza Centre	
EPI1607364	NS	Hong Kong (SAR)	2018-Apr-12	EPI_ISL_380229	C/Hong Kong/94533/2018	Government Virus Unit	Crick Worldwide Influenza Centre	
EPI1566813	P3	Hong Kong (SAR)	2018-Apr-12	EPI_ISL_380229	C/Hong Kong/94533/2018	Government Virus Unit	Crick Worldwide Influenza Centre	
EPI1567389	PB1	Hong Kong (SAR)	2018-Apr-12	EPI_ISL_380229	C/Hong Kong/94533/2018	Government Virus Unit	Crick Worldwide Influenza Centre	
EPI1590458	PB2	Hong Kong (SAR)	2018-Apr-12	EPI_ISL_380229	C/Hong Kong/94533/2018	Government Virus Unit	Crick Worldwide Influenza Centre	
EPI1564368	HE	Hong Kong (SAR)	2018-Apr-14	EPI_ISL_380230	C/Hong Kong/95361/2018	Government Virus Unit	Crick Worldwide Influenza Centre	
EPI1595535	MP	Hong Kong (SAR)	2018-Apr-14	EPI_ISL_380230	C/Hong Kong/95361/2018	Government Virus Unit	Crick Worldwide Influenza Centre	
EPI1558339	NP	Hong Kong (SAR)	2018-Apr-14	EPI_ISL_380230	C/Hong Kong/95361/2018	Government Virus Unit	Crick Worldwide Influenza Centre	
EPI1607366	NS	Hong Kong (SAR)	2018-Apr-14	EPI_ISL_380230	C/Hong Kong/95361/2018	Government Virus Unit	Crick Worldwide Influenza Centre	
EPI1566814	P3	Hong Kong (SAR)	2018-Apr-14	EPI_ISL_380230	C/Hong Kong/95361/2018	Government Virus Unit	Crick Worldwide Influenza Centre	
EPI1590460	PB2	Hong Kong (SAR)	2018-Apr-14	EPI_ISL_380230	C/Hong Kong/95361/2018	Government Virus Unit	Crick Worldwide Influenza Centre	
EPI1564370	HE	Hong Kong (SAR)	2018-Apr-15	EPI_ISL_380231	C/Hong Kong/96123/2018	Government Virus Unit	Crick Worldwide Influenza Centre	
EPI1595537	MP	Hong Kong (SAR)	2018-Apr-15	EPI_ISL_380231	C/Hong Kong/96123/2018	Government Virus Unit	Crick Worldwide Influenza Centre	
EPI1558340	NP	Hong Kong (SAR)	2018-Apr-15	EPI_ISL_380231	C/Hong Kong/96123/2018	Government Virus Unit	Crick Worldwide Influenza Centre	
EPI1607367	NS	Hong Kong (SAR)	2018-Apr-15	EPI_ISL_380231	C/Hong Kong/96123/2018	Government Virus Unit	Crick Worldwide Influenza Centre	
EPI1566815	P3	Hong Kong (SAR)	2018-Apr-15	EPI_ISL_380231	C/Hong Kong/96123/2018	Government Virus Unit	Crick Worldwide Influenza Centre	
EPI1567390	PB1	Hong Kong (SAR)	2018-Apr-15	EPI_ISL_380231	C/Hong Kong/96123/2018	Government Virus Unit	Crick Worldwide Influenza Centre	
EPI1590461	PB2	Hong Kong (SAR)	2018-Apr-15	EPI_ISL_380231	C/Hong Kong/96123/2018	Government Virus Unit	Crick Worldwide Influenza Centre	
EPI1564372	HE	Hong Kong (SAR)	2018-Apr-14	EPI_ISL_380233	C/Hong Kong/96417/2018	Government Virus Unit	Crick Worldwide Influenza Centre	
EPI1595539	MP	Hong Kong (SAR)	2018-Apr-14	EPI_ISL_380233	C/Hong Kong/96417/2018	Government Virus Unit	Crick Worldwide Influenza Centre	
EPI1558342	NP	Hong Kong (SAR)	2018-Apr-14	EPI_ISL_380233	C/Hong Kong/96417/2018	Government Virus Unit	Crick Worldwide Influenza Centre	
EPI1607368	NS	Hong Kong (SAR)	2018-Apr-14	EPI_ISL_380233	C/Hong Kong/96417/2018	Government Virus Unit	Crick Worldwide Influenza Centre	
EPI1566816	P3	Hong Kong (SAR)	2018-Apr-14	EPI_ISL_380233	C/Hong Kong/96417/2018	Government Virus Unit	Crick Worldwide Influenza Centre	
EPI1567392	PB1	Hong Kong (SAR)	2018-Apr-14	EPI_ISL_380233	C/Hong Kong/96417/2018	Government Virus Unit	Crick Worldwide Influenza Centre	
EPI1590463	PB2	Hong Kong (SAR)	2018-Apr-14	EPI_ISL_380233	C/Hong Kong/96417/2018	Government Virus Unit	Crick Worldwide Influenza Centre	
EPI1564373	HE	Hong Kong (SAR)	2018-Apr-17	EPI_ISL_381035	C/Hong Kong/97303/2018	Government Virus Unit	Crick Worldwide Influenza Centre	
EPI1595540	MP	Hong Kong (SAR)	2018-Apr-17	EPI_ISL_381035	C/Hong Kong/97303/2018	Government Virus Unit	Crick Worldwide Influenza Centre	
EPI231555	HE	Japan	1983-Jan-01	EPI_ISL_66346	C/Hyogo/1/83		Import from public-domain	Adachi,K.; Kitame,F.; Sugawara,K.; Nishimura,H.; Nakamura,K.
EPI232077	MP	Japan	1983-Jan-01	EPI_ISL_66436	C/Hyogo/1/83		Import from public-domain	Matsuzaki,Y.
EPI232026	NS	Japan	1983-Jan-01	EPI_ISL_66436	C/Hyogo/1/83		Import from public-domain	Matsuzaki,Y.
EPI743436	HE	India	2012-Feb-24	EPI_ISL_218313	C/India/P121719/2012		Import from public-domain	Potdar,V.A.; Dakhave,M.R.; Manchanda,A.
EPI743438	MP	India	2012-Feb-24	EPI_ISL_218313	C/India/P121719/2012		Import from public-domain	Potdar,V.A.; Dakhave,M.R.; Manchanda,A.
EPI743437	NP	India	2012-Feb-24	EPI_ISL_218313	C/India/P121719/2012		Import from public-domain	Potdar,V.A.; Dakhave,M.R.; Manchanda,A.
EPI743538	NS	India	2012-Feb-24	EPI_ISL_218313	C/India/P121719/2012		Import from public-domain	Potdar,V.A.; Dakhave,M.R.; Manchanda,A.
EPI743435	P3	India	2012-Feb-24	EPI_ISL_218313	C/India/P121719/2012		Import from public-domain	Potdar,V.A.; Dakhave,M.R.; Manchanda,A.
EPI743434	PB1	India	2012-Feb-24	EPI_ISL_218313	C/India/P121719/2012		Import from public-domain	Potdar,V.A.; Dakhave,M.R.; Manchanda,A.
EPI743433	PB2	India	2012-Feb-24	EPI_ISL_218313	C/India/P121719/2012		Import from public-domain	Potdar,V.A.; Dakhave,M.R.; Manchanda,A.
EPI743443	HE	India	2013-Apr-25	EPI_ISL_218314	C/India/P135047/2013		Import from public-domain	Potdar,V.A.; Dakhave,M.R.; Manchanda,A.
EPI743445	MP	India	2013-Apr-25	EPI_ISL_218314	C/India/P135047/2013		Import from public-domain	Potdar,V.A.; Dakhave,M.R.; Manchanda,A.
EPI743444	NP	India	2013-Apr-25	EPI_ISL_218314	C/India/P135047/2013		Import from public-domain	Potdar,V.A.; Dakhave,M.R.; Manchanda,A.
EPI743539	NS	India	2013-Apr-25	EPI_ISL_218314	C/India/P135047/2013		Import from public-domain	Potdar,V.A.; Dakhave,M.R.; Manchanda,A.
EPI743442	P3	India	2013-Apr-25	EPI_ISL_218314	C/India/P135047/2013		Import from public-domain	Potdar,V.A.; Dakhave,M.R.; Manchanda,A.
EPI743441	PB1	India	2013-Apr-25	EPI_ISL_218314	C/India/P135047/2013		Import from public-domain	Potdar,V.A.; Dakhave,M.R.; Manchanda,A.
EPI743440	PB2	India	2013-Apr-25	EPI_ISL_218314	C/India/P135047/2013		Import from public-domain	Potdar,V.A.; Dakhave,M.R.; Manchanda,A.
EPI283749	HE		1950-Jan-01	EPI_ISL_80396	C/JJ/1950		Import from public-domain	Pachler,K.
EPI283750	NP		1950-Jan-01	EPI_ISL_80396	C/JJ/1950		Import from public-domain	Pachler,K.
EPI283752	NS		1950-Jan-01	EPI_ISL_80396	C/JJ/1950		Import from public-domain	Pachler,K.
EPI231515	MP		1950-Jan-01	EPI_ISL_66314	C/JJ/50		Import from public-domain	Yamashita,M.; Krystal,M.; Palese,P.
EPI231509	P3		1950-Jan-01	EPI_ISL_66314	C/JJ/50		Import from public-domain	Yamashita,M.; Krystal,M.; Palese,P.
EPI231510	PB1		1950-Jan-01	EPI_ISL_66314	C/JJ/50		Import from public-domain	Yamashita,M.; Krystal,M.; Palese,P.
EPI231511	PB2		1950-Jan-01	EPI_ISL_66314	C/JJ/50		Import from public-domain	Yamashita,M.; Krystal,M.; Palese,P.
EPI232083	HE	South Africa	1966-Jan-01	EPI_ISL_66432	C/Johannesburg/1/66		Import from public-domain	Crescenzo-Chaigne,B.
EPI232017	MP	South Africa	1966-Jan-01	EPI_ISL_66432	C/Johannesburg/1/66		Import from public-domain	Crescenzo-Chaigne,B.
EPI232078	NP	South Africa	1966-Jan-01	EPI_ISL_66432	C/Johannesburg/1/66		Import from public-domain	Crescenzo-Chaigne,B.
EPI232018	NS	South Africa	1966-Jan-01	EPI_ISL_66432	C/Johannesburg/1/66		Import from public-domain	Crescenzo-Chaigne,B.
EPI232079	P3	South Africa	1966-Jan-01	EPI_ISL_66432	C/Johannesburg/1/66		Import from public-domain	Crescenzo-Chaigne,B.
EPI232080	PB1	South Africa	1966-Jan-01	EPI_ISL_66432	C/Johannesburg/1/66		Import from public-domain	Crescenzo-Chaigne,B.
EPI232081	PB2	South Africa	1966-Jan-01	EPI_ISL_66432	C/Johannesburg/1/66		Import from public-domain	Crescenzo-Chaigne,B.
EPI231523	HE	South Africa	1967-Jan-01	EPI_ISL_66323	C/Johannesburg/4/67		Import from public-domain	Matsuzaki,Y.; Ikeda,T.; Abiko,C.; Aoki,Y.; Mizuta,K.; Shimotai,Y.; Sugawara,K.; Hongo,S.; Sugawara,K.; Furuse,Y.; Shimotai,Y.; Hongo,S.; Oshitani,H.; Mizuta,K.; Nishimura,H.
EPI816669	MP	South Africa	1967-Jan-01	EPI_ISL_66323	C/Johannesburg/4/67		Import from public-domain	Matsuzaki,Y.; Ikeda,T.; Abiko,C.; Aoki,Y.; Mizuta,K.; Shimotai,Y.; Sugawara,K.; Hongo,S.; Sugawara,K.; Furuse,Y.; Shimotai,Y.; Hongo,S.; Oshitani,H.; Mizuta,K.; Nishimura,H.
EPI369793	NP	South Africa	1967-Jan-01	EPI_ISL_118428	C/Johannesburg/4/67		Import from public-domain	Matsuzaki,Y.; Ikeda,T.; Abiko,C.; Aoki,Y.; Mizuta,K.; Shimotai,Y.; Sugawara,K.; Hongo,S.
EPI231524	NS	South Africa	1967-Jan-01	EPI_ISL_66323	C/Johannesburg/4/67		Import from public-domain	Matsuzaki,Y.; Ikeda,T.; Abiko,C.; Aoki,Y.; Mizuta,K.; Shimotai,Y.; Sugawara,K.; Hongo,S.; Sugawara,K.; Furuse,Y.; Shimotai,Y.; Hongo,S.; Oshitani,H.; Mizuta,K.; Nishimura,H.
EPI816461	P3	South Africa	1967-Jan-01	EPI_ISL_66323	C/Johannesburg/4/67		Import from public-domain	Matsuzaki,Y.; Ikeda,T.; Abiko,C.; Aoki,Y.; Mizuta,K.; Shimotai,Y.; Sugawara,K.; Hongo,S.; Sugawara,K.; Furuse,Y.; Shimotai,Y.; Hongo,S.; Oshitani,H.; Mizuta,K.; Nishimura,H.
EPI813784	PB1	South Africa	1967-Jan-01	EPI_ISL_66323	C/Johannesburg/4/67		Import from public-domain	Matsuzaki,Y.; Ikeda,T.; Abiko,C.; Aoki,Y.; Mizuta,K.; Shimotai,Y.; Sugawara,K.; Hongo,S.; Sugawara,K.; Furuse,Y.; Shimotai,Y.; Hongo,S.; Oshitani,H.; Mizuta,K.; Nishimura,H.
EPI814734	PB2	South Africa	1967-Jan-01	EPI_ISL_66323	C/Johannesburg/4/67		Import from public-domain	Matsuzaki,Y.; Ikeda,T.; Abiko,C.; Aoki,Y.; Mizuta,K.; Shimotai,Y.; Sugawara,K.; Hongo,S.; Sugawara,K.; Furuse,Y.; Shimotai,Y.; Hongo,S.; Oshitani,H.; Mizuta,K.; Nishimura,H.
EPI232085	HE	Japan	1976-Jan-01	EPI_ISL_66328	C/Kanagawa/1/76		Import from public-domain	Matsuzaki,Y.; Sugawara,K.; Furuse,Y.; Shimotai,Y.; Hongo,S.; Oshitani,H.; Mizuta,K.; Nishimura,H.
EPI232086	MP	Japan	1976-Jan-01	EPI_ISL_66328	C/Kanagawa/1/76		Import from public-domain	Matsuzaki,Y.; Sugawara,K.; Furuse,Y.; Shimotai,Y.; Hongo,S.; Oshitani,H.; Mizuta,K.; Nishimura,H.
EPI816576	NP	Japan	1976-Jan-01	EPI_ISL_66328	C/Kanagawa/1/76		Import from public-domain	Matsuzaki,Y.; Sugawara,K.; Furuse,Y.; Shimotai,Y.; Hongo,S.; Oshitani,H.; Mizuta,K.; Nishimura,H.
EPI231529	NS	Japan	1976-Jan-01	EPI_ISL_66328	C/Kanagawa/1/76		Import from public-domain	Matsuzaki,Y.; Sugawara,K.; Furuse,Y.; Shimotai,Y.; Hongo,S.; Oshitani,H.; Mizuta,K.; Nishimura,H.
EPI816467	P3	Japan	1976-Jan-01	EPI_ISL_66328	C/Kanagawa/1/76		Import from public-domain	Matsuzaki,Y.; Sugawara,K.; Furuse,Y.; Shimotai,Y.; Hongo,S.; Oshitani,H.; Mizuta,K.; Nishimura,H.
EPI813787	PB1	Japan	1976-Jan-01	EPI_ISL_66328	C/Kanagawa/1/76		Import from public-domain	Matsuzaki,Y.; Sugawara,K.; Furuse,Y.; Shimotai,Y.; Hongo,S.; Oshitani,H.; Mizuta,K.; Nishimura,H.
EPI814540	PB2	Japan	1976-Jan-01	EPI_ISL_66328	C/Kanagawa/1/76		Import from public-domain	Matsuzaki,Y.; Sugawara,K.; Furuse,Y.; Shimotai,Y.; Hongo,S.; Oshitani,H.; Mizuta,K.; Nishimura,H.
EPI228327	HE	Japan	2004-Jan-01	EPI_ISL_65163	C/Kanagawa/2/2004		Import from public-domain	Matsuzaki,Y.
EPI231532	HE	United States	1979-Jan-01	EPI_ISL_66331	C/Kansas/1/79		Import from public-domain	Matsuzaki,Y.; Sugawara,K.; Furuse,Y.; Shimotai,Y.; Hongo,S.; Oshitani,H.; Mizuta,K.; Nishimura,H.
EPI231649	MP	United States	1979-Jan-01	EPI_ISL_66331	C/Kansas/1/79		Import from public-domain	Matsuzaki,Y.; Sugawara,K.; Furuse,Y.; Shimotai,Y.; Hongo,S.; Oshitani,H.; Mizuta,K.; Nishimura,H.
EPI816579	NP	United States	1979-Jan-01	EPI_ISL_66331	C/Kansas/1/79		Import from public-domain	Matsuzaki,Y.; Sugawara,K.; Furuse,Y.; Shimotai,Y.; Hongo,S.; Oshitani,H.; Mizuta,K.; Nishimura,H.
EPI231648	NS	United States	1979-Jan-01	EPI_ISL_66331	C/Kansas/1/79		Import from public-domain	Matsuzaki,Y.; Sugawara,K.; Furuse,Y.; Shimotai,Y.; Hongo,S.; Oshitani,H.; Mizuta,K.; Nishimura,H.

Segment ID	Segment	Country	Collection date	Isolate-ID	Isolate name	Originating Lab	Submitting Lab	Authors
EPI816470	P3	United States	1979-Jan-01	EPI_ISL_66331	C/Kansas/1/79		Import from public-domain	Matsuzaki,Y.; Sugawara,K.; Furuse,Y.; Shimotai,Y.; Hongo,S.; Oshitani,H.; Mizuta,K.; Nishimura,H.
EPI813788	PB1	United States	1979-Jan-01	EPI_ISL_66331	C/Kansas/1/79		Import from public-domain	Matsuzaki,Y.; Sugawara,K.; Furuse,Y.; Shimotai,Y.; Hongo,S.; Oshitani,H.; Mizuta,K.; Nishimura,H.
EPI814533	PB2	United States	1979-Jan-01	EPI_ISL_66331	C/Kansas/1/79		Import from public-domain	Matsuzaki,Y.; Sugawara,K.; Furuse,Y.; Shimotai,Y.; Hongo,S.; Oshitani,H.; Mizuta,K.; Nishimura,H.
EPI231533	HE	United States	1979-Jan-01	EPI_ISL_66332	C/Kansas/2/79		Import from public-domain	Matsuzaki,Y.
EPI1564378	HE	United States	2009-Jan-01	EPI_ISL_380250	C/Kowloon/4956/2009	St. Jude Children's Research Hospital	Crick Worldwide Influenza Centre	
EPI1595541	MP	United States	2009-Jan-01	EPI_ISL_380250	C/Kowloon/4956/2009	St. Jude Children's Research Hospital	Crick Worldwide Influenza Centre	
EPI1558359	NP	United States	2009-Jan-01	EPI_ISL_380250	C/Kowloon/4956/2009	St. Jude Children's Research Hospital	Crick Worldwide Influenza Centre	
EPI1607369	NS	United States	2009-Jan-01	EPI_ISL_380250	C/Kowloon/4956/2009	St. Jude Children's Research Hospital	Crick Worldwide Influenza Centre	
EPI1566779	P3	United States	2009-Jan-01	EPI_ISL_380250	C/Kowloon/4956/2009	St. Jude Children's Research Hospital	Crick Worldwide Influenza Centre	
EPI1567404	PB1	United States	2009-Jan-01	EPI_ISL_380250	C/Kowloon/4956/2009	St. Jude Children's Research Hospital	Crick Worldwide Influenza Centre	
EPI1590464	PB2	United States	2009-Jan-01	EPI_ISL_380250	C/Kowloon/4956/2009	St. Jude Children's Research Hospital	Crick Worldwide Influenza Centre	
EPI232087	HE	Japan	1979-Jan-01	EPI_ISL_66334	C/Kyoto/1/79		Import from public-domain	Matsuzaki,Y.; Sugawara,K.; Furuse,Y.; Shimotai,Y.; Hongo,S.; Oshitani,H.; Mizuta,K.; Nishimura,H.
EPI232088	MP	Japan	1979-Jan-01	EPI_ISL_66334	C/Kyoto/1/79		Import from public-domain	Matsuzaki,Y.; Sugawara,K.; Furuse,Y.; Shimotai,Y.; Hongo,S.; Oshitani,H.; Mizuta,K.; Nishimura,H.
EPI816580	NP	Japan	1979-Jan-01	EPI_ISL_66334	C/Kyoto/1/79		Import from public-domain	Matsuzaki,Y.; Sugawara,K.; Furuse,Y.; Shimotai,Y.; Hongo,S.; Oshitani,H.; Mizuta,K.; Nishimura,H.
EPI231535	NS	Japan	1979-Jan-01	EPI_ISL_66334	C/Kyoto/1/79		Import from public-domain	Matsuzaki,Y.; Sugawara,K.; Furuse,Y.; Shimotai,Y.; Hongo,S.; Oshitani,H.; Mizuta,K.; Nishimura,H.
EPI816481	P3	Japan	1979-Jan-01	EPI_ISL_66334	C/Kyoto/1/79		Import from public-domain	Matsuzaki,Y.; Sugawara,K.; Furuse,Y.; Shimotai,Y.; Hongo,S.; Oshitani,H.; Mizuta,K.; Nishimura,H.
EPI813791	PB1	Japan	1979-Jan-01	EPI_ISL_66334	C/Kyoto/1/79		Import from public-domain	Matsuzaki,Y.; Sugawara,K.; Furuse,Y.; Shimotai,Y.; Hongo,S.; Oshitani,H.; Mizuta,K.; Nishimura,H.
EPI814535	PB2	Japan	1979-Jan-01	EPI_ISL_66334	C/Kyoto/1/79		Import from public-domain	Matsuzaki,Y.; Sugawara,K.; Furuse,Y.; Shimotai,Y.; Hongo,S.; Oshitani,H.; Mizuta,K.; Nishimura,H.
EPI231544	HE	Japan	1982-Jan-01	EPI_ISL_66341	C/Kyoto/41/82		Import from public-domain	Adachi,K.; Kitame,F.; Sugawara,K.; Nishimura,H.; Nakamura,K.
EPI232089	MP	Japan	1982-Jan-01	EPI_ISL_66435	C/Kyoto/41/82		Import from public-domain	Matsuzaki,Y.
EPI232090	MP	Japan	1982-Jan-01	EPI_ISL_66435	C/Kyoto/41/82		Import from public-domain	Matsuzaki,Y.
EPI232024	NS	Japan	1982-Jan-01	EPI_ISL_66435	C/Kyoto/41/82		Import from public-domain	Matsuzaki,Y.
EPI581537	HE	Philippines	2011-Jan-01	EPI_ISL_176772	C/Leyte/1/2011		Import from public-domain	Odagiri,T.; Matsuzaki,Y.; Okamoto,M.; Hongo,S.; Oshitani,H.
EPI621701	MP	Philippines	2011-Jan-01	EPI_ISL_176772	C/Leyte/1/2011		Import from public-domain	Odagiri,T.; Matsuzaki,Y.; Okamoto,M.; Hongo,S.; Oshitani,H.
EPI621685	NS	Philippines	2011-Jan-01	EPI_ISL_176772	C/Leyte/1/2011		Import from public-domain	Odagiri,T.; Matsuzaki,Y.; Okamoto,M.; Hongo,S.; Oshitani,H.
EPI581549	HE	Philippines	2013-Jan-01	EPI_ISL_176784	C/Leyte/1/2013		Import from public-domain	Odagiri,T.; Matsuzaki,Y.; Okamoto,M.; Hongo,S.; Oshitani,H.
EPI621704	MP	Philippines	2013-Jan-01	EPI_ISL_176784	C/Leyte/1/2013		Import from public-domain	Odagiri,T.; Matsuzaki,Y.; Okamoto,M.; Hongo,S.; Oshitani,H.
EPI621688	NS	Philippines	2013-Jan-01	EPI_ISL_176784	C/Leyte/1/2013		Import from public-domain	Odagiri,T.; Matsuzaki,Y.; Okamoto,M.; Hongo,S.; Oshitani,H.
EPI581538	HE	Philippines	2011-Jan-01	EPI_ISL_176773	C/Leyte/2/2011		Import from public-domain	Odagiri,T.; Matsuzaki,Y.; Okamoto,M.; Hongo,S.; Oshitani,H.
EPI621702	MP	Philippines	2011-Jan-01	EPI_ISL_176773	C/Leyte/2/2011		Import from public-domain	Odagiri,T.; Matsuzaki,Y.; Okamoto,M.; Hongo,S.; Oshitani,H.
EPI621686	NS	Philippines	2011-Jan-01	EPI_ISL_176773	C/Leyte/2/2011		Import from public-domain	Odagiri,T.; Matsuzaki,Y.; Okamoto,M.; Hongo,S.; Oshitani,H.
EPI581550	HE	Philippines	2013-Jan-01	EPI_ISL_176785	C/Leyte/2/2013		Import from public-domain	Odagiri,T.; Matsuzaki,Y.; Okamoto,M.; Hongo,S.; Oshitani,H.
EPI621705	MP	Philippines	2013-Jan-01	EPI_ISL_176785	C/Leyte/2/2013		Import from public-domain	Odagiri,T.; Matsuzaki,Y.; Okamoto,M.; Hongo,S.; Oshitani,H.
EPI621689	NS	Philippines	2013-Jan-01	EPI_ISL_176785	C/Leyte/2/2013		Import from public-domain	Odagiri,T.; Matsuzaki,Y.; Okamoto,M.; Hongo,S.; Oshitani,H.
EPI581539	HE	Philippines	2011-Jan-01	EPI_ISL_176774	C/Leyte/3/2011		Import from public-domain	Odagiri,T.; Matsuzaki,Y.; Okamoto,M.; Hongo,S.; Oshitani,H.
EPI621703	MP	Philippines	2011-Jan-01	EPI_ISL_176774	C/Leyte/3/2011		Import from public-domain	Odagiri,T.; Matsuzaki,Y.; Okamoto,M.; Hongo,S.; Oshitani,H.
EPI621687	NS	Philippines	2011-Jan-01	EPI_ISL_176774	C/Leyte/3/2011		Import from public-domain	Odagiri,T.; Matsuzaki,Y.; Okamoto,M.; Hongo,S.; Oshitani,H.
EPI581551	HE	Philippines	2013-Jan-01	EPI_ISL_176787	C/Leyte/3/2013		Import from public-domain	Odagiri,T.; Matsuzaki,Y.; Okamoto,M.; Hongo,S.; Oshitani,H.
EPI621706	MP	Philippines	2013-Jan-01	EPI_ISL_176787	C/Leyte/3/2013		Import from public-domain	Odagiri,T.; Matsuzaki,Y.; Okamoto,M.; Hongo,S.; Oshitani,H.
EPI621690	NS	Philippines	2013-Jan-01	EPI_ISL_176787	C/Leyte/3/2013		Import from public-domain	Odagiri,T.; Matsuzaki,Y.; Okamoto,M.; Hongo,S.; Oshitani,H.
EPI581553	HE	Philippines	2011-Jan-01	EPI_ISL_176994	C/Leyte/4/2011		Import from public-domain	Odagiri,T.; Matsuzaki,Y.; Okamoto,M.; Suzuki,A.; Saito,M.; Tamaki,R.; Lupisan,S.P.; Sombrero,L.T.; Hongo,S.; Oshitani,H.
EPI581554	HE	Philippines	2011-Jan-01	EPI_ISL_176995	C/Leyte/5/2011		Import from public-domain	Odagiri,T.; Matsuzaki,Y.; Okamoto,M.; Suzuki,A.; Saito,M.; Tamaki,R.; Lupisan,S.P.; Sombrero,L.T.; Hongo,S.; Oshitani,H.
EPI1507012	HE	Mauritania	2018	EPI_ISL_368021	C/Mauritania/252-2018/2018	INRSP-Institut National de Recherches en Sante Publique	Crick Worldwide Influenza Centre	
EPI475501	HE	Japan	2012-Jan-01	EPI_ISL_148120	C/Mie/180/2012cs		Import from public-domain	Yano,T.
EPI475497	HE	Japan	2012-Jan-01	EPI_ISL_148116	C/Mie/204/2012cs		Import from public-domain	Yano,T.
EPI1033772	HE	United States	2014-Dec-27	EPI_ISL_272577	C/Minnesota/01/2014	Minnesota Department of Health	Centers for Disease Control and Prevention	
EPI1033773	MP	United States	2014-Dec-27	EPI_ISL_272577	C/Minnesota/01/2014	Minnesota Department of Health	Centers for Disease Control and Prevention	
EPI1033774	HE	United States	2015-Jan-01	EPI_ISL_272578	C/Minnesota/01/2015	Minnesota Department of Health	Centers for Disease Control and Prevention	
EPI1033775	MP	United States	2015-Jan-01	EPI_ISL_272578	C/Minnesota/01/2015	Minnesota Department of Health	Centers for Disease Control and Prevention	
EPI1033840	HE	United States	2016-Apr-21	EPI_ISL_272613	C/Minnesota/01/2016	Minnesota Department of Health	Centers for Disease Control and Prevention	
EPI1033841	MP	United States	2016-Apr-21	EPI_ISL_272613	C/Minnesota/01/2016	Minnesota Department of Health	Centers for Disease Control and Prevention	
EPI1033770	HE	United States	2014-Dec-26	EPI_ISL_272576	C/Minnesota/02/2014	Minnesota Department of Health	Centers for Disease Control and Prevention	
EPI1033771	MP	United States	2014-Dec-26	EPI_ISL_272576	C/Minnesota/02/2014	Minnesota Department of Health	Centers for Disease Control and Prevention	
EPI1033776	HE	United States	2015-Jan-09	EPI_ISL_272579	C/Minnesota/02/2015	Minnesota Department of Health	Centers for Disease Control and Prevention	
EPI1033777	MP	United States	2015-Jan-09	EPI_ISL_272579	C/Minnesota/02/2015	Minnesota Department of Health	Centers for Disease Control and Prevention	
EPI1033821	HE	United States	2014-Dec-15	EPI_ISL_272603	C/Minnesota/03/2014	Minnesota Department of Health	Centers for Disease Control and Prevention	
EPI1033822	MP	United States	2014-Dec-15	EPI_ISL_272603	C/Minnesota/03/2014	Minnesota Department of Health	Centers for Disease Control and Prevention	
EPI1033778	HE	United States	2015-Jan-14	EPI_ISL_272580	C/Minnesota/03/2015	Minnesota Department of Health	Centers for Disease Control and Prevention	
EPI1033779	MP	United States	2015-Jan-14	EPI_ISL_272580	C/Minnesota/03/2015	Minnesota Department of Health	Centers for Disease Control and Prevention	
EPI1033823	HE	United States	2014-Dec-25	EPI_ISL_272604	C/Minnesota/04/2014	Minnesota Department of Health	Centers for Disease Control and Prevention	
EPI1033824	MP	United States	2014-Dec-25	EPI_ISL_272604	C/Minnesota/04/2014	Minnesota Department of Health	Centers for Disease Control and Prevention	
EPI1033780	HE	United States	2015-Feb-06	EPI_ISL_272581	C/Minnesota/04/2015	Minnesota Department of Health	Centers for Disease Control and Prevention	
EPI1033781	MP	United States	2015-Feb-06	EPI_ISL_272581	C/Minnesota/04/2015	Minnesota Department of Health	Centers for Disease Control and Prevention	
EPI1033782	HE	United States	2015-Feb-05	EPI_ISL_272582	C/Minnesota/05/2015	Minnesota Department of Health	Centers for Disease Control and Prevention	
EPI1033783	MP	United States	2015-Feb-05	EPI_ISL_272582	C/Minnesota/05/2015	Minnesota Department of Health	Centers for Disease Control and Prevention	
EPI1033784	HE	United States	2015-Feb-15	EPI_ISL_272583	C/Minnesota/06/2015	Minnesota Department of Health	Centers for Disease Control and Prevention	
EPI1033785	MP	United States	2015-Feb-15	EPI_ISL_272583	C/Minnesota/06/2015	Minnesota Department of Health	Centers for Disease Control and Prevention	
EPI1033786	HE	United States	2015-Feb-18	EPI_ISL_272584	C/Minnesota/07/2015	Minnesota Department of Health	Centers for Disease Control and Prevention	
EPI1033787	MP	United States	2015-Feb-18	EPI_ISL_272584	C/Minnesota/07/2015	Minnesota Department of Health	Centers for Disease Control and Prevention	
EPI1033788	HE	United States	2015-Mar-04	EPI_ISL_272585	C/Minnesota/08/2015	Minnesota Department of Health	Centers for Disease Control and Prevention	
EPI1033789	MP	United States	2015-Mar-04	EPI_ISL_272585	C/Minnesota/08/2015	Minnesota Department of Health	Centers for Disease Control and Prevention	
EPI1033790	HE	United States	2015-Mar-10	EPI_ISL_272586	C/Minnesota/09/2015	Minnesota Department of Health	Centers for Disease Control and Prevention	
EPI1033792	HE	United States	2015-Mar-09	EPI_ISL_272587	C/Minnesota/10/2015	Minnesota Department of Health	Centers for Disease Control and Prevention	
EPI1033793	MP	United States	2015-Mar-09	EPI_ISL_272587	C/Minnesota/10/2015	Minnesota Department of Health	Centers for Disease Control and Prevention	
EPI1033794	HE	United States	2015-Mar-12	EPI_ISL_272588	C/Minnesota/12/2015	Minnesota Department of Health	Centers for Disease Control and Prevention	
EPI1033795	MP	United States	2015-Mar-12	EPI_ISL_272588	C/Minnesota/12/2015	Minnesota Department of Health	Centers for Disease Control and Prevention	
EPI1033796	HE	United States	2015-Mar-21	EPI_ISL_272589	C/Minnesota/13/2015	Minnesota Department of Health	Centers for Disease Control and Prevention	
EPI1033797	MP	United States	2015-Mar-21	EPI_ISL_272589	C/Minnesota/13/2015	Minnesota Department of Health	Centers for Disease Control and Prevention	
EPI1033798	HE	United States	2015-Mar-23	EPI_ISL_272590	C/Minnesota/14/2015	Minnesota Department of Health	Centers for Disease Control and Prevention	
EPI1033799	MP	United States	2015-Mar-23	EPI_ISL_272590	C/Minnesota/14/2015	Minnesota Department of Health	Centers for Disease Control and Prevention	
EPI1033800	HE	United States	2015-Mar-20	EPI_ISL_272591	C/Minnesota/15/2015	Minnesota Department of Health	Centers for Disease Control and Prevention	
EPI1033801	MP	United States	2015-Mar-20	EPI_ISL_272591	C/Minnesota/15/2015	Minnesota Department of Health	Centers for Disease Control and Prevention	
EPI1033802	HE	United States	2015-Mar-19	EPI_ISL_272592	C/Minnesota/16/2015	Minnesota Department of Health	Centers for Disease Control and Prevention	
EPI1033803	HE	United States	2015-Mar-27	EPI_ISL_272593	C/Minnesota/17/2015	Minnesota Department of Health	Centers for Disease Control and Prevention	
EPI1033804	HE	United States	2015-Mar-27	EPI_ISL_272594	C/Minnesota/18/2015	Minnesota Department of Health	Centers for Disease Control and Prevention	
EPI1033805	MP	United States	2015-Mar-27	EPI_ISL_272594	C/Minnesota/18/2015	Minnesota Department of Health	Centers for Disease Control and Prevention	
EPI1033806	HE	United States	2015-Mar-30	EPI_ISL_272595	C/Minnesota/19/2015	Minnesota Department of Health	Centers for Disease Control and Prevention	
EPI1033807	MP	United States	2015-Mar-30	EPI_ISL_272595	C/Minnesota/19/2015	Minnesota Department of Health	Centers for Disease Control and Prevention	
EPI1033808	HE	United States	2015-Mar-29	EPI_ISL_272596	C/Minnesota/20/2015	Minnesota Department of Health	Centers for Disease Control and Prevention	
EPI1033809	MP	United States	2015-Mar-29	EPI_ISL_272596	C/Minnesota/20/2015	Minnesota Department of Health	Centers for Disease Control and Prevention	
EPI1033810	HE	United States	2015-Mar-29	EPI_ISL_272597	C/Minnesota/21/2015	Minnesota Department of Health	Centers for Disease Control and Prevention	
EPI1033811	HE	United States	2015-Mar-31	EPI_ISL_272598	C/Minnesota/22/2015	Minnesota Department of Health	Centers for Disease Control and Prevention	
EPI1033812	MP	United States	2015-Mar-31	EPI_ISL_272598	C/Minnesota/22/2015	Minnesota Department of Health	Centers for Disease Control and Prevention	
EPI1033813	HE	United States	2015-Apr-15	EPI_ISL_272599	C/Minnesota/23/2015	Minnesota Department of Health	Centers for Disease Control and Prevention	
EPI1033814	MP	United States	2015-Apr-15	EPI_ISL_272599	C/Minnesota/23/2015	Minnesota Department of Health	Centers for Disease Control and Prevention	
EPI1033815	HE	United States	2015-Apr-14	EPI_ISL_272600	C/Minnesota/24/2015	Minnesota Department of Health	Centers for Disease Control and Prevention	
EPI1033816	MP	United States	2015-Apr-14	EPI_ISL_272600	C/Minnesota/24/2015	Minnesota Department of Health	Centers for Disease Control and Prevention	
EPI1033817	HE	United States	2015-Apr-14	EPI_ISL_272601	C/Minnesota/25/2015	Minnesota Department of Health	Centers for Disease Control and Prevention	
EPI1033818	MP	United States	2015-Apr-14	EPI_ISL_272601	C/Minnesota/25/2015	Minnesota Department of Health	Centers for Disease Control and Prevention	
EPI1033819	HE	United States	2015-Apr-15	EPI_ISL_272602	C/Minnesota/26/2015	Minnesota Department of Health	Centers for Disease Control and Prevention	
EPI1033820	MP	United States	2015-Apr-15	EPI_ISL_272602	C/Minnesota/26/2015	Minnesota Department of Health	Centers for Disease Control and Prevention	
EPI1033825	HE	United States	2015-Feb-04	EPI_ISL_272605	C/Minnesota/27/2015	Minnesota Department of Health	Centers for Disease Control and Prevention	
EPI1033826	MP	United States	2015-Feb-04	EPI_ISL_272605	C/Minnesota/27/2015	Minnesota Department of Health	Centers for Disease Control and Prevention	
EPI1033827	HE	United States	2015-Feb-08	EPI_ISL_272606	C/Minnesota/28/2015	Minnesota Department of Health	Centers for Disease Control and Prevention	
EPI1033828	MP	United States	2015-Feb-08	EPI_ISL_272606	C/Minnesota/28/2015	Minnesota Department of Health	Centers for Disease Control and Prevention	
EPI1033829	HE	United States	2015-Feb-23	EPI_ISL_272607	C/Minnesota/29/2015	Minnesota Department of Health	Centers for Disease Control and Prevention	
EPI1033830	MP	United States	2015-Feb-23	EPI_ISL_272607	C/Minnesota/29/2015	Minnesota Department of Health	Centers for Disease Control and Prevention	
EPI1033831	HE	United States	2015-Feb-23	EPI_ISL_272608	C/Minnesota/30/2015	Minnesota Department of Health	Centers for Disease Control and Prevention	
EPI1033832	MP	United States	2015-Feb-23	EPI_ISL_272608	C/Minnesota/30/2015	Minnesota Department of Health	Centers for Disease Control and Prevention	
EPI1033833	HE	United States	2015-Feb-28	EPI_ISL_272609	C/Minnesota/31/2015	Minnesota Department of Health	Centers for Disease Control and Prevention	
EPI1033834	MP	United States	2015-Feb-28	EPI_ISL_272609	C/Minnesota/31/2015	Minnesota Department of Health	Centers for Disease Control and Prevention	
EPI1033835	HE	United States	2015-Mar-02	EPI_ISL_272610	C/Minnesota/32/2015	Minnesota Department of Health	Centers for Disease Control and Prevention	
EPI1033836	MP	United States	2015-Mar-02	EPI_ISL_272610	C/Minnesota/32/2015	Minnesota Department of Health	Centers for Disease Control and Prevention	
EPI1033837	HE	United States	2015-Mar-11	EPI_ISL_272611	C/Minnesota/33/2015	Minnesota Department of Health	Centers for Disease Control and Prevention	
EPI1033838	MP	United States	2015-Mar-11	EPI_ISL_272611	C/Minnesota/33/2015	Minnesota Department of Health	Centers for Disease Control and Prevention	
EPI231536	HE	United States	1980-Jan-01	EPI_ISL_66335	C/Mississippi/80		Import from public-domain	Buonagurio,D.A.; Nakada,S.; Desselberger,U.; Krystal,M.; Palese,P.
EPI232094	MP	United States	1980-Jan-01	EPI_ISL_230286	C/Mississippi/80		Import from public-domain	Tada,Y.; Hongo,S.; Muraki,Y.; Sugawara,K.; Kitame,F.; Nakamura,K.
EPI816582	NP	United States	1980-Jan-01	EPI_ISL_230286	C/Mississippi/80		Import from public-domain	Tada,Y.; Hongo,S.; Muraki,Y.; Sugawara,K.; Kitame,F.; Nakamura,K.
EPI816740	NS	United States	1980-Jan-01	EPI_ISL_230286	C/Mississippi/80		Import from public-domain	Tada,Y.; Hongo,S.; Muraki,Y.; Sugawara,K.; Kitame,F.; Nakamura,K.
EPI816483	P3	United States	1980-Jan-01	EPI_ISL_230286	C/Mississippi/80		Import from public-domain	Tada,Y.; Hongo,S.; Muraki,Y.; Sugawara,K.; Kitame,F.; Nakamura,K.
EPI815430	PB1	United States	1980-Jan-01	EPI_ISL_230286	C/Mississippi/80		Import from public-domain	Tada,Y.; Hongo,S.; Muraki,Y.; Sugawara,K.; Kitame,F.; Nakamura,K.
EPI814537	PB2	United States	1980-Jan-01	EPI_ISL_230286	C/Mississippi/80		Import from public-domain	Tada,Y.; Hongo,S.; Muraki,Y.; Sugawara,K.; Kitame,F.; Nakamura,K.
EPI231660	HE	Japan	1990-Jan-01	EPI_ISL_66355	C/Miyagi/1/90		Import from public-domain	Matsuzaki,Y.
EPI231665	MP	Japan	1990-Jan-01	EPI_ISL_66355	C/Miyagi/1/90		Import from public-domain	Matsuzaki,Y.
EPI231566	NS	Japan	1990-Jan-01	EPI_ISL_66355	C/Miyagi/1/90		Import from public-domain	Matsuzaki,Y.
EPI231666	HE	Japan	1993-Feb-02	EPI_ISL_66378	C/Miyagi/1/93		Import from public-domain	Matsuzaki,Y.; Sugawara,K.; Furuse,Y.; Shimotai,Y.; Hongo,S.; Oshitani,H.; Mizuta,K.; Nishimura,H.
EPI231671	MP	Japan	1993-Feb-02	EPI_ISL_66378	C/Miyagi/1/93		Import from public-domain	Matsuzaki,Y.; Sugawara,K.; Furuse,Y.; Shimotai,Y.; Hongo,S.; Oshitani,H.; Mizuta,K.; Nishimura,H.
EPI816604	NP	Japan	1993-Feb-02	EPI_ISL_66378	C/Miyagi/1/93		Import from public-domain	Matsuzaki,Y.; Sugawara,K.; Furuse,Y.; Shimotai,Y.; Hongo,S.; Oshitani,H.; Mizuta,K.; Nishimura,H.
EPI231672	NS	Japan	1993-Feb-02	EPI_ISL_66378	C/Miyagi/1/93		Import from public-domain	Matsuzaki,Y.; Sugawara,K.; Furuse,Y.; Shimotai,Y.; Hongo,S.; Oshitani,H.; Mizuta,K.; Nishimura,H.
EPI816499	P3	Japan	1993-Feb-02	EPI_ISL_66378	C/Miyagi/1/93		Import from public-domain	Matsuzaki,Y.; Sugawara,K.; Furuse,Y.; Shimotai,Y.; Hongo,S.; Oshitani,H.; Mizuta,K.; Nishimura,H.
EPI816416	PB1	Japan	1993-Feb-02	EPI_ISL_66378	C/Miyagi/1/93		Import from public-domain	Matsuzaki,Y.; Sugawara,K.; Furuse,Y.; Shimotai,Y.; Hongo,S.; Oshitani,H.; Mizuta,K.; Nishimura,H.
EPI813731	PB2	Japan	1993-Feb-02	EPI_ISL_66378	C/Miyagi/1/93		Import from public-domain	Matsuzaki,Y.; Sugawara,K.; Furuse,Y.; Shimotai,Y.; Hongo,S.; Oshitani,H.; Mizuta,K.; Nishimura,H.
EPI231673	HE	Japan	1994-Jan-01	EPI_ISL_66379	C/Miyagi/1/94		Import from public-domain	Matsuzaki,Y.
EPI231678	MP	Japan	1994-Jan-01	EPI_ISL_66379	C/Miyagi/1/94		Import from public-domain	Matsuzaki,Y.
EPI231679	NS	Japan	1994-Jan-01	EPI_ISL_66379	C/Miyagi/1/94		Import from public-domain	Matsuzaki,Y.
EPI231680	HE	Japan	1997-Jan-01	EPI_ISL_66380	C/Miyagi/1/97		Import from public-domain	Matsuzaki,Y.
EPI231685	MP	Japan	1997-Jan-01	EPI_ISL_66380	C/Miyagi/1/97		Import from public-domain	Matsuzaki,Y.
EPI231686	NS	Japan	1997-Jan-01	EPI_ISL_66380	C/Miyagi/1/97		Import from public-domain	Matsuzaki,Y.
EPI231687	HE	Japan	1999-Jan-01	EPI_ISL_66381	C/Miyagi/1/99		Import from public-domain	Matsuzaki,Y.
EPI231692	MP	Japan	1999-Jan-01	EPI_ISL_66381	C/Miyagi/1/99		Import from public-domain	Matsuzaki,Y.
EPI231693	NS	Japan	1999-Jan-01	EPI_ISL_66381	C/Miyagi/1/99		Import from public-domain	Matsuzaki,Y.
EPI813654	HE	Japan	2002-Feb-22	EPI_ISL_230239	C/Miyagi/11/2002		Import from public-domain	Matsuzaki,Y.; Sugawara,K.; Furuse,Y.; Shimotai,Y.; Hongo,S.; Oshitani,H.; Mizuta,K.; Nishimura,H.
EPI816679	MP	Japan	2002-Feb-22	EPI_ISL_230239	C/Miyagi/11/2002		Import from public-domain	Matsuzaki,Y.; Sugawara,K.; Furuse,Y.; Shimotai,Y.; Hongo,S.; Oshitani,H.; Mizuta,K.; Nishimura,H.
EPI816615	NP	Japan	2002-Feb-22	EPI_ISL_230239	C/Miyagi/11/2002		Import from public-domain	Matsuzaki,Y.; Sugawara,K.; Furuse,Y.; Shimotai,Y.; Hongo,S.; Oshitani,H.; Mizuta,K.; Nishimura,H.
EPI816748	NS	Japan	2002-Feb-22	EPI_ISL_230239	C/Miyagi/11/2002		Import from public-domain	Matsuzaki,Y.; Sugawara,K.; Furuse,Y.; Shimotai,Y.; Hongo,S.; Oshitani,H.; Mizuta,K.; Nishimura,H.
EPI816512	P3	Japan	2002-Feb-22	EPI_ISL_230239	C/Miyagi/11/2002		Import from public-domain	Matsuzaki,Y.; Sugawara,K.; Furuse,Y.; Shimotai,Y.; Hongo,S.; Oshitani,H.; Mizuta,K.; Nishimura,H.
EPI813799	PB1	Japan	2002-Feb-22	EPI_ISL_230239	C/Miyagi/11/2002		Import from public-domain	Matsuzaki,Y.; Sugawara,K.; Furuse,Y.; Shimotai,Y.; Hongo,S.; Oshitani,H.; Mizuta,K.; Nishimura,H.
EPI813702	PB2	Japan	2002-Feb-22	EPI_ISL_230239	C/Miyagi/11/2002		Import from public-domain	Matsuzaki,Y.; Sugawara,K.; Furuse,Y.; Shimotai,Y.; Hongo,S.; Oshitani,H.; Mizuta,K.; Nishimura,H.
EPI228322	HE	Japan	2004-Feb-27	EPI_ISL_65158	C/Miyagi/12/2004		Import from public-domain	Matsuzaki,Y.; Sugawara,K.; Furuse,Y.; Shimotai,Y.; Hongo,S.; Oshitani,H.; Mizuta,K.; Nishimura,H.
EPI816689	MP	Japan	2004-Feb-27	EPI_ISL_65158	C/Miyagi/12/2004		Import from public-domain	Matsuzaki,Y.; Sugawara,K.; Furuse,Y.; Shimotai,Y.; Hongo,S.; Oshitani,H.; Mizuta,K.; Nishimura,H.
EPI816624	NP	Japan	2004-Feb-27	EPI_ISL_65158	C/Miyagi/12/2004		Import from public-domain	Matsuzaki,Y.; Sugawara,K.; Furuse,Y.; Shimotai,Y.; Hongo,S.; Oshitani,H.; Mizuta,K.; Nishimura,H.

Segment ID	Segment	Country	Collection date	Isolate-ID	Isolate name	Originating Lab	Submitting Lab	Authors
EPI816758	NS	Japan	2004-Feb-27	EPI_ISL_65158	C/Miyagi/12/2004		Import from public-domain	Matsuzaki,Y.; Sugawara,K.; Furuse,Y.; Shimotai,Y.; Hongo,S.; Oshitani,H.; Mizuta,K.; Nishimura,H.
EPI816522	P3	Japan	2004-Feb-27	EPI_ISL_65158	C/Miyagi/12/2004		Import from public-domain	Matsuzaki,Y.; Sugawara,K.; Furuse,Y.; Shimotai,Y.; Hongo,S.; Oshitani,H.; Mizuta,K.; Nishimura,H.
EPI816429	PB1	Japan	2004-Feb-27	EPI_ISL_65158	C/Miyagi/12/2004		Import from public-domain	Matsuzaki,Y.; Sugawara,K.; Furuse,Y.; Shimotai,Y.; Hongo,S.; Oshitani,H.; Mizuta,K.; Nishimura,H.
EPI813733	PB2	Japan	2004-Feb-27	EPI_ISL_65158	C/Miyagi/12/2004		Import from public-domain	Matsuzaki,Y.; Sugawara,K.; Furuse,Y.; Shimotai,Y.; Hongo,S.; Oshitani,H.; Mizuta,K.; Nishimura,H.
EPI231696	HE	Japan	2000-Jan-01	EPI_ISL_66382	C/Miyagi/2/2000		Import from public-domain	Matsuzaki,Y.
EPI231694	MP	Japan	2000-Jan-01	EPI_ISL_66382	C/Miyagi/2/2000		Import from public-domain	Matsuzaki,Y.
EPI231695	NS	Japan	2000-Jan-01	EPI_ISL_66382	C/Miyagi/2/2000		Import from public-domain	Matsuzaki,Y.
EPI813665	HE	Japan	2005-Apr-07	EPI_ISL_230250	C/Miyagi/2/2005		Import from public-domain	Matsuzaki,Y.; Sugawara,K.; Furuse,Y.; Shimotai,Y.; Hongo,S.; Oshitani,H.; Mizuta,K.; Nishimura,H.
EPI816696	MP	Japan	2005-Apr-07	EPI_ISL_230250	C/Miyagi/2/2005		Import from public-domain	Matsuzaki,Y.; Sugawara,K.; Furuse,Y.; Shimotai,Y.; Hongo,S.; Oshitani,H.; Mizuta,K.; Nishimura,H.
EPI816630	NP	Japan	2005-Apr-07	EPI_ISL_230250	C/Miyagi/2/2005		Import from public-domain	Matsuzaki,Y.; Sugawara,K.; Furuse,Y.; Shimotai,Y.; Hongo,S.; Oshitani,H.; Mizuta,K.; Nishimura,H.
EPI816765	NS	Japan	2005-Apr-07	EPI_ISL_230250	C/Miyagi/2/2005		Import from public-domain	Matsuzaki,Y.; Sugawara,K.; Furuse,Y.; Shimotai,Y.; Hongo,S.; Oshitani,H.; Mizuta,K.; Nishimura,H.
EPI816529	P3	Japan	2005-Apr-07	EPI_ISL_230250	C/Miyagi/2/2005		Import from public-domain	Matsuzaki,Y.; Sugawara,K.; Furuse,Y.; Shimotai,Y.; Hongo,S.; Oshitani,H.; Mizuta,K.; Nishimura,H.
EPI813811	PB1	Japan	2005-Apr-07	EPI_ISL_230250	C/Miyagi/2/2005		Import from public-domain	Matsuzaki,Y.; Sugawara,K.; Furuse,Y.; Shimotai,Y.; Hongo,S.; Oshitani,H.; Mizuta,K.; Nishimura,H.
EPI813714	PB2	Japan	2005-Apr-07	EPI_ISL_230250	C/Miyagi/2/2005		Import from public-domain	Matsuzaki,Y.; Sugawara,K.; Furuse,Y.; Shimotai,Y.; Hongo,S.; Oshitani,H.; Mizuta,K.; Nishimura,H.
EPI813678	HE	Japan	2008-Feb-21	EPI_ISL_230263	C/Miyagi/2/2008		Import from public-domain	Matsuzaki,Y.; Sugawara,K.; Furuse,Y.; Shimotai,Y.; Hongo,S.; Oshitani,H.; Mizuta,K.; Nishimura,H.
EPI816711	MP	Japan	2008-Feb-21	EPI_ISL_230263	C/Miyagi/2/2008		Import from public-domain	Matsuzaki,Y.; Sugawara,K.; Furuse,Y.; Shimotai,Y.; Hongo,S.; Oshitani,H.; Mizuta,K.; Nishimura,H.
EPI816644	NP	Japan	2008-Feb-21	EPI_ISL_230263	C/Miyagi/2/2008		Import from public-domain	Matsuzaki,Y.; Sugawara,K.; Furuse,Y.; Shimotai,Y.; Hongo,S.; Oshitani,H.; Mizuta,K.; Nishimura,H.
EPI816780	NS	Japan	2008-Feb-21	EPI_ISL_230263	C/Miyagi/2/2008		Import from public-domain	Matsuzaki,Y.; Sugawara,K.; Furuse,Y.; Shimotai,Y.; Hongo,S.; Oshitani,H.; Mizuta,K.; Nishimura,H.
EPI816544	P3	Japan	2008-Feb-21	EPI_ISL_230263	C/Miyagi/2/2008		Import from public-domain	Matsuzaki,Y.; Sugawara,K.; Furuse,Y.; Shimotai,Y.; Hongo,S.; Oshitani,H.; Mizuta,K.; Nishimura,H.
EPI816440	PB1	Japan	2008-Feb-21	EPI_ISL_230263	C/Miyagi/2/2008		Import from public-domain	Matsuzaki,Y.; Sugawara,K.; Furuse,Y.; Shimotai,Y.; Hongo,S.; Oshitani,H.; Mizuta,K.; Nishimura,H.
EPI813750	PB2	Japan	2008-Feb-21	EPI_ISL_230263	C/Miyagi/2/2008		Import from public-domain	Matsuzaki,Y.; Sugawara,K.; Furuse,Y.; Shimotai,Y.; Hongo,S.; Oshitani,H.; Mizuta,K.; Nishimura,H.
EPI813686	HE	Japan	2012-Jan-26	EPI_ISL_230271	C/Miyagi/2/2012		Import from public-domain	Matsuzaki,Y.; Sugawara,K.; Furuse,Y.; Shimotai,Y.; Hongo,S.; Oshitani,H.; Mizuta,K.; Nishimura,H.
EPI816722	MP	Japan	2012-Jan-26	EPI_ISL_230271	C/Miyagi/2/2012		Import from public-domain	Matsuzaki,Y.; Sugawara,K.; Furuse,Y.; Shimotai,Y.; Hongo,S.; Oshitani,H.; Mizuta,K.; Nishimura,H.
EPI816654	NP	Japan	2012-Jan-26	EPI_ISL_230271	C/Miyagi/2/2012		Import from public-domain	Matsuzaki,Y.; Sugawara,K.; Furuse,Y.; Shimotai,Y.; Hongo,S.; Oshitani,H.; Mizuta,K.; Nishimura,H.
EPI816791	NS	Japan	2012-Jan-26	EPI_ISL_230271	C/Miyagi/2/2012		Import from public-domain	Matsuzaki,Y.; Sugawara,K.; Furuse,Y.; Shimotai,Y.; Hongo,S.; Oshitani,H.; Mizuta,K.; Nishimura,H.
EPI816555	P3	Japan	2012-Jan-26	EPI_ISL_230271	C/Miyagi/2/2012		Import from public-domain	Matsuzaki,Y.; Sugawara,K.; Furuse,Y.; Shimotai,Y.; Hongo,S.; Oshitani,H.; Mizuta,K.; Nishimura,H.
EPI816451	PB1	Japan	2012-Jan-26	EPI_ISL_230271	C/Miyagi/2/2012		Import from public-domain	Matsuzaki,Y.; Sugawara,K.; Furuse,Y.; Shimotai,Y.; Hongo,S.; Oshitani,H.; Mizuta,K.; Nishimura,H.
EPI813761	PB2	Japan	2012-Jan-26	EPI_ISL_230271	C/Miyagi/2/2012		Import from public-domain	Matsuzaki,Y.; Sugawara,K.; Furuse,Y.; Shimotai,Y.; Hongo,S.; Oshitani,H.; Mizuta,K.; Nishimura,H.
EPI813688	HE	Japan	2014-Apr-30	EPI_ISL_230273	C/Miyagi/2/2014		Import from public-domain	Matsuzaki,Y.; Sugawara,K.; Furuse,Y.; Shimotai,Y.; Hongo,S.; Oshitani,H.; Mizuta,K.; Nishimura,H.
EPI816731	MP	Japan	2014-Apr-30	EPI_ISL_230273	C/Miyagi/2/2014		Import from public-domain	Matsuzaki,Y.; Sugawara,K.; Furuse,Y.; Shimotai,Y.; Hongo,S.; Oshitani,H.; Mizuta,K.; Nishimura,H.
EPI816663	NP	Japan	2014-Apr-30	EPI_ISL_230273	C/Miyagi/2/2014		Import from public-domain	Matsuzaki,Y.; Sugawara,K.; Furuse,Y.; Shimotai,Y.; Hongo,S.; Oshitani,H.; Mizuta,K.; Nishimura,H.
EPI816800	NS	Japan	2014-Apr-30	EPI_ISL_230273	C/Miyagi/2/2014		Import from public-domain	Matsuzaki,Y.; Sugawara,K.; Furuse,Y.; Shimotai,Y.; Hongo,S.; Oshitani,H.; Mizuta,K.; Nishimura,H.
EPI816564	P3	Japan	2014-Apr-30	EPI_ISL_230273	C/Miyagi/2/2014		Import from public-domain	Matsuzaki,Y.; Sugawara,K.; Furuse,Y.; Shimotai,Y.; Hongo,S.; Oshitani,H.; Mizuta,K.; Nishimura,H.
EPI816453	PB1	Japan	2014-Apr-30	EPI_ISL_230273	C/Miyagi/2/2014		Import from public-domain	Matsuzaki,Y.; Sugawara,K.; Furuse,Y.; Shimotai,Y.; Hongo,S.; Oshitani,H.; Mizuta,K.; Nishimura,H.
EPI813763	PB2	Japan	2014-Apr-30	EPI_ISL_230273	C/Miyagi/2/2014		Import from public-domain	Matsuzaki,Y.; Sugawara,K.; Furuse,Y.; Shimotai,Y.; Hongo,S.; Oshitani,H.; Mizuta,K.; Nishimura,H.
EPI232105	HE	Japan	1992-Jan-01	EPI_ISL_66383	C/Miyagi/2/92		Import from public-domain	Matsuzaki,Y.
EPI231701	MP	Japan	1992-Jan-01	EPI_ISL_66383	C/Miyagi/2/92		Import from public-domain	Matsuzaki,Y.
EPI231702	NS	Japan	1992-Jan-01	EPI_ISL_66383	C/Miyagi/2/92		Import from public-domain	Matsuzaki,Y.
EPI232106	HE	Japan	1993-Jan-01	EPI_ISL_66384	C/Miyagi/2/93		Import from public-domain	Matsuzaki,Y.
EPI231706	MP	Japan	1993-Jan-01	EPI_ISL_66384	C/Miyagi/2/93		Import from public-domain	Matsuzaki,Y.
EPI231705	NS	Japan	1993-Jan-01	EPI_ISL_66384	C/Miyagi/2/93		Import from public-domain	Matsuzaki,Y.
EPI231710	HE	Japan	1994-Jan-01	EPI_ISL_66385	C/Miyagi/2/94		Import from public-domain	Matsuzaki,Y.
EPI231711	MP	Japan	1994-Jan-01	EPI_ISL_66385	C/Miyagi/2/94		Import from public-domain	Matsuzaki,Y.
EPI231715	NS	Japan	1994-Jan-01	EPI_ISL_66385	C/Miyagi/2/94		Import from public-domain	Matsuzaki,Y.
EPI231720	HE	Japan	1996-Jan-01	EPI_ISL_66386	C/Miyagi/2/96		Import from public-domain	Matsuzaki,Y.
EPI231722	MP	Japan	1996-Jan-01	EPI_ISL_66386	C/Miyagi/2/96		Import from public-domain	Matsuzaki,Y.
EPI231716	NS	Japan	1996-Jan-01	EPI_ISL_66386	C/Miyagi/2/96		Import from public-domain	Matsuzaki,Y.
EPI231728	HE	Japan	1998-Jan-01	EPI_ISL_66387	C/Miyagi/2/98		Import from public-domain	Matsuzaki,Y.
EPI231727	MP	Japan	1998-Jan-01	EPI_ISL_66387	C/Miyagi/2/98		Import from public-domain	Matsuzaki,Y.
EPI231723	NS	Japan	1998-Jan-01	EPI_ISL_66387	C/Miyagi/2/98		Import from public-domain	Matsuzaki,Y.
EPI813655	HE	Japan	2002-Mar-17	EPI_ISL_230240	C/Miyagi/20/2002		Import from public-domain	Matsuzaki,Y.; Sugawara,K.; Furuse,Y.; Shimotai,Y.; Hongo,S.; Oshitani,H.; Mizuta,K.; Nishimura,H.
EPI816680	MP	Japan	2002-Mar-17	EPI_ISL_230240	C/Miyagi/20/2002		Import from public-domain	Matsuzaki,Y.; Sugawara,K.; Furuse,Y.; Shimotai,Y.; Hongo,S.; Oshitani,H.; Mizuta,K.; Nishimura,H.
EPI816616	NP	Japan	2002-Mar-17	EPI_ISL_230240	C/Miyagi/20/2002		Import from public-domain	Matsuzaki,Y.; Sugawara,K.; Furuse,Y.; Shimotai,Y.; Hongo,S.; Oshitani,H.; Mizuta,K.; Nishimura,H.
EPI816749	NS	Japan	2002-Mar-17	EPI_ISL_230240	C/Miyagi/20/2002		Import from public-domain	Matsuzaki,Y.; Sugawara,K.; Furuse,Y.; Shimotai,Y.; Hongo,S.; Oshitani,H.; Mizuta,K.; Nishimura,H.
EPI816513	P3	Japan	2002-Mar-17	EPI_ISL_230240	C/Miyagi/20/2002		Import from public-domain	Matsuzaki,Y.; Sugawara,K.; Furuse,Y.; Shimotai,Y.; Hongo,S.; Oshitani,H.; Mizuta,K.; Nishimura,H.
EPI813800	PB1	Japan	2002-Mar-17	EPI_ISL_230240	C/Miyagi/20/2002		Import from public-domain	Matsuzaki,Y.; Sugawara,K.; Furuse,Y.; Shimotai,Y.; Hongo,S.; Oshitani,H.; Mizuta,K.; Nishimura,H.
EPI813703	PB2	Japan	2002-Mar-17	EPI_ISL_230240	C/Miyagi/20/2002		Import from public-domain	Matsuzaki,Y.; Sugawara,K.; Furuse,Y.; Shimotai,Y.; Hongo,S.; Oshitani,H.; Mizuta,K.; Nishimura,H.
EPI813660	HE	Japan	2004-Mar-31	EPI_ISL_230245	C/Miyagi/25/2004		Import from public-domain	Matsuzaki,Y.; Sugawara,K.; Furuse,Y.; Shimotai,Y.; Hongo,S.; Oshitani,H.; Mizuta,K.; Nishimura,H.
EPI816690	MP	Japan	2004-Mar-31	EPI_ISL_230245	C/Miyagi/25/2004		Import from public-domain	Matsuzaki,Y.; Sugawara,K.; Furuse,Y.; Shimotai,Y.; Hongo,S.; Oshitani,H.; Mizuta,K.; Nishimura,H.
EPI816625	NP	Japan	2004-Mar-31	EPI_ISL_230245	C/Miyagi/25/2004		Import from public-domain	Matsuzaki,Y.; Sugawara,K.; Furuse,Y.; Shimotai,Y.; Hongo,S.; Oshitani,H.; Mizuta,K.; Nishimura,H.
EPI816759	NS	Japan	2004-Mar-31	EPI_ISL_230245	C/Miyagi/25/2004		Import from public-domain	Matsuzaki,Y.; Sugawara,K.; Furuse,Y.; Shimotai,Y.; Hongo,S.; Oshitani,H.; Mizuta,K.; Nishimura,H.
EPI816523	P3	Japan	2004-Mar-31	EPI_ISL_230245	C/Miyagi/25/2004		Import from public-domain	Matsuzaki,Y.; Sugawara,K.; Furuse,Y.; Shimotai,Y.; Hongo,S.; Oshitani,H.; Mizuta,K.; Nishimura,H.
EPI813806	PB1	Japan	2004-Mar-31	EPI_ISL_230245	C/Miyagi/25/2004		Import from public-domain	Matsuzaki,Y.; Sugawara,K.; Furuse,Y.; Shimotai,Y.; Hongo,S.; Oshitani,H.; Mizuta,K.; Nishimura,H.
EPI813709	PB2	Japan	2004-Mar-31	EPI_ISL_230245	C/Miyagi/25/2004		Import from public-domain	Matsuzaki,Y.; Sugawara,K.; Furuse,Y.; Shimotai,Y.; Hongo,S.; Oshitani,H.; Mizuta,K.; Nishimura,H.
EPI813656	HE	Japan	2002-Apr-22	EPI_ISL_230241	C/Miyagi/28/2002		Import from public-domain	Matsuzaki,Y.; Sugawara,K.; Furuse,Y.; Shimotai,Y.; Hongo,S.; Oshitani,H.; Mizuta,K.; Nishimura,H.
EPI816681	MP	Japan	2002-Apr-22	EPI_ISL_230241	C/Miyagi/28/2002		Import from public-domain	Matsuzaki,Y.; Sugawara,K.; Furuse,Y.; Shimotai,Y.; Hongo,S.; Oshitani,H.; Mizuta,K.; Nishimura,H.
EPI816617	NP	Japan	2002-Apr-22	EPI_ISL_230241	C/Miyagi/28/2002		Import from public-domain	Matsuzaki,Y.; Sugawara,K.; Furuse,Y.; Shimotai,Y.; Hongo,S.; Oshitani,H.; Mizuta,K.; Nishimura,H.
EPI816750	NS	Japan	2002-Apr-22	EPI_ISL_230241	C/Miyagi/28/2002		Import from public-domain	Matsuzaki,Y.; Sugawara,K.; Furuse,Y.; Shimotai,Y.; Hongo,S.; Oshitani,H.; Mizuta,K.; Nishimura,H.
EPI816514	P3	Japan	2002-Apr-22	EPI_ISL_230241	C/Miyagi/28/2002		Import from public-domain	Matsuzaki,Y.; Sugawara,K.; Furuse,Y.; Shimotai,Y.; Hongo,S.; Oshitani,H.; Mizuta,K.; Nishimura,H.
EPI813801	PB1	Japan	2002-Apr-22	EPI_ISL_230241	C/Miyagi/28/2002		Import from public-domain	Matsuzaki,Y.; Sugawara,K.; Furuse,Y.; Shimotai,Y.; Hongo,S.; Oshitani,H.; Mizuta,K.; Nishimura,H.
EPI813704	PB2	Japan	2002-Apr-22	EPI_ISL_230241	C/Miyagi/28/2002		Import from public-domain	Matsuzaki,Y.; Sugawara,K.; Furuse,Y.; Shimotai,Y.; Hongo,S.; Oshitani,H.; Mizuta,K.; Nishimura,H.
EPI231730	HE	Japan	2000-Jan-01	EPI_ISL_66388	C/Miyagi/3/2000		Import from public-domain	Matsuzaki,Y.
EPI231736	MP	Japan	2000-Jan-01	EPI_ISL_66388	C/Miyagi/3/2000		Import from public-domain	Matsuzaki,Y.
EPI231735	NS	Japan	2000-Jan-01	EPI_ISL_66388	C/Miyagi/3/2000		Import from public-domain	Matsuzaki,Y.
EPI813679	HE	Japan	2010-Apr-12	EPI_ISL_230264	C/Miyagi/3/2010		Import from public-domain	Matsuzaki,Y.; Sugawara,K.; Furuse,Y.; Shimotai,Y.; Hongo,S.; Oshitani,H.; Mizuta,K.; Nishimura,H.
EPI816713	MP	Japan	2010-Apr-12	EPI_ISL_230264	C/Miyagi/3/2010		Import from public-domain	Matsuzaki,Y.; Sugawara,K.; Furuse,Y.; Shimotai,Y.; Hongo,S.; Oshitani,H.; Mizuta,K.; Nishimura,H.
EPI816645	NP	Japan	2010-Apr-12	EPI_ISL_230264	C/Miyagi/3/2010		Import from public-domain	Matsuzaki,Y.; Sugawara,K.; Furuse,Y.; Shimotai,Y.; Hongo,S.; Oshitani,H.; Mizuta,K.; Nishimura,H.
EPI816782	NS	Japan	2010-Apr-12	EPI_ISL_230264	C/Miyagi/3/2010		Import from public-domain	Matsuzaki,Y.; Sugawara,K.; Furuse,Y.; Shimotai,Y.; Hongo,S.; Oshitani,H.; Mizuta,K.; Nishimura,H.
EPI816546	P3	Japan	2010-Apr-12	EPI_ISL_230264	C/Miyagi/3/2010		Import from public-domain	Matsuzaki,Y.; Sugawara,K.; Furuse,Y.; Shimotai,Y.; Hongo,S.; Oshitani,H.; Mizuta,K.; Nishimura,H.
EPI816442	PB1	Japan	2010-Apr-12	EPI_ISL_230264	C/Miyagi/3/2010		Import from public-domain	Matsuzaki,Y.; Sugawara,K.; Furuse,Y.; Shimotai,Y.; Hongo,S.; Oshitani,H.; Mizuta,K.; Nishimura,H.
EPI813752	PB2	Japan	2010-Apr-12	EPI_ISL_230264	C/Miyagi/3/2010		Import from public-domain	Matsuzaki,Y.; Sugawara,K.; Furuse,Y.; Shimotai,Y.; Hongo,S.; Oshitani,H.; Mizuta,K.; Nishimura,H.
EPI231737	HE	Japan	1991-Jan-01	EPI_ISL_66389	C/Miyagi/3/91		Import from public-domain	Kimura,H.
EPI231744	HE	Japan	1992-Jan-01	EPI_ISL_66390	C/Miyagi/3/92		Import from public-domain	Matsuzaki,Y.
EPI231747	HE	Japan	1993-Apr-13	EPI_ISL_66391	C/Miyagi/3/93		Import from public-domain	Matsuzaki,Y.; Sugawara,K.; Furuse,Y.; Shimotai,Y.; Hongo,S.; Oshitani,H.; Mizuta,K.; Nishimura,H.
EPI231750	MP	Japan	1993-Apr-13	EPI_ISL_66391	C/Miyagi/3/93		Import from public-domain	Matsuzaki,Y.; Sugawara,K.; Furuse,Y.; Shimotai,Y.; Hongo,S.; Oshitani,H.; Mizuta,K.; Nishimura,H.
EPI816605	NP	Japan	1993-Apr-13	EPI_ISL_66391	C/Miyagi/3/93		Import from public-domain	Matsuzaki,Y.; Sugawara,K.; Furuse,Y.; Shimotai,Y.; Hongo,S.; Oshitani,H.; Mizuta,K.; Nishimura,H.
EPI231751	NS	Japan	1993-Apr-13	EPI_ISL_66391	C/Miyagi/3/93		Import from public-domain	Matsuzaki,Y.; Sugawara,K.; Furuse,Y.; Shimotai,Y.; Hongo,S.; Oshitani,H.; Mizuta,K.; Nishimura,H.
EPI816500	P3	Japan	1993-Apr-13	EPI_ISL_66391	C/Miyagi/3/93		Import from public-domain	Matsuzaki,Y.; Sugawara,K.; Furuse,Y.; Shimotai,Y.; Hongo,S.; Oshitani,H.; Mizuta,K.; Nishimura,H.
EPI816417	PB1	Japan	1993-Apr-13	EPI_ISL_66391	C/Miyagi/3/93		Import from public-domain	Matsuzaki,Y.; Sugawara,K.; Furuse,Y.; Shimotai,Y.; Hongo,S.; Oshitani,H.; Mizuta,K.; Nishimura,H.
EPI813729	PB2	Japan	1993-Apr-13	EPI_ISL_66391	C/Miyagi/3/93		Import from public-domain	Matsuzaki,Y.; Sugawara,K.; Furuse,Y.; Shimotai,Y.; Hongo,S.; Oshitani,H.; Mizuta,K.; Nishimura,H.
EPI231756	HE	Japan	1994-Jan-01	EPI_ISL_66392	C/Miyagi/3/94		Import from public-domain	Matsuzaki,Y.
EPI231755	MP	Japan	1994-Jan-01	EPI_ISL_66392	C/Miyagi/3/94		Import from public-domain	Matsuzaki,Y.
EPI231758	NS	Japan	1994-Jan-01	EPI_ISL_66392	C/Miyagi/3/94		Import from public-domain	Matsuzaki,Y.
EPI231759	HE	Japan	1997-Jan-01	EPI_ISL_66393	C/Miyagi/3/97		Import from public-domain	Matsuzaki,Y.
EPI231764	HE	Japan	1999-Jan-01	EPI_ISL_66394	C/Miyagi/3/99		Import from public-domain	Matsuzaki,Y.
EPI231761	MP	Japan	1999-Jan-01	EPI_ISL_66394	C/Miyagi/3/99		Import from public-domain	Matsuzaki,Y.
EPI231760	NS	Japan	1999-Jan-01	EPI_ISL_66394	C/Miyagi/3/99		Import from public-domain	Matsuzaki,Y.
EPI813657	HE	Japan	2002-Jun-18	EPI_ISL_230242	C/Miyagi/31/2002		Import from public-domain	Matsuzaki,Y.; Sugawara,K.; Furuse,Y.; Shimotai,Y.; Hongo,S.; Oshitani,H.; Mizuta,K.; Nishimura,H.
EPI816682	MP	Japan	2002-Jun-18	EPI_ISL_230242	C/Miyagi/31/2002		Import from public-domain	Matsuzaki,Y.; Sugawara,K.; Furuse,Y.; Shimotai,Y.; Hongo,S.; Oshitani,H.; Mizuta,K.; Nishimura,H.
EPI816618	NP	Japan	2002-Jun-18	EPI_ISL_230242	C/Miyagi/31/2002		Import from public-domain	Matsuzaki,Y.; Sugawara,K.; Furuse,Y.; Shimotai,Y.; Hongo,S.; Oshitani,H.; Mizuta,K.; Nishimura,H.
EPI816751	NS	Japan	2002-Jun-18	EPI_ISL_230242	C/Miyagi/31/2002		Import from public-domain	Matsuzaki,Y.; Sugawara,K.; Furuse,Y.; Shimotai,Y.; Hongo,S.; Oshitani,H.; Mizuta,K.; Nishimura,H.
EPI816515	P3	Japan	2002-Jun-18	EPI_ISL_230242	C/Miyagi/31/2002		Import from public-domain	Matsuzaki,Y.; Sugawara,K.; Furuse,Y.; Shimotai,Y.; Hongo,S.; Oshitani,H.; Mizuta,K.; Nishimura,H.
EPI813802	PB1	Japan	2002-Jun-18	EPI_ISL_230242	C/Miyagi/31/2002		Import from public-domain	Matsuzaki,Y.; Sugawara,K.; Furuse,Y.; Shimotai,Y.; Hongo,S.; Oshitani,H.; Mizuta,K.; Nishimura,H.
EPI813705	PB2	Japan	2002-Jun-18	EPI_ISL_230242	C/Miyagi/31/2002		Import from public-domain	Matsuzaki,Y.; Sugawara,K.; Furuse,Y.; Shimotai,Y.; Hongo,S.; Oshitani,H.; Mizuta,K.; Nishimura,H.
EPI231767	HE	Japan	2000-Jan-01	EPI_ISL_66395	C/Miyagi/4/2000		Import from public-domain	Matsuzaki,Y.
EPI231772	MP	Japan	2000-Jan-01	EPI_ISL_66395	C/Miyagi/4/2000		Import from public-domain	Matsuzaki,Y.
EPI231773	NS	Japan	2000-Jan-01	EPI_ISL_66395	C/Miyagi/4/2000		Import from public-domain	Matsuzaki,Y.
EPI813653	HE	Japan	2002-Jan-24	EPI_ISL_230238	C/Miyagi/4/2002		Import from public-domain	Matsuzaki,Y.; Sugawara,K.; Furuse,Y.; Shimotai,Y.; Hongo,S.; Oshitani,H.; Mizuta,K.; Nishimura,H.
EPI816678	MP	Japan	2002-Jan-24	EPI_ISL_230238	C/Miyagi/4/2002		Import from public-domain	Matsuzaki,Y.; Sugawara,K.; Furuse,Y.; Shimotai,Y.; Hongo,S.; Oshitani,H.; Mizuta,K.; Nishimura,H.
EPI369791	NP	Japan	2002-Jan-24	EPI_ISL_118426	C/Miyagi/4/2002		Import from public-domain	Matsuzaki,Y.; Ikeda,T.; Abiko,C.; Aoki,Y.; Mizuta,K.; Shimotai,Y.; Sugawara,K.; Hongo,S.
EPI816747	NS	Japan	2002-Jan-24	EPI_ISL_230238	C/Miyagi/4/2002		Import from public-domain	Matsuzaki,Y.; Sugawara,K.; Furuse,Y.; Shimotai,Y.; Hongo,S.; Oshitani,H.; Mizuta,K.; Nishimura,H.

Segment ID	Segment	Country	Collection date	Isolate-ID	Isolate name	Originating Lab	Submitting Lab	Authors
EPI816511	P3	Japan	2002-Jan-24	EPI_ISL_230238	C/Miyagi/4/2002		Import from public-domain	Matsuzaki,Y.; Sugawara,K.; Furuse,Y.; Shimotai,Y.; Hongo,S.; Oshitani,H.; Mizuta,K.; Nishimura,H.
EPI813798	PB1	Japan	2002-Jan-24	EPI_ISL_230238	C/Miyagi/4/2002		Import from public-domain	Matsuzaki,Y.; Sugawara,K.; Furuse,Y.; Shimotai,Y.; Hongo,S.; Oshitani,H.; Mizuta,K.; Nishimura,H.
EPI813701	PB2	Japan	2002-Jan-24	EPI_ISL_230238	C/Miyagi/4/2002		Import from public-domain	Matsuzaki,Y.; Sugawara,K.; Furuse,Y.; Shimotai,Y.; Hongo,S.; Oshitani,H.; Mizuta,K.; Nishimura,H.
EPI813666	HE	Japan	2005-May-16	EPI_ISL_230251	C/Miyagi/4/2005		Import from public-domain	Matsuzaki,Y.; Sugawara,K.; Furuse,Y.; Shimotai,Y.; Hongo,S.; Oshitani,H.; Mizuta,K.; Nishimura,H.
EPI816697	MP	Japan	2005-May-16	EPI_ISL_230251	C/Miyagi/4/2005		Import from public-domain	Matsuzaki,Y.; Sugawara,K.; Furuse,Y.; Shimotai,Y.; Hongo,S.; Oshitani,H.; Mizuta,K.; Nishimura,H.
EPI816631	NP	Japan	2005-May-16	EPI_ISL_230251	C/Miyagi/4/2005		Import from public-domain	Matsuzaki,Y.; Sugawara,K.; Furuse,Y.; Shimotai,Y.; Hongo,S.; Oshitani,H.; Mizuta,K.; Nishimura,H.
EPI816766	NS	Japan	2005-May-16	EPI_ISL_230251	C/Miyagi/4/2005		Import from public-domain	Matsuzaki,Y.; Sugawara,K.; Furuse,Y.; Shimotai,Y.; Hongo,S.; Oshitani,H.; Mizuta,K.; Nishimura,H.
EPI816530	P3	Japan	2005-May-16	EPI_ISL_230251	C/Miyagi/4/2005		Import from public-domain	Matsuzaki,Y.; Sugawara,K.; Furuse,Y.; Shimotai,Y.; Hongo,S.; Oshitani,H.; Mizuta,K.; Nishimura,H.
EPI813812	PB1	Japan	2005-May-16	EPI_ISL_230251	C/Miyagi/4/2005		Import from public-domain	Matsuzaki,Y.; Sugawara,K.; Furuse,Y.; Shimotai,Y.; Hongo,S.; Oshitani,H.; Mizuta,K.; Nishimura,H.
EPI813715	PB2	Japan	2005-May-16	EPI_ISL_230251	C/Miyagi/4/2005		Import from public-domain	Matsuzaki,Y.; Sugawara,K.; Furuse,Y.; Shimotai,Y.; Hongo,S.; Oshitani,H.; Mizuta,K.; Nishimura,H.
EPI813673	HE	Japan	2006-Jun-04	EPI_ISL_230258	C/Miyagi/4/2006		Import from public-domain	Matsuzaki,Y.; Sugawara,K.; Furuse,Y.; Shimotai,Y.; Hongo,S.; Oshitani,H.; Mizuta,K.; Nishimura,H.
EPI816705	MP	Japan	2006-Jun-04	EPI_ISL_230258	C/Miyagi/4/2006		Import from public-domain	Matsuzaki,Y.; Sugawara,K.; Furuse,Y.; Shimotai,Y.; Hongo,S.; Oshitani,H.; Mizuta,K.; Nishimura,H.
EPI816639	NP	Japan	2006-Jun-04	EPI_ISL_230258	C/Miyagi/4/2006		Import from public-domain	Matsuzaki,Y.; Sugawara,K.; Furuse,Y.; Shimotai,Y.; Hongo,S.; Oshitani,H.; Mizuta,K.; Nishimura,H.
EPI816774	NS	Japan	2006-Jun-04	EPI_ISL_230258	C/Miyagi/4/2006		Import from public-domain	Matsuzaki,Y.; Sugawara,K.; Furuse,Y.; Shimotai,Y.; Hongo,S.; Oshitani,H.; Mizuta,K.; Nishimura,H.
EPI816538	P3	Japan	2006-Jun-04	EPI_ISL_230258	C/Miyagi/4/2006		Import from public-domain	Matsuzaki,Y.; Sugawara,K.; Furuse,Y.; Shimotai,Y.; Hongo,S.; Oshitani,H.; Mizuta,K.; Nishimura,H.
EPI816435	PB1	Japan	2006-Jun-04	EPI_ISL_230258	C/Miyagi/4/2006		Import from public-domain	Matsuzaki,Y.; Sugawara,K.; Furuse,Y.; Shimotai,Y.; Hongo,S.; Oshitani,H.; Mizuta,K.; Nishimura,H.
EPI813745	PB2	Japan	2006-Jun-04	EPI_ISL_230258	C/Miyagi/4/2006		Import from public-domain	Matsuzaki,Y.; Sugawara,K.; Furuse,Y.; Shimotai,Y.; Hongo,S.; Oshitani,H.; Mizuta,K.; Nishimura,H.
EPI231778	HE	Japan	1993-Jan-01	EPI_ISL_66396	C/Miyagi/4/93		Import from public-domain	Matsuzaki,Y.
EPI231775	MP	Japan	1993-Jan-01	EPI_ISL_66396	C/Miyagi/4/93		Import from public-domain	Matsuzaki,Y.
EPI231774	NS	Japan	1993-Jan-01	EPI_ISL_66396	C/Miyagi/4/93		Import from public-domain	Matsuzaki,Y.
EPI231785	HE	Japan	1996-Jan-01	EPI_ISL_66397	C/Miyagi/4/96		Import from public-domain	Matsuzaki,Y.
EPI231782	MP	Japan	1996-Jan-01	EPI_ISL_66397	C/Miyagi/4/96		Import from public-domain	Matsuzaki,Y.
EPI231781	NS	Japan	1996-Jan-01	EPI_ISL_66397	C/Miyagi/4/96		Import from public-domain	Matsuzaki,Y.
EPI231788	HE	Japan	1997-Jan-01	EPI_ISL_66398	C/Miyagi/4/97		Import from public-domain	Matsuzaki,Y.
EPI231795	HE	Japan	1998-Jan-01	EPI_ISL_66399	C/Miyagi/4/98		Import from public-domain	Matsuzaki,Y.
EPI231790	MP	Japan	1998-Jan-01	EPI_ISL_66399	C/Miyagi/4/98		Import from public-domain	Matsuzaki,Y.
EPI231789	NS	Japan	1998-Jan-01	EPI_ISL_66399	C/Miyagi/4/98		Import from public-domain	Matsuzaki,Y.
EPI228323	HE	Japan	2004-May-27	EPI_ISL_65159	C/Miyagi/42/2004		Import from public-domain	Matsuzaki,Y.; Sugawara,K.; Furuse,Y.; Shimotai,Y.; Hongo,S.; Oshitani,H.; Mizuta,K.; Nishimura,H.
EPI816691	MP	Japan	2004-May-27	EPI_ISL_65159	C/Miyagi/42/2004		Import from public-domain	Matsuzaki,Y.; Sugawara,K.; Furuse,Y.; Shimotai,Y.; Hongo,S.; Oshitani,H.; Mizuta,K.; Nishimura,H.
EPI816626	NP	Japan	2004-May-27	EPI_ISL_65159	C/Miyagi/42/2004		Import from public-domain	Matsuzaki,Y.; Sugawara,K.; Furuse,Y.; Shimotai,Y.; Hongo,S.; Oshitani,H.; Mizuta,K.; Nishimura,H.
EPI816760	NS	Japan	2004-May-27	EPI_ISL_65159	C/Miyagi/42/2004		Import from public-domain	Matsuzaki,Y.; Sugawara,K.; Furuse,Y.; Shimotai,Y.; Hongo,S.; Oshitani,H.; Mizuta,K.; Nishimura,H.
EPI816524	P3	Japan	2004-May-27	EPI_ISL_65159	C/Miyagi/42/2004		Import from public-domain	Matsuzaki,Y.; Sugawara,K.; Furuse,Y.; Shimotai,Y.; Hongo,S.; Oshitani,H.; Mizuta,K.; Nishimura,H.
EPI816430	PB1	Japan	2004-May-27	EPI_ISL_65159	C/Miyagi/42/2004		Import from public-domain	Matsuzaki,Y.; Sugawara,K.; Furuse,Y.; Shimotai,Y.; Hongo,S.; Oshitani,H.; Mizuta,K.; Nishimura,H.
EPI813726	PB2	Japan	2004-May-27	EPI_ISL_65159	C/Miyagi/42/2004		Import from public-domain	Matsuzaki,Y.; Sugawara,K.; Furuse,Y.; Shimotai,Y.; Hongo,S.; Oshitani,H.; Mizuta,K.; Nishimura,H.
EPI813661	HE	Japan	2004-Jun-18	EPI_ISL_230246	C/Miyagi/47/2004		Import from public-domain	Matsuzaki,Y.; Sugawara,K.; Furuse,Y.; Shimotai,Y.; Hongo,S.; Oshitani,H.; Mizuta,K.; Nishimura,H.
EPI816692	MP	Japan	2004-Jun-18	EPI_ISL_230246	C/Miyagi/47/2004		Import from public-domain	Matsuzaki,Y.; Sugawara,K.; Furuse,Y.; Shimotai,Y.; Hongo,S.; Oshitani,H.; Mizuta,K.; Nishimura,H.
EPI816627	NP	Japan	2004-Jun-18	EPI_ISL_230246	C/Miyagi/47/2004		Import from public-domain	Matsuzaki,Y.; Sugawara,K.; Furuse,Y.; Shimotai,Y.; Hongo,S.; Oshitani,H.; Mizuta,K.; Nishimura,H.
EPI816761	NS	Japan	2004-Jun-18	EPI_ISL_230246	C/Miyagi/47/2004		Import from public-domain	Matsuzaki,Y.; Sugawara,K.; Furuse,Y.; Shimotai,Y.; Hongo,S.; Oshitani,H.; Mizuta,K.; Nishimura,H.
EPI816525	P3	Japan	2004-Jun-18	EPI_ISL_230246	C/Miyagi/47/2004		Import from public-domain	Matsuzaki,Y.; Sugawara,K.; Furuse,Y.; Shimotai,Y.; Hongo,S.; Oshitani,H.; Mizuta,K.; Nishimura,H.
EPI813807	PB1	Japan	2004-Jun-18	EPI_ISL_230246	C/Miyagi/47/2004		Import from public-domain	Matsuzaki,Y.; Sugawara,K.; Furuse,Y.; Shimotai,Y.; Hongo,S.; Oshitani,H.; Mizuta,K.; Nishimura,H.
EPI813710	PB2	Japan	2004-Jun-18	EPI_ISL_230246	C/Miyagi/47/2004		Import from public-domain	Matsuzaki,Y.; Sugawara,K.; Furuse,Y.; Shimotai,Y.; Hongo,S.; Oshitani,H.; Mizuta,K.; Nishimura,H.
EPI231798	HE	Japan	2000-Jan-01	EPI_ISL_66400	C/Miyagi/5/2000		Import from public-domain	Matsuzaki,Y.
EPI231797	MP	Japan	2000-Jan-01	EPI_ISL_66400	C/Miyagi/5/2000		Import from public-domain	Matsuzaki,Y.
EPI231796	NS	Japan	2000-Jan-01	EPI_ISL_66400	C/Miyagi/5/2000		Import from public-domain	Matsuzaki,Y.
EPI813689	HE	Japan	2014-May-17	EPI_ISL_230274	C/Miyagi/5/2014		Import from public-domain	Matsuzaki,Y.; Sugawara,K.; Furuse,Y.; Shimotai,Y.; Hongo,S.; Oshitani,H.; Mizuta,K.; Nishimura,H.
EPI816732	MP	Japan	2014-May-17	EPI_ISL_230274	C/Miyagi/5/2014		Import from public-domain	Matsuzaki,Y.; Sugawara,K.; Furuse,Y.; Shimotai,Y.; Hongo,S.; Oshitani,H.; Mizuta,K.; Nishimura,H.
EPI816664	NP	Japan	2014-May-17	EPI_ISL_230274	C/Miyagi/5/2014		Import from public-domain	Matsuzaki,Y.; Sugawara,K.; Furuse,Y.; Shimotai,Y.; Hongo,S.; Oshitani,H.; Mizuta,K.; Nishimura,H.
EPI816801	NS	Japan	2014-May-17	EPI_ISL_230274	C/Miyagi/5/2014		Import from public-domain	Matsuzaki,Y.; Sugawara,K.; Furuse,Y.; Shimotai,Y.; Hongo,S.; Oshitani,H.; Mizuta,K.; Nishimura,H.
EPI816565	P3	Japan	2014-May-17	EPI_ISL_230274	C/Miyagi/5/2014		Import from public-domain	Matsuzaki,Y.; Sugawara,K.; Furuse,Y.; Shimotai,Y.; Hongo,S.; Oshitani,H.; Mizuta,K.; Nishimura,H.
EPI816454	PB1	Japan	2014-May-17	EPI_ISL_230274	C/Miyagi/5/2014		Import from public-domain	Matsuzaki,Y.; Sugawara,K.; Furuse,Y.; Shimotai,Y.; Hongo,S.; Oshitani,H.; Mizuta,K.; Nishimura,H.
EPI813764	PB2	Japan	2014-May-17	EPI_ISL_230274	C/Miyagi/5/2014		Import from public-domain	Matsuzaki,Y.; Sugawara,K.; Furuse,Y.; Shimotai,Y.; Hongo,S.; Oshitani,H.; Mizuta,K.; Nishimura,H.
EPI231808	HE	Japan	1991-Feb-25	EPI_ISL_66356	C/Miyagi/5/91		Import from public-domain	Matsuzaki,Y.; Sugawara,K.; Furuse,Y.; Shimotai,Y.; Hongo,S.; Oshitani,H.; Mizuta,K.; Nishimura,H.
EPI231804	MP	Japan	1991-Feb-25	EPI_ISL_66356	C/Miyagi/5/91		Import from public-domain	Matsuzaki,Y.; Sugawara,K.; Furuse,Y.; Shimotai,Y.; Hongo,S.; Oshitani,H.; Mizuta,K.; Nishimura,H.
EPI816594	NP	Japan	1991-Feb-25	EPI_ISL_66356	C/Miyagi/5/91		Import from public-domain	Matsuzaki,Y.; Sugawara,K.; Furuse,Y.; Shimotai,Y.; Hongo,S.; Oshitani,H.; Mizuta,K.; Nishimura,H.
EPI231567	NS	Japan	1991-Feb-25	EPI_ISL_66356	C/Miyagi/5/91		Import from public-domain	Matsuzaki,Y.; Sugawara,K.; Furuse,Y.; Shimotai,Y.; Hongo,S.; Oshitani,H.; Mizuta,K.; Nishimura,H.
EPI816495	P3	Japan	1991-Feb-25	EPI_ISL_66356	C/Miyagi/5/91		Import from public-domain	Matsuzaki,Y.; Sugawara,K.; Furuse,Y.; Shimotai,Y.; Hongo,S.; Oshitani,H.; Mizuta,K.; Nishimura,H.
EPI816413	PB1	Japan	1991-Feb-25	EPI_ISL_66356	C/Miyagi/5/91		Import from public-domain	Matsuzaki,Y.; Sugawara,K.; Furuse,Y.; Shimotai,Y.; Hongo,S.; Oshitani,H.; Mizuta,K.; Nishimura,H.
EPI813727	PB2	Japan	1991-Feb-25	EPI_ISL_66356	C/Miyagi/5/91		Import from public-domain	Matsuzaki,Y.; Sugawara,K.; Furuse,Y.; Shimotai,Y.; Hongo,S.; Oshitani,H.; Mizuta,K.; Nishimura,H.
EPI231813	HE	Japan	1993-Jan-01	EPI_ISL_66401	C/Miyagi/5/93		Import from public-domain	Matsuzaki,Y.
EPI231811	MP	Japan	1993-Jan-01	EPI_ISL_66401	C/Miyagi/5/93		Import from public-domain	Matsuzaki,Y.
EPI231812	NS	Japan	1993-Jan-01	EPI_ISL_66401	C/Miyagi/5/93		Import from public-domain	Matsuzaki,Y.
EPI813690	HE	Japan	2014-Jun-02	EPI_ISL_230275	C/Miyagi/6/2014		Import from public-domain	Matsuzaki,Y.; Sugawara,K.; Furuse,Y.; Shimotai,Y.; Hongo,S.; Oshitani,H.; Mizuta,K.; Nishimura,H.
EPI816733	MP	Japan	2014-Jun-02	EPI_ISL_230275	C/Miyagi/6/2014		Import from public-domain	Matsuzaki,Y.; Sugawara,K.; Furuse,Y.; Shimotai,Y.; Hongo,S.; Oshitani,H.; Mizuta,K.; Nishimura,H.
EPI816665	NP	Japan	2014-Jun-02	EPI_ISL_230275	C/Miyagi/6/2014		Import from public-domain	Matsuzaki,Y.; Sugawara,K.; Furuse,Y.; Shimotai,Y.; Hongo,S.; Oshitani,H.; Mizuta,K.; Nishimura,H.
EPI816802	NS	Japan	2014-Jun-02	EPI_ISL_230275	C/Miyagi/6/2014		Import from public-domain	Matsuzaki,Y.; Sugawara,K.; Furuse,Y.; Shimotai,Y.; Hongo,S.; Oshitani,H.; Mizuta,K.; Nishimura,H.
EPI816566	P3	Japan	2014-Jun-02	EPI_ISL_230275	C/Miyagi/6/2014		Import from public-domain	Matsuzaki,Y.; Sugawara,K.; Furuse,Y.; Shimotai,Y.; Hongo,S.; Oshitani,H.; Mizuta,K.; Nishimura,H.
EPI816455	PB1	Japan	2014-Jun-02	EPI_ISL_230275	C/Miyagi/6/2014		Import from public-domain	Matsuzaki,Y.; Sugawara,K.; Furuse,Y.; Shimotai,Y.; Hongo,S.; Oshitani,H.; Mizuta,K.; Nishimura,H.
EPI813765	PB2	Japan	2014-Jun-02	EPI_ISL_230275	C/Miyagi/6/2014		Import from public-domain	Matsuzaki,Y.; Sugawara,K.; Furuse,Y.; Shimotai,Y.; Hongo,S.; Oshitani,H.; Mizuta,K.; Nishimura,H.
EPI231822	HE	Japan	1993-Jan-01	EPI_ISL_66403	C/Miyagi/6/93		Import from public-domain	Matsuzaki,Y.
EPI231817	MP	Japan	1993-Jan-01	EPI_ISL_66403	C/Miyagi/6/93		Import from public-domain	Matsuzaki,Y.
EPI231816	NS	Japan	1993-Jan-01	EPI_ISL_66403	C/Miyagi/6/93		Import from public-domain	Matsuzaki,Y.
EPI231829	HE	Japan	1996-Jan-01	EPI_ISL_66404	C/Miyagi/6/96		Import from public-domain	Matsuzaki,Y.
EPI231827	MP	Japan	1996-Jan-01	EPI_ISL_66404	C/Miyagi/6/96		Import from public-domain	Matsuzaki,Y.
EPI231823	NS	Japan	1996-Jan-01	EPI_ISL_66404	C/Miyagi/6/96		Import from public-domain	Matsuzaki,Y.
EPI231830	HE	Japan	1991-Jan-01	EPI_ISL_66357	C/Miyagi/7/91		Import from public-domain	Matsuzaki,Y.
EPI231834	MP	Japan	1991-Jan-01	EPI_ISL_66357	C/Miyagi/7/91		Import from public-domain	Matsuzaki,Y.
EPI231568	NS	Japan	1991-Jan-01	EPI_ISL_66357	C/Miyagi/7/91		Import from public-domain	Matsuzaki,Y.
EPI231836	HE	Japan	1993-May-19	EPI_ISL_66405	C/Miyagi/7/93		Import from public-domain	Matsuzaki,Y.; Sugawara,K.; Furuse,Y.; Shimotai,Y.; Hongo,S.; Oshitani,H.; Mizuta,K.; Nishimura,H.
EPI231841	MP	Japan	1993-May-19	EPI_ISL_66405	C/Miyagi/7/93		Import from public-domain	Matsuzaki,Y.; Sugawara,K.; Furuse,Y.; Shimotai,Y.; Hongo,S.; Oshitani,H.; Mizuta,K.; Nishimura,H.
EPI816606	NP	Japan	1993-May-19	EPI_ISL_66405	C/Miyagi/7/93		Import from public-domain	Matsuzaki,Y.; Sugawara,K.; Furuse,Y.; Shimotai,Y.; Hongo,S.; Oshitani,H.; Mizuta,K.; Nishimura,H.
EPI231842	NS	Japan	1993-May-19	EPI_ISL_66405	C/Miyagi/7/93		Import from public-domain	Matsuzaki,Y.; Sugawara,K.; Furuse,Y.; Shimotai,Y.; Hongo,S.; Oshitani,H.; Mizuta,K.; Nishimura,H.
EPI816501	P3	Japan	1993-May-19	EPI_ISL_66405	C/Miyagi/7/93		Import from public-domain	Matsuzaki,Y.; Sugawara,K.; Furuse,Y.; Shimotai,Y.; Hongo,S.; Oshitani,H.; Mizuta,K.; Nishimura,H.
EPI816418	PB1	Japan	1993-May-19	EPI_ISL_66405	C/Miyagi/7/93		Import from public-domain	Matsuzaki,Y.; Sugawara,K.; Furuse,Y.; Shimotai,Y.; Hongo,S.; Oshitani,H.; Mizuta,K.; Nishimura,H.
EPI813728	PB2	Japan	1993-May-19	EPI_ISL_66405	C/Miyagi/7/93		Import from public-domain	Matsuzaki,Y.; Sugawara,K.; Furuse,Y.; Shimotai,Y.; Hongo,S.; Oshitani,H.; Mizuta,K.; Nishimura,H.
EPI231849	HE	Japan	1996-Jan-01	EPI_ISL_66406	C/Miyagi/7/96		Import from public-domain	Matsuzaki,Y.
EPI231843	MP	Japan	1996-Jan-01	EPI_ISL_66406	C/Miyagi/7/96		Import from public-domain	Matsuzaki,Y.
EPI231846	NS	Japan	1996-Jan-01	EPI_ISL_66406	C/Miyagi/7/96		Import from public-domain	Matsuzaki,Y.
EPI232107	HE	Japan	1977-Jan-01	EPI_ISL_66329	C/Miyagi/77		Import from public-domain	Matsuzaki,Y.; Sugawara,K.; Furuse,Y.; Shimotai,Y.; Hongo,S.; Oshitani,H.; Mizuta,K.; Nishimura,H.
EPI232108	MP	Japan	1977-Jan-01	EPI_ISL_66329	C/Miyagi/77		Import from public-domain	Matsuzaki,Y.; Sugawara,K.; Furuse,Y.; Shimotai,Y.; Hongo,S.; Oshitani,H.; Mizuta,K.; Nishimura,H.
EPI816577	NP	Japan	1977-Jan-01	EPI_ISL_66329	C/Miyagi/77		Import from public-domain	Matsuzaki,Y.; Sugawara,K.; Furuse,Y.; Shimotai,Y.; Hongo,S.; Oshitani,H.; Mizuta,K.; Nishimura,H.
EPI231530	NS	Japan	1977-Jan-01	EPI_ISL_66329	C/Miyagi/77		Import from public-domain	Matsuzaki,Y.; Sugawara,K.; Furuse,Y.; Shimotai,Y.; Hongo,S.; Oshitani,H.; Mizuta,K.; Nishimura,H.
EPI816468	P3	Japan	1977-Jan-01	EPI_ISL_66329	C/Miyagi/77		Import from public-domain	Matsuzaki,Y.; Sugawara,K.; Furuse,Y.; Shimotai,Y.; Hongo,S.; Oshitani,H.; Mizuta,K.; Nishimura,H.
EPI813781	PB1	Japan	1977-Jan-01	EPI_ISL_66329	C/Miyagi/77		Import from public-domain	Matsuzaki,Y.; Sugawara,K.; Furuse,Y.; Shimotai,Y.; Hongo,S.; Oshitani,H.; Mizuta,K.; Nishimura,H.
EPI814971	PB2	Japan	1977-Jan-01	EPI_ISL_66329	C/Miyagi/77		Import from public-domain	Matsuzaki,Y.; Sugawara,K.; Furuse,Y.; Shimotai,Y.; Hongo,S.; Oshitani,H.; Mizuta,K.; Nishimura,H.
EPI231854	HE	Japan	1996-May-23	EPI_ISL_66407	C/Miyagi/8/96		Import from public-domain	Matsuzaki,Y.; Sugawara,K.; Furuse,Y.; Shimotai,Y.; Hongo,S.; Oshitani,H.; Mizuta,K.; Nishimura,H.
EPI231860	MP	Japan	1996-May-23	EPI_ISL_66407	C/Miyagi/8/96		Import from public-domain	Matsuzaki,Y.; Sugawara,K.; Furuse,Y.; Shimotai,Y.; Hongo,S.; Oshitani,H.; Mizuta,K.; Nishimura,H.
EPI816607	NP	Japan	1996-May-23	EPI_ISL_66407	C/Miyagi/8/96		Import from public-domain	Matsuzaki,Y.; Sugawara,K.; Furuse,Y.; Shimotai,Y.; Hongo,S.; Oshitani,H.; Mizuta,K.; Nishimura,H.
EPI231857	NS	Japan	1996-May-23	EPI_ISL_66407	C/Miyagi/8/96		Import from public-domain	Matsuzaki,Y.; Sugawara,K.; Furuse,Y.; Shimotai,Y.; Hongo,S.; Oshitani,H.; Mizuta,K.; Nishimura,H.
EPI816502	P3	Japan	1996-May-23	EPI_ISL_66407	C/Miyagi/8/96		Import from public-domain	Matsuzaki,Y.; Sugawara,K.; Furuse,Y.; Shimotai,Y.; Hongo,S.; Oshitani,H.; Mizuta,K.; Nishimura,H.
EPI231859	PB1	Japan	1996-May-23	EPI_ISL_66407	C/Miyagi/8/96		Import from public-domain	Matsuzaki,Y.; Sugawara,K.; Furuse,Y.; Shimotai,Y.; Hongo,S.; Oshitani,H.; Mizuta,K.; Nishimura,H.
EPI816419	PB1	Japan	1996-May-23	EPI_ISL_66407	C/Miyagi/8/96		Import from public-domain	Matsuzaki,Y.; Sugawara,K.; Furuse,Y.; Shimotai,Y.; Hongo,S.; Oshitani,H.; Mizuta,K.; Nishimura,H.
EPI813730	PB2	Japan	1996-May-23	EPI_ISL_66407	C/Miyagi/8/96		Import from public-domain	Matsuzaki,Y.; Sugawara,K.; Furuse,Y.; Shimotai,Y.; Hongo,S.; Oshitani,H.; Mizuta,K.; Nishimura,H.
EPI232109	HE	Japan	1991-Jan-01	EPI_ISL_66358	C/Miyagi/9/91		Import from public-domain	Matsuzaki,Y.
EPI232115	MP	Japan	1991-Jan-01	EPI_ISL_66358	C/Miyagi/9/91		Import from public-domain	Matsuzaki,Y.
EPI231569	NS	Japan	1991-Jan-01	EPI_ISL_66358	C/Miyagi/9/91		Import from public-domain	Matsuzaki,Y.
EPI231862	HE	Japan	1996-Jun-03	EPI_ISL_66408	C/Miyagi/9/96		Import from public-domain	Matsuzaki,Y.; Sugawara,K.; Furuse,Y.; Shimotai,Y.; Hongo,S.; Oshitani,H.; Mizuta,K.; Nishimura,H.
EPI231861	MP	Japan	1996-Jun-03	EPI_ISL_66408	C/Miyagi/9/96		Import from public-domain	Matsuzaki,Y.; Sugawara,K.; Furuse,Y.; Shimotai,Y.; Hongo,S.; Oshitani,H.; Mizuta,K.; Nishimura,H.
EPI816608	NP	Japan	1996-Jun-03	EPI_ISL_66408	C/Miyagi/9/96		Import from public-domain	Matsuzaki,Y.; Sugawara,K.; Furuse,Y.; Shimotai,Y.; Hongo,S.; Oshitani,H.; Mizuta,K.; Nishimura,H.
EPI231866	NS	Japan	1996-Jun-03	EPI_ISL_66408	C/Miyagi/9/96		Import from public-domain	Matsuzaki,Y.; Sugawara,K.; Furuse,Y.; Shimotai,Y.; Hongo,S.; Oshitani,H.; Mizuta,K.; Nishimura,H.
EPI816503	P3	Japan	1996-Jun-03	EPI_ISL_66408	C/Miyagi/9/96		Import from public-domain	Matsuzaki,Y.; Sugawara,K.; Furuse,Y.; Shimotai,Y.; Hongo,S.; Oshitani,H.; Mizuta,K.; Nishimura,H.

Segment ID	Segment	Country	Collection date	Isolate-ID	Isolate name	Originating Lab	Submitting Lab	Authors
EPI816420	PB1	Japan	1996-Jun-03	EPI_ISL_66408	C/Miyagi/9/96		Import from public-domain	Matsuzaki,Y.; Sugawara,K.; Furuse,Y.; Shimotai,Y.; Hongo,S.; Oshitani,H.; Mizuta,K.; Nishimura,H.
EPI813732	PB2	Japan	1996-Jun-03	EPI_ISL_66408	C/Miyagi/9/96		Import from public-domain	Matsuzaki,Y.; Sugawara,K.; Furuse,Y.; Shimotai,Y.; Hongo,S.; Oshitani,H.; Mizuta,K.; Nishimura,H.
EPI232128	MP	Japan	1985-Jan-01	EPI_ISL_66352	C/Nara/1/85		Import from public-domain	Matsuzaki,Y.
EPI231561	NS	Japan	1985-Jan-01	EPI_ISL_66352	C/Nara/1/85		Import from public-domain	Matsuzaki,Y.
EPI231557	NS	Japan	1986-Jan-01	EPI_ISL_66348	C/Nara/1/86		Import from public-domain	Matsuzaki,Y.
EPI232141	HE	Japan	1985-Jan-01	EPI_ISL_66353	C/Nara/2/85		Import from public-domain	Matsuzaki,Y.
EPI232129	MP	Japan	1985-Jan-01	EPI_ISL_66353	C/Nara/2/85		Import from public-domain	Matsuzaki,Y.
EPI231562	NS	Japan	1985-Jan-01	EPI_ISL_66353	C/Nara/2/85		Import from public-domain	Matsuzaki,Y.
EPI231545	HE	Japan	1982-Jan-01	EPI_ISL_66342	C/Nara/82		Import from public-domain	Adachi,K.; Kitame,F.; Sugawara,K.; Nishimura,H.; Nakamura,K.
EPI232146	MP	Japan	1982-Jan-01	EPI_ISL_66434	C/Nara/82		Import from public-domain	Matsuzaki,Y.; Sugawara,K.; Furuse,Y.; Shimotai,Y.; Hongo,S.; Oshitani,H.; Mizuta,K.; Nishimura,H.
EPI816589	NP	Japan	1982-Jan-01	EPI_ISL_66434	C/Nara/82		Import from public-domain	Matsuzaki,Y.; Sugawara,K.; Furuse,Y.; Shimotai,Y.; Hongo,S.; Oshitani,H.; Mizuta,K.; Nishimura,H.
EPI232023	NS	Japan	1982-Jan-01	EPI_ISL_66434	C/Nara/82		Import from public-domain	Matsuzaki,Y.; Sugawara,K.; Furuse,Y.; Shimotai,Y.; Hongo,S.; Oshitani,H.; Mizuta,K.; Nishimura,H.
EPI816490	P3	Japan	1982-Jan-01	EPI_ISL_66434	C/Nara/82		Import from public-domain	Matsuzaki,Y.; Sugawara,K.; Furuse,Y.; Shimotai,Y.; Hongo,S.; Oshitani,H.; Mizuta,K.; Nishimura,H.
EPI815446	PB1	Japan	1982-Jan-01	EPI_ISL_66434	C/Nara/82		Import from public-domain	Matsuzaki,Y.; Sugawara,K.; Furuse,Y.; Shimotai,Y.; Hongo,S.; Oshitani,H.; Mizuta,K.; Nishimura,H.
EPI815369	PB2	Japan	1982-Jan-01	EPI_ISL_66434	C/Nara/82		Import from public-domain	Matsuzaki,Y.; Sugawara,K.; Furuse,Y.; Shimotai,Y.; Hongo,S.; Oshitani,H.; Mizuta,K.; Nishimura,H.
EPI231528	HE	United States	1976-Jan-01	EPI_ISL_66327	C/New Jersey/1/76		Import from public-domain	Matsuzaki,Y.; Sugawara,K.; Furuse,Y.; Shimotai,Y.; Hongo,S.; Oshitani,H.; Mizuta,K.; Nishimura,H.
EPI816466	P3	United States	1976-Jan-01	EPI_ISL_66327	C/New Jersey/1/76		Import from public-domain	Matsuzaki,Y.; Sugawara,K.; Furuse,Y.; Shimotai,Y.; Hongo,S.; Oshitani,H.; Mizuta,K.; Nishimura,H.
EPI813786	PB1	United States	1976-Jan-01	EPI_ISL_66327	C/New Jersey/1/76		Import from public-domain	Matsuzaki,Y.; Sugawara,K.; Furuse,Y.; Shimotai,Y.; Hongo,S.; Oshitani,H.; Mizuta,K.; Nishimura,H.
EPI814937	PB2	United States	1976-Jan-01	EPI_ISL_66327	C/New Jersey/1/76		Import from public-domain	Matsuzaki,Y.; Sugawara,K.; Furuse,Y.; Shimotai,Y.; Hongo,S.; Oshitani,H.; Mizuta,K.; Nishimura,H.
EPI231872	MP	United States	1976-Jan-01	EPI_ISL_66409	C/NewJersey/76		Import from public-domain	Matsuzaki,Y.
EPI369794	NP	United States	1976-Jan-01	EPI_ISL_118429	C/NewJersey/76		Import from public-domain	Matsuzaki,Y.; Ikeda,T.; Abiko,C.; Aoki,Y.; Mizuta,K.; Shimotai,Y.; Sugawara,K.; Hongo,S.
EPI231873	NS	United States	1976-Jan-01	EPI_ISL_66409	C/NewJersey/76		Import from public-domain	Matsuzaki,Y.
EPI228324	HE	Japan	2004-Jan-01	EPI_ISL_65160	C/Niigata/1/2004		Import from public-domain	Matsuzaki,Y.
EPI1183986	HE	Germany	2012-Nov-16	EPI_ISL_300534	C/Nordrhein-Westfalen/13-00344/2012		Robert Koch Institute Nationales Referenzzentrum für Influenza	Biere, B.; Fritsch, A.; Schweiger, B.
EPI1183992	HE	Germany	2013-Feb-27	EPI_ISL_300540	C/Nordrhein-Westfalen/13-04022/2013		Robert Koch Institute Nationales Referenzzentrum für Influenza	Biere, B.; Fritsch, A.; Schweiger, B.
EPI228328	HE	Japan	2004-Jan-01	EPI_ISL_65164	C/Osaka/2/2004		Import from public-domain	Matsuzaki,Y.
EPI581552	HE	Philippines	2013-Jan-01	EPI_ISL_176788	C/Palawan/1/2013		Import from public-domain	Odagiri,T.; Matsuzaki,Y.; Okamoto,M.; Hongo,S.; Oshitani,H.
EPI621717	MP	Philippines	2013-Jan-01	EPI_ISL_176788	C/Palawan/1/2013		Import from public-domain	Odagiri,T.; Matsuzaki,Y.; Okamoto,M.; Hongo,S.; Oshitani,H.
EPI621700	NS	Philippines	2013-Jan-01	EPI_ISL_176788	C/Palawan/1/2013		Import from public-domain	Odagiri,T.; Matsuzaki,Y.; Okamoto,M.; Hongo,S.; Oshitani,H.
EPI231522	HE	France	1967-Jan-01	EPI_ISL_66322	C/Paris/1/67		Import from public-domain	Matsuzaki,Y.; Sugawara,K.; Furuse,Y.; Shimotai,Y.; Hongo,S.; Oshitani,H.; Mizuta,K.; Nishimura,H.
EPI816670	MP	France	1967-Jan-01	EPI_ISL_66322	C/Paris/1/67		Import from public-domain	Matsuzaki,Y.; Sugawara,K.; Furuse,Y.; Shimotai,Y.; Hongo,S.; Oshitani,H.; Mizuta,K.; Nishimura,H.
EPI816738	NS	France	1967-Jan-01	EPI_ISL_66322	C/Paris/1/67		Import from public-domain	Matsuzaki,Y.; Sugawara,K.; Furuse,Y.; Shimotai,Y.; Hongo,S.; Oshitani,H.; Mizuta,K.; Nishimura,H.
EPI816462	P3	France	1967-Jan-01	EPI_ISL_66322	C/Paris/1/67		Import from public-domain	Matsuzaki,Y.; Sugawara,K.; Furuse,Y.; Shimotai,Y.; Hongo,S.; Oshitani,H.; Mizuta,K.; Nishimura,H.
EPI813782	PB1	France	1967-Jan-01	EPI_ISL_66322	C/Paris/1/67		Import from public-domain	Matsuzaki,Y.; Sugawara,K.; Furuse,Y.; Shimotai,Y.; Hongo,S.; Oshitani,H.; Mizuta,K.; Nishimura,H.
EPI814787	PB2	France	1967-Jan-01	EPI_ISL_66322	C/Paris/1/67		Import from public-domain	Matsuzaki,Y.; Sugawara,K.; Furuse,Y.; Shimotai,Y.; Hongo,S.; Oshitani,H.; Mizuta,K.; Nishimura,H.
EPI711146	HE	Australia	2008-Oct-29	EPI_ISL_212084	C/Perth/1/2008	Pathwest QE II Medical Centre	WHO Collaborating Centre for Reference and Research on Influenza	
EPI711132	HE	Australia	2012-Sep-23	EPI_ISL_212070	C/Perth/1/2012	Pathwest QE II Medical Centre	WHO Collaborating Centre for Reference and Research on Influenza	
EPI675716	HE	Australia	2014-Jul-18	EPI_ISL_202517	C/Perth/1/2014	Pathwest QE II Medical Centre	WHO Collaborating Centre for Reference and Research on Influenza	Deng,Y-M.; Spirason,N.; Jelley,L.; Komadina,N.
EPI711140	HE	Australia	2012-Jul-18	EPI_ISL_212078	C/Perth/10/2012	Pathwest QE II Medical Centre	WHO Collaborating Centre for Reference and Research on Influenza	
EPI675712	HE	Australia	2014-Aug-17	EPI_ISL_202513	C/Perth/10/2014	Pathwest QE II Medical Centre	WHO Collaborating Centre for Reference and Research on Influenza	Deng,Y-M.; Spirason,N.; Jelley,L.; Komadina,N.
EPI711141	HE	Australia	2012-Jul-18	EPI_ISL_212079	C/Perth/11/2012	Pathwest QE II Medical Centre	WHO Collaborating Centre for Reference and Research on Influenza	
EPI711142	HE	Australia	2012-Jul-14	EPI_ISL_212080	C/Perth/12/2012	Pathwest QE II Medical Centre	WHO Collaborating Centre for Reference and Research on Influenza	
EPI675713	HE	Australia	2014-Aug-18	EPI_ISL_202514	C/Perth/12/2014	Pathwest QE II Medical Centre	WHO Collaborating Centre for Reference and Research on Influenza	Deng,Y-M.; Spirason,N.; Jelley,L.; Komadina,N.
EPI711143	HE	Australia	2012-Jun-01	EPI_ISL_212081	C/Perth/13/2012	Pathwest QE II Medical Centre	WHO Collaborating Centre for Reference and Research on Influenza	
EPI675722	HE	Australia	2014-Aug-13	EPI_ISL_202523	C/Perth/15/2014	Pathwest QE II Medical Centre	WHO Collaborating Centre for Reference and Research on Influenza	Deng,Y-M.; Spirason,N.; Jelley,L.; Komadina,N.
EPI675720	HE	Australia	2014-Aug-14	EPI_ISL_202521	C/Perth/17/2014	Pathwest QE II Medical Centre	WHO Collaborating Centre for Reference and Research on Influenza	Deng,Y-M.; Spirason,N.; Jelley,L.; Komadina,N.
EPI675721	HE	Australia	2014-Aug-23	EPI_ISL_202522	C/Perth/19/2014	Pathwest QE II Medical Centre	WHO Collaborating Centre for Reference and Research on Influenza	Deng,Y-M.; Spirason,N.; Jelley,L.; Komadina,N.
EPI711145	HE	Australia	2008-Oct-20	EPI_ISL_212083	C/Perth/2/2008	Pathwest QE II Medical Centre	WHO Collaborating Centre for Reference and Research on Influenza	
EPI711133	HE	Australia	2012-Sep-02	EPI_ISL_212071	C/Perth/2/2012	Pathwest QE II Medical Centre	WHO Collaborating Centre for Reference and Research on Influenza	
EPI675717	HE	Australia	2014-Jul-20	EPI_ISL_202518	C/Perth/2/2014	Pathwest QE II Medical Centre	WHO Collaborating Centre for Reference and Research on Influenza	Deng,Y-M.; Spirason,N.; Jelley,L.; Komadina,N.
EPI675714	HE	Australia	2014-Aug-26	EPI_ISL_202515	C/Perth/23/2014	Pathwest QE II Medical Centre	WHO Collaborating Centre for Reference and Research on Influenza	Deng,Y-M.; Spirason,N.; Jelley,L.; Komadina,N.
EPI675715	HE	Australia	2014-Sep-13	EPI_ISL_202516	C/Perth/26/2014	Pathwest QE II Medical Centre	WHO Collaborating Centre for Reference and Research on Influenza	Deng,Y-M.; Spirason,N.; Jelley,L.; Komadina,N.
EPI711147	HE	Australia	2008-Oct-03	EPI_ISL_212085	C/Perth/3/2008	Pathwest QE II Medical Centre	WHO Collaborating Centre for Reference and Research on Influenza	
EPI711134	HE	Australia	2012-Sep-01	EPI_ISL_212072	C/Perth/3/2012	Pathwest QE II Medical Centre	WHO Collaborating Centre for Reference and Research on Influenza	
EPI675710	HE	Australia	2014-Oct-03	EPI_ISL_202511	C/Perth/31/2014	Pathwest QE II Medical Centre	WHO Collaborating Centre for Reference and Research on Influenza	Deng,Y-M.; Spirason,N.; Jelley,L.; Komadina,N.
EPI711135	HE	Australia	2012-Aug-19	EPI_ISL_212073	C/Perth/5/2012	Pathwest QE II Medical Centre	WHO Collaborating Centre for Reference and Research on Influenza	
EPI675718	HE	Australia	2014-Aug-06	EPI_ISL_202519	C/Perth/5/2014	Pathwest QE II Medical Centre	WHO Collaborating Centre for Reference and Research on Influenza	Deng,Y-M.; Spirason,N.; Jelley,L.; Komadina,N.
EPI711136	HE	Australia	2012-Aug-19	EPI_ISL_212074	C/Perth/6/2012	Pathwest QE II Medical Centre	WHO Collaborating Centre for Reference and Research on Influenza	
EPI711137	HE	Australia	2012-Aug-14	EPI_ISL_212075	C/Perth/7/2012	Pathwest QE II Medical Centre	WHO Collaborating Centre for Reference and Research on Influenza	
EPI711138	HE	Australia	2012-Aug-10	EPI_ISL_212076	C/Perth/8/2012	Pathwest QE II Medical Centre	WHO Collaborating Centre for Reference and Research on Influenza	
EPI675719	HE	Australia	2014-Jul-31	EPI_ISL_202520	C/Perth/8/2014	Pathwest QE II Medical Centre	WHO Collaborating Centre for Reference and Research on Influenza	Deng,Y-M.; Spirason,N.; Jelley,L.; Komadina,N.
EPI711144	HE	Australia	2010-Jul-01	EPI_ISL_212082	C/Perth/9/2010	Pathwest QE II Medical Centre	WHO Collaborating Centre for Reference and Research on Influenza	
EPI711139	HE	Australia	2012-Jul-28	EPI_ISL_212077	C/Perth/9/2012	Pathwest QE II Medical Centre	WHO Collaborating Centre for Reference and Research on Influenza	
EPI675711	HE	Australia	2014-Aug-17	EPI_ISL_202512	C/Perth/9/2014	Pathwest QE II Medical Centre	WHO Collaborating Centre for Reference and Research on Influenza	Deng,Y-M.; Spirason,N.; Jelley,L.; Komadina,N.
EPI1183998	HE	Germany	2014-Apr-07	EPI_ISL_300546	C/Rheinland-Pfalz/14-03242/2014		Robert Koch Institute Nationales Referenzzentrum für Influenza	Biere, B.; Fritsch, A.; Schweiger, B.
EPI1183984	HE	Germany	2012-Mar-26	EPI_ISL_300532	C/Sachsen/12-02741/2012		Robert Koch Institute Nationales Referenzzentrum für Influenza	Biere, B.; Fritsch, A.; Schweiger, B.
EPI231880	HE	Japan	2000-Jan-01	EPI_ISL_66410	C/Saitama/1/2000		Import from public-domain	Matsuzaki,Y.
EPI231874	MP	Japan	2000-Jan-01	EPI_ISL_66410	C/Saitama/1/2000		Import from public-domain	Matsuzaki,Y.
EPI231875	NS	Japan	2000-Jan-01	EPI_ISL_66410	C/Saitama/1/2000		Import from public-domain	Matsuzaki,Y.
EPI228326	HE	Japan	2004-Jan-01	EPI_ISL_65162	C/Saitama/1/2004		Import from public-domain	Matsuzaki,Y.
EPI231883	HE	Japan	2000-Jan-01	EPI_ISL_66411	C/Saitama/2/2000		Import from public-domain	Matsuzaki,Y.
EPI231882	MP	Japan	2000-Jan-01	EPI_ISL_66411	C/Saitama/2/2000		Import from public-domain	Matsuzaki,Y.
EPI231881	NS	Japan	2000-Jan-01	EPI_ISL_66411	C/Saitama/2/2000		Import from public-domain	Matsuzaki,Y.
EPI231892	HE	Japan	2000-Jan-01	EPI_ISL_66412	C/Saitama/3/2000		Import from public-domain	Matsuzaki,Y.
EPI231893	MP	Japan	2000-Jan-01	EPI_ISL_66412	C/Saitama/3/2000		Import from public-domain	Matsuzaki,Y.
EPI231894	NS	Japan	2000-Jan-01	EPI_ISL_66412	C/Saitama/3/2000		Import from public-domain	Matsuzaki,Y.
EPI231547	HE	Brazil	1982-Jan-01	EPI_ISL_66344	C/Sao Paulo/378/82		Import from public-domain	Matsuzaki,Y.; Sugawara,K.; Furuse,Y.; Shimotai,Y.; Hongo,S.; Oshitani,H.; Mizuta,K.; Nishimura,H.
EPI231551	MP	Brazil	1982-Jan-01	EPI_ISL_66344	C/Sao Paulo/378/82		Import from public-domain	Matsuzaki,Y.; Sugawara,K.; Furuse,Y.; Shimotai,Y.; Hongo,S.; Oshitani,H.; Mizuta,K.; Nishimura,H.
EPI816588	NP	Brazil	1982-Jan-01	EPI_ISL_66344	C/Sao Paulo/378/82		Import from public-domain	Matsuzaki,Y.; Sugawara,K.; Furuse,Y.; Shimotai,Y.; Hongo,S.; Oshitani,H.; Mizuta,K.; Nishimura,H.
EPI231548	NS	Brazil	1982-Jan-01	EPI_ISL_66344	C/Sao Paulo/378/82		Import from public-domain	Matsuzaki,Y.; Sugawara,K.; Furuse,Y.; Shimotai,Y.; Hongo,S.; Oshitani,H.; Mizuta,K.; Nishimura,H.
EPI816489	P3	Brazil	1982-Jan-01	EPI_ISL_66344	C/Sao Paulo/378/82		Import from public-domain	Matsuzaki,Y.; Sugawara,K.; Furuse,Y.; Shimotai,Y.; Hongo,S.; Oshitani,H.; Mizuta,K.; Nishimura,H.
EPI815604	PB1	Brazil	1982-Jan-01	EPI_ISL_66344	C/Sao Paulo/378/82		Import from public-domain	Matsuzaki,Y.; Sugawara,K.; Furuse,Y.; Shimotai,Y.; Hongo,S.; Oshitani,H.; Mizuta,K.; Nishimura,H.
EPI813721	PB2	Brazil	1982-Jan-01	EPI_ISL_66344	C/Sao Paulo/378/82		Import from public-domain	Matsuzaki,Y.; Sugawara,K.; Furuse,Y.; Shimotai,Y.; Hongo,S.; Oshitani,H.; Mizuta,K.; Nishimura,H.
EPI232156	HE	Japan	1971-Jan-01	EPI_ISL_66325	C/Sapporo/71		Import from public-domain	Matsuzaki,Y.; Sugawara,K.; Furuse,Y.; Shimotai,Y.; Hongo,S.; Oshitani,H.; Mizuta,K.; Nishimura,H.
EPI232157	MP	Japan	1971-Jan-01	EPI_ISL_66325	C/Sapporo/71		Import from public-domain	Matsuzaki,Y.; Sugawara,K.; Furuse,Y.; Shimotai,Y.; Hongo,S.; Oshitani,H.; Mizuta,K.; Nishimura,H.
EPI816574	NP	Japan	1971-Jan-01	EPI_ISL_66325	C/Sapporo/71		Import from public-domain	Matsuzaki,Y.; Sugawara,K.; Furuse,Y.; Shimotai,Y.; Hongo,S.; Oshitani,H.; Mizuta,K.; Nishimura,H.
EPI231526	NS	Japan	1971-Jan-01	EPI_ISL_66325	C/Sapporo/71		Import from public-domain	Matsuzaki,Y.; Sugawara,K.; Furuse,Y.; Shimotai,Y.; Hongo,S.; Oshitani,H.; Mizuta,K.; Nishimura,H.
EPI816464	P3	Japan	1971-Jan-01	EPI_ISL_66325	C/Sapporo/71		Import from public-domain	Matsuzaki,Y.; Sugawara,K.; Furuse,Y.; Shimotai,Y.; Hongo,S.; Oshitani,H.; Mizuta,K.; Nishimura,H.
EPI813780	PB1	Japan	1971-Jan-01	EPI_ISL_66325	C/Sapporo/71		Import from public-domain	Matsuzaki,Y.; Sugawara,K.; Furuse,Y.; Shimotai,Y.; Hongo,S.; Oshitani,H.; Mizuta,K.; Nishimura,H.
EPI814887	PB2	Japan	1971-Jan-01	EPI_ISL_66325	C/Sapporo/71		Import from public-domain	Matsuzaki,Y.; Sugawara,K.; Furuse,Y.; Shimotai,Y.; Hongo,S.; Oshitani,H.; Mizuta,K.; Nishimura,H.
EPI1183988	HE	Germany	2012-Nov-27	EPI_ISL_300536	C/Schleswig-Holstein/13-00498/2012		Robert Koch Institute Nationales Referenzzentrum für Influenza	Biere, B.; Fritsch, A.; Schweiger, B.
EPI669896	NP	United Kingdom	2007-Jul-01	EPI_ISL_201031	C/Scotland/5500/2007		Import from public-domain	Smith,D.B.; Gaunt,E.R.; Digard,P.; Templeton,K.; Simmonds,P.
EPI669891	P3	United Kingdom	2007-Jul-01	EPI_ISL_201031	C/Scotland/5500/2007		Import from public-domain	Smith,D.B.; Gaunt,E.R.; Digard,P.; Templeton,K.; Simmonds,P.
EPI669877	PB1	United Kingdom	2007-Jul-01	EPI_ISL_201031	C/Scotland/5500/2007		Import from public-domain	Smith,D.B.; Gaunt,E.R.; Digard,P.; Templeton,K.; Simmonds,P.
EPI669886	PB2	United Kingdom	2007-Jul-01	EPI_ISL_201031	C/Scotland/5500/2007		Import from public-domain	Smith,D.B.; Gaunt,E.R.; Digard,P.; Templeton,K.; Simmonds,P.
EPI669892	HE	United Kingdom	2007-Nov-01	EPI_ISL_201034	C/Scotland/6855/2007		Import from public-domain	Smith,D.B.; Gaunt,E.R.; Digard,P.; Templeton,K.; Simmonds,P.
EPI669902	MP	United Kingdom	2007-Nov-01	EPI_ISL_201034	C/Scotland/6855/2007		Import from public-domain	Smith,D.B.; Gaunt,E.R.; Digard,P.; Templeton,K.; Simmonds,P.
EPI669901	NP	United Kingdom	2007-Nov-01	EPI_ISL_201034	C/Scotland/6855/2007		Import from public-domain	Smith,D.B.; Gaunt,E.R.; Digard,P.; Templeton,K.; Simmonds,P.
EPI671829	NS	United Kingdom	2007-Nov-01	EPI_ISL_201034	C/Scotland/6855/2007		Import from public-domain	Smith,D.B.; Gaunt,E.R.; Digard,P.; Templeton,K.; Simmonds,P.
EPI669890	P3	United Kingdom	2007-Nov-01	EPI_ISL_201034	C/Scotland/6855/2007		Import from public-domain	Smith,D.B.; Gaunt,E.R.; Digard,P.; Templeton,K.; Simmonds,P.
EPI669880	PB1	United Kingdom	2007-Nov-01	EPI_ISL_201034	C/Scotland/6855/2007		Import from public-domain	Smith,D.B.; Gaunt,E.R.; Digard,P.; Templeton,K.; Simmonds,P.
EPI669885	PB2	United Kingdom	2007-Nov-01	EPI_ISL_201034	C/Scotland/6855/2007		Import from public-domain	Smith,D.B.; Gaunt,E.R.; Digard,P.; Templeton,K.; Simmonds,P.
EPI669893	HE	United Kingdom	2007-Dec-01	EPI_ISL_201035	C/Scotland/7274/2007		Import from public-domain	Smith,D.B.; Gaunt,E.R.; Digard,P.; Templeton,K.; Simmonds,P.
EPI669903	MP	United Kingdom	2007-Dec-01	EPI_ISL_201035	C/Scotland/7274/2007		Import from public-domain	Smith,D.B.; Gaunt,E.R.; Digard,P.; Templeton,K.; Simmonds,P.
EPI669899	NP	United Kingdom	2007-Dec-01	EPI_ISL_201035	C/Scotland/7274/2007		Import from public-domain	Smith,D.B.; Gaunt,E.R.; Digard,P.; Templeton,K.; Simmonds,P.
EPI671832	NS	United Kingdom	2007-Dec-01	EPI_ISL_201035	C/Scotland/7274/2007		Import from public-domain	Smith,D.B.; Gaunt,E.R.; Digard,P.; Templeton,K.; Simmonds,P.
EPI669889	P3	United Kingdom	2007-Dec-01	EPI_ISL_201035	C/Scotland/7274/2007		Import from public-domain	Smith,D.B.; Gaunt,E.R.; Digard,P.; Templeton,K.; Simmonds,P.
EPI669881	PB1	United Kingdom	2007-Dec-01	EPI_ISL_201035	C/Scotland/7274/2007		Import from public-domain	Smith,D.B.; Gaunt,E.R.; Digard,P.; Templeton,K.; Simmonds,P.
EPI669884	PB2	United Kingdom	2007-Dec-01	EPI_ISL_201035	C/Scotland/7274/2007		Import from public-domain	Smith,D.B.; Gaunt,E.R.; Digard,P.; Templeton,K.; Simmonds,P.
EPI669894	HE	United Kingdom	2007-Dec-01	EPI_ISL_201033	C/Scotland/7383/2007		Import from public-domain	Smith,D.B.; Gaunt,E.R.; Digard,P.; Templeton,K.; Simmonds,P.
EPI669905	MP	United Kingdom	2007-Dec-01	EPI_ISL_201033	C/Scotland/7383/2007		Import from public-domain	Smith,D.B.; Gaunt,E.R.; Digard,P.; Templeton,K.; Simmonds,P.
EPI669897	NP	United Kingdom	2007-Dec-01	EPI_ISL_201033	C/Scotland/7383/2007		Import from public-domain	Smith,D.B.; Gaunt,E.R.; Digard,P.; Templeton,K.; Simmonds,P.
EPI671833	NS	United Kingdom	2007-Dec-01	EPI_ISL_201033	C/Scotland/7383/2007		Import from public-domain	Smith,D.B.; Gaunt,E.R.; Digard,P.; Templeton,K.; Simmonds,P.
EPI669888	P3	United Kingdom	2007-Dec-01	EPI_ISL_201033	C/Scotland/7383/2007		Import from public-domain	Smith,D.B.; Gaunt,E.R.; Digard,P.; Templeton,K.; Simmonds,P.
EPI669879	PB1	United Kingdom	2007-Dec-01	EPI_ISL_201033	C/Scotland/7383/2007		Import from public-domain	Smith,D.B.; Gaunt,E.R.; Digard,P.; Templeton,K.; Simmonds,P.
EPI669883	PB2	United Kingdom	2007-Dec-01	EPI_ISL_201033	C/Scotland/7383/2007		Import from public-domain	Smith,D.B.; Gaunt,E.R.; Digard,P.; Templeton,K.; Simmonds,P.
EPI669895	HE	United Kingdom	2007-Dec-01	EPI_ISL_201032	C/Scotland/7482/2007		Import from public-domain	Smith,D.B.; Gaunt,E.R.; Digard,P.; Templeton,K.; Simmonds,P.
EPI669904	MP	United Kingdom	2007-Dec-01	EPI_ISL_201032	C/Scotland/7482/2007		Import from public-domain	Smith,D.B.; Gaunt,E.R.; Digard,P.; Templeton,K.; Simmonds,P.
EPI671830	NS	United Kingdom	2007-Dec-01	EPI_ISL_201032	C/Scotland/7482/2007		Import from public-domain	Smith,D.B.; Gaunt,E.R.; Digard,P.; Templeton,K.; Simmonds,P.
EPI669887	P3	United Kingdom	2007-Dec-01	EPI_ISL_201032	C/Scotland/7482/2007		Import from public-domain	Smith,D.B.; Gaunt,E.R.; Digard,P.; Templeton,K.; Simmonds,P.
EPI669878	PB1	United Kingdom	2007-Dec-01	EPI_ISL_201032	C/Scotland/7482/2007		Import from public-domain	Smith,D.B.; Gaunt,E.R.; Digard,P.; Templeton,K.; Simmonds,P.
EPI669882	PB2	United Kingdom	2007-Dec-01	EPI_ISL_201032	C/Scotland/7482/2007		Import from public-domain	Smith,D.B.; Gaunt,E.R.; Digard,P.; Templeton,K.; Simmonds,P.
EPI581540	HE	Japan	2008-Jan-01	EPI_ISL_176775	C/Sendai/TU1/2008		Import from public-domain	Odagiri,T.; Matsuzaki,Y.; Okamoto,M.; Hongo,S.; Oshitani,H.
EPI621707	MP	Japan	2008-Jan-01	EPI_ISL_176775	C/Sendai/TU1/2008		Import from public-domain	Odagiri,T.; Matsuzaki,Y.; Okamoto,M.; Hongo,S.; Oshitani,H.
EPI621691	NS	Japan	2008-Jan-01	EPI_ISL_176775	C/Sendai/TU1/2008		Import from public-domain	Odagiri,T.; Matsuzaki,Y.; Okamoto,M.; Hongo,S.; Oshitani,H.
EPI581544	HE	Japan	2012-Jan-01	EPI_ISL_176779	C/Sendai/TU1/2012		Import from public-domain	Odagiri,T.; Matsuzaki,Y.; Okamoto,M.; Hongo,S.; Oshitani,H.
EPI621711	MP	Japan	2012-Jan-01	EPI_ISL_176779	C/Sendai/TU1/2012		Import from public-domain	Odagiri,T.; Matsuzaki,Y.; Okamoto,M.; Hongo,S.; Oshitani,H.
EPI621695	NS	Japan	2012-Jan-01	EPI_ISL_176779	C/Sendai/TU1/2012		Import from public-domain	Odagiri,T.; Matsuzaki,Y.; Okamoto,M.; Hongo,S.; Oshitani,H.
EPI581541	HE	Japan	2008-Jan-01	EPI_ISL_176776	C/Sendai/TU2/2008		Import from public-domain	Odagiri,T.; Matsuzaki,Y.; Okamoto,M.; Hongo,S.; Oshitani,H.
EPI621708	MP	Japan	2008-Jan-01	EPI_ISL_176776	C/Sendai/TU2/2008		Import from public-domain	Odagiri,T.; Matsuzaki,Y.; Okamoto,M.; Hongo,S.; Oshitani,H.
EPI621692	NS	Japan	2008-Jan-01	EPI_ISL_176776	C/Sendai/TU2/2008		Import from public-domain	Odagiri,T.; Matsuzaki,Y.; Okamoto,M.; Hongo,S.; Oshitani,H.
EPI581545	HE	Japan	2012-Jan-01	EPI_ISL_176780	C/Sendai/TU2/2012		Import from public-domain	Odagiri,T.; Matsuzaki,Y.; Okamoto,M.; Hongo,S.; Oshitani,H.
EPI621712	MP	Japan	2012-Jan-01	EPI_ISL_176780	C/Sendai/TU2/2012		Import from public-domain	Odagiri,T.; Matsuzaki,Y.; Okamoto,M.; Hongo,S.; Oshitani,H.
EPI621696	NS	Japan	2012-Jan-01	EPI_ISL_176780	C/Sendai/TU2/2012		Import from public-domain	Odagiri,T.; Matsuzaki,Y.; Okamoto,M.; Hongo,S.; Oshitani,H.
EPI581542	HE	Japan	2008-Jan-01	EPI_ISL_176777	C/Sendai/TU3/2008		Import from public-domain	Odagiri,T.; Matsuzaki,Y.; Okamoto,M.; Hongo,S.; Oshitani,H.
EPI621709	MP	Japan	2008-Jan-01	EPI_ISL_176777	C/Sendai/TU3/2008		Import from public-domain	Odagiri,T.; Matsuzaki,Y.; Okamoto,M.; Hongo,S.; Oshitani,H.
EPI621693	NS	Japan	2008-Jan-01	EPI_ISL_176777	C/Sendai/TU3/2008		Import from public-domain	Odagiri,T.; Matsuzaki,Y.; Okamoto,M.; Hongo,S.; Oshitani,H.
EPI581555	HE	Japan	2008-Jan-01	EPI_ISL_176996	C/Sendai/TU4/2008		Import from public-domain	Odagiri,T.; Matsuzaki,Y.; Okamoto,M.; Suzuki,A.; Saito,M.; Tamaki,R.; Lupisan,S.P.; Sombrero,L.T.; Hongo,S.; Oshitani,H.
EPI581543	HE	Japan	2008-Jan-01	EPI_ISL_176778	C/Sendai/TU5/2008		Import from public-domain	Odagiri,T.; Matsuzaki,Y.; Okamoto,M.; Hongo,S.; Oshitani,H.
EPI621710	MP	Japan	2008-Jan-01	EPI_ISL_176778	C/Sendai/TU5/2008		Import from public-domain	Odagiri,T.; Matsuzaki,Y.; Okamoto,M.; Hongo,S.; Oshitani,H.
EPI621694	NS	Japan	2008-Jan-01	EPI_ISL_176778	C/Sendai/TU5/2008		Import from public-domain	Odagiri,T.; Matsuzaki,Y.; Okamoto,M.; Hongo,S.; Oshitani,H.
EPI231902	HE	Japan	1979-Jan-01	EPI_ISL_66413	C/Shizuoka/79		Import from public-domain	Matsuzaki,Y.; Sugawara,K.; Furuse,Y.; Shimotai,Y.; Hongo,S.; Oshitani,H.; Mizuta,K.; Nishimura,H.
EPI231904	MP	Japan	1979-Jan-01	EPI_ISL_66413	C/Shizuoka/79		Import from public-domain	Matsuzaki,Y.; Sugawara,K.; Furuse,Y.; Shimotai,Y.; Hongo,S.; Oshitani,H.; Mizuta,K.; Nishimura,H.
EPI816581	NP	Japan	1979-Jan-01	EPI_ISL_66413	C/Shizuoka/79		Import from public-domain	Matsuzaki,Y.; Sugawara,K.; Furuse,Y.; Shimotai,Y.; Hongo,S.; Oshitani,H.; Mizuta,K.; Nishimura,H.
EPI231905	NS	Japan	1979-Jan-01	EPI_ISL_66413	C/Shizuoka/79		Import from public-domain	Matsuzaki,Y.; Sugawara,K.; Furuse,Y.; Shimotai,Y.; Hongo,S.; Oshitani,H.; Mizuta,K.; Nishimura,H.

Segment ID	Segment	Country	Collection date	Isolate-ID	Isolate name	Originating Lab	Submitting Lab	Authors
EPI816482	P3	Japan	1979-Jan-01	EPI_ISL_66413	C/Shizuoka/79		Import from public-domain	Matsuzaki,Y.; Sugawara,K.; Furuse,Y.; Shimotai,Y.; Hongo,S.; Oshitani,H.; Mizuta,K.; Nishimura,H.
EPI813790	PB1	Japan	1979-Jan-01	EPI_ISL_66413	C/Shizuoka/79		Import from public-domain	Matsuzaki,Y.; Sugawara,K.; Furuse,Y.; Shimotai,Y.; Hongo,S.; Oshitani,H.; Mizuta,K.; Nishimura,H.
EPI814536	PB2	Japan	1979-Jan-01	EPI_ISL_66413	C/Shizuoka/79		Import from public-domain	Matsuzaki,Y.; Sugawara,K.; Furuse,Y.; Shimotai,Y.; Hongo,S.; Oshitani,H.; Mizuta,K.; Nishimura,H.
EPI280421	HE	Singapore	2006-May-18	EPI_ISL_79749	C/Singapore/DSO-050530/2006		Import from public-domain	Ting,P.; Seah,S.L.K.; Lim,E.A.S.; Liaw,J.C.W.; Tan,B.H.
EPI509214	MP	Singapore	2006-May-18	EPI_ISL_79749	C/Singapore/DSO-050530/2006		Import from public-domain	Ting,P.; Seah,S.L.K.; Lim,E.A.S.; Liaw,J.C.W.; Tan,B.H.
EPI509218	NP	Singapore	2006-May-18	EPI_ISL_79749	C/Singapore/DSO-050530/2006		Import from public-domain	Ting,P.; Seah,S.L.K.; Lim,E.A.S.; Liaw,J.C.W.; Tan,B.H.
EPI509223	NS	Singapore	2006-May-18	EPI_ISL_79749	C/Singapore/DSO-050530/2006		Import from public-domain	Ting,P.; Seah,S.L.K.; Lim,E.A.S.; Liaw,J.C.W.; Tan,B.H.
EPI509227	P3	Singapore	2006-May-18	EPI_ISL_79749	C/Singapore/DSO-050530/2006		Import from public-domain	Ting,P.; Seah,S.L.K.; Lim,E.A.S.; Liaw,J.C.W.; Tan,B.H.
EPI509231	PB1	Singapore	2006-May-18	EPI_ISL_79749	C/Singapore/DSO-050530/2006		Import from public-domain	Ting,P.; Seah,S.L.K.; Lim,E.A.S.; Liaw,J.C.W.; Tan,B.H.
EPI509235	PB2	Singapore	2006-May-18	EPI_ISL_79749	C/Singapore/DSO-050530/2006		Import from public-domain	Ting,P.; Seah,S.L.K.; Lim,E.A.S.; Liaw,J.C.W.; Tan,B.H.
EPI280419	HE	Singapore	2006-Nov-17	EPI_ISL_79747	C/Singapore/DSO-070170/2006		Import from public-domain	Ting,P.; Seah,S.L.K.; Lim,E.A.S.; Liaw,J.C.W.; Tan,B.H.
EPI509215	MP	Singapore	2006-Nov-17	EPI_ISL_79747	C/Singapore/DSO-070170/2006		Import from public-domain	Ting,P.; Seah,S.L.K.; Lim,E.A.S.; Liaw,J.C.W.; Tan,B.H.
EPI509219	NP	Singapore	2006-Nov-17	EPI_ISL_79747	C/Singapore/DSO-070170/2006		Import from public-domain	Ting,P.; Seah,S.L.K.; Lim,E.A.S.; Liaw,J.C.W.; Tan,B.H.
EPI509224	NS	Singapore	2006-Nov-17	EPI_ISL_79747	C/Singapore/DSO-070170/2006		Import from public-domain	Ting,P.; Seah,S.L.K.; Lim,E.A.S.; Liaw,J.C.W.; Tan,B.H.
EPI509228	P3	Singapore	2006-Nov-17	EPI_ISL_79747	C/Singapore/DSO-070170/2006		Import from public-domain	Ting,P.; Seah,S.L.K.; Lim,E.A.S.; Liaw,J.C.W.; Tan,B.H.
EPI509232	PB1	Singapore	2006-Nov-17	EPI_ISL_79747	C/Singapore/DSO-070170/2006		Import from public-domain	Ting,P.; Seah,S.L.K.; Lim,E.A.S.; Liaw,J.C.W.; Tan,B.H.
EPI509236	PB2	Singapore	2006-Nov-17	EPI_ISL_79747	C/Singapore/DSO-070170/2006		Import from public-domain	Ting,P.; Seah,S.L.K.; Lim,E.A.S.; Liaw,J.C.W.; Tan,B.H.
EPI280420	HE	Singapore	2006-Nov-29	EPI_ISL_79748	C/Singapore/DSO-070193/2006		Import from public-domain	Ting,P.; Seah,S.L.K.; Lim,E.A.S.; Liaw,J.C.W.; Tan,B.H.
EPI509216	MP	Singapore	2006-Nov-29	EPI_ISL_79748	C/Singapore/DSO-070193/2006		Import from public-domain	Ting,P.; Seah,S.L.K.; Lim,E.A.S.; Liaw,J.C.W.; Tan,B.H.
EPI509220	NP	Singapore	2006-Nov-29	EPI_ISL_79748	C/Singapore/DSO-070193/2006		Import from public-domain	Ting,P.; Seah,S.L.K.; Lim,E.A.S.; Liaw,J.C.W.; Tan,B.H.
EPI509225	NS	Singapore	2006-Nov-29	EPI_ISL_79748	C/Singapore/DSO-070193/2006		Import from public-domain	Ting,P.; Seah,S.L.K.; Lim,E.A.S.; Liaw,J.C.W.; Tan,B.H.
EPI509229	P3	Singapore	2006-Nov-29	EPI_ISL_79748	C/Singapore/DSO-070193/2006		Import from public-domain	Ting,P.; Seah,S.L.K.; Lim,E.A.S.; Liaw,J.C.W.; Tan,B.H.
EPI509233	PB1	Singapore	2006-Nov-29	EPI_ISL_79748	C/Singapore/DSO-070193/2006		Import from public-domain	Ting,P.; Seah,S.L.K.; Lim,E.A.S.; Liaw,J.C.W.; Tan,B.H.
EPI509237	PB2	Singapore	2006-Nov-29	EPI_ISL_79748	C/Singapore/DSO-070193/2006		Import from public-domain	Ting,P.; Seah,S.L.K.; Lim,E.A.S.; Liaw,J.C.W.; Tan,B.H.
EPI280418	HE	Singapore	2006-Dec-04	EPI_ISL_79746	C/Singapore/DSO-070203/2006		Import from public-domain	Ting,P.; Seah,S.L.K.; Lim,E.A.S.; Liaw,J.C.W.; Tan,B.H.
EPI509217	MP	Singapore	2006-Dec-04	EPI_ISL_79746	C/Singapore/DSO-070203/2006		Import from public-domain	Ting,P.; Seah,S.L.K.; Lim,E.A.S.; Liaw,J.C.W.; Tan,B.H.
EPI509221	NP	Singapore	2006-Dec-04	EPI_ISL_79746	C/Singapore/DSO-070203/2006		Import from public-domain	Ting,P.; Seah,S.L.K.; Lim,E.A.S.; Liaw,J.C.W.; Tan,B.H.
EPI509226	NS	Singapore	2006-Dec-04	EPI_ISL_79746	C/Singapore/DSO-070203/2006		Import from public-domain	Ting,P.; Seah,S.L.K.; Lim,E.A.S.; Liaw,J.C.W.; Tan,B.H.
EPI509230	P3	Singapore	2006-Dec-04	EPI_ISL_79746	C/Singapore/DSO-070203/2006		Import from public-domain	Ting,P.; Seah,S.L.K.; Lim,E.A.S.; Liaw,J.C.W.; Tan,B.H.
EPI509234	PB1	Singapore	2006-Dec-04	EPI_ISL_79746	C/Singapore/DSO-070203/2006		Import from public-domain	Ting,P.; Seah,S.L.K.; Lim,E.A.S.; Liaw,J.C.W.; Tan,B.H.
EPI509238	PB2	Singapore	2006-Dec-04	EPI_ISL_79746	C/Singapore/DSO-070203/2006		Import from public-domain	Ting,P.; Seah,S.L.K.; Lim,E.A.S.; Liaw,J.C.W.; Tan,B.H.
EPI813649	HE	United States	1947-Jan-01	EPI_ISL_230234	C/Taylor/1233/1947		Import from public-domain	Matsuzaki,Y.; Sugawara,K.; Furuse,Y.; Shimotai,Y.; Hongo,S.; Oshitani,H.; Mizuta,K.; Nishimura,H.
EPI816458	P3	United States	1947-Jan-01	EPI_ISL_230234	C/Taylor/1233/1947		Import from public-domain	Matsuzaki,Y.; Sugawara,K.; Furuse,Y.; Shimotai,Y.; Hongo,S.; Oshitani,H.; Mizuta,K.; Nishimura,H.
EPI813768	PB1	United States	1947-Jan-01	EPI_ISL_230234	C/Taylor/1233/1947		Import from public-domain	Matsuzaki,Y.; Sugawara,K.; Furuse,Y.; Shimotai,Y.; Hongo,S.; Oshitani,H.; Mizuta,K.; Nishimura,H.
EPI813693	PB2	United States	1947-Jan-01	EPI_ISL_230234	C/Taylor/1233/1947		Import from public-domain	Matsuzaki,Y.; Sugawara,K.; Furuse,Y.; Shimotai,Y.; Hongo,S.; Oshitani,H.; Mizuta,K.; Nishimura,H.
EPI232199	MP		1947-Jan-01	EPI_ISL_66449	C/Taylor/1233/47		Import from public-domain	Hidekazu,N.
EPI816736	NS		1947-Jan-01	EPI_ISL_230234	C/Taylor/1233/47		Import from public-domain	Matsuzaki,Y.; Sugawara,K.; Furuse,Y.; Shimotai,Y.; Hongo,S.; Oshitani,H.; Mizuta,K.; Nishimura,H.
EPI1183982	HE	Germany	2012-Mar-12	EPI_ISL_300530	C/Thueringen/12-02332/2012		Robert Koch Institute Nationales Referenzzentrum für Influenza	Biere, B.; Fritsch, A.; Schweiger, B.
EPI1183983	HE	Germany	2012-Mar-19	EPI_ISL_300531	C/Thueringen/12-02562/2012		Robert Koch Institute Nationales Referenzzentrum für Influenza	Biere, B.; Fritsch, A.; Schweiger, B.
EPI1183985	HE	Germany	2012-Oct-29	EPI_ISL_300533	C/Thueringen/13-00167/2012		Robert Koch Institute Nationales Referenzzentrum für Influenza	Biere, B.; Fritsch, A.; Schweiger, B.
EPI1183989	HE	Germany	2012-Nov-28	EPI_ISL_300537	C/Thueringen/13-00580/2012		Robert Koch Institute Nationales Referenzzentrum für Influenza	Biere, B.; Fritsch, A.; Schweiger, B.
EPI813691	HE	Japan	2014-Feb-01	EPI_ISL_230276	C/Tokyo/1/2014		Import from public-domain	Matsuzaki,Y.; Sugawara,K.; Furuse,Y.; Shimotai,Y.; Hongo,S.; Oshitani,H.; Mizuta,K.; Nishimura,H.
EPI816734	MP	Japan	2014-Feb-01	EPI_ISL_230276	C/Tokyo/1/2014		Import from public-domain	Matsuzaki,Y.; Sugawara,K.; Furuse,Y.; Shimotai,Y.; Hongo,S.; Oshitani,H.; Mizuta,K.; Nishimura,H.
EPI816666	NP	Japan	2014-Feb-01	EPI_ISL_230276	C/Tokyo/1/2014		Import from public-domain	Matsuzaki,Y.; Sugawara,K.; Furuse,Y.; Shimotai,Y.; Hongo,S.; Oshitani,H.; Mizuta,K.; Nishimura,H.
EPI816803	NS	Japan	2014-Feb-01	EPI_ISL_230276	C/Tokyo/1/2014		Import from public-domain	Matsuzaki,Y.; Sugawara,K.; Furuse,Y.; Shimotai,Y.; Hongo,S.; Oshitani,H.; Mizuta,K.; Nishimura,H.
EPI816567	P3	Japan	2014-Feb-01	EPI_ISL_230276	C/Tokyo/1/2014		Import from public-domain	Matsuzaki,Y.; Sugawara,K.; Furuse,Y.; Shimotai,Y.; Hongo,S.; Oshitani,H.; Mizuta,K.; Nishimura,H.
EPI816456	PB1	Japan	2014-Feb-01	EPI_ISL_230276	C/Tokyo/1/2014		Import from public-domain	Matsuzaki,Y.; Sugawara,K.; Furuse,Y.; Shimotai,Y.; Hongo,S.; Oshitani,H.; Mizuta,K.; Nishimura,H.
EPI813766	PB2	Japan	2014-Feb-01	EPI_ISL_230276	C/Tokyo/1/2014		Import from public-domain	Matsuzaki,Y.; Sugawara,K.; Furuse,Y.; Shimotai,Y.; Hongo,S.; Oshitani,H.; Mizuta,K.; Nishimura,H.
EPI813680	HE	Japan	2010-Apr-23	EPI_ISL_230265	C/Tokyo/3/2010		Import from public-domain	Matsuzaki,Y.; Sugawara,K.; Furuse,Y.; Shimotai,Y.; Hongo,S.; Oshitani,H.; Mizuta,K.; Nishimura,H.
EPI816714	MP	Japan	2010-Apr-23	EPI_ISL_230265	C/Tokyo/3/2010		Import from public-domain	Matsuzaki,Y.; Sugawara,K.; Furuse,Y.; Shimotai,Y.; Hongo,S.; Oshitani,H.; Mizuta,K.; Nishimura,H.
EPI816646	NP	Japan	2010-Apr-23	EPI_ISL_230265	C/Tokyo/3/2010		Import from public-domain	Matsuzaki,Y.; Sugawara,K.; Furuse,Y.; Shimotai,Y.; Hongo,S.; Oshitani,H.; Mizuta,K.; Nishimura,H.
EPI816783	NS	Japan	2010-Apr-23	EPI_ISL_230265	C/Tokyo/3/2010		Import from public-domain	Matsuzaki,Y.; Sugawara,K.; Furuse,Y.; Shimotai,Y.; Hongo,S.; Oshitani,H.; Mizuta,K.; Nishimura,H.
EPI816547	P3	Japan	2010-Apr-23	EPI_ISL_230265	C/Tokyo/3/2010		Import from public-domain	Matsuzaki,Y.; Sugawara,K.; Furuse,Y.; Shimotai,Y.; Hongo,S.; Oshitani,H.; Mizuta,K.; Nishimura,H.
EPI816443	PB1	Japan	2010-Apr-23	EPI_ISL_230265	C/Tokyo/3/2010		Import from public-domain	Matsuzaki,Y.; Sugawara,K.; Furuse,Y.; Shimotai,Y.; Hongo,S.; Oshitani,H.; Mizuta,K.; Nishimura,H.
EPI813753	PB2	Japan	2010-Apr-23	EPI_ISL_230265	C/Tokyo/3/2010		Import from public-domain	Matsuzaki,Y.; Sugawara,K.; Furuse,Y.; Shimotai,Y.; Hongo,S.; Oshitani,H.; Mizuta,K.; Nishimura,H.
EPI813692	HE	Japan	2014-Apr-23	EPI_ISL_230277	C/Tokyo/4/2014		Import from public-domain	Matsuzaki,Y.; Sugawara,K.; Furuse,Y.; Shimotai,Y.; Hongo,S.; Oshitani,H.; Mizuta,K.; Nishimura,H.
EPI816735	MP	Japan	2014-Apr-23	EPI_ISL_230277	C/Tokyo/4/2014		Import from public-domain	Matsuzaki,Y.; Sugawara,K.; Furuse,Y.; Shimotai,Y.; Hongo,S.; Oshitani,H.; Mizuta,K.; Nishimura,H.
EPI816667	NP	Japan	2014-Apr-23	EPI_ISL_230277	C/Tokyo/4/2014		Import from public-domain	Matsuzaki,Y.; Sugawara,K.; Furuse,Y.; Shimotai,Y.; Hongo,S.; Oshitani,H.; Mizuta,K.; Nishimura,H.
EPI816804	NS	Japan	2014-Apr-23	EPI_ISL_230277	C/Tokyo/4/2014		Import from public-domain	Matsuzaki,Y.; Sugawara,K.; Furuse,Y.; Shimotai,Y.; Hongo,S.; Oshitani,H.; Mizuta,K.; Nishimura,H.
EPI816568	P3	Japan	2014-Apr-23	EPI_ISL_230277	C/Tokyo/4/2014		Import from public-domain	Matsuzaki,Y.; Sugawara,K.; Furuse,Y.; Shimotai,Y.; Hongo,S.; Oshitani,H.; Mizuta,K.; Nishimura,H.
EPI816457	PB1	Japan	2014-Apr-23	EPI_ISL_230277	C/Tokyo/4/2014		Import from public-domain	Matsuzaki,Y.; Sugawara,K.; Furuse,Y.; Shimotai,Y.; Hongo,S.; Oshitani,H.; Mizuta,K.; Nishimura,H.
EPI813767	PB2	Japan	2014-Apr-23	EPI_ISL_230277	C/Tokyo/4/2014		Import from public-domain	Matsuzaki,Y.; Sugawara,K.; Furuse,Y.; Shimotai,Y.; Hongo,S.; Oshitani,H.; Mizuta,K.; Nishimura,H.
EPI1564379	HE	United States	2011-Jan-01	EPI_ISL_380251	C/Victoria/1/2011	St. Jude Children's Research Hospital	Crick Worldwide Influenza Centre	
EPI1595542	MP	United States	2011-Jan-01	EPI_ISL_380251	C/Victoria/1/2011	St. Jude Children's Research Hospital	Crick Worldwide Influenza Centre	
EPI1558360	NP	United States	2011-Jan-01	EPI_ISL_380251	C/Victoria/1/2011	St. Jude Children's Research Hospital	Crick Worldwide Influenza Centre	
EPI1607370	NS	United States	2011-Jan-01	EPI_ISL_380251	C/Victoria/1/2011	St. Jude Children's Research Hospital	Crick Worldwide Influenza Centre	
EPI1566811	P3	United States	2011-Jan-01	EPI_ISL_380251	C/Victoria/1/2011	St. Jude Children's Research Hospital	Crick Worldwide Influenza Centre	
EPI1567405	PB1	United States	2011-Jan-01	EPI_ISL_380251	C/Victoria/1/2011	St. Jude Children's Research Hospital	Crick Worldwide Influenza Centre	
EPI1590465	PB2	United States	2011-Jan-01	EPI_ISL_380251	C/Victoria/1/2011	St. Jude Children's Research Hospital	Crick Worldwide Influenza Centre	
EPI711148	HE	Australia	2012-Aug-13	EPI_ISL_212086	C/Victoria/2/2012	Victorian Infectious Diseases Reference Laboratory	WHO Collaborating Centre for Reference and Research on Influenza	
EPI545175	MP	Australia	2012-Nov-01	EPI_ISL_167197	C/Victoria/2/2012		Import from public-domain	Liu,R.; Hause,B.M.; Li,F.
EPI545174	NP	Australia	2012-Nov-01	EPI_ISL_167197	C/Victoria/2/2012		Import from public-domain	Liu,R.; Hause,B.M.; Li,F.
EPI545176	NS	Australia	2012-Nov-01	EPI_ISL_167197	C/Victoria/2/2012		Import from public-domain	Liu,R.; Hause,B.M.; Li,F.
EPI545172	P3	Australia	2012-Nov-01	EPI_ISL_167197	C/Victoria/2/2012		Import from public-domain	Liu,R.; Hause,B.M.; Li,F.
EPI545171	PB1	Australia	2012-Nov-01	EPI_ISL_167197	C/Victoria/2/2012		Import from public-domain	Liu,R.; Hause,B.M.; Li,F.
EPI545170	PB2	Australia	2012-Nov-01	EPI_ISL_167197	C/Victoria/2/2012		Import from public-domain	Liu,R.; Hause,B.M.; Li,F.
EPI711150	HE	Australia	2014-May-07	EPI_ISL_212088	C/Victoria/2/2014	Victorian Infectious Diseases Reference Laboratory	WHO Collaborating Centre for Reference and Research on Influenza	
EPI711151	HE	Australia	2014-Aug-12	EPI_ISL_212089	C/Victoria/3/2014	Victorian Infectious Diseases Reference Laboratory	WHO Collaborating Centre for Reference and Research on Influenza	
EPI711149	HE	Australia	2012-Oct-08	EPI_ISL_212087	C/Victoria/4/2012	Victorian Infectious Diseases Reference Laboratory	WHO Collaborating Centre for Reference and Research on Influenza	
EPI711152	HE	Australia	2014-Aug-08	EPI_ISL_212090	C/Victoria/4/2014	Victorian Infectious Diseases Reference Laboratory	WHO Collaborating Centre for Reference and Research on Influenza	
EPI813662	HE	Japan	2005-Jun-07	EPI_ISL_230247	C/Yamagata/1/2005		Import from public-domain	Matsuzaki,Y.; Sugawara,K.; Furuse,Y.; Shimotai,Y.; Hongo,S.; Oshitani,H.; Mizuta,K.; Nishimura,H.
EPI816693	MP	Japan	2005-Jun-07	EPI_ISL_230247	C/Yamagata/1/2005		Import from public-domain	Matsuzaki,Y.; Sugawara,K.; Furuse,Y.; Shimotai,Y.; Hongo,S.; Oshitani,H.; Mizuta,K.; Nishimura,H.
EPI816628	NP	Japan	2005-Jun-07	EPI_ISL_230247	C/Yamagata/1/2005		Import from public-domain	Matsuzaki,Y.; Sugawara,K.; Furuse,Y.; Shimotai,Y.; Hongo,S.; Oshitani,H.; Mizuta,K.; Nishimura,H.
EPI816762	NS	Japan	2005-Jun-07	EPI_ISL_230247	C/Yamagata/1/2005		Import from public-domain	Matsuzaki,Y.; Sugawara,K.; Furuse,Y.; Shimotai,Y.; Hongo,S.; Oshitani,H.; Mizuta,K.; Nishimura,H.
EPI816526	P3	Japan	2005-Jun-07	EPI_ISL_230247	C/Yamagata/1/2005		Import from public-domain	Matsuzaki,Y.; Sugawara,K.; Furuse,Y.; Shimotai,Y.; Hongo,S.; Oshitani,H.; Mizuta,K.; Nishimura,H.
EPI813808	PB1	Japan	2005-Jun-07	EPI_ISL_230247	C/Yamagata/1/2005		Import from public-domain	Matsuzaki,Y.; Sugawara,K.; Furuse,Y.; Shimotai,Y.; Hongo,S.; Oshitani,H.; Mizuta,K.; Nishimura,H.
EPI813711	PB2	Japan	2005-Jun-07	EPI_ISL_230247	C/Yamagata/1/2005		Import from public-domain	Matsuzaki,Y.; Sugawara,K.; Furuse,Y.; Shimotai,Y.; Hongo,S.; Oshitani,H.; Mizuta,K.; Nishimura,H.
EPI813669	HE	Japan	2006-Jan-23	EPI_ISL_230254	C/Yamagata/1/2006		Import from public-domain	Matsuzaki,Y.; Sugawara,K.; Furuse,Y.; Shimotai,Y.; Hongo,S.; Oshitani,H.; Mizuta,K.; Nishimura,H.
EPI816700	MP	Japan	2006-Jan-23	EPI_ISL_230254	C/Yamagata/1/2006		Import from public-domain	Matsuzaki,Y.; Sugawara,K.; Furuse,Y.; Shimotai,Y.; Hongo,S.; Oshitani,H.; Mizuta,K.; Nishimura,H.
EPI816634	NP	Japan	2006-Jan-23	EPI_ISL_230254	C/Yamagata/1/2006		Import from public-domain	Matsuzaki,Y.; Sugawara,K.; Furuse,Y.; Shimotai,Y.; Hongo,S.; Oshitani,H.; Mizuta,K.; Nishimura,H.
EPI816769	NS	Japan	2006-Jan-23	EPI_ISL_230254	C/Yamagata/1/2006		Import from public-domain	Matsuzaki,Y.; Sugawara,K.; Furuse,Y.; Shimotai,Y.; Hongo,S.; Oshitani,H.; Mizuta,K.; Nishimura,H.
EPI816533	P3	Japan	2006-Jan-23	EPI_ISL_230254	C/Yamagata/1/2006		Import from public-domain	Matsuzaki,Y.; Sugawara,K.; Furuse,Y.; Shimotai,Y.; Hongo,S.; Oshitani,H.; Mizuta,K.; Nishimura,H.
EPI816431	PB1	Japan	2006-Jan-23	EPI_ISL_230254	C/Yamagata/1/2006		Import from public-domain	Matsuzaki,Y.; Sugawara,K.; Furuse,Y.; Shimotai,Y.; Hongo,S.; Oshitani,H.; Mizuta,K.; Nishimura,H.
EPI813718	PB2	Japan	2006-Jan-23	EPI_ISL_230254	C/Yamagata/1/2006		Import from public-domain	Matsuzaki,Y.; Sugawara,K.; Furuse,Y.; Shimotai,Y.; Hongo,S.; Oshitani,H.; Mizuta,K.; Nishimura,H.
EPI813674	HE	Japan	2007-Jan-17	EPI_ISL_230259	C/Yamagata/1/2007		Import from public-domain	Matsuzaki,Y.; Sugawara,K.; Furuse,Y.; Shimotai,Y.; Hongo,S.; Oshitani,H.; Mizuta,K.; Nishimura,H.
EPI816706	MP	Japan	2007-Jan-17	EPI_ISL_230259	C/Yamagata/1/2007		Import from public-domain	Matsuzaki,Y.; Sugawara,K.; Furuse,Y.; Shimotai,Y.; Hongo,S.; Oshitani,H.; Mizuta,K.; Nishimura,H.
EPI369790	NP	Japan	2007-Jan-17	EPI_ISL_118425	C/Yamagata/1/2007		Import from public-domain	Matsuzaki,Y.; Ikeda,T.; Abiko,C.; Aoki,Y.; Mizuta,K.; Shimotai,Y.; Sugawara,K.; Hongo,S.
EPI816775	NS	Japan	2007-Jan-17	EPI_ISL_230259	C/Yamagata/1/2007		Import from public-domain	Matsuzaki,Y.; Sugawara,K.; Furuse,Y.; Shimotai,Y.; Hongo,S.; Oshitani,H.; Mizuta,K.; Nishimura,H.
EPI816539	P3	Japan	2007-Jan-17	EPI_ISL_230259	C/Yamagata/1/2007		Import from public-domain	Matsuzaki,Y.; Sugawara,K.; Furuse,Y.; Shimotai,Y.; Hongo,S.; Oshitani,H.; Mizuta,K.; Nishimura,H.
EPI816436	PB1	Japan	2007-Jan-17	EPI_ISL_230259	C/Yamagata/1/2007		Import from public-domain	Matsuzaki,Y.; Sugawara,K.; Furuse,Y.; Shimotai,Y.; Hongo,S.; Oshitani,H.; Mizuta,K.; Nishimura,H.
EPI813746	PB2	Japan	2007-Jan-17	EPI_ISL_230259	C/Yamagata/1/2007		Import from public-domain	Matsuzaki,Y.; Sugawara,K.; Furuse,Y.; Shimotai,Y.; Hongo,S.; Oshitani,H.; Mizuta,K.; Nishimura,H.
EPI813681	HE	Japan	2012-Jan-16	EPI_ISL_230266	C/Yamagata/1/2012		Import from public-domain	Matsuzaki,Y.; Sugawara,K.; Furuse,Y.; Shimotai,Y.; Hongo,S.; Oshitani,H.; Mizuta,K.; Nishimura,H.
EPI816715	MP	Japan	2012-Jan-16	EPI_ISL_230266	C/Yamagata/1/2012		Import from public-domain	Matsuzaki,Y.; Sugawara,K.; Furuse,Y.; Shimotai,Y.; Hongo,S.; Oshitani,H.; Mizuta,K.; Nishimura,H.
EPI816647	NP	Japan	2012-Jan-16	EPI_ISL_230266	C/Yamagata/1/2012		Import from public-domain	Matsuzaki,Y.; Sugawara,K.; Furuse,Y.; Shimotai,Y.; Hongo,S.; Oshitani,H.; Mizuta,K.; Nishimura,H.
EPI816784	NS	Japan	2012-Jan-16	EPI_ISL_230266	C/Yamagata/1/2012		Import from public-domain	Matsuzaki,Y.; Sugawara,K.; Furuse,Y.; Shimotai,Y.; Hongo,S.; Oshitani,H.; Mizuta,K.; Nishimura,H.
EPI816548	P3	Japan	2012-Jan-16	EPI_ISL_230266	C/Yamagata/1/2012		Import from public-domain	Matsuzaki,Y.; Sugawara,K.; Furuse,Y.; Shimotai,Y.; Hongo,S.; Oshitani,H.; Mizuta,K.; Nishimura,H.
EPI816444	PB1	Japan	2012-Jan-16	EPI_ISL_230266	C/Yamagata/1/2012		Import from public-domain	Matsuzaki,Y.; Sugawara,K.; Furuse,Y.; Shimotai,Y.; Hongo,S.; Oshitani,H.; Mizuta,K.; Nishimura,H.
EPI813754	PB2	Japan	2012-Jan-16	EPI_ISL_230266	C/Yamagata/1/2012		Import from public-domain	Matsuzaki,Y.; Sugawara,K.; Furuse,Y.; Shimotai,Y.; Hongo,S.; Oshitani,H.; Mizuta,K.; Nishimura,H.
EPI231906	HE	Japan	1986-Jan-01	EPI_ISL_66347	C/Yamagata/1/86		Import from public-domain	Matsuzaki,Y.
EPI232158	MP	Japan	1986-Jan-01	EPI_ISL_66347	C/Yamagata/1/86		Import from public-domain	Matsuzaki,Y.
EPI231556	NS	Japan	1986-Jan-01	EPI_ISL_66347	C/Yamagata/1/86		Import from public-domain	Matsuzaki,Y.
EPI231912	MP	Japan	1988-Jan-01	EPI_ISL_66414	C/Yamagata/1/88		Import from public-domain	Hongo,S.
EPI231911	NS	Japan	1988-Jan-01	EPI_ISL_66414	C/Yamagata/1/88		Import from public-domain	Hongo,S.
EPI231565	NS	Japan	1990-Jan-01	EPI_ISL_66354	C/Yamagata/1/90		Import from public-domain	Matsuzaki,Y.
EPI231573	HE	Japan	1993-Apr-30	EPI_ISL_66362	C/Yamagata/1/93		Import from public-domain	Matsuzaki,Y.; Sugawara,K.; Furuse,Y.; Shimotai,Y.; Hongo,S.; Oshitani,H.; Mizuta,K.; Nishimura,H.
EPI231577	MP	Japan	1993-Apr-30	EPI_ISL_66362	C/Yamagata/1/93		Import from public-domain	Matsuzaki,Y.; Sugawara,K.; Furuse,Y.; Shimotai,Y.; Hongo,S.; Oshitani,H.; Mizuta,K.; Nishimura,H.
EPI816597	NP	Japan	1993-Apr-30	EPI_ISL_66362	C/Yamagata/1/93		Import from public-domain	Matsuzaki,Y.; Sugawara,K.; Furuse,Y.; Shimotai,Y.; Hongo,S.; Oshitani,H.; Mizuta,K.; Nishimura,H.
EPI231574	NS	Japan	1993-Apr-30	EPI_ISL_66362	C/Yamagata/1/93		Import from public-domain	Matsuzaki,Y.; Sugawara,K.; Furuse,Y.; Shimotai,Y.; Hongo,S.; Oshitani,H.; Mizuta,K.; Nishimura,H.
EPI816498	P3	Japan	1993-Apr-30	EPI_ISL_66362	C/Yamagata/1/93		Import from public-domain	Matsuzaki,Y.; Sugawara,K.; Furuse,Y.; Shimotai,Y.; Hongo,S.; Oshitani,H.; Mizuta,K.; Nishimura,H.
EPI816415	PB1	Japan	1993-Apr-30	EPI_ISL_66362	C/Yamagata/1/93		Import from public-domain	Matsuzaki,Y.; Sugawara,K.; Furuse,Y.; Shimotai,Y.; Hongo,S.; Oshitani,H.; Mizuta,K.; Nishimura,H.
EPI813741	PB2	Japan	1993-Apr-30	EPI_ISL_66362	C/Yamagata/1/93		Import from public-domain	Matsuzaki,Y.; Sugawara,K.; Furuse,Y.; Shimotai,Y.; Hongo,S.; Oshitani,H.; Mizuta,K.; Nishimura,H.
EPI816673	MP	Japan	1981-Mar-18	EPI_ISL_230279	C/Yamagata/10/1981		Import from public-domain	Matsuzaki,Y.; Sugawara,K.; Furuse,Y.; Shimotai,Y.; Hongo,S.; Oshitani,H.; Mizuta,K.; Nishimura,H.

Segment ID	Segment	Country	Collection date	Isolate-ID	Isolate name	Originating Lab	Submitting Lab	Authors
EPI816585	NP	Japan	1981-Mar-18	EPI_ISL_230279	C/Yamagata/10/1981		Import from public-domain	Matsuzaki,Y.; Sugawara,K.; Furuse,Y.; Shimotai,Y.; Hongo,S.; Oshitani,H.; Mizuta,K.; Nishimura,H.
EPI816742	NS	Japan	1981-Mar-18	EPI_ISL_230279	C/Yamagata/10/1981		Import from public-domain	Matsuzaki,Y.; Sugawara,K.; Furuse,Y.; Shimotai,Y.; Hongo,S.; Oshitani,H.; Mizuta,K.; Nishimura,H.
EPI816486	P3	Japan	1981-Mar-18	EPI_ISL_230279	C/Yamagata/10/1981		Import from public-domain	Matsuzaki,Y.; Sugawara,K.; Furuse,Y.; Shimotai,Y.; Hongo,S.; Oshitani,H.; Mizuta,K.; Nishimura,H.
EPI813793	PB1	Japan	1981-Mar-18	EPI_ISL_230279	C/Yamagata/10/1981		Import from public-domain	Matsuzaki,Y.; Sugawara,K.; Furuse,Y.; Shimotai,Y.; Hongo,S.; Oshitani,H.; Mizuta,K.; Nishimura,H.
EPI813696	PB2	Japan	1981-Mar-18	EPI_ISL_230279	C/Yamagata/10/1981		Import from public-domain	Matsuzaki,Y.; Sugawara,K.; Furuse,Y.; Shimotai,Y.; Hongo,S.; Oshitani,H.; Mizuta,K.; Nishimura,H.
EPI231539	HE	Japan	1981-Jan-01	EPI_ISL_66338	C/Yamagata/10/81		Import from public-domain	Buonagurio,D.A.; Nakada,S.; Desselberger,U.; Krystal,M.; Palese,P.
EPI231913	HE	Japan	1989-Aug-03	EPI_ISL_66360	C/Yamagata/10/89		Import from public-domain	Matsuzaki,Y.; Sugawara,K.; Furuse,Y.; Shimotai,Y.; Hongo,S.; Oshitani,H.; Mizuta,K.; Nishimura,H.
EPI232159	MP	Japan	1989-Aug-03	EPI_ISL_66360	C/Yamagata/10/89		Import from public-domain	Matsuzaki,Y.; Sugawara,K.; Furuse,Y.; Shimotai,Y.; Hongo,S.; Oshitani,H.; Mizuta,K.; Nishimura,H.
EPI816593	NP	Japan	1989-Aug-03	EPI_ISL_66360	C/Yamagata/10/89		Import from public-domain	Matsuzaki,Y.; Sugawara,K.; Furuse,Y.; Shimotai,Y.; Hongo,S.; Oshitani,H.; Mizuta,K.; Nishimura,H.
EPI231571	NS	Japan	1989-Aug-03	EPI_ISL_66360	C/Yamagata/10/89		Import from public-domain	Matsuzaki,Y.; Sugawara,K.; Furuse,Y.; Shimotai,Y.; Hongo,S.; Oshitani,H.; Mizuta,K.; Nishimura,H.
EPI816494	P3	Japan	1989-Aug-03	EPI_ISL_66360	C/Yamagata/10/89		Import from public-domain	Matsuzaki,Y.; Sugawara,K.; Furuse,Y.; Shimotai,Y.; Hongo,S.; Oshitani,H.; Mizuta,K.; Nishimura,H.
EPI816412	PB1	Japan	1989-Aug-03	EPI_ISL_66360	C/Yamagata/10/89		Import from public-domain	Matsuzaki,Y.; Sugawara,K.; Furuse,Y.; Shimotai,Y.; Hongo,S.; Oshitani,H.; Mizuta,K.; Nishimura,H.
EPI813739	PB2	Japan	1989-Aug-03	EPI_ISL_66360	C/Yamagata/10/89		Import from public-domain	Matsuzaki,Y.; Sugawara,K.; Furuse,Y.; Shimotai,Y.; Hongo,S.; Oshitani,H.; Mizuta,K.; Nishimura,H.
EPI813676	HE	Japan	2008-Apr-30	EPI_ISL_230261	C/Yamagata/11/2008		Import from public-domain	Matsuzaki,Y.; Sugawara,K.; Furuse,Y.; Shimotai,Y.; Hongo,S.; Oshitani,H.; Mizuta,K.; Nishimura,H.
EPI816708	MP	Japan	2008-Apr-30	EPI_ISL_230261	C/Yamagata/11/2008		Import from public-domain	Matsuzaki,Y.; Sugawara,K.; Furuse,Y.; Shimotai,Y.; Hongo,S.; Oshitani,H.; Mizuta,K.; Nishimura,H.
EPI816641	NP	Japan	2008-Apr-30	EPI_ISL_230261	C/Yamagata/11/2008		Import from public-domain	Matsuzaki,Y.; Sugawara,K.; Furuse,Y.; Shimotai,Y.; Hongo,S.; Oshitani,H.; Mizuta,K.; Nishimura,H.
EPI816777	NS	Japan	2008-Apr-30	EPI_ISL_230261	C/Yamagata/11/2008		Import from public-domain	Matsuzaki,Y.; Sugawara,K.; Furuse,Y.; Shimotai,Y.; Hongo,S.; Oshitani,H.; Mizuta,K.; Nishimura,H.
EPI816541	P3	Japan	2008-Apr-30	EPI_ISL_230261	C/Yamagata/11/2008		Import from public-domain	Matsuzaki,Y.; Sugawara,K.; Furuse,Y.; Shimotai,Y.; Hongo,S.; Oshitani,H.; Mizuta,K.; Nishimura,H.
EPI816438	PB1	Japan	2008-Apr-30	EPI_ISL_230261	C/Yamagata/11/2008		Import from public-domain	Matsuzaki,Y.; Sugawara,K.; Furuse,Y.; Shimotai,Y.; Hongo,S.; Oshitani,H.; Mizuta,K.; Nishimura,H.
EPI813748	PB2	Japan	2008-Apr-30	EPI_ISL_230261	C/Yamagata/11/2008		Import from public-domain	Matsuzaki,Y.; Sugawara,K.; Furuse,Y.; Shimotai,Y.; Hongo,S.; Oshitani,H.; Mizuta,K.; Nishimura,H.
EPI813671	HE	Japan	2006-Aug-08	EPI_ISL_230256	C/Yamagata/13/2006		Import from public-domain	Matsuzaki,Y.; Sugawara,K.; Furuse,Y.; Shimotai,Y.; Hongo,S.; Oshitani,H.; Mizuta,K.; Nishimura,H.
EPI816702	MP	Japan	2006-Aug-08	EPI_ISL_230256	C/Yamagata/13/2006		Import from public-domain	Matsuzaki,Y.; Sugawara,K.; Furuse,Y.; Shimotai,Y.; Hongo,S.; Oshitani,H.; Mizuta,K.; Nishimura,H.
EPI816636	NP	Japan	2006-Aug-08	EPI_ISL_230256	C/Yamagata/13/2006		Import from public-domain	Matsuzaki,Y.; Sugawara,K.; Furuse,Y.; Shimotai,Y.; Hongo,S.; Oshitani,H.; Mizuta,K.; Nishimura,H.
EPI816771	NS	Japan	2006-Aug-08	EPI_ISL_230256	C/Yamagata/13/2006		Import from public-domain	Matsuzaki,Y.; Sugawara,K.; Furuse,Y.; Shimotai,Y.; Hongo,S.; Oshitani,H.; Mizuta,K.; Nishimura,H.
EPI816535	P3	Japan	2006-Aug-08	EPI_ISL_230256	C/Yamagata/13/2006		Import from public-domain	Matsuzaki,Y.; Sugawara,K.; Furuse,Y.; Shimotai,Y.; Hongo,S.; Oshitani,H.; Mizuta,K.; Nishimura,H.
EPI816433	PB1	Japan	2006-Aug-08	EPI_ISL_230256	C/Yamagata/13/2006		Import from public-domain	Matsuzaki,Y.; Sugawara,K.; Furuse,Y.; Shimotai,Y.; Hongo,S.; Oshitani,H.; Mizuta,K.; Nishimura,H.
EPI813743	PB2	Japan	2006-Aug-08	EPI_ISL_230256	C/Yamagata/13/2006		Import from public-domain	Matsuzaki,Y.; Sugawara,K.; Furuse,Y.; Shimotai,Y.; Hongo,S.; Oshitani,H.; Mizuta,K.; Nishimura,H.
EPI603680	HE	Japan	2014-May-14	EPI_ISL_182751	C/Yamagata/13/2014		Import from public-domain	Tanaka,S.; Aoki,Y.; Matoba,Y.; Yahagi,K.; Mizuta,K.; Itagaki,T.; Katsushima,F.; Katsushima,Y.; Matsuzaki,Y.; Sugawara,K.; Furuse,Y.; Shimotai,Y.; Hongo,S.; Oshitani,H.; Mizuta,K.; Nishimura,H.
EPI816725	MP	Japan	2014-May-14	EPI_ISL_182751	C/Yamagata/13/2014		Import from public-domain	Tanaka,S.; Aoki,Y.; Matoba,Y.; Yahagi,K.; Mizuta,K.; Itagaki,T.; Katsushima,F.; Katsushima,Y.; Matsuzaki,Y.; Sugawara,K.; Furuse,Y.; Shimotai,Y.; Hongo,S.; Oshitani,H.; Mizuta,K.; Nishimura,H.
EPI816657	NP	Japan	2014-May-14	EPI_ISL_182751	C/Yamagata/13/2014		Import from public-domain	Tanaka,S.; Aoki,Y.; Matoba,Y.; Yahagi,K.; Mizuta,K.; Itagaki,T.; Katsushima,F.; Katsushima,Y.; Matsuzaki,Y.; Sugawara,K.; Furuse,Y.; Shimotai,Y.; Hongo,S.; Oshitani,H.; Mizuta,K.; Nishimura,H.
EPI816794	NS	Japan	2014-May-14	EPI_ISL_182751	C/Yamagata/13/2014		Import from public-domain	Tanaka,S.; Aoki,Y.; Matoba,Y.; Yahagi,K.; Mizuta,K.; Itagaki,T.; Katsushima,F.; Katsushima,Y.; Matsuzaki,Y.; Sugawara,K.; Furuse,Y.; Shimotai,Y.; Hongo,S.; Oshitani,H.; Mizuta,K.; Nishimura,H.
EPI816558	P3	Japan	2014-May-14	EPI_ISL_182751	C/Yamagata/13/2014		Import from public-domain	Tanaka,S.; Aoki,Y.; Matoba,Y.; Yahagi,K.; Mizuta,K.; Itagaki,T.; Katsushima,F.; Katsushima,Y.; Matsuzaki,Y.; Sugawara,K.; Furuse,Y.; Shimotai,Y.; Hongo,S.; Oshitani,H.; Mizuta,K.; Nishimura,H.
EPI816475	PB1	Japan	2014-May-14	EPI_ISL_182751	C/Yamagata/13/2014		Import from public-domain	Tanaka,S.; Aoki,Y.; Matoba,Y.; Yahagi,K.; Mizuta,K.; Itagaki,T.; Katsushima,F.; Katsushima,Y.; Matsuzaki,Y.; Sugawara,K.; Furuse,Y.; Shimotai,Y.; Hongo,S.; Oshitani,H.; Mizuta,K.; Nishimura,H.
EPI813775	PB2	Japan	2014-May-14	EPI_ISL_182751	C/Yamagata/13/2014		Import from public-domain	Tanaka,S.; Aoki,Y.; Matoba,Y.; Yahagi,K.; Mizuta,K.; Itagaki,T.; Katsushima,F.; Katsushima,Y.; Matsuzaki,Y.; Sugawara,K.; Furuse,Y.; Shimotai,Y.; Hongo,S.; Oshitani,H.; Mizuta,K.; Nishimura,H.
EPI231920	HE	Japan	1998-Jun-08	EPI_ISL_66415	C/Yamagata/13/98		Import from public-domain	Matsuzaki,Y.; Sugawara,K.; Furuse,Y.; Shimotai,Y.; Hongo,S.; Oshitani,H.; Mizuta,K.; Nishimura,H.
EPI231918	MP	Japan	1998-Jun-08	EPI_ISL_66415	C/Yamagata/13/98		Import from public-domain	Matsuzaki,Y.; Sugawara,K.; Furuse,Y.; Shimotai,Y.; Hongo,S.; Oshitani,H.; Mizuta,K.; Nishimura,H.
EPI816610	NP	Japan	1998-Jun-08	EPI_ISL_66415	C/Yamagata/13/98		Import from public-domain	Matsuzaki,Y.; Sugawara,K.; Furuse,Y.; Shimotai,Y.; Hongo,S.; Oshitani,H.; Mizuta,K.; Nishimura,H.
EPI231919	NS	Japan	1998-Jun-08	EPI_ISL_66415	C/Yamagata/13/98		Import from public-domain	Matsuzaki,Y.; Sugawara,K.; Furuse,Y.; Shimotai,Y.; Hongo,S.; Oshitani,H.; Mizuta,K.; Nishimura,H.
EPI816505	P3	Japan	1998-Jun-08	EPI_ISL_66415	C/Yamagata/13/98		Import from public-domain	Matsuzaki,Y.; Sugawara,K.; Furuse,Y.; Shimotai,Y.; Hongo,S.; Oshitani,H.; Mizuta,K.; Nishimura,H.
EPI816422	PB1	Japan	1998-Jun-08	EPI_ISL_66415	C/Yamagata/13/98		Import from public-domain	Matsuzaki,Y.; Sugawara,K.; Furuse,Y.; Shimotai,Y.; Hongo,S.; Oshitani,H.; Mizuta,K.; Nishimura,H.
EPI813724	PB2	Japan	1998-Jun-08	EPI_ISL_66415	C/Yamagata/13/98		Import from public-domain	Matsuzaki,Y.; Sugawara,K.; Furuse,Y.; Shimotai,Y.; Hongo,S.; Oshitani,H.; Mizuta,K.; Nishimura,H.
EPI816684	MP	Japan	2004-May-07	EPI_ISL_230282	C/Yamagata/14/2004		Import from public-domain	Matsuzaki,Y.; Sugawara,K.; Furuse,Y.; Shimotai,Y.; Hongo,S.; Oshitani,H.; Mizuta,K.; Nishimura,H.
EPI816620	NP	Japan	2004-May-07	EPI_ISL_230282	C/Yamagata/14/2004		Import from public-domain	Matsuzaki,Y.; Sugawara,K.; Furuse,Y.; Shimotai,Y.; Hongo,S.; Oshitani,H.; Mizuta,K.; Nishimura,H.
EPI816753	NS	Japan	2004-May-07	EPI_ISL_230282	C/Yamagata/14/2004		Import from public-domain	Matsuzaki,Y.; Sugawara,K.; Furuse,Y.; Shimotai,Y.; Hongo,S.; Oshitani,H.; Mizuta,K.; Nishimura,H.
EPI816517	P3	Japan	2004-May-07	EPI_ISL_230282	C/Yamagata/14/2004		Import from public-domain	Matsuzaki,Y.; Sugawara,K.; Furuse,Y.; Shimotai,Y.; Hongo,S.; Oshitani,H.; Mizuta,K.; Nishimura,H.
EPI813803	PB1	Japan	2004-May-07	EPI_ISL_230282	C/Yamagata/14/2004		Import from public-domain	Matsuzaki,Y.; Sugawara,K.; Furuse,Y.; Shimotai,Y.; Hongo,S.; Oshitani,H.; Mizuta,K.; Nishimura,H.
EPI813706	PB2	Japan	2004-May-07	EPI_ISL_230282	C/Yamagata/14/2004		Import from public-domain	Matsuzaki,Y.; Sugawara,K.; Furuse,Y.; Shimotai,Y.; Hongo,S.; Oshitani,H.; Mizuta,K.; Nishimura,H.
EPI603688	HE	Japan	2006-Aug-28	EPI_ISL_182759	C/Yamagata/14/2006		Import from public-domain	Tanaka,S.; Aoki,Y.; Matoba,Y.; Yahagi,K.; Mizuta,K.; Itagaki,T.; Katsushima,F.; Katsushima,Y.; Matsuzaki,Y.; Sugawara,K.; Furuse,Y.; Shimotai,Y.; Hongo,S.; Oshitani,H.; Mizuta,K.; Nishimura,H.
EPI816703	MP	Japan	2006-Aug-28	EPI_ISL_182759	C/Yamagata/14/2006		Import from public-domain	Tanaka,S.; Aoki,Y.; Matoba,Y.; Yahagi,K.; Mizuta,K.; Itagaki,T.; Katsushima,F.; Katsushima,Y.; Matsuzaki,Y.; Sugawara,K.; Furuse,Y.; Shimotai,Y.; Hongo,S.; Oshitani,H.; Mizuta,K.; Nishimura,H.
EPI816637	NP	Japan	2006-Aug-28	EPI_ISL_182759	C/Yamagata/14/2006		Import from public-domain	Tanaka,S.; Aoki,Y.; Matoba,Y.; Yahagi,K.; Mizuta,K.; Itagaki,T.; Katsushima,F.; Katsushima,Y.; Matsuzaki,Y.; Sugawara,K.; Furuse,Y.; Shimotai,Y.; Hongo,S.; Oshitani,H.; Mizuta,K.; Nishimura,H.
EPI816772	NS	Japan	2006-Aug-28	EPI_ISL_182759	C/Yamagata/14/2006		Import from public-domain	Tanaka,S.; Aoki,Y.; Matoba,Y.; Yahagi,K.; Mizuta,K.; Itagaki,T.; Katsushima,F.; Katsushima,Y.; Matsuzaki,Y.; Sugawara,K.; Furuse,Y.; Shimotai,Y.; Hongo,S.; Oshitani,H.; Mizuta,K.; Nishimura,H.
EPI816536	P3	Japan	2006-Aug-28	EPI_ISL_182759	C/Yamagata/14/2006		Import from public-domain	Tanaka,S.; Aoki,Y.; Matoba,Y.; Yahagi,K.; Mizuta,K.; Itagaki,T.; Katsushima,F.; Katsushima,Y.; Matsuzaki,Y.; Sugawara,K.; Furuse,Y.; Shimotai,Y.; Hongo,S.; Oshitani,H.; Mizuta,K.; Nishimura,H.
EPI816472	PB1	Japan	2006-Aug-28	EPI_ISL_182759	C/Yamagata/14/2006		Import from public-domain	Tanaka,S.; Aoki,Y.; Matoba,Y.; Yahagi,K.; Mizuta,K.; Itagaki,T.; Katsushima,F.; Katsushima,Y.; Matsuzaki,Y.; Sugawara,K.; Furuse,Y.; Shimotai,Y.; Hongo,S.; Oshitani,H.; Mizuta,K.; Nishimura,H.
EPI813770	PB2	Japan	2006-Aug-28	EPI_ISL_182759	C/Yamagata/14/2006		Import from public-domain	Tanaka,S.; Aoki,Y.; Matoba,Y.; Yahagi,K.; Mizuta,K.; Itagaki,T.; Katsushima,F.; Katsushima,Y.; Matsuzaki,Y.; Sugawara,K.; Furuse,Y.; Shimotai,Y.; Hongo,S.; Oshitani,H.; Mizuta,K.; Nishimura,H.
EPI813684	HE	Japan	2012-Jun-01	EPI_ISL_230269	C/Yamagata/14/2012		Import from public-domain	Matsuzaki,Y.; Sugawara,K.; Furuse,Y.; Shimotai,Y.; Hongo,S.; Oshitani,H.; Mizuta,K.; Nishimura,H.
EPI816719	MP	Japan	2012-Jun-01	EPI_ISL_230269	C/Yamagata/14/2012		Import from public-domain	Matsuzaki,Y.; Sugawara,K.; Furuse,Y.; Shimotai,Y.; Hongo,S.; Oshitani,H.; Mizuta,K.; Nishimura,H.
EPI816651	NP	Japan	2012-Jun-01	EPI_ISL_230269	C/Yamagata/14/2012		Import from public-domain	Matsuzaki,Y.; Sugawara,K.; Furuse,Y.; Shimotai,Y.; Hongo,S.; Oshitani,H.; Mizuta,K.; Nishimura,H.
EPI816788	NS	Japan	2012-Jun-01	EPI_ISL_230269	C/Yamagata/14/2012		Import from public-domain	Matsuzaki,Y.; Sugawara,K.; Furuse,Y.; Shimotai,Y.; Hongo,S.; Oshitani,H.; Mizuta,K.; Nishimura,H.
EPI816552	P3	Japan	2012-Jun-01	EPI_ISL_230269	C/Yamagata/14/2012		Import from public-domain	Matsuzaki,Y.; Sugawara,K.; Furuse,Y.; Shimotai,Y.; Hongo,S.; Oshitani,H.; Mizuta,K.; Nishimura,H.
EPI816448	PB1	Japan	2012-Jun-01	EPI_ISL_230269	C/Yamagata/14/2012		Import from public-domain	Matsuzaki,Y.; Sugawara,K.; Furuse,Y.; Shimotai,Y.; Hongo,S.; Oshitani,H.; Mizuta,K.; Nishimura,H.
EPI813758	PB2	Japan	2012-Jun-01	EPI_ISL_230269	C/Yamagata/14/2012		Import from public-domain	Matsuzaki,Y.; Sugawara,K.; Furuse,Y.; Shimotai,Y.; Hongo,S.; Oshitani,H.; Mizuta,K.; Nishimura,H.
EPI603681	HE	Japan	2014-May-14	EPI_ISL_182752	C/Yamagata/14/2014		Import from public-domain	Tanaka,S.; Aoki,Y.; Matoba,Y.; Yahagi,K.; Mizuta,K.; Itagaki,T.; Katsushima,F.; Katsushima,Y.; Matsuzaki,Y.
EPI228320	HE	Japan	2004-May-11	EPI_ISL_65156	C/Yamagata/15/2004		Import from public-domain	Matsuzaki,Y.; Sugawara,K.; Furuse,Y.; Shimotai,Y.; Hongo,S.; Oshitani,H.; Mizuta,K.; Nishimura,H.
EPI816685	MP	Japan	2004-May-11	EPI_ISL_65156	C/Yamagata/15/2004		Import from public-domain	Matsuzaki,Y.; Sugawara,K.; Furuse,Y.; Shimotai,Y.; Hongo,S.; Oshitani,H.; Mizuta,K.; Nishimura,H.
EPI369797	NP	Japan	2004-May-11	EPI_ISL_118432	C/Yamagata/15/2004		Import from public-domain	Matsuzaki,Y.; Ikeda,T.; Abiko,C.; Aoki,Y.; Mizuta,K.; Shimotai,Y.; Sugawara,K.; Hongo,S.
EPI816754	NS	Japan	2004-May-11	EPI_ISL_65156	C/Yamagata/15/2004		Import from public-domain	Matsuzaki,Y.; Sugawara,K.; Furuse,Y.; Shimotai,Y.; Hongo,S.; Oshitani,H.; Mizuta,K.; Nishimura,H.
EPI816518	P3	Japan	2004-May-11	EPI_ISL_65156	C/Yamagata/15/2004		Import from public-domain	Matsuzaki,Y.; Sugawara,K.; Furuse,Y.; Shimotai,Y.; Hongo,S.; Oshitani,H.; Mizuta,K.; Nishimura,H.
EPI816426	PB1	Japan	2004-May-11	EPI_ISL_65156	C/Yamagata/15/2004		Import from public-domain	Matsuzaki,Y.; Sugawara,K.; Furuse,Y.; Shimotai,Y.; Hongo,S.; Oshitani,H.; Mizuta,K.; Nishimura,H.
EPI813737	PB2	Japan	2004-May-11	EPI_ISL_65156	C/Yamagata/15/2004		Import from public-domain	Matsuzaki,Y.; Sugawara,K.; Furuse,Y.; Shimotai,Y.; Hongo,S.; Oshitani,H.; Mizuta,K.; Nishimura,H.
EPI603682	HE	Japan	2014-May-16	EPI_ISL_182753	C/Yamagata/15/2014		Import from public-domain	Tanaka,S.; Aoki,Y.; Matoba,Y.; Yahagi,K.; Mizuta,K.; Itagaki,T.; Katsushima,F.; Katsushima,Y.; Matsuzaki,Y.
EPI813677	HE	Japan	2008-May-19	EPI_ISL_230262	C/Yamagata/16/2008		Import from public-domain	Matsuzaki,Y.; Sugawara,K.; Furuse,Y.; Shimotai,Y.; Hongo,S.; Oshitani,H.; Mizuta,K.; Nishimura,H.
EPI816709	MP	Japan	2008-May-19	EPI_ISL_230262	C/Yamagata/16/2008		Import from public-domain	Matsuzaki,Y.; Sugawara,K.; Furuse,Y.; Shimotai,Y.; Hongo,S.; Oshitani,H.; Mizuta,K.; Nishimura,H.
EPI816642	NP	Japan	2008-May-19	EPI_ISL_230262	C/Yamagata/16/2008		Import from public-domain	Matsuzaki,Y.; Sugawara,K.; Furuse,Y.; Shimotai,Y.; Hongo,S.; Oshitani,H.; Mizuta,K.; Nishimura,H.
EPI816778	NS	Japan	2008-May-19	EPI_ISL_230262	C/Yamagata/16/2008		Import from public-domain	Matsuzaki,Y.; Sugawara,K.; Furuse,Y.; Shimotai,Y.; Hongo,S.; Oshitani,H.; Mizuta,K.; Nishimura,H.
EPI816542	P3	Japan	2008-May-19	EPI_ISL_230262	C/Yamagata/16/2008		Import from public-domain	Matsuzaki,Y.; Sugawara,K.; Furuse,Y.; Shimotai,Y.; Hongo,S.; Oshitani,H.; Mizuta,K.; Nishimura,H.
EPI816439	PB1	Japan	2008-May-19	EPI_ISL_230262	C/Yamagata/16/2008		Import from public-domain	Matsuzaki,Y.; Sugawara,K.; Furuse,Y.; Shimotai,Y.; Hongo,S.; Oshitani,H.; Mizuta,K.; Nishimura,H.
EPI813749	PB2	Japan	2008-May-19	EPI_ISL_230262	C/Yamagata/16/2008		Import from public-domain	Matsuzaki,Y.; Sugawara,K.; Furuse,Y.; Shimotai,Y.; Hongo,S.; Oshitani,H.; Mizuta,K.; Nishimura,H.
EPI603683	HE	Japan	2014-May-21	EPI_ISL_182754	C/Yamagata/16/2014		Import from public-domain	Tanaka,S.; Aoki,Y.; Matoba,Y.; Yahagi,K.; Mizuta,K.; Itagaki,T.; Katsushima,F.; Katsushima,Y.; Matsuzaki,Y.; Sugawara,K.; Furuse,Y.; Shimotai,Y.; Hongo,S.; Oshitani,H.; Mizuta,K.; Nishimura,H.
EPI816726	MP	Japan	2014-May-21	EPI_ISL_182754	C/Yamagata/16/2014		Import from public-domain	Tanaka,S.; Aoki,Y.; Matoba,Y.; Yahagi,K.; Mizuta,K.; Itagaki,T.; Katsushima,F.; Katsushima,Y.; Matsuzaki,Y.; Sugawara,K.; Furuse,Y.; Shimotai,Y.; Hongo,S.; Oshitani,H.; Mizuta,K.; Nishimura,H.
EPI816658	NP	Japan	2014-May-21	EPI_ISL_182754	C/Yamagata/16/2014		Import from public-domain	Tanaka,S.; Aoki,Y.; Matoba,Y.; Yahagi,K.; Mizuta,K.; Itagaki,T.; Katsushima,F.; Katsushima,Y.; Matsuzaki,Y.; Sugawara,K.; Furuse,Y.; Shimotai,Y.; Hongo,S.; Oshitani,H.; Mizuta,K.; Nishimura,H.
EPI816795	NS	Japan	2014-May-21	EPI_ISL_182754	C/Yamagata/16/2014		Import from public-domain	Tanaka,S.; Aoki,Y.; Matoba,Y.; Yahagi,K.; Mizuta,K.; Itagaki,T.; Katsushima,F.; Katsushima,Y.; Matsuzaki,Y.; Sugawara,K.; Furuse,Y.; Shimotai,Y.; Hongo,S.; Oshitani,H.; Mizuta,K.; Nishimura,H.
EPI816559	P3	Japan	2014-May-21	EPI_ISL_182754	C/Yamagata/16/2014		Import from public-domain	Tanaka,S.; Aoki,Y.; Matoba,Y.; Yahagi,K.; Mizuta,K.; Itagaki,T.; Katsushima,F.; Katsushima,Y.; Matsuzaki,Y.; Sugawara,K.; Furuse,Y.; Shimotai,Y.; Hongo,S.; Oshitani,H.; Mizuta,K.; Nishimura,H.
EPI816476	PB1	Japan	2014-May-21	EPI_ISL_182754	C/Yamagata/16/2014		Import from public-domain	Tanaka,S.; Aoki,Y.; Matoba,Y.; Yahagi,K.; Mizuta,K.; Itagaki,T.; Katsushima,F.; Katsushima,Y.; Matsuzaki,Y.; Sugawara,K.; Furuse,Y.; Shimotai,Y.; Hongo,S.; Oshitani,H.; Mizuta,K.; Nishimura,H.
EPI813773	PB2	Japan	2014-May-21	EPI_ISL_182754	C/Yamagata/16/2014		Import from public-domain	Tanaka,S.; Aoki,Y.; Matoba,Y.; Yahagi,K.; Mizuta,K.; Itagaki,T.; Katsushima,F.; Katsushima,Y.; Matsuzaki,Y.; Sugawara,K.; Furuse,Y.; Shimotai,Y.; Hongo,S.; Oshitani,H.; Mizuta,K.; Nishimura,H.
EPI813658	HE	Japan	2004-May-14	EPI_ISL_230243	C/Yamagata/18/2004		Import from public-domain	Matsuzaki,Y.; Sugawara,K.; Furuse,Y.; Shimotai,Y.; Hongo,S.; Oshitani,H.; Mizuta,K.; Nishimura,H.
EPI816686	MP	Japan	2004-May-14	EPI_ISL_230243	C/Yamagata/18/2004		Import from public-domain	Matsuzaki,Y.; Sugawara,K.; Furuse,Y.; Shimotai,Y.; Hongo,S.; Oshitani,H.; Mizuta,K.; Nishimura,H.
EPI816621	NP	Japan	2004-May-14	EPI_ISL_230243	C/Yamagata/18/2004		Import from public-domain	Matsuzaki,Y.; Sugawara,K.; Furuse,Y.; Shimotai,Y.; Hongo,S.; Oshitani,H.; Mizuta,K.; Nishimura,H.

Segment ID	Segment	Country	Collection date	Isolate-ID	Isolate name	Originating Lab	Submitting Lab	Authors
EPI816755	NS	Japan	2004-May-14	EPI_ISL_230243	C/Yamagata/18/2004		Import from public-domain	Matsuzaki,Y.; Sugawara,K.; Furuse,Y.; Shimotai,Y.; Hongo,S.; Oshitani,H.; Mizuta,K.; Nishimura,H.
EPI816519	P3	Japan	2004-May-14	EPI_ISL_230243	C/Yamagata/18/2004		Import from public-domain	Matsuzaki,Y.; Sugawara,K.; Furuse,Y.; Shimotai,Y.; Hongo,S.; Oshitani,H.; Mizuta,K.; Nishimura,H.
EPI813804	PB1	Japan	2004-May-14	EPI_ISL_230243	C/Yamagata/18/2004		Import from public-domain	Matsuzaki,Y.; Sugawara,K.; Furuse,Y.; Shimotai,Y.; Hongo,S.; Oshitani,H.; Mizuta,K.; Nishimura,H.
EPI813707	PB2	Japan	2004-May-14	EPI_ISL_230243	C/Yamagata/18/2004		Import from public-domain	Matsuzaki,Y.; Sugawara,K.; Furuse,Y.; Shimotai,Y.; Hongo,S.; Oshitani,H.; Mizuta,K.; Nishimura,H.
EPI603689	HE	Japan	2008-Jun-25	EPI_ISL_182760	C/Yamagata/18/2008		Import from public-domain	Tanaka,S.; Aoki,Y.; Matoba,Y.; Yahagi,K.; Mizuta,K.; Itagaki,T.; Katsushima,F.; Katsushima,Y.; Matsuzaki,Y.; Sugawara,K.; Furuse,Y.; Shimotai,Y.; Hongo,S.; Oshitani,H.; Mizuta,K.; Nishimura,H.
EPI816710	MP	Japan	2008-Jun-25	EPI_ISL_182760	C/Yamagata/18/2008		Import from public-domain	Tanaka,S.; Aoki,Y.; Matoba,Y.; Yahagi,K.; Mizuta,K.; Itagaki,T.; Katsushima,F.; Katsushima,Y.; Matsuzaki,Y.; Sugawara,K.; Furuse,Y.; Shimotai,Y.; Hongo,S.; Oshitani,H.; Mizuta,K.; Nishimura,H.
EPI816643	NP	Japan	2008-Jun-25	EPI_ISL_182760	C/Yamagata/18/2008		Import from public-domain	Tanaka,S.; Aoki,Y.; Matoba,Y.; Yahagi,K.; Mizuta,K.; Itagaki,T.; Katsushima,F.; Katsushima,Y.; Matsuzaki,Y.; Sugawara,K.; Furuse,Y.; Shimotai,Y.; Hongo,S.; Oshitani,H.; Mizuta,K.; Nishimura,H.
EPI816779	NS	Japan	2008-Jun-25	EPI_ISL_182760	C/Yamagata/18/2008		Import from public-domain	Tanaka,S.; Aoki,Y.; Matoba,Y.; Yahagi,K.; Mizuta,K.; Itagaki,T.; Katsushima,F.; Katsushima,Y.; Matsuzaki,Y.; Sugawara,K.; Furuse,Y.; Shimotai,Y.; Hongo,S.; Oshitani,H.; Mizuta,K.; Nishimura,H.
EPI816543	P3	Japan	2008-Jun-25	EPI_ISL_182760	C/Yamagata/18/2008		Import from public-domain	Tanaka,S.; Aoki,Y.; Matoba,Y.; Yahagi,K.; Mizuta,K.; Itagaki,T.; Katsushima,F.; Katsushima,Y.; Matsuzaki,Y.; Sugawara,K.; Furuse,Y.; Shimotai,Y.; Hongo,S.; Oshitani,H.; Mizuta,K.; Nishimura,H.
EPI816473	PB1	Japan	2008-Jun-25	EPI_ISL_182760	C/Yamagata/18/2008		Import from public-domain	Tanaka,S.; Aoki,Y.; Matoba,Y.; Yahagi,K.; Mizuta,K.; Itagaki,T.; Katsushima,F.; Katsushima,Y.; Matsuzaki,Y.; Sugawara,K.; Furuse,Y.; Shimotai,Y.; Hongo,S.; Oshitani,H.; Mizuta,K.; Nishimura,H.
EPI813771	PB2	Japan	2008-Jun-25	EPI_ISL_182760	C/Yamagata/18/2008		Import from public-domain	Tanaka,S.; Aoki,Y.; Matoba,Y.; Yahagi,K.; Mizuta,K.; Itagaki,T.; Katsushima,F.; Katsushima,Y.; Matsuzaki,Y.; Sugawara,K.; Furuse,Y.; Shimotai,Y.; Hongo,S.; Oshitani,H.; Mizuta,K.; Nishimura,H.
EPI231922	HE	Japan	2000-Jan-01	EPI_ISL_66416	C/Yamagata/2/2000		Import from public-domain	Matsuzaki,Y.
EPI231926	MP	Japan	2000-Jan-01	EPI_ISL_66416	C/Yamagata/2/2000		Import from public-domain	Matsuzaki,Y.
EPI231921	NS	Japan	2000-Jan-01	EPI_ISL_66416	C/Yamagata/2/2000		Import from public-domain	Matsuzaki,Y.
EPI813663	HE	Japan	2005-Jul-05	EPI_ISL_230248	C/Yamagata/2/2005		Import from public-domain	Matsuzaki,Y.; Sugawara,K.; Furuse,Y.; Shimotai,Y.; Hongo,S.; Oshitani,H.; Mizuta,K.; Nishimura,H.
EPI816694	MP	Japan	2005-Jul-05	EPI_ISL_230248	C/Yamagata/2/2005		Import from public-domain	Matsuzaki,Y.; Sugawara,K.; Furuse,Y.; Shimotai,Y.; Hongo,S.; Oshitani,H.; Mizuta,K.; Nishimura,H.
EPI816629	NP	Japan	2005-Jul-05	EPI_ISL_230248	C/Yamagata/2/2005		Import from public-domain	Matsuzaki,Y.; Sugawara,K.; Furuse,Y.; Shimotai,Y.; Hongo,S.; Oshitani,H.; Mizuta,K.; Nishimura,H.
EPI816763	NS	Japan	2005-Jul-05	EPI_ISL_230248	C/Yamagata/2/2005		Import from public-domain	Matsuzaki,Y.; Sugawara,K.; Furuse,Y.; Shimotai,Y.; Hongo,S.; Oshitani,H.; Mizuta,K.; Nishimura,H.
EPI816527	P3	Japan	2005-Jul-05	EPI_ISL_230248	C/Yamagata/2/2005		Import from public-domain	Matsuzaki,Y.; Sugawara,K.; Furuse,Y.; Shimotai,Y.; Hongo,S.; Oshitani,H.; Mizuta,K.; Nishimura,H.
EPI813809	PB1	Japan	2005-Jul-05	EPI_ISL_230248	C/Yamagata/2/2005		Import from public-domain	Matsuzaki,Y.; Sugawara,K.; Furuse,Y.; Shimotai,Y.; Hongo,S.; Oshitani,H.; Mizuta,K.; Nishimura,H.
EPI813712	PB2	Japan	2005-Jul-05	EPI_ISL_230248	C/Yamagata/2/2005		Import from public-domain	Matsuzaki,Y.; Sugawara,K.; Furuse,Y.; Shimotai,Y.; Hongo,S.; Oshitani,H.; Mizuta,K.; Nishimura,H.
EPI816712	MP	Japan	2010-Feb-09	EPI_ISL_230283	C/Yamagata/2/2010		Import from public-domain	Matsuzaki,Y.; Sugawara,K.; Furuse,Y.; Shimotai,Y.; Hongo,S.; Oshitani,H.; Mizuta,K.; Nishimura,H.
EPI369788	NP	Japan	2010-Feb-09	EPI_ISL_118424	C/Yamagata/2/2010		Import from public-domain	Matsuzaki,Y.; Ikeda,T.; Abiko,C.; Aoki,Y.; Mizuta,K.; Shimotai,Y.; Sugawara,K.; Hongo,S.
EPI816781	NS	Japan	2010-Feb-09	EPI_ISL_230283	C/Yamagata/2/2010		Import from public-domain	Matsuzaki,Y.; Sugawara,K.; Furuse,Y.; Shimotai,Y.; Hongo,S.; Oshitani,H.; Mizuta,K.; Nishimura,H.
EPI816545	P3	Japan	2010-Feb-09	EPI_ISL_230283	C/Yamagata/2/2010		Import from public-domain	Matsuzaki,Y.; Sugawara,K.; Furuse,Y.; Shimotai,Y.; Hongo,S.; Oshitani,H.; Mizuta,K.; Nishimura,H.
EPI816441	PB1	Japan	2010-Feb-09	EPI_ISL_230283	C/Yamagata/2/2010		Import from public-domain	Matsuzaki,Y.; Sugawara,K.; Furuse,Y.; Shimotai,Y.; Hongo,S.; Oshitani,H.; Mizuta,K.; Nishimura,H.
EPI813751	PB2	Japan	2010-Feb-09	EPI_ISL_230283	C/Yamagata/2/2010		Import from public-domain	Matsuzaki,Y.; Sugawara,K.; Furuse,Y.; Shimotai,Y.; Hongo,S.; Oshitani,H.; Mizuta,K.; Nishimura,H.
EPI231934	HE	Japan	1998-Jan-01	EPI_ISL_66417	C/Yamagata/2/98		Import from public-domain	Matsuzaki,Y.
EPI231930	MP	Japan	1998-Jan-01	EPI_ISL_66417	C/Yamagata/2/98		Import from public-domain	Matsuzaki,Y.
EPI231929	NS	Japan	1998-Jan-01	EPI_ISL_66417	C/Yamagata/2/98		Import from public-domain	Matsuzaki,Y.
EPI231935	HE	Japan	1999-Jan-01	EPI_ISL_66418	C/Yamagata/2/99		Import from public-domain	Matsuzaki,Y.
EPI231939	MP	Japan	1999-Jan-01	EPI_ISL_66418	C/Yamagata/2/99		Import from public-domain	Matsuzaki,Y.
EPI231941	NS	Japan	1999-Jan-01	EPI_ISL_66418	C/Yamagata/2/99		Import from public-domain	Matsuzaki,Y.
EPI228317	HE	Japan	2004-Jan-01	EPI_ISL_65153	C/Yamagata/20/2004		Import from public-domain	Matsuzaki,Y.
EPI813672	HE	Japan	2006-Nov-24	EPI_ISL_230257	C/Yamagata/20/2006		Import from public-domain	Matsuzaki,Y.; Sugawara,K.; Furuse,Y.; Shimotai,Y.; Hongo,S.; Oshitani,H.; Mizuta,K.; Nishimura,H.
EPI816704	MP	Japan	2006-Nov-24	EPI_ISL_230257	C/Yamagata/20/2006		Import from public-domain	Matsuzaki,Y.; Sugawara,K.; Furuse,Y.; Shimotai,Y.; Hongo,S.; Oshitani,H.; Mizuta,K.; Nishimura,H.
EPI816638	NP	Japan	2006-Nov-24	EPI_ISL_230257	C/Yamagata/20/2006		Import from public-domain	Matsuzaki,Y.; Sugawara,K.; Furuse,Y.; Shimotai,Y.; Hongo,S.; Oshitani,H.; Mizuta,K.; Nishimura,H.
EPI816773	NS	Japan	2006-Nov-24	EPI_ISL_230257	C/Yamagata/20/2006		Import from public-domain	Matsuzaki,Y.; Sugawara,K.; Furuse,Y.; Shimotai,Y.; Hongo,S.; Oshitani,H.; Mizuta,K.; Nishimura,H.
EPI816537	P3	Japan	2006-Nov-24	EPI_ISL_230257	C/Yamagata/20/2006		Import from public-domain	Matsuzaki,Y.; Sugawara,K.; Furuse,Y.; Shimotai,Y.; Hongo,S.; Oshitani,H.; Mizuta,K.; Nishimura,H.
EPI816434	PB1	Japan	2006-Nov-24	EPI_ISL_230257	C/Yamagata/20/2006		Import from public-domain	Matsuzaki,Y.; Sugawara,K.; Furuse,Y.; Shimotai,Y.; Hongo,S.; Oshitani,H.; Mizuta,K.; Nishimura,H.
EPI813744	PB2	Japan	2006-Nov-24	EPI_ISL_230257	C/Yamagata/20/2006		Import from public-domain	Matsuzaki,Y.; Sugawara,K.; Furuse,Y.; Shimotai,Y.; Hongo,S.; Oshitani,H.; Mizuta,K.; Nishimura,H.
EPI231943	HE	Japan	1996-Jan-01	EPI_ISL_66419	C/Yamagata/20/96		Import from public-domain	Matsuzaki,Y.
EPI231945	MP	Japan	1996-Jan-01	EPI_ISL_66419	C/Yamagata/20/96		Import from public-domain	Matsuzaki,Y.
EPI231946	NS	Japan	1996-Jan-01	EPI_ISL_66419	C/Yamagata/20/96		Import from public-domain	Matsuzaki,Y.
EPI228318	HE	Japan	2004-Jan-01	EPI_ISL_65154	C/Yamagata/21/2004		Import from public-domain	Matsuzaki,Y.
EPI813659	HE	Japan	2004-May-28	EPI_ISL_230244	C/Yamagata/26/2004		Import from public-domain	Matsuzaki,Y.; Sugawara,K.; Furuse,Y.; Shimotai,Y.; Hongo,S.; Oshitani,H.; Mizuta,K.; Nishimura,H.
EPI816687	MP	Japan	2004-May-28	EPI_ISL_230244	C/Yamagata/26/2004		Import from public-domain	Matsuzaki,Y.; Sugawara,K.; Furuse,Y.; Shimotai,Y.; Hongo,S.; Oshitani,H.; Mizuta,K.; Nishimura,H.
EPI816622	NP	Japan	2004-May-28	EPI_ISL_230244	C/Yamagata/26/2004		Import from public-domain	Matsuzaki,Y.; Sugawara,K.; Furuse,Y.; Shimotai,Y.; Hongo,S.; Oshitani,H.; Mizuta,K.; Nishimura,H.
EPI816756	NS	Japan	2004-May-28	EPI_ISL_230244	C/Yamagata/26/2004		Import from public-domain	Matsuzaki,Y.; Sugawara,K.; Furuse,Y.; Shimotai,Y.; Hongo,S.; Oshitani,H.; Mizuta,K.; Nishimura,H.
EPI816520	P3	Japan	2004-May-28	EPI_ISL_230244	C/Yamagata/26/2004		Import from public-domain	Matsuzaki,Y.; Sugawara,K.; Furuse,Y.; Shimotai,Y.; Hongo,S.; Oshitani,H.; Mizuta,K.; Nishimura,H.
EPI813805	PB1	Japan	2004-May-28	EPI_ISL_230244	C/Yamagata/26/2004		Import from public-domain	Matsuzaki,Y.; Sugawara,K.; Furuse,Y.; Shimotai,Y.; Hongo,S.; Oshitani,H.; Mizuta,K.; Nishimura,H.
EPI813708	PB2	Japan	2004-May-28	EPI_ISL_230244	C/Yamagata/26/2004		Import from public-domain	Matsuzaki,Y.; Sugawara,K.; Furuse,Y.; Shimotai,Y.; Hongo,S.; Oshitani,H.; Mizuta,K.; Nishimura,H.
EPI231538	HE	Japan	1981-Mar-18	EPI_ISL_66337	C/Yamagata/26/81		Import from public-domain	Matsuzaki,Y.; Sugawara,K.; Furuse,Y.; Shimotai,Y.; Hongo,S.; Oshitani,H.; Mizuta,K.; Nishimura,H.
EPI232166	MP	Japan	1981-Mar-18	EPI_ISL_66337	C/Yamagata/26/81		Import from public-domain	Matsuzaki,Y.; Sugawara,K.; Furuse,Y.; Shimotai,Y.; Hongo,S.; Oshitani,H.; Mizuta,K.; Nishimura,H.
EPI816586	NP	Japan	1981-Mar-18	EPI_ISL_66337	C/Yamagata/26/81		Import from public-domain	Matsuzaki,Y.; Sugawara,K.; Furuse,Y.; Shimotai,Y.; Hongo,S.; Oshitani,H.; Mizuta,K.; Nishimura,H.
EPI231543	NS	Japan	1981-Mar-18	EPI_ISL_66337	C/Yamagata/26/81		Import from public-domain	Matsuzaki,Y.; Sugawara,K.; Furuse,Y.; Shimotai,Y.; Hongo,S.; Oshitani,H.; Mizuta,K.; Nishimura,H.
EPI232169	P3	Japan	1981-Mar-18	EPI_ISL_66337	C/Yamagata/26/81		Import from public-domain	Matsuzaki,Y.; Sugawara,K.; Furuse,Y.; Shimotai,Y.; Hongo,S.; Oshitani,H.; Mizuta,K.; Nishimura,H.
EPI816487	P3	Japan	1981-Mar-18	EPI_ISL_66337	C/Yamagata/26/81		Import from public-domain	Matsuzaki,Y.; Sugawara,K.; Furuse,Y.; Shimotai,Y.; Hongo,S.; Oshitani,H.; Mizuta,K.; Nishimura,H.
EPI815441	PB1	Japan	1981-Mar-18	EPI_ISL_66337	C/Yamagata/26/81		Import from public-domain	Matsuzaki,Y.; Sugawara,K.; Furuse,Y.; Shimotai,Y.; Hongo,S.; Oshitani,H.; Mizuta,K.; Nishimura,H.
EPI813720	PB2	Japan	1981-Mar-18	EPI_ISL_66337	C/Yamagata/26/81		Import from public-domain	Matsuzaki,Y.; Sugawara,K.; Furuse,Y.; Shimotai,Y.; Hongo,S.; Oshitani,H.; Mizuta,K.; Nishimura,H.
EPI228321	HE	Japan	2004-May-24	EPI_ISL_65157	C/Yamagata/27/2004		Import from public-domain	Matsuzaki,Y.; Sugawara,K.; Furuse,Y.; Shimotai,Y.; Hongo,S.; Oshitani,H.; Mizuta,K.; Nishimura,H.
EPI816688	MP	Japan	2004-May-24	EPI_ISL_65157	C/Yamagata/27/2004		Import from public-domain	Matsuzaki,Y.; Sugawara,K.; Furuse,Y.; Shimotai,Y.; Hongo,S.; Oshitani,H.; Mizuta,K.; Nishimura,H.
EPI816623	NP	Japan	2004-May-24	EPI_ISL_65157	C/Yamagata/27/2004		Import from public-domain	Matsuzaki,Y.; Sugawara,K.; Furuse,Y.; Shimotai,Y.; Hongo,S.; Oshitani,H.; Mizuta,K.; Nishimura,H.
EPI816757	NS	Japan	2004-May-24	EPI_ISL_65157	C/Yamagata/27/2004		Import from public-domain	Matsuzaki,Y.; Sugawara,K.; Furuse,Y.; Shimotai,Y.; Hongo,S.; Oshitani,H.; Mizuta,K.; Nishimura,H.
EPI816521	P3	Japan	2004-May-24	EPI_ISL_65157	C/Yamagata/27/2004		Import from public-domain	Matsuzaki,Y.; Sugawara,K.; Furuse,Y.; Shimotai,Y.; Hongo,S.; Oshitani,H.; Mizuta,K.; Nishimura,H.
EPI816428	PB1	Japan	2004-May-24	EPI_ISL_65157	C/Yamagata/27/2004		Import from public-domain	Matsuzaki,Y.; Sugawara,K.; Furuse,Y.; Shimotai,Y.; Hongo,S.; Oshitani,H.; Mizuta,K.; Nishimura,H.
EPI813738	PB2	Japan	2004-May-24	EPI_ISL_65157	C/Yamagata/27/2004		Import from public-domain	Matsuzaki,Y.; Sugawara,K.; Furuse,Y.; Shimotai,Y.; Hongo,S.; Oshitani,H.; Mizuta,K.; Nishimura,H.
EPI813685	HE	Japan	2012-Jul-12	EPI_ISL_230270	C/Yamagata/28/2012		Import from public-domain	Matsuzaki,Y.; Sugawara,K.; Furuse,Y.; Shimotai,Y.; Hongo,S.; Oshitani,H.; Mizuta,K.; Nishimura,H.
EPI816720	MP	Japan	2012-Jul-12	EPI_ISL_230270	C/Yamagata/28/2012		Import from public-domain	Matsuzaki,Y.; Sugawara,K.; Furuse,Y.; Shimotai,Y.; Hongo,S.; Oshitani,H.; Mizuta,K.; Nishimura,H.
EPI816652	NP	Japan	2012-Jul-12	EPI_ISL_230270	C/Yamagata/28/2012		Import from public-domain	Matsuzaki,Y.; Sugawara,K.; Furuse,Y.; Shimotai,Y.; Hongo,S.; Oshitani,H.; Mizuta,K.; Nishimura,H.
EPI816789	NS	Japan	2012-Jul-12	EPI_ISL_230270	C/Yamagata/28/2012		Import from public-domain	Matsuzaki,Y.; Sugawara,K.; Furuse,Y.; Shimotai,Y.; Hongo,S.; Oshitani,H.; Mizuta,K.; Nishimura,H.
EPI816553	P3	Japan	2012-Jul-12	EPI_ISL_230270	C/Yamagata/28/2012		Import from public-domain	Matsuzaki,Y.; Sugawara,K.; Furuse,Y.; Shimotai,Y.; Hongo,S.; Oshitani,H.; Mizuta,K.; Nishimura,H.
EPI816449	PB1	Japan	2012-Jul-12	EPI_ISL_230270	C/Yamagata/28/2012		Import from public-domain	Matsuzaki,Y.; Sugawara,K.; Furuse,Y.; Shimotai,Y.; Hongo,S.; Oshitani,H.; Mizuta,K.; Nishimura,H.
EPI813759	PB2	Japan	2012-Jul-12	EPI_ISL_230270	C/Yamagata/28/2012		Import from public-domain	Matsuzaki,Y.; Sugawara,K.; Furuse,Y.; Shimotai,Y.; Hongo,S.; Oshitani,H.; Mizuta,K.; Nishimura,H.
EPI816721	MP	Japan	2012-Jul-30	EPI_ISL_230285	C/Yamagata/29/2012		Import from public-domain	Matsuzaki,Y.; Sugawara,K.; Furuse,Y.; Shimotai,Y.; Hongo,S.; Oshitani,H.; Mizuta,K.; Nishimura,H.
EPI816653	NP	Japan	2012-Jul-30	EPI_ISL_230285	C/Yamagata/29/2012		Import from public-domain	Matsuzaki,Y.; Sugawara,K.; Furuse,Y.; Shimotai,Y.; Hongo,S.; Oshitani,H.; Mizuta,K.; Nishimura,H.
EPI816790	NS	Japan	2012-Jul-30	EPI_ISL_230285	C/Yamagata/29/2012		Import from public-domain	Matsuzaki,Y.; Sugawara,K.; Furuse,Y.; Shimotai,Y.; Hongo,S.; Oshitani,H.; Mizuta,K.; Nishimura,H.
EPI816554	P3	Japan	2012-Jul-30	EPI_ISL_230285	C/Yamagata/29/2012		Import from public-domain	Matsuzaki,Y.; Sugawara,K.; Furuse,Y.; Shimotai,Y.; Hongo,S.; Oshitani,H.; Mizuta,K.; Nishimura,H.
EPI816450	PB1	Japan	2012-Jul-30	EPI_ISL_230285	C/Yamagata/29/2012		Import from public-domain	Matsuzaki,Y.; Sugawara,K.; Furuse,Y.; Shimotai,Y.; Hongo,S.; Oshitani,H.; Mizuta,K.; Nishimura,H.
EPI813760	PB2	Japan	2012-Jul-30	EPI_ISL_230285	C/Yamagata/29/2012		Import from public-domain	Matsuzaki,Y.; Sugawara,K.; Furuse,Y.; Shimotai,Y.; Hongo,S.; Oshitani,H.; Mizuta,K.; Nishimura,H.
EPI231950	HE	Japan	2000-Feb-02	EPI_ISL_66420	C/Yamagata/3/2000		Import from public-domain	Matsuzaki,Y.; Sugawara,K.; Furuse,Y.; Shimotai,Y.; Hongo,S.; Oshitani,H.; Mizuta,K.; Nishimura,H.
EPI231953	MP	Japan	2000-Feb-02	EPI_ISL_66420	C/Yamagata/3/2000		Import from public-domain	Matsuzaki,Y.; Sugawara,K.; Furuse,Y.; Shimotai,Y.; Hongo,S.; Oshitani,H.; Mizuta,K.; Nishimura,H.
EPI369795	NP	Japan	2000-Feb-02	EPI_ISL_118430	C/Yamagata/3/2000		Import from public-domain	Matsuzaki,Y.; Ikeda,T.; Abiko,C.; Aoki,Y.; Mizuta,K.; Shimotai,Y.; Sugawara,K.; Hongo,S.
EPI231949	NS	Japan	2000-Feb-02	EPI_ISL_66420	C/Yamagata/3/2000		Import from public-domain	Matsuzaki,Y.; Sugawara,K.; Furuse,Y.; Shimotai,Y.; Hongo,S.; Oshitani,H.; Mizuta,K.; Nishimura,H.
EPI816507	P3	Japan	2000-Feb-02	EPI_ISL_66420	C/Yamagata/3/2000		Import from public-domain	Matsuzaki,Y.; Sugawara,K.; Furuse,Y.; Shimotai,Y.; Hongo,S.; Oshitani,H.; Mizuta,K.; Nishimura,H.
EPI816424	PB1	Japan	2000-Feb-02	EPI_ISL_66420	C/Yamagata/3/2000		Import from public-domain	Matsuzaki,Y.; Sugawara,K.; Furuse,Y.; Shimotai,Y.; Hongo,S.; Oshitani,H.; Mizuta,K.; Nishimura,H.
EPI813734	PB2	Japan	2000-Feb-02	EPI_ISL_66420	C/Yamagata/3/2000		Import from public-domain	Matsuzaki,Y.; Sugawara,K.; Furuse,Y.; Shimotai,Y.; Hongo,S.; Oshitani,H.; Mizuta,K.; Nishimura,H.
EPI228319	HE	Japan	2004-Apr-16	EPI_ISL_65155	C/Yamagata/3/2004		Import from public-domain	Matsuzaki,Y.; Sugawara,K.; Furuse,Y.; Shimotai,Y.; Hongo,S.; Oshitani,H.; Mizuta,K.; Nishimura,H.
EPI816683	MP	Japan	2004-Apr-16	EPI_ISL_65155	C/Yamagata/3/2004		Import from public-domain	Matsuzaki,Y.; Sugawara,K.; Furuse,Y.; Shimotai,Y.; Hongo,S.; Oshitani,H.; Mizuta,K.; Nishimura,H.
EPI816619	NP	Japan	2004-Apr-16	EPI_ISL_65155	C/Yamagata/3/2004		Import from public-domain	Matsuzaki,Y.; Sugawara,K.; Furuse,Y.; Shimotai,Y.; Hongo,S.; Oshitani,H.; Mizuta,K.; Nishimura,H.
EPI816752	NS	Japan	2004-Apr-16	EPI_ISL_65155	C/Yamagata/3/2004		Import from public-domain	Matsuzaki,Y.; Sugawara,K.; Furuse,Y.; Shimotai,Y.; Hongo,S.; Oshitani,H.; Mizuta,K.; Nishimura,H.
EPI816516	P3	Japan	2004-Apr-16	EPI_ISL_65155	C/Yamagata/3/2004		Import from public-domain	Matsuzaki,Y.; Sugawara,K.; Furuse,Y.; Shimotai,Y.; Hongo,S.; Oshitani,H.; Mizuta,K.; Nishimura,H.
EPI816427	PB1	Japan	2004-Apr-16	EPI_ISL_65155	C/Yamagata/3/2004		Import from public-domain	Matsuzaki,Y.; Sugawara,K.; Furuse,Y.; Shimotai,Y.; Hongo,S.; Oshitani,H.; Mizuta,K.; Nishimura,H.
EPI813736	PB2	Japan	2004-Apr-16	EPI_ISL_65155	C/Yamagata/3/2004		Import from public-domain	Matsuzaki,Y.; Sugawara,K.; Furuse,Y.; Shimotai,Y.; Hongo,S.; Oshitani,H.; Mizuta,K.; Nishimura,H.
EPI813664	HE	Japan	2005-Sep-07	EPI_ISL_230249	C/Yamagata/3/2005		Import from public-domain	Matsuzaki,Y.; Sugawara,K.; Furuse,Y.; Shimotai,Y.; Hongo,S.; Oshitani,H.; Mizuta,K.; Nishimura,H.
EPI816695	MP	Japan	2005-Sep-07	EPI_ISL_230249	C/Yamagata/3/2005		Import from public-domain	Matsuzaki,Y.; Sugawara,K.; Furuse,Y.; Shimotai,Y.; Hongo,S.; Oshitani,H.; Mizuta,K.; Nishimura,H.
EPI369792	NP	Japan	2005-Sep-07	EPI_ISL_118427	C/Yamagata/3/2005		Import from public-domain	Matsuzaki,Y.; Ikeda,T.; Abiko,C.; Aoki,Y.; Mizuta,K.; Shimotai,Y.; Sugawara,K.; Hongo,S.
EPI816764	NS	Japan	2005-Sep-07	EPI_ISL_230249	C/Yamagata/3/2005		Import from public-domain	Matsuzaki,Y.; Sugawara,K.; Furuse,Y.; Shimotai,Y.; Hongo,S.; Oshitani,H.; Mizuta,K.; Nishimura,H.
EPI816528	P3	Japan	2005-Sep-07	EPI_ISL_230249	C/Yamagata/3/2005		Import from public-domain	Matsuzaki,Y.; Sugawara,K.; Furuse,Y.; Shimotai,Y.; Hongo,S.; Oshitani,H.; Mizuta,K.; Nishimura,H.
EPI813810	PB1	Japan	2005-Sep-07	EPI_ISL_230249	C/Yamagata/3/2005		Import from public-domain	Matsuzaki,Y.; Sugawara,K.; Furuse,Y.; Shimotai,Y.; Hongo,S.; Oshitani,H.; Mizuta,K.; Nishimura,H.

Segment ID	Segment	Country	Collection date	Isolate-ID	Isolate name	Originating Lab	Submitting Lab	Authors
EPI813713	PB2	Japan	2005-Sep-07	EPI_ISL_230249	C/Yamagata/3/2005		Import from public-domain	Matsuzaki,Y.; Sugawara,K.; Furuse,Y.; Shimotai,Y.; Hongo,S.; Oshitani,H.; Mizuta,K.; Nishimura,H.
EPI813675	HE	Japan	2008-Mar-26	EPI_ISL_230260	C/Yamagata/3/2008		Import from public-domain	Matsuzaki,Y.; Sugawara,K.; Furuse,Y.; Shimotai,Y.; Hongo,S.; Oshitani,H.; Mizuta,K.; Nishimura,H.
EPI816707	MP	Japan	2008-Mar-26	EPI_ISL_230260	C/Yamagata/3/2008		Import from public-domain	Matsuzaki,Y.; Sugawara,K.; Furuse,Y.; Shimotai,Y.; Hongo,S.; Oshitani,H.; Mizuta,K.; Nishimura,H.
EPI816640	NP	Japan	2008-Mar-26	EPI_ISL_230260	C/Yamagata/3/2008		Import from public-domain	Matsuzaki,Y.; Sugawara,K.; Furuse,Y.; Shimotai,Y.; Hongo,S.; Oshitani,H.; Mizuta,K.; Nishimura,H.
EPI816776	NS	Japan	2008-Mar-26	EPI_ISL_230260	C/Yamagata/3/2008		Import from public-domain	Matsuzaki,Y.; Sugawara,K.; Furuse,Y.; Shimotai,Y.; Hongo,S.; Oshitani,H.; Mizuta,K.; Nishimura,H.
EPI816540	P3	Japan	2008-Mar-26	EPI_ISL_230260	C/Yamagata/3/2008		Import from public-domain	Matsuzaki,Y.; Sugawara,K.; Furuse,Y.; Shimotai,Y.; Hongo,S.; Oshitani,H.; Mizuta,K.; Nishimura,H.
EPI816437	PB1	Japan	2008-Mar-26	EPI_ISL_230260	C/Yamagata/3/2008		Import from public-domain	Matsuzaki,Y.; Sugawara,K.; Furuse,Y.; Shimotai,Y.; Hongo,S.; Oshitani,H.; Mizuta,K.; Nishimura,H.
EPI813747	PB2	Japan	2008-Mar-26	EPI_ISL_230260	C/Yamagata/3/2008		Import from public-domain	Matsuzaki,Y.; Sugawara,K.; Furuse,Y.; Shimotai,Y.; Hongo,S.; Oshitani,H.; Mizuta,K.; Nishimura,H.
EPI813682	HE	Japan	2012-Feb-07	EPI_ISL_230267	C/Yamagata/3/2012		Import from public-domain	Matsuzaki,Y.; Sugawara,K.; Furuse,Y.; Shimotai,Y.; Hongo,S.; Oshitani,H.; Mizuta,K.; Nishimura,H.
EPI816716	MP	Japan	2012-Feb-07	EPI_ISL_230267	C/Yamagata/3/2012		Import from public-domain	Matsuzaki,Y.; Sugawara,K.; Furuse,Y.; Shimotai,Y.; Hongo,S.; Oshitani,H.; Mizuta,K.; Nishimura,H.
EPI816648	NP	Japan	2012-Feb-07	EPI_ISL_230267	C/Yamagata/3/2012		Import from public-domain	Matsuzaki,Y.; Sugawara,K.; Furuse,Y.; Shimotai,Y.; Hongo,S.; Oshitani,H.; Mizuta,K.; Nishimura,H.
EPI816785	NS	Japan	2012-Feb-07	EPI_ISL_230267	C/Yamagata/3/2012		Import from public-domain	Matsuzaki,Y.; Sugawara,K.; Furuse,Y.; Shimotai,Y.; Hongo,S.; Oshitani,H.; Mizuta,K.; Nishimura,H.
EPI816549	P3	Japan	2012-Feb-07	EPI_ISL_230267	C/Yamagata/3/2012		Import from public-domain	Matsuzaki,Y.; Sugawara,K.; Furuse,Y.; Shimotai,Y.; Hongo,S.; Oshitani,H.; Mizuta,K.; Nishimura,H.
EPI816445	PB1	Japan	2012-Feb-07	EPI_ISL_230267	C/Yamagata/3/2012		Import from public-domain	Matsuzaki,Y.; Sugawara,K.; Furuse,Y.; Shimotai,Y.; Hongo,S.; Oshitani,H.; Mizuta,K.; Nishimura,H.
EPI813755	PB2	Japan	2012-Feb-07	EPI_ISL_230267	C/Yamagata/3/2012		Import from public-domain	Matsuzaki,Y.; Sugawara,K.; Furuse,Y.; Shimotai,Y.; Hongo,S.; Oshitani,H.; Mizuta,K.; Nishimura,H.
EPI231956	HE	Japan	1988-Jan-01	EPI_ISL_66421	C/Yamagata/3/88		Import from public-domain	Muraki,Y.
EPI231960	HE	Japan	1996-Jan-01	EPI_ISL_66422	C/Yamagata/3/96		Import from public-domain	Matsuzaki,Y.
EPI231957	MP	Japan	1996-Jan-01	EPI_ISL_66422	C/Yamagata/3/96		Import from public-domain	Matsuzaki,Y.
EPI231962	NS	Japan	1996-Jan-01	EPI_ISL_66422	C/Yamagata/3/96		Import from public-domain	Matsuzaki,Y.
EPI603684	HE	Japan	2014-Jun-04	EPI_ISL_182755	C/Yamagata/30/2014		Import from public-domain	Tanaka,S.; Aoki,Y.; Matoba,Y.; Yahagi,K.; Mizuta,K.; Itagaki,T.; Katsushima,F.; Katsushima,Y.; Matsuzaki,Y.; Sugawara,K.; Furuse,Y.; Shimotai,Y.; Hongo,S.; Oshitani,H.; Mizuta,K.; Nishimura,H.
EPI816727	MP	Japan	2014-Jun-04	EPI_ISL_182755	C/Yamagata/30/2014		Import from public-domain	Tanaka,S.; Aoki,Y.; Matoba,Y.; Yahagi,K.; Mizuta,K.; Itagaki,T.; Katsushima,F.; Katsushima,Y.; Matsuzaki,Y.; Sugawara,K.; Furuse,Y.; Shimotai,Y.; Hongo,S.; Oshitani,H.; Mizuta,K.; Nishimura,H.
EPI816659	NP	Japan	2014-Jun-04	EPI_ISL_182755	C/Yamagata/30/2014		Import from public-domain	Tanaka,S.; Aoki,Y.; Matoba,Y.; Yahagi,K.; Mizuta,K.; Itagaki,T.; Katsushima,F.; Katsushima,Y.; Matsuzaki,Y.; Sugawara,K.; Furuse,Y.; Shimotai,Y.; Hongo,S.; Oshitani,H.; Mizuta,K.; Nishimura,H.
EPI816796	NS	Japan	2014-Jun-04	EPI_ISL_182755	C/Yamagata/30/2014		Import from public-domain	Tanaka,S.; Aoki,Y.; Matoba,Y.; Yahagi,K.; Mizuta,K.; Itagaki,T.; Katsushima,F.; Katsushima,Y.; Matsuzaki,Y.; Sugawara,K.; Furuse,Y.; Shimotai,Y.; Hongo,S.; Oshitani,H.; Mizuta,K.; Nishimura,H.
EPI816560	P3	Japan	2014-Jun-04	EPI_ISL_182755	C/Yamagata/30/2014		Import from public-domain	Tanaka,S.; Aoki,Y.; Matoba,Y.; Yahagi,K.; Mizuta,K.; Itagaki,T.; Katsushima,F.; Katsushima,Y.; Matsuzaki,Y.; Sugawara,K.; Furuse,Y.; Shimotai,Y.; Hongo,S.; Oshitani,H.; Mizuta,K.; Nishimura,H.
EPI816477	PB1	Japan	2014-Jun-04	EPI_ISL_182755	C/Yamagata/30/2014		Import from public-domain	Tanaka,S.; Aoki,Y.; Matoba,Y.; Yahagi,K.; Mizuta,K.; Itagaki,T.; Katsushima,F.; Katsushima,Y.; Matsuzaki,Y.; Sugawara,K.; Furuse,Y.; Shimotai,Y.; Hongo,S.; Oshitani,H.; Mizuta,K.; Nishimura,H.
EPI813774	PB2	Japan	2014-Jun-04	EPI_ISL_182755	C/Yamagata/30/2014		Import from public-domain	Tanaka,S.; Aoki,Y.; Matoba,Y.; Yahagi,K.; Mizuta,K.; Itagaki,T.; Katsushima,F.; Katsushima,Y.; Matsuzaki,Y.; Sugawara,K.; Furuse,Y.; Shimotai,Y.; Hongo,S.; Oshitani,H.; Mizuta,K.; Nishimura,H.
EPI603685	HE	Japan	2014-Jun-17	EPI_ISL_182756	C/Yamagata/32/2014		Import from public-domain	Tanaka,S.; Aoki,Y.; Matoba,Y.; Yahagi,K.; Mizuta,K.; Itagaki,T.; Katsushima,F.; Katsushima,Y.; Matsuzaki,Y.; Sugawara,K.; Furuse,Y.; Shimotai,Y.; Hongo,S.; Oshitani,H.; Mizuta,K.; Nishimura,H.
EPI816728	MP	Japan	2014-Jun-17	EPI_ISL_182756	C/Yamagata/32/2014		Import from public-domain	Tanaka,S.; Aoki,Y.; Matoba,Y.; Yahagi,K.; Mizuta,K.; Itagaki,T.; Katsushima,F.; Katsushima,Y.; Matsuzaki,Y.; Sugawara,K.; Furuse,Y.; Shimotai,Y.; Hongo,S.; Oshitani,H.; Mizuta,K.; Nishimura,H.
EPI816660	NP	Japan	2014-Jun-17	EPI_ISL_182756	C/Yamagata/32/2014		Import from public-domain	Tanaka,S.; Aoki,Y.; Matoba,Y.; Yahagi,K.; Mizuta,K.; Itagaki,T.; Katsushima,F.; Katsushima,Y.; Matsuzaki,Y.; Sugawara,K.; Furuse,Y.; Shimotai,Y.; Hongo,S.; Oshitani,H.; Mizuta,K.; Nishimura,H.
EPI816797	NS	Japan	2014-Jun-17	EPI_ISL_182756	C/Yamagata/32/2014		Import from public-domain	Tanaka,S.; Aoki,Y.; Matoba,Y.; Yahagi,K.; Mizuta,K.; Itagaki,T.; Katsushima,F.; Katsushima,Y.; Matsuzaki,Y.; Sugawara,K.; Furuse,Y.; Shimotai,Y.; Hongo,S.; Oshitani,H.; Mizuta,K.; Nishimura,H.
EPI816561	P3	Japan	2014-Jun-17	EPI_ISL_182756	C/Yamagata/32/2014		Import from public-domain	Tanaka,S.; Aoki,Y.; Matoba,Y.; Yahagi,K.; Mizuta,K.; Itagaki,T.; Katsushima,F.; Katsushima,Y.; Matsuzaki,Y.; Sugawara,K.; Furuse,Y.; Shimotai,Y.; Hongo,S.; Oshitani,H.; Mizuta,K.; Nishimura,H.
EPI816478	PB1	Japan	2014-Jun-17	EPI_ISL_182756	C/Yamagata/32/2014		Import from public-domain	Tanaka,S.; Aoki,Y.; Matoba,Y.; Yahagi,K.; Mizuta,K.; Itagaki,T.; Katsushima,F.; Katsushima,Y.; Matsuzaki,Y.; Sugawara,K.; Furuse,Y.; Shimotai,Y.; Hongo,S.; Oshitani,H.; Mizuta,K.; Nishimura,H.
EPI813776	PB2	Japan	2014-Jun-17	EPI_ISL_182756	C/Yamagata/32/2014		Import from public-domain	Tanaka,S.; Aoki,Y.; Matoba,Y.; Yahagi,K.; Mizuta,K.; Itagaki,T.; Katsushima,F.; Katsushima,Y.; Matsuzaki,Y.; Sugawara,K.; Furuse,Y.; Shimotai,Y.; Hongo,S.; Oshitani,H.; Mizuta,K.; Nishimura,H.
EPI603686	HE	Japan	2014-Jul-22	EPI_ISL_182757	C/Yamagata/33/2014		Import from public-domain	Tanaka,S.; Aoki,Y.; Matoba,Y.; Yahagi,K.; Mizuta,K.; Itagaki,T.; Katsushima,F.; Katsushima,Y.; Matsuzaki,Y.; Sugawara,K.; Furuse,Y.; Shimotai,Y.; Hongo,S.; Oshitani,H.; Mizuta,K.; Nishimura,H.
EPI816729	MP	Japan	2014-Jul-22	EPI_ISL_182757	C/Yamagata/33/2014		Import from public-domain	Tanaka,S.; Aoki,Y.; Matoba,Y.; Yahagi,K.; Mizuta,K.; Itagaki,T.; Katsushima,F.; Katsushima,Y.; Matsuzaki,Y.; Sugawara,K.; Furuse,Y.; Shimotai,Y.; Hongo,S.; Oshitani,H.; Mizuta,K.; Nishimura,H.
EPI816661	NP	Japan	2014-Jul-22	EPI_ISL_182757	C/Yamagata/33/2014		Import from public-domain	Tanaka,S.; Aoki,Y.; Matoba,Y.; Yahagi,K.; Mizuta,K.; Itagaki,T.; Katsushima,F.; Katsushima,Y.; Matsuzaki,Y.; Sugawara,K.; Furuse,Y.; Shimotai,Y.; Hongo,S.; Oshitani,H.; Mizuta,K.; Nishimura,H.
EPI816798	NS	Japan	2014-Jul-22	EPI_ISL_182757	C/Yamagata/33/2014		Import from public-domain	Tanaka,S.; Aoki,Y.; Matoba,Y.; Yahagi,K.; Mizuta,K.; Itagaki,T.; Katsushima,F.; Katsushima,Y.; Matsuzaki,Y.; Sugawara,K.; Furuse,Y.; Shimotai,Y.; Hongo,S.; Oshitani,H.; Mizuta,K.; Nishimura,H.
EPI816562	P3	Japan	2014-Jul-22	EPI_ISL_182757	C/Yamagata/33/2014		Import from public-domain	Tanaka,S.; Aoki,Y.; Matoba,Y.; Yahagi,K.; Mizuta,K.; Itagaki,T.; Katsushima,F.; Katsushima,Y.; Matsuzaki,Y.; Sugawara,K.; Furuse,Y.; Shimotai,Y.; Hongo,S.; Oshitani,H.; Mizuta,K.; Nishimura,H.
EPI816479	PB1	Japan	2014-Jul-22	EPI_ISL_182757	C/Yamagata/33/2014		Import from public-domain	Tanaka,S.; Aoki,Y.; Matoba,Y.; Yahagi,K.; Mizuta,K.; Itagaki,T.; Katsushima,F.; Katsushima,Y.; Matsuzaki,Y.; Sugawara,K.; Furuse,Y.; Shimotai,Y.; Hongo,S.; Oshitani,H.; Mizuta,K.; Nishimura,H.
EPI813777	PB2	Japan	2014-Jul-22	EPI_ISL_182757	C/Yamagata/33/2014		Import from public-domain	Tanaka,S.; Aoki,Y.; Matoba,Y.; Yahagi,K.; Mizuta,K.; Itagaki,T.; Katsushima,F.; Katsushima,Y.; Matsuzaki,Y.; Sugawara,K.; Furuse,Y.; Shimotai,Y.; Hongo,S.; Oshitani,H.; Mizuta,K.; Nishimura,H.
EPI603687	HE	Japan	2014-Aug-20	EPI_ISL_182758	C/Yamagata/35/2014		Import from public-domain	Tanaka,S.; Aoki,Y.; Matoba,Y.; Yahagi,K.; Mizuta,K.; Itagaki,T.; Katsushima,F.; Katsushima,Y.; Matsuzaki,Y.; Sugawara,K.; Furuse,Y.; Shimotai,Y.; Hongo,S.; Oshitani,H.; Mizuta,K.; Nishimura,H.
EPI816730	MP	Japan	2014-Aug-20	EPI_ISL_182758	C/Yamagata/35/2014		Import from public-domain	Tanaka,S.; Aoki,Y.; Matoba,Y.; Yahagi,K.; Mizuta,K.; Itagaki,T.; Katsushima,F.; Katsushima,Y.; Matsuzaki,Y.; Sugawara,K.; Furuse,Y.; Shimotai,Y.; Hongo,S.; Oshitani,H.; Mizuta,K.; Nishimura,H.
EPI816662	NP	Japan	2014-Aug-20	EPI_ISL_182758	C/Yamagata/35/2014		Import from public-domain	Tanaka,S.; Aoki,Y.; Matoba,Y.; Yahagi,K.; Mizuta,K.; Itagaki,T.; Katsushima,F.; Katsushima,Y.; Matsuzaki,Y.; Sugawara,K.; Furuse,Y.; Shimotai,Y.; Hongo,S.; Oshitani,H.; Mizuta,K.; Nishimura,H.
EPI816799	NS	Japan	2014-Aug-20	EPI_ISL_182758	C/Yamagata/35/2014		Import from public-domain	Tanaka,S.; Aoki,Y.; Matoba,Y.; Yahagi,K.; Mizuta,K.; Itagaki,T.; Katsushima,F.; Katsushima,Y.; Matsuzaki,Y.; Sugawara,K.; Furuse,Y.; Shimotai,Y.; Hongo,S.; Oshitani,H.; Mizuta,K.; Nishimura,H.
EPI816563	P3	Japan	2014-Aug-20	EPI_ISL_182758	C/Yamagata/35/2014		Import from public-domain	Tanaka,S.; Aoki,Y.; Matoba,Y.; Yahagi,K.; Mizuta,K.; Itagaki,T.; Katsushima,F.; Katsushima,Y.; Matsuzaki,Y.; Sugawara,K.; Furuse,Y.; Shimotai,Y.; Hongo,S.; Oshitani,H.; Mizuta,K.; Nishimura,H.
EPI816480	PB1	Japan	2014-Aug-20	EPI_ISL_182758	C/Yamagata/35/2014		Import from public-domain	Tanaka,S.; Aoki,Y.; Matoba,Y.; Yahagi,K.; Mizuta,K.; Itagaki,T.; Katsushima,F.; Katsushima,Y.; Matsuzaki,Y.; Sugawara,K.; Furuse,Y.; Shimotai,Y.; Hongo,S.; Oshitani,H.; Mizuta,K.; Nishimura,H.
EPI813778	PB2	Japan	2014-Aug-20	EPI_ISL_182758	C/Yamagata/35/2014		Import from public-domain	Tanaka,S.; Aoki,Y.; Matoba,Y.; Yahagi,K.; Mizuta,K.; Itagaki,T.; Katsushima,F.; Katsushima,Y.; Matsuzaki,Y.; Sugawara,K.; Furuse,Y.; Shimotai,Y.; Hongo,S.; Oshitani,H.; Mizuta,K.; Nishimura,H.
EPI816717	MP	Japan	2012-Feb-22	EPI_ISL_230284	C/Yamagata/4/2012		Import from public-domain	Matsuzaki,Y.; Sugawara,K.; Furuse,Y.; Shimotai,Y.; Hongo,S.; Oshitani,H.; Mizuta,K.; Nishimura,H.
EPI816649	NP	Japan	2012-Feb-22	EPI_ISL_230284	C/Yamagata/4/2012		Import from public-domain	Matsuzaki,Y.; Sugawara,K.; Furuse,Y.; Shimotai,Y.; Hongo,S.; Oshitani,H.; Mizuta,K.; Nishimura,H.
EPI816786	NS	Japan	2012-Feb-22	EPI_ISL_230284	C/Yamagata/4/2012		Import from public-domain	Matsuzaki,Y.; Sugawara,K.; Furuse,Y.; Shimotai,Y.; Hongo,S.; Oshitani,H.; Mizuta,K.; Nishimura,H.
EPI816550	P3	Japan	2012-Feb-22	EPI_ISL_230284	C/Yamagata/4/2012		Import from public-domain	Matsuzaki,Y.; Sugawara,K.; Furuse,Y.; Shimotai,Y.; Hongo,S.; Oshitani,H.; Mizuta,K.; Nishimura,H.
EPI816446	PB1	Japan	2012-Feb-22	EPI_ISL_230284	C/Yamagata/4/2012		Import from public-domain	Matsuzaki,Y.; Sugawara,K.; Furuse,Y.; Shimotai,Y.; Hongo,S.; Oshitani,H.; Mizuta,K.; Nishimura,H.
EPI813756	PB2	Japan	2012-Feb-22	EPI_ISL_230284	C/Yamagata/4/2012		Import from public-domain	Matsuzaki,Y.; Sugawara,K.; Furuse,Y.; Shimotai,Y.; Hongo,S.; Oshitani,H.; Mizuta,K.; Nishimura,H.
EPI231964	HE	Japan	1988-Jan-01	EPI_ISL_66423	C/Yamagata/4/88		Import from public-domain	Umetsu,Y.
EPI231965	HE	Japan	1989-Jan-01	EPI_ISL_66424	C/Yamagata/4/89		Import from public-domain	Asahi,Y.; Sugawara,K.; Muraki,Y.; Hongo,S.; Kitame,F.; Nakamura,K.
EPI816674	MP	Japan	1992-Nov-19	EPI_ISL_230280	C/Yamagata/4/92		Import from public-domain	Matsuzaki,Y.; Sugawara,K.; Furuse,Y.; Shimotai,Y.; Hongo,S.; Oshitani,H.; Mizuta,K.; Nishimura,H.
EPI816595	NP	Japan	1992-Nov-19	EPI_ISL_230280	C/Yamagata/4/92		Import from public-domain	Matsuzaki,Y.; Sugawara,K.; Furuse,Y.; Shimotai,Y.; Hongo,S.; Oshitani,H.; Mizuta,K.; Nishimura,H.
EPI816744	NS	Japan	1992-Nov-19	EPI_ISL_230280	C/Yamagata/4/92		Import from public-domain	Matsuzaki,Y.; Sugawara,K.; Furuse,Y.; Shimotai,Y.; Hongo,S.; Oshitani,H.; Mizuta,K.; Nishimura,H.
EPI816496	P3	Japan	1992-Nov-19	EPI_ISL_230280	C/Yamagata/4/92		Import from public-domain	Matsuzaki,Y.; Sugawara,K.; Furuse,Y.; Shimotai,Y.; Hongo,S.; Oshitani,H.; Mizuta,K.; Nishimura,H.
EPI813795	PB1	Japan	1992-Nov-19	EPI_ISL_230280	C/Yamagata/4/92		Import from public-domain	Matsuzaki,Y.; Sugawara,K.; Furuse,Y.; Shimotai,Y.; Hongo,S.; Oshitani,H.; Mizuta,K.; Nishimura,H.
EPI813698	PB2	Japan	1992-Nov-19	EPI_ISL_230280	C/Yamagata/4/92		Import from public-domain	Matsuzaki,Y.; Sugawara,K.; Furuse,Y.; Shimotai,Y.; Hongo,S.; Oshitani,H.; Mizuta,K.; Nishimura,H.
EPI813652	HE	Japan	2002-Feb-07	EPI_ISL_230237	C/Yamagata/5/2002		Import from public-domain	Matsuzaki,Y.; Sugawara,K.; Furuse,Y.; Shimotai,Y.; Hongo,S.; Oshitani,H.; Mizuta,K.; Nishimura,H.
EPI816676	MP	Japan	2002-Feb-07	EPI_ISL_230237	C/Yamagata/5/2002		Import from public-domain	Matsuzaki,Y.; Sugawara,K.; Furuse,Y.; Shimotai,Y.; Hongo,S.; Oshitani,H.; Mizuta,K.; Nishimura,H.
EPI816613	NP	Japan	2002-Feb-07	EPI_ISL_230237	C/Yamagata/5/2002		Import from public-domain	Matsuzaki,Y.; Sugawara,K.; Furuse,Y.; Shimotai,Y.; Hongo,S.; Oshitani,H.; Mizuta,K.; Nishimura,H.
EPI816745	NS	Japan	2002-Feb-07	EPI_ISL_230237	C/Yamagata/5/2002		Import from public-domain	Matsuzaki,Y.; Sugawara,K.; Furuse,Y.; Shimotai,Y.; Hongo,S.; Oshitani,H.; Mizuta,K.; Nishimura,H.
EPI816509	P3	Japan	2002-Feb-07	EPI_ISL_230237	C/Yamagata/5/2002		Import from public-domain	Matsuzaki,Y.; Sugawara,K.; Furuse,Y.; Shimotai,Y.; Hongo,S.; Oshitani,H.; Mizuta,K.; Nishimura,H.
EPI813796	PB1	Japan	2002-Feb-07	EPI_ISL_230237	C/Yamagata/5/2002		Import from public-domain	Matsuzaki,Y.; Sugawara,K.; Furuse,Y.; Shimotai,Y.; Hongo,S.; Oshitani,H.; Mizuta,K.; Nishimura,H.
EPI813699	PB2	Japan	2002-Feb-07	EPI_ISL_230237	C/Yamagata/5/2002		Import from public-domain	Matsuzaki,Y.; Sugawara,K.; Furuse,Y.; Shimotai,Y.; Hongo,S.; Oshitani,H.; Mizuta,K.; Nishimura,H.
EPI231570	NS	Japan	1989-Jan-01	EPI_ISL_66359	C/Yamagata/5/89		Import from public-domain	Matsuzaki,Y.
EPI232176	HE	Japan	1992-Nov-26	EPI_ISL_66361	C/Yamagata/5/92		Import from public-domain	Matsuzaki,Y.; Sugawara,K.; Furuse,Y.; Shimotai,Y.; Hongo,S.; Oshitani,H.; Mizuta,K.; Nishimura,H.
EPI816675	MP	Japan	1992-Nov-26	EPI_ISL_66361	C/Yamagata/5/92		Import from public-domain	Matsuzaki,Y.; Sugawara,K.; Furuse,Y.; Shimotai,Y.; Hongo,S.; Oshitani,H.; Mizuta,K.; Nishimura,H.
EPI816596	NP	Japan	1992-Nov-26	EPI_ISL_66361	C/Yamagata/5/92		Import from public-domain	Matsuzaki,Y.; Sugawara,K.; Furuse,Y.; Shimotai,Y.; Hongo,S.; Oshitani,H.; Mizuta,K.; Nishimura,H.
EPI231572	NS	Japan	1992-Nov-26	EPI_ISL_66361	C/Yamagata/5/92		Import from public-domain	Matsuzaki,Y.; Sugawara,K.; Furuse,Y.; Shimotai,Y.; Hongo,S.; Oshitani,H.; Mizuta,K.; Nishimura,H.
EPI816497	P3	Japan	1992-Nov-26	EPI_ISL_66361	C/Yamagata/5/92		Import from public-domain	Matsuzaki,Y.; Sugawara,K.; Furuse,Y.; Shimotai,Y.; Hongo,S.; Oshitani,H.; Mizuta,K.; Nishimura,H.
EPI816414	PB1	Japan	1992-Nov-26	EPI_ISL_66361	C/Yamagata/5/92		Import from public-domain	Matsuzaki,Y.; Sugawara,K.; Furuse,Y.; Shimotai,Y.; Hongo,S.; Oshitani,H.; Mizuta,K.; Nishimura,H.
EPI813740	PB2	Japan	1992-Nov-26	EPI_ISL_66361	C/Yamagata/5/92		Import from public-domain	Matsuzaki,Y.; Sugawara,K.; Furuse,Y.; Shimotai,Y.; Hongo,S.; Oshitani,H.; Mizuta,K.; Nishimura,H.
EPI231971	HE	Japan	2000-Mar-21	EPI_ISL_66425	C/Yamagata/6/2000		Import from public-domain	Matsuzaki,Y.; Sugawara,K.; Furuse,Y.; Shimotai,Y.; Hongo,S.; Oshitani,H.; Mizuta,K.; Nishimura,H.
EPI231968	MP	Japan	2000-Mar-21	EPI_ISL_66425	C/Yamagata/6/2000		Import from public-domain	Matsuzaki,Y.; Sugawara,K.; Furuse,Y.; Shimotai,Y.; Hongo,S.; Oshitani,H.; Mizuta,K.; Nishimura,H.
EPI816612	NP	Japan	2000-Mar-21	EPI_ISL_66425	C/Yamagata/6/2000		Import from public-domain	Matsuzaki,Y.; Sugawara,K.; Furuse,Y.; Shimotai,Y.; Hongo,S.; Oshitani,H.; Mizuta,K.; Nishimura,H.
EPI231972	NS	Japan	2000-Mar-21	EPI_ISL_66425	C/Yamagata/6/2000		Import from public-domain	Matsuzaki,Y.; Sugawara,K.; Furuse,Y.; Shimotai,Y.; Hongo,S.; Oshitani,H.; Mizuta,K.; Nishimura,H.
EPI816508	P3	Japan	2000-Mar-21	EPI_ISL_66425	C/Yamagata/6/2000		Import from public-domain	Matsuzaki,Y.; Sugawara,K.; Furuse,Y.; Shimotai,Y.; Hongo,S.; Oshitani,H.; Mizuta,K.; Nishimura,H.
EPI816425	PB1	Japan	2000-Mar-21	EPI_ISL_66425	C/Yamagata/6/2000		Import from public-domain	Matsuzaki,Y.; Sugawara,K.; Furuse,Y.; Shimotai,Y.; Hongo,S.; Oshitani,H.; Mizuta,K.; Nishimura,H.
EPI813735	PB2	Japan	2000-Mar-21	EPI_ISL_66425	C/Yamagata/6/2000		Import from public-domain	Matsuzaki,Y.; Sugawara,K.; Furuse,Y.; Shimotai,Y.; Hongo,S.; Oshitani,H.; Mizuta,K.; Nishimura,H.
EPI603679	HE	Japan	2014-Apr-16	EPI_ISL_182750	C/Yamagata/6/2014		Import from public-domain	Tanaka,S.; Aoki,Y.; Matoba,Y.; Yahagi,K.; Mizuta,K.; Itagaki,T.; Katsushima,F.; Katsushima,Y.; Matsuzaki,Y.; Sugawara,K.; Furuse,Y.; Shimotai,Y.; Hongo,S.; Oshitani,H.; Mizuta,K.; Nishimura,H.

Segment ID	Segment	Country	Collection date	Isolate-ID	Isolate name	Originating Lab	Submitting Lab	Authors
EPI816724	MP	Japan	2014-Apr-16	EPI_ISL_182750	C/Yamagata/6/2014		Import from public-domain	Tanaka,S.; Aoki,Y.; Matoba,Y.; Yahagi,K.; Mizuta,K.; Itagaki,T.; Katsushima,F.; Katsushima,Y.; Matsuzaki,Y.; Sugawara,K.; Furuse,Y.; Shimotai,Y.; Hongo,S.; Oshitani,H.; Mizuta,K.; Nishimura,H.
EPI816656	NP	Japan	2014-Apr-16	EPI_ISL_182750	C/Yamagata/6/2014		Import from public-domain	Tanaka,S.; Aoki,Y.; Matoba,Y.; Yahagi,K.; Mizuta,K.; Itagaki,T.; Katsushima,F.; Katsushima,Y.; Matsuzaki,Y.; Sugawara,K.; Furuse,Y.; Shimotai,Y.; Hongo,S.; Oshitani,H.; Mizuta,K.; Nishimura,H.
EPI816793	NS	Japan	2014-Apr-16	EPI_ISL_182750	C/Yamagata/6/2014		Import from public-domain	Tanaka,S.; Aoki,Y.; Matoba,Y.; Yahagi,K.; Mizuta,K.; Itagaki,T.; Katsushima,F.; Katsushima,Y.; Matsuzaki,Y.; Sugawara,K.; Furuse,Y.; Shimotai,Y.; Hongo,S.; Oshitani,H.; Mizuta,K.; Nishimura,H.
EPI816557	P3	Japan	2014-Apr-16	EPI_ISL_182750	C/Yamagata/6/2014		Import from public-domain	Tanaka,S.; Aoki,Y.; Matoba,Y.; Yahagi,K.; Mizuta,K.; Itagaki,T.; Katsushima,F.; Katsushima,Y.; Matsuzaki,Y.; Sugawara,K.; Furuse,Y.; Shimotai,Y.; Hongo,S.; Oshitani,H.; Mizuta,K.; Nishimura,H.
EPI816474	PB1	Japan	2014-Apr-16	EPI_ISL_182750	C/Yamagata/6/2014		Import from public-domain	Tanaka,S.; Aoki,Y.; Matoba,Y.; Yahagi,K.; Mizuta,K.; Itagaki,T.; Katsushima,F.; Katsushima,Y.; Matsuzaki,Y.; Sugawara,K.; Furuse,Y.; Shimotai,Y.; Hongo,S.; Oshitani,H.; Mizuta,K.; Nishimura,H.
EPI813772	PB2	Japan	2014-Apr-16	EPI_ISL_182750	C/Yamagata/6/2014		Import from public-domain	Tanaka,S.; Aoki,Y.; Matoba,Y.; Yahagi,K.; Mizuta,K.; Itagaki,T.; Katsushima,F.; Katsushima,Y.; Matsuzaki,Y.; Sugawara,K.; Furuse,Y.; Shimotai,Y.; Hongo,S.; Oshitani,H.; Mizuta,K.; Nishimura,H.
EPI231978	HE	Japan	1998-Jan-01	EPI_ISL_66426	C/Yamagata/6/98		Import from public-domain	Matsuzaki,Y.
EPI231975	MP	Japan	1998-Jan-01	EPI_ISL_66426	C/Yamagata/6/98		Import from public-domain	Matsuzaki,Y.
EPI231976	NS	Japan	1998-Jan-01	EPI_ISL_66426	C/Yamagata/6/98		Import from public-domain	Matsuzaki,Y.
EPI232180	HE	Japan	1964-Jan-01	EPI_ISL_66319	C/Yamagata/64		Import from public-domain	Matsuzaki,Y.; Sugawara,K.; Furuse,Y.; Shimotai,Y.; Hongo,S.; Oshitani,H.; Mizuta,K.; Nishimura,H.
EPI232179	MP	Japan	1964-Jan-01	EPI_ISL_66319	C/Yamagata/64		Import from public-domain	Matsuzaki,Y.; Sugawara,K.; Furuse,Y.; Shimotai,Y.; Hongo,S.; Oshitani,H.; Mizuta,K.; Nishimura,H.
EPI816571	NP	Japan	1964-Jan-01	EPI_ISL_66319	C/Yamagata/64		Import from public-domain	Matsuzaki,Y.; Sugawara,K.; Furuse,Y.; Shimotai,Y.; Hongo,S.; Oshitani,H.; Mizuta,K.; Nishimura,H.
EPI231517	NS	Japan	1964-Jan-01	EPI_ISL_66319	C/Yamagata/64		Import from public-domain	Matsuzaki,Y.; Sugawara,K.; Furuse,Y.; Shimotai,Y.; Hongo,S.; Oshitani,H.; Mizuta,K.; Nishimura,H.
EPI816460	P3	Japan	1964-Jan-01	EPI_ISL_66319	C/Yamagata/64		Import from public-domain	Matsuzaki,Y.; Sugawara,K.; Furuse,Y.; Shimotai,Y.; Hongo,S.; Oshitani,H.; Mizuta,K.; Nishimura,H.
EPI813779	PB1	Japan	1964-Jan-01	EPI_ISL_66319	C/Yamagata/64		Import from public-domain	Matsuzaki,Y.; Sugawara,K.; Furuse,Y.; Shimotai,Y.; Hongo,S.; Oshitani,H.; Mizuta,K.; Nishimura,H.
EPI814696	PB2	Japan	1964-Jan-01	EPI_ISL_66319	C/Yamagata/64		Import from public-domain	Matsuzaki,Y.; Sugawara,K.; Furuse,Y.; Shimotai,Y.; Hongo,S.; Oshitani,H.; Mizuta,K.; Nishimura,H.
EPI816677	MP	Japan	2002-Feb-16	EPI_ISL_230281	C/Yamagata/7/2002		Import from public-domain	Matsuzaki,Y.; Sugawara,K.; Furuse,Y.; Shimotai,Y.; Hongo,S.; Oshitani,H.; Mizuta,K.; Nishimura,H.
EPI816614	NP	Japan	2002-Feb-16	EPI_ISL_230281	C/Yamagata/7/2002		Import from public-domain	Matsuzaki,Y.; Sugawara,K.; Furuse,Y.; Shimotai,Y.; Hongo,S.; Oshitani,H.; Mizuta,K.; Nishimura,H.
EPI816746	NS	Japan	2002-Feb-16	EPI_ISL_230281	C/Yamagata/7/2002		Import from public-domain	Matsuzaki,Y.; Sugawara,K.; Furuse,Y.; Shimotai,Y.; Hongo,S.; Oshitani,H.; Mizuta,K.; Nishimura,H.
EPI816510	P3	Japan	2002-Feb-16	EPI_ISL_230281	C/Yamagata/7/2002		Import from public-domain	Matsuzaki,Y.; Sugawara,K.; Furuse,Y.; Shimotai,Y.; Hongo,S.; Oshitani,H.; Mizuta,K.; Nishimura,H.
EPI813797	PB1	Japan	2002-Feb-16	EPI_ISL_230281	C/Yamagata/7/2002		Import from public-domain	Matsuzaki,Y.; Sugawara,K.; Furuse,Y.; Shimotai,Y.; Hongo,S.; Oshitani,H.; Mizuta,K.; Nishimura,H.
EPI813700	PB2	Japan	2002-Feb-16	EPI_ISL_230281	C/Yamagata/7/2002		Import from public-domain	Matsuzaki,Y.; Sugawara,K.; Furuse,Y.; Shimotai,Y.; Hongo,S.; Oshitani,H.; Mizuta,K.; Nishimura,H.
EPI813683	HE	Japan	2012-Mar-14	EPI_ISL_230268	C/Yamagata/7/2012		Import from public-domain	Matsuzaki,Y.; Sugawara,K.; Furuse,Y.; Shimotai,Y.; Hongo,S.; Oshitani,H.; Mizuta,K.; Nishimura,H.
EPI816718	MP	Japan	2012-Mar-14	EPI_ISL_230268	C/Yamagata/7/2012		Import from public-domain	Matsuzaki,Y.; Sugawara,K.; Furuse,Y.; Shimotai,Y.; Hongo,S.; Oshitani,H.; Mizuta,K.; Nishimura,H.
EPI816650	NP	Japan	2012-Mar-14	EPI_ISL_230268	C/Yamagata/7/2012		Import from public-domain	Matsuzaki,Y.; Sugawara,K.; Furuse,Y.; Shimotai,Y.; Hongo,S.; Oshitani,H.; Mizuta,K.; Nishimura,H.
EPI816787	NS	Japan	2012-Mar-14	EPI_ISL_230268	C/Yamagata/7/2012		Import from public-domain	Matsuzaki,Y.; Sugawara,K.; Furuse,Y.; Shimotai,Y.; Hongo,S.; Oshitani,H.; Mizuta,K.; Nishimura,H.
EPI816551	P3	Japan	2012-Mar-14	EPI_ISL_230268	C/Yamagata/7/2012		Import from public-domain	Matsuzaki,Y.; Sugawara,K.; Furuse,Y.; Shimotai,Y.; Hongo,S.; Oshitani,H.; Mizuta,K.; Nishimura,H.
EPI816447	PB1	Japan	2012-Mar-14	EPI_ISL_230268	C/Yamagata/7/2012		Import from public-domain	Matsuzaki,Y.; Sugawara,K.; Furuse,Y.; Shimotai,Y.; Hongo,S.; Oshitani,H.; Mizuta,K.; Nishimura,H.
EPI813757	PB2	Japan	2012-Mar-14	EPI_ISL_230268	C/Yamagata/7/2012		Import from public-domain	Matsuzaki,Y.; Sugawara,K.; Furuse,Y.; Shimotai,Y.; Hongo,S.; Oshitani,H.; Mizuta,K.; Nishimura,H.
EPI816672	MP	Japan	1981-Mar-18	EPI_ISL_230278	C/Yamagata/7/81		Import from public-domain	Matsuzaki,Y.; Sugawara,K.; Furuse,Y.; Shimotai,Y.; Hongo,S.; Oshitani,H.; Mizuta,K.; Nishimura,H.
EPI816584	NP	Japan	1981-Mar-18	EPI_ISL_230278	C/Yamagata/7/81		Import from public-domain	Matsuzaki,Y.; Sugawara,K.; Furuse,Y.; Shimotai,Y.; Hongo,S.; Oshitani,H.; Mizuta,K.; Nishimura,H.
EPI816741	NS	Japan	1981-Mar-18	EPI_ISL_230278	C/Yamagata/7/81		Import from public-domain	Matsuzaki,Y.; Sugawara,K.; Furuse,Y.; Shimotai,Y.; Hongo,S.; Oshitani,H.; Mizuta,K.; Nishimura,H.
EPI816485	P3	Japan	1981-Mar-18	EPI_ISL_230278	C/Yamagata/7/81		Import from public-domain	Matsuzaki,Y.; Sugawara,K.; Furuse,Y.; Shimotai,Y.; Hongo,S.; Oshitani,H.; Mizuta,K.; Nishimura,H.
EPI813792	PB1	Japan	1981-Mar-18	EPI_ISL_230278	C/Yamagata/7/81		Import from public-domain	Matsuzaki,Y.; Sugawara,K.; Furuse,Y.; Shimotai,Y.; Hongo,S.; Oshitani,H.; Mizuta,K.; Nishimura,H.
EPI813695	PB2	Japan	1981-Mar-18	EPI_ISL_230278	C/Yamagata/7/81		Import from public-domain	Matsuzaki,Y.; Sugawara,K.; Furuse,Y.; Shimotai,Y.; Hongo,S.; Oshitani,H.; Mizuta,K.; Nishimura,H.
EPI231558	HE	Japan	1988-Nov-03	EPI_ISL_66349	C/Yamagata/7/88		Import from public-domain	Matsuzaki,Y.; Sugawara,K.; Furuse,Y.; Shimotai,Y.; Hongo,S.; Oshitani,H.; Mizuta,K.; Nishimura,H.
EPI231980	MP	Japan	1988-Nov-03	EPI_ISL_66349	C/Yamagata/7/88		Import from public-domain	Matsuzaki,Y.; Sugawara,K.; Furuse,Y.; Shimotai,Y.; Hongo,S.; Oshitani,H.; Mizuta,K.; Nishimura,H.
EPI816591	NP	Japan	1988-Nov-03	EPI_ISL_66349	C/Yamagata/7/88		Import from public-domain	Matsuzaki,Y.; Sugawara,K.; Furuse,Y.; Shimotai,Y.; Hongo,S.; Oshitani,H.; Mizuta,K.; Nishimura,H.
EPI231563	NS	Japan	1988-Nov-03	EPI_ISL_66349	C/Yamagata/7/88		Import from public-domain	Matsuzaki,Y.; Sugawara,K.; Furuse,Y.; Shimotai,Y.; Hongo,S.; Oshitani,H.; Mizuta,K.; Nishimura,H.
EPI816492	P3	Japan	1988-Nov-03	EPI_ISL_66349	C/Yamagata/7/88		Import from public-domain	Matsuzaki,Y.; Sugawara,K.; Furuse,Y.; Shimotai,Y.; Hongo,S.; Oshitani,H.; Mizuta,K.; Nishimura,H.
EPI816410	PB1	Japan	1988-Nov-03	EPI_ISL_66349	C/Yamagata/7/88		Import from public-domain	Matsuzaki,Y.; Sugawara,K.; Furuse,Y.; Shimotai,Y.; Hongo,S.; Oshitani,H.; Mizuta,K.; Nishimura,H.
EPI813722	PB2	Japan	1988-Nov-03	EPI_ISL_66349	C/Yamagata/7/88		Import from public-domain	Matsuzaki,Y.; Sugawara,K.; Furuse,Y.; Shimotai,Y.; Hongo,S.; Oshitani,H.; Mizuta,K.; Nishimura,H.
EPI231981	HE	Japan	2000-Jan-01	EPI_ISL_66427	C/Yamagata/8/2000		Import from public-domain	Matsuzaki,Y.
EPI231984	MP	Japan	2000-Jan-01	EPI_ISL_66427	C/Yamagata/8/2000		Import from public-domain	Matsuzaki,Y.
EPI231987	NS	Japan	2000-Jan-01	EPI_ISL_66427	C/Yamagata/8/2000		Import from public-domain	Matsuzaki,Y.
EPI231559	HE	Japan	1988-Jan-01	EPI_ISL_66350	C/Yamagata/8/88		Import from public-domain	Matsuzaki,Y.
EPI232187	MP	Japan	1988-Jan-01	EPI_ISL_66350	C/Yamagata/8/88		Import from public-domain	Matsuzaki,Y.
EPI231991	HE	Japan	1996-Jun-13	EPI_ISL_66428	C/Yamagata/8/96		Import from public-domain	Matsuzaki,Y.
EPI231994	MP	Japan	1996-Jun-13	EPI_ISL_66428	C/Yamagata/8/96		Import from public-domain	Matsuzaki,Y.
EPI816609	NP	Japan	1996-Jun-13	EPI_ISL_66428	C/Yamagata/8/96		Import from public-domain	Matsuzaki,Y.
EPI231989	NS	Japan	1996-Jun-13	EPI_ISL_66428	C/Yamagata/8/96		Import from public-domain	Matsuzaki,Y.
EPI816504	P3	Japan	1996-Jun-13	EPI_ISL_66428	C/Yamagata/8/96		Import from public-domain	Matsuzaki,Y.
EPI816421	PB1	Japan	1996-Jun-13	EPI_ISL_66428	C/Yamagata/8/96		Import from public-domain	Matsuzaki,Y.
EPI813723	PB2	Japan	1996-Jun-13	EPI_ISL_66428	C/Yamagata/8/96		Import from public-domain	Matsuzaki,Y.
EPI232000	HE	Japan	2000-Jan-01	EPI_ISL_66429	C/Yamagata/9/2000		Import from public-domain	Matsuzaki,Y.
EPI231996	MP	Japan	2000-Jan-01	EPI_ISL_66429	C/Yamagata/9/2000		Import from public-domain	Matsuzaki,Y.
EPI232001	NS	Japan	2000-Jan-01	EPI_ISL_66429	C/Yamagata/9/2000		Import from public-domain	Matsuzaki,Y.
EPI813670	HE	Japan	2006-Jun-13	EPI_ISL_230255	C/Yamagata/9/2006		Import from public-domain	Matsuzaki,Y.; Sugawara,K.; Furuse,Y.; Shimotai,Y.; Hongo,S.; Oshitani,H.; Mizuta,K.; Nishimura,H.
EPI816701	MP	Japan	2006-Jun-13	EPI_ISL_230255	C/Yamagata/9/2006		Import from public-domain	Matsuzaki,Y.; Sugawara,K.; Furuse,Y.; Shimotai,Y.; Hongo,S.; Oshitani,H.; Mizuta,K.; Nishimura,H.
EPI816635	NP	Japan	2006-Jun-13	EPI_ISL_230255	C/Yamagata/9/2006		Import from public-domain	Matsuzaki,Y.; Sugawara,K.; Furuse,Y.; Shimotai,Y.; Hongo,S.; Oshitani,H.; Mizuta,K.; Nishimura,H.
EPI816770	NS	Japan	2006-Jun-13	EPI_ISL_230255	C/Yamagata/9/2006		Import from public-domain	Matsuzaki,Y.; Sugawara,K.; Furuse,Y.; Shimotai,Y.; Hongo,S.; Oshitani,H.; Mizuta,K.; Nishimura,H.
EPI816534	P3	Japan	2006-Jun-13	EPI_ISL_230255	C/Yamagata/9/2006		Import from public-domain	Matsuzaki,Y.; Sugawara,K.; Furuse,Y.; Shimotai,Y.; Hongo,S.; Oshitani,H.; Mizuta,K.; Nishimura,H.
EPI816432	PB1	Japan	2006-Jun-13	EPI_ISL_230255	C/Yamagata/9/2006		Import from public-domain	Matsuzaki,Y.; Sugawara,K.; Furuse,Y.; Shimotai,Y.; Hongo,S.; Oshitani,H.; Mizuta,K.; Nishimura,H.
EPI813719	PB2	Japan	2006-Jun-13	EPI_ISL_230255	C/Yamagata/9/2006		Import from public-domain	Matsuzaki,Y.; Sugawara,K.; Furuse,Y.; Shimotai,Y.; Hongo,S.; Oshitani,H.; Mizuta,K.; Nishimura,H.
EPI231560	HE	Japan	1988-Dec-12	EPI_ISL_66351	C/Yamagata/9/88		Import from public-domain	Matsuzaki,Y.; Sugawara,K.; Furuse,Y.; Shimotai,Y.; Hongo,S.; Oshitani,H.; Mizuta,K.; Nishimura,H.
EPI232188	MP	Japan	1988-Dec-12	EPI_ISL_66351	C/Yamagata/9/88		Import from public-domain	Matsuzaki,Y.; Sugawara,K.; Furuse,Y.; Shimotai,Y.; Hongo,S.; Oshitani,H.; Mizuta,K.; Nishimura,H.
EPI816592	NP	Japan	1988-Dec-12	EPI_ISL_66351	C/Yamagata/9/88		Import from public-domain	Matsuzaki,Y.; Sugawara,K.; Furuse,Y.; Shimotai,Y.; Hongo,S.; Oshitani,H.; Mizuta,K.; Nishimura,H.
EPI231564	NS	Japan	1988-Dec-12	EPI_ISL_66351	C/Yamagata/9/88		Import from public-domain	Matsuzaki,Y.; Sugawara,K.; Furuse,Y.; Shimotai,Y.; Hongo,S.; Oshitani,H.; Mizuta,K.; Nishimura,H.
EPI816493	P3	Japan	1988-Dec-12	EPI_ISL_66351	C/Yamagata/9/88		Import from public-domain	Matsuzaki,Y.; Sugawara,K.; Furuse,Y.; Shimotai,Y.; Hongo,S.; Oshitani,H.; Mizuta,K.; Nishimura,H.
EPI816411	PB1	Japan	1988-Dec-12	EPI_ISL_66351	C/Yamagata/9/88		Import from public-domain	Matsuzaki,Y.; Sugawara,K.; Furuse,Y.; Shimotai,Y.; Hongo,S.; Oshitani,H.; Mizuta,K.; Nishimura,H.
EPI813725	PB2	Japan	1988-Dec-12	EPI_ISL_66351	C/Yamagata/9/88		Import from public-domain	Matsuzaki,Y.; Sugawara,K.; Furuse,Y.; Shimotai,Y.; Hongo,S.; Oshitani,H.; Mizuta,K.; Nishimura,H.
EPI232005	HE	Japan	1996-Jan-01	EPI_ISL_66430	C/Yamagata/9/96		Import from public-domain	Matsuzaki,Y.
EPI232002	MP	Japan	1996-Jan-01	EPI_ISL_66430	C/Yamagata/9/96		Import from public-domain	Matsuzaki,Y.
EPI232007	NS	Japan	1996-Jan-01	EPI_ISL_66430	C/Yamagata/9/96		Import from public-domain	Matsuzaki,Y.
